# Targeting the lncRNA DUXAP8/miR-29a/*PIK3CA* Network Restores Doxorubicin Chemosensitivity *via* PI3K-AKT-mTOR Signaling and Synergizes With Inotuzumab Ozogamicin in Chemotherapy-Resistant B-Cell Acute Lymphoblastic Leukemia

**DOI:** 10.3389/fonc.2022.773601

**Published:** 2022-03-02

**Authors:** Li Zhang, Shixia Zhou, Tiejun Zhou, Xiaoming Li, Junling Tang

**Affiliations:** ^1^ Department of Oral and Maxillofacial Surgery, The Affiliated Stomatology Hospital of Southwest Medical University, Luzhou, China; ^2^ Orofacial Reconstruction and Regeneration Laboratory, The Affiliated Stomatology Hospital of Southwest Medical University, Luzhou, China; ^3^ Department of Oral and Maxillofacial Surgery, The Affiliated Hospital of Southwest Medical University, Luzhou, China; ^4^ Department of Hematology, The Affiliated Hospital of Southwest Medical University, Luzhou, China; ^5^ Stem Cell Laboratory, The Affiliated Hospital of Southwest Medical University, Luzhou, China; ^6^ Department of Pathology, The Affiliated Hospital of Southwest Medical University, Luzhou, China

**Keywords:** B-cell acute lymphoblastic leukemia, lncRNA expression profile, chemotherapy resistance, lncRNA DUXAP8/miR-29a/*PIK3CA* network, PI3K-AKT-mTOR signaling

## Abstract

**Purpose:**

This study aimed to determine the expression profiles of long non-coding RNA (lncRNA), microRNA (miRNA), and mRNA in chemotherapy-resistant B-cell acute lymphoblastic leukemia (B-ALL).

**Methods:**

LncRNA, miRNA, and mRNA profiles were assessed by RNA-seq in diagnostic bone marrow samples from 6 chemotherapy-resistant and 6 chemotherapy-sensitive B-ALL patients. The lncRNA DUXAP8/miR-29a/*PIK3CA* signaling network was identified as the most dysregulated in chemoresistant patient samples, and its effect on cellular phenotypes, PI3K-AKT-mTOR signaling, and chemosensitivity of doxorubicin (Dox)-resistant Nalm-6 (N6/ADR), and Dox-resistant 697 (697/ADR) cells were assessed. Furthermore, its synergy with inotuzumab ozogamicin treatment was investigated.

**Results:**

1,338 lncRNAs, 75 miRNAs, and 1620 mRNAs were found to be dysregulated in chemotherapy-resistant B-ALL in comparison to chemotherapy-sensitive B-ALL patient samples. Through bioinformatics analyses and RT-qPCR validation, the lncRNA DUXAP8/miR-29a/*PIK3CA* network and PI3K-AKT-mTOR signaling were identified as significantly associated with B-ALL chemotherapy resistance. In N6/ADR and 697/ADR cells, LncRNA DUXAP8 overexpression and *PIK3CA* overexpression induced proliferation and inhibited apoptosis, and their respective knockdowns inhibited proliferation, facilitated apoptosis, and restored Dox chemosensitivity. MiR-29a was shown to affect the lncRNA DUXAP8/*PIK3CA* network, and luciferase reporter gene assay showed direct binding between lncRNA DUXAP8 and miR-29a, as well as between miR-29a and *PIK3CA*. Targeting lncRNA DUXAP8/miR-29a/*PIK3CA* network synergized with inotuzumab ozogamicin’s effect on N6/ADR and 697/ADR cells.

**Conclusion:**

Targeting the lncRNA DUXAP8/miR-29a/*PIK3CA* network not only induced an apoptotic effect on Dox-resistant B-ALL and restored Dox chemosensitivity *via* PI3K-AKT-mTOR signaling but also showed synergism with inotuzumab ozogamicin treatment.

## Introduction

Acute lymphoblastic leukemia (ALL), as a hematological malignancy with the unrestricted proliferation of abnormal, immature lymphocytes or their progenitors that cause bone marrow element dysregulation, affects 1.7 new cases per 100,000 populations annually and accounts for nearly 2% of all lymphoid neoplasms in the United States (1, 2). ALL mainly occurs in children but less in adults, with the predominant type of B-cell ALL (B-ALL), which accounts for approximately 85% of total ALL cases (1, 2). Along with the improvement of treatment strategies, precision medicine, drug innovations, and so on, the prognosis of ALL is much improved in recent decades (3–6). However, long-term survival benefits are only achieved in the majority of childhood ALL, while only 30%–40% of adult ALL achieves long-term disease-free survival; especially, after relapse or refractoriness, the role of salvage chemotherapy is very limited, with less than 10% of long survival (7, 8). Therefore, the efforts to uncover underlying pathogenesis then discover novel treatment targets for ALL are never ceased.

Long non-coding RNA (lncRNA), a kind of non-coding RNA recognized recently with a length of more than 200 bp, has been reported to regulate a huge amount of biological processes and to be involved in the pathogenesis of most diseases *via* its interaction with cellular compounds such as miRNA, mRNA, DNA, and proteins (9–13). Besides, lncRNA has been also observed to be deeply involved in the development, progression, and treatment response of hematological malignancies (14–16). In the aspect of B-ALL, a recent comprehensive bioinformatics analysis identifies 1,235 dysregulated lncRNAs engaged in B-ALL subtype classification, and 942 aberrant lncRNAs related to B-ALL relapse (17). In addition, some specific lncRNAs were identified to regulate B-ALL growth and apoptosis, including lncRNA TEX41, lncRNA CRNDE, and lncRNA TCL6 (18–20). However, the function of a large majority of lncRNAs in B-ALL remains obscure, and in particular, seldom studies have investigated the comprehensive lncRNA profile involved in treatment refractoriness, not to mention the detailed molecule mechanism of some key lncRNAs underlying chemotherapy resistance.

Therefore, the current study aimed to investigate lncRNA, miRNA, and mRNA expression profiles related to B-ALL chemotherapy resistance *via* RNA sequencing, then further to explore the effect and interaction of the lncRNA DUXAP8/miR-29a/*PIK3CA* network on drug-resistant B-ALL cell proliferation, apoptosis, and chemosensitivity as well as its synergy with inotuzumab ozogamicin.

## Methods

### Samples

A total of 12 diagnostic bone marrow samples were collected from adult patients with B-ALL treated at our hospital. All bone marrows were sampled before the initiation of induction therapy. Of 12 bone marrow samples, 6 samples were from the adult B-ALL patients who achieved complete remission (CR) following the induction therapy with the VDP (vincristine, doxorubicin, prednisone) regimen, and these patients were considered as chemotherapy-sensitive patients (S-ALL) in the study; then, another 6 samples were from the adult B-ALL patients failing to achieve response at the end of the induction therapy with the VDP regimen, who exhibited a minimal residual disease (MRD) >1 × 10^-2^ by flow cytometry at the end of the induction therapy, and these patients were considered as chemotherapy-resistant patients (R-ALL) in the study.

The inclusion criteria of patients were as follows: (1) newly diagnosed as B-ALL by ESMO criteria (21); (2) older than 18 years; (3) received VDP regimen induction treatment; (4) had available induction treatment response data; and (5) had accessible bone marrow samples before treatment. The exclusion criteria of patients were as follows: (1) other types of ALL; (2) complicated with or history of other hematological malignancies or solid tumors; and (3) history of chemotherapy. This study was approved by the Ethics Committee of our hospital. The written informed consents were offered by patients.

### Workflow of RNA Sequencing Including lncRNA, mRNA, and MicroRNA

Total RNA in R-ALL and S-ALL bone marrow samples was isolated with TRIzol reagent (Invitrogen, Carlsbad, CA, USA) and quantified with Agilent 2100 (Agilent, Santa Clara, CA, USA). The ribosomal RNA was depleted using the Ribo-Zero rRNA Removal Kit (Illumina, San Diego, CA, USA), and the library of mRNA and lncRNA was constructed using the Illumina TruSeq Stranded Total RNA Library Prep Kit (Illumina, USA) according to the kit’s protocol. For the construction of the miRNA library, a miRNA library kit (Qiagen, Hilden, Germany) was applied. HiSeq 2500 (Illumina, USA) was applied to complete the sequencing of lncRNA, mRNA, and miRNA, respectively. After the acquisition of data, the R project (Version 3.6.3) was applied to complete quantile normalization, data processing, bioinformatics analysis, and graph plotting. Briefly, the Factoextra package and Pheatmap package were acquired to perform principal component analysis (PCA) and heatmap analysis, respectively; the DESeq2 and Limma package was adopted to analyze the mean gene expression, fold change (FC), and differentially expressed lncRNA, mRNA, or miRNA (DElncRNA, DEmRNA, or DEmiRNA). The DElncRNAs, DEmRNAs, and DEmiRNAs were shown with a volcano plot. Gene Ontology (GO) and Kyoko Encyclopedia of Genes and Genomes (KEGG) enrichment analyses were conducted *via* the DAVID web server. Pearson’s correlation coefficient was used to calculate the correlation between the expression of DElncRNAs and DEmRNAs. The potential target of DEmiRNAs was investigated by miRanda. The competing endogenous RNA (ceRNA) network was displayed using the graph in R packages, which contained the following ceRNAs: (1) top 10 deregulated (5 upregulated and 5 downregulated) DElncRNAs according to log_2_FC; (2) DEmRNAs, which have a significant correlation with the DElncRNAs mentioned in (1) (Pearson’s correlation coefficient >0.9 or <-0.9); and (3) DEmiRNAs, whose potential targets were DEmRNAs and DElncRNAs mentioned in (1) and (2). The expressions of the selected lncRNA, miRNA, and mRNA were evaluated by reverse transcription-quantitative polymerase chain reaction (RT-qPCR).

RNA-sequencing raw data in the article are available on the public database “National Omics Data Encyclopedia (NODE) database (https://www.biosino.org/node)” with tracking number OEP003016. The link is https://www.biosino.org/node/project/detail/OEP003016.

### Cell Culture

Human B-ALL cells Nalm-6 and 697 were obtained from DSMZ (Braunschweig, Germany). The doxorubicin (Dox)-resistant Nalm-6 (N6/ADR) cells were obtained from ATCC (Rockville, USA). The Dox-resistant 697 (697/ADR) cells were constructed according to a previous study (22). The Nalm-6 and 697 cells were cultured in RPMI-1640 medium (HyClone, Logan, UT, USA) supplemented with 10% fetal bovine serum (FBS) (HyClone, USA). The N6/ADR and 697/ADR cells were cultured in 70 nM Dox (Selleck, Houston, UT, USA) containing RPMI-1640 medium supplemented with 10% FBS. The Dox resistance of cells was confirmed by chemosensitivity to Dox assay. The expressions of lncRNA DUXAP8, miR-29a, *PIK3CA* in Nalm-6, N6/ADR, 697, and 697/ADR cells were evaluated.

### LncRNA DUXAP8 Plasmid Transfection

Control lncRNA overexpression plasmid, lncRNA DUXAP8 overexpression plasmid, control lncRNA short hairpin RNA (shRNA) plasmid, and lncRNA DUXAP8 shRNA plasmid were constructed by Shanghai GenePharma Co., Ltd (Shanghai, China), with pEX-2 vector (GenePharma, Shanghai, China) and pGPU6 vector (GenePharma, China), respectively. The plasmids were transfected into N6/ADR or 697/ADR cells in the presence of Lipofectamine™ 2000 (Invitrogen, USA). After transfection, the cells were divided into oeCTL, oeLNC, shCTL, and shLNC groups. The N6/ADR and 697/ADR cells that were not transfected served as blank control.

### miR-29a Plasmid Transfection

Control miR shRNA and miR-29a shRNA plasmids were constructed with the pGCMV/miR/inhibit vector (GenePharma) by Shanghai GenePharma Co., Ltd (Shanghai, China). After the construction, the control miR shRNA plasmid, miR-29a shRNA plasmid, control lncRNA shRNA plasmid, and lncRNA DUXAP8 shRNA plasmid were co-transfected into N6/ADR and 697/ADR cells by applying Lipofectamine™ 2000, accordingly. The cells were then divided into 5 groups: (a) blank group, no transfection cells; (b) shCTL group, cells were transfected with control lncRNA shRNA plasmid and control miR shRNA plasmid; (c) shLNC group, cells were transfected with lncRNA DUXAP8 shRNA plasmid and control miR shRNA plasmid; (d) shMIR group, cells were transfected with control lncRNA shRNA plasmid and miR-29a shRNA plasmid; and (e) shLNC + shMIR group, cells were transfected with lncRNA DUXAP8 shRNA plasmid and miR-29a shRNA plasmid.

### *PIK3CA* Plasmid Transfection

The pEX-2 vector and pGCMV/miR vector (GenePharma, China) were applied to construct the control mRNA overexpression, *PIK3CA* overexpression, control miR overexpression, and miR-29a overexpression plasmids by Shanghai GenePharma Co., Ltd. (Shanghai, China). To perform the co-transfection of control mRNA overexpression plasmid, *PIK3CA* overexpression plasmid, control miR overexpression plasmid, and miR-29a overexpression plasmid, Lipofectamine™ 2000 was adopted with the manufacturer’s instruction followed. The following groups were generated after transfection: (a) blank group, cells without transfection; (b) oeCTL group, cells were transfected with control mRNA overexpression plasmid and control miR overexpression plasmid; (c) oeMIR group, cells were transfected with miR-29a overexpression plasmid and control mRNA overexpression plasmid; (d) oePIK3CA group, cells were transfected with control miR overexpression plasmid and *PIK3CA* overexpression plasmid; and (e) oeMIR + oePIK3CA group, cells were transfected with miR-29a overexpression plasmid and *PIK3CA* overexpression plasmid.

### PI3K-AKT-mTOR Pathway Activation

The *PIK3CA* overexpression plasmid or mTOR activator (MHY1485) (2 μM, MCE, China) was cocultured with lncRNA DUXAP8 knockdown plasmid in N6/ADR and 697/ADR cells, followed by the detection of cell viability *via* CCK-8 assay, to explore the role of the PI3K-AKT-mTOR pathway in the restored chemosensitivity by lncRNA DUXAP8 knockdown.

### RT-qPCR

The TRIzol Reagent was adopted to extract total RNA. The concentration of total RNA was evaluated by NanoDrop 2000 (Thermo, USA). 1 μg total RNA was transcribed into cDNA using the RT Reagent Kit (Takara, Shiga, Japan) and subjected to the following thermal cycling: 37°C for 15 minutes (min) and 85°C for 5 seconds (sec). The qPCR mix (Takara, Japan) was used to complete qPCR. *β-Actin* was used as an internal reference for lncRNA and mRNA, and *U6* served as an internal reference for miRNA. Primers were listed as follows: lncRNA DUXAP8, forward, 5′ ACCAGCCTCACTAGCACTCTC 3′, reverse, 5′ CTTCCAGCCTCAGCCTCCTAA 3′; *PIK3CA*, forward, 5′ TTCTCAACTGCCAATGGACTGT 3′, reverse, 5′ AGCACGAGGAAGATCAGGAATG 3′; β-actin, forward 5′ GGCACCACACCTTCTACAATGA 3′, reverse, 5′ TCTCCTTAATGTCACGCACGATT 3′; miR-29a, forward, 5′ ACACTCCAGCTGGGTAGCACCATCTGAAAT 3′, reverse, 5′ TGTCGTGGAGTCGGCAATTC 3′; U6, forward, 5′ GCTCGCTTCGGCAGCACATA3′, reverse, 5′ AATATGGAACGCTTCACGAATTTGC 3′.

### Western Blot

The cells were lysed with RIPA buffer (Beyotime, Shanghai, China). The BCA protein assay kit (Beyotime, China) was used to determine the protein concentration. After thermal denaturation, 20 μg protein was separated with 4%–20% precast gel (Beyotime, China). The protein was then transferred to a nitrocellulose membrane (PALL, Port Washington, NY, USA). After being blocked, the membrane was incubated with diluted primary antibodies and secondary antibodies, successively. Finally, the protein bands were visualized with the ECL Kit (Beyotime, China). The antibodies’ information was as follows: PIK3CA monoclonal antibody (1: 2,000) (Invitrogen, USA); Bcl-2 monoclonal antibody (1: 500) (Invitrogen, USA); cleaved caspase 3 monoclonal antibody (1: 1,000) (CST, UK); AKT monoclonal antibody (1:1,000) (CST; UK); phospho-AKT (pAKT) monoclonal antibody (1:1,000) (CST, UK); mTOR monoclonal antibody (1:3,000) (Invitrogen, USA); phospho-mTOR (pmTOR) monoclonal antibody (1:1,000) (CST, UK); S6K monoclonal antibody (1:3,000) (CST, UK); phospho-S6K (pS6k) monoclonal antibody (1:1,000) (CST, UK); and anti-rabbit IgG, HRP-linked antibody (1: 10,000) (Invitrogen, USA).

### Cell Proliferation and Cell Apoptosis

The Cell Counting Kit-8 (Beyotime, China) was applied to assess cell proliferation. 3 × 10^3^ cells were loaded into a 96-well plate. 10 μl reagent was added and incubated with cells for 2 h after transfection. A microplate reader was used to read optical density (OD) values. The TUNEL Apoptosis Assay Kit (Beyotime, China) was used to assess the cell apoptosis rate according to the instructions, with 2.5 × 10^4^ cells seeded into a 24-well plate.

### Chemosensitivity to Dox Assay

For the confirmation of Dox resistance, un-transfected Nalm-6, N6/ADR, 697, and 697/ADR were seeded in a 96-well plate with the number of 1 × 10^4^ and incubated with a range concentration (0, 5, 10, 20, 40, and 80 nM) of Dox for 24 h referring to a previous paper (23). For chemosensitivity detection of transfected cells, the cells were cultured with a range concentration (0, 5, 20, 80 nM) of Dox for 24 h. After the incubation, the cell viability was detected with Cell Counting Kit-8 as described in *Cell Proliferation*.

### Luciferase Reporter Gene Assay

To detect the binding of lncRNA DUXAP8 and miR-29a, the wild type and mutant type of lncRNA DUXAP8 were cloned into the pGL6 vector (Beyotime, China). The wild-type and mutant-type lncRNA DUXAP8 plasmids, miR-29a mimic, and control mimic were co-transfected into 293T cells (ATCC, USA) by Lipofectamine™ 2000. After 48 h of incubation, the cells were lysed and the fluorescence value was detected using Luciferase Reporter Gene Assay Kit (Beyotime, China). To detect the binding of *PIK3CA* and miR-29a, the *PIK3CA* wild-type and mutant-type plasmids were constructed with a pGL6 vector. With the application of Lipofectamine™ 2000, the *PIK3CA* wild-type plasmid, *PIK3CA* mutant-type plasmid, miR-29a mimic, and control mimic were co-transfected into 293T cells. At 48 h, the fluorescence value was detected as mentioned above.

### LncRNA DUXAP8 Modification and Inotuzumab Ozogamicin (CMC-544) Treatment

The control lncRNA overexpression plasmid, lncRNA DUXAP8 overexpression plasmid, control lncRNA shRNA plasmid, and lncRNA DUXAP8 shRNA plasmid were transfected into N6/ADR and 697/ADR cells according to the methods described in the *LncRNA DUXAP8 Plasmid Transfection*. The N6/ADR and 697/ADR cells that were not transfected served as blank control. Then, CMC-544 (Pfizer Inc., New York, NY, USA) was added into cells with various concentrations of 0, 0.5, 1, 2, 4, and 8 ng/ml (24). After incubation for 48 h, cell viability was evaluated with Cell Counting Kit-8, and the half-maximal inhibitory concentration (IC_50_) was calculated using Probit regression analysis by SPSS 23.0 (IBM, Armonk, NY, USA). At last, the transfected and blank control cells were incubated with CMC-544 at an amount of 2.670 or 2.794 ng/ml for 48 h, with the apoptosis rate determined by TUNEL Apoptosis Assay Kit. The cell viability and apoptosis detection were carried out as described in *Cell Proliferation and Cell Apoptosis*.

### Statistical Analysis

All data in this study were expressed as mean ± standard deviation. The difference among groups and between two groups was assessed by one-way ANOVA followed by Dunnett’s test or t-test. GraphPad Prism 7.02 (GraphPad Software Inc., La Jolla, CA, USA) was applied to analyze data and make graphs. The statistical significance was defined as *p* < 0.05. No significance: NS (p>0.05); *p<0.05, **p<0.01, ***p<0.001.

## Results

### Study Flow

The current study consisted of two main parts: the clinical part and the experimental part (**Supplementary Figure 1**). In the clinical part, 6 R-ALL patients and S-ALL patients were enrolled to detect the lncRNA, miRNA, and mRNA profiles *via* RNA-seq and RT-qPCR validation to sort key networks related to chemoresistance. Then, experiments were performed *via* modifying lncRNA DUXAP8, miR-29a, and *PIK3CA* to explore their effect (alone or in combination) on Dox-resistant B-ALL cell proliferation, apoptosis, Dox chemosensitivity, etc.

### Characteristics

The enrolled B-ALL patients were aged 40 ± 9 years, with 50% males and 50% females. Meanwhile, the mean WBC was 59 ± 45 × 10^9^/l, and the mean bone marrow blast percentage was (83 ± 10)%. The detailed information of their characteristics is shown in **Table 1**. It was notable that by comparison, S-ALL patients showed a trend of lower WBC (*p* = 0.064) and CD19 percentage (*p* = 0.065) compared to R-ALL patients but lacked statistical significance. Furthermore, other characteristics were of no difference between them (**Table 1**).

**Table 1:** Clinical characteristics of B-ALL patients.

Items	Total patients (N = 12)	S-ALL patients (n = 6)	R-ALL patients (n = 6)	*p* value
Age (years), mean ± SD	40 ± 9	38 ± 9	42 ± 9	0.556
Male, no. (%)	6 (50)	4 (67)	2 (33)	0.248
Immunophenotype, no. (%)				1.000
T-ALL	0 (0)	0 (0)	0 (0)	
B-ALL	6 (100)	6 (100)	12 (100)	
Philadelphia chromosome (%)				1.000
Positive	0 (0)	0 (0)	0 (0)	
Negative	6 (100)	6 (100)	12 (100)	
CNSL, no. (%)	1 (8)	0 (0)	1 (16.7)	0.296
Laboratory index				
WBC (×10^9^/L), mean ± SD	59 ± 45	35 ± 21	83 ± 51	0.064
Hemoglobin (g/L), mean ± SD	84 ± 25	91 ± 27	77 ± 23	0.348
Platelet (×10^9^/L), mean ± SD	45 ± 27	49 ± 31	42 ± 25	0.701
Bone marrow blasts (%), mean ± SD	83 ± 10	79 ± 9	86 ± 11	0.246
Cytofluorimetric information				
CD19 (%), median (IQR)	82 (72–90)	76 (65–84)	88 (80–94)	0.065
CD79a (%), median (IQR)	86 (74–93)	84 (73–93)	87 (72–94)	0.937
CD22 (%), median (IQR)	79 (71–92)	77 (69–90)	84 (75–93)	0.485
CD20 (%), median (IQR)	44 (18–62)	44 (17–67)	45 (13–61)	0.818
Molecule genetics (%)				
BCR/ABL, no. (%)	0 (0)	0 (0)	0 (0)	1.000
MLL rearrangement, no. (%)	1 (8)	0 (0)	1 (8)	0.296
TEL-AML1, no. (%)	1 (8)	1 (8)	0 (0)	0.296
E2A-PBX1, no. (%)	1 (8)	0 (0)	1 (8)	0.296
IL3-IGH, no. (%)	0 (0)	0 (0)	0 (0)	1.000

ALL, acute lymphoblastic leukemia; SD, standard deviation; CNSL, central nervous system leukemia; WBC, white blood cell; IQR, interquartile range.

### LncRNA, miRNA, and mRNA Expression Profiles

**Figure 1:**
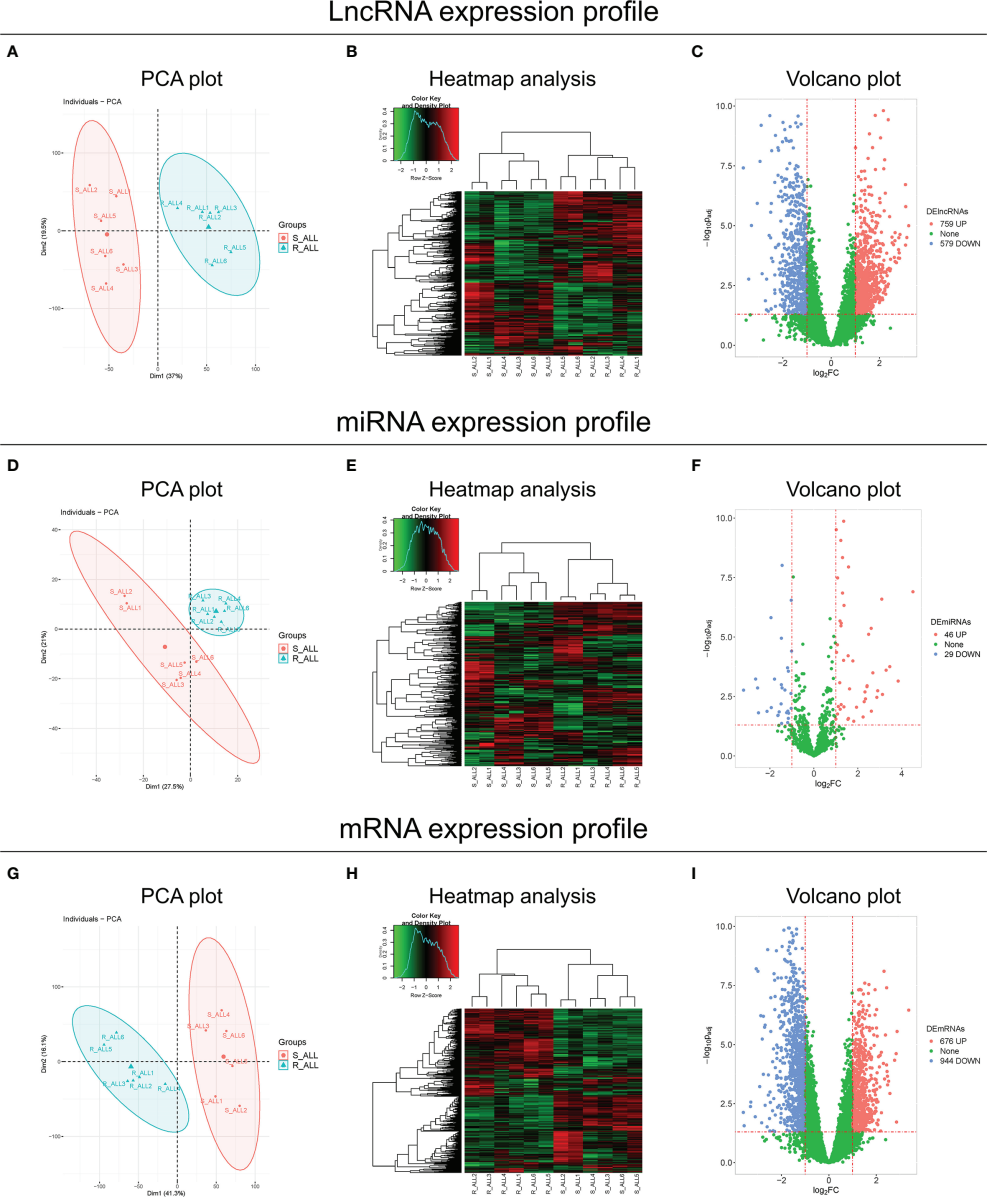
PCA, heatmap, and volcano analyses of RNA sequencing. PCA plot and heatmap analyses of the lncRNA expression profile, and volcano analysis of dysregulated lncRNAs between R-ALL and S-ALL patients **(A–C)**. PCA plot and heatmap analyses of the miRNA expression profile, and volcano analysis of dysregulated miRNAs between R-ALL and S-ALL patients **(D–F)**. PCA plot and heatmap analyses of the mRNA expression profile, and volcano analysis of dysregulated mRNAs between R-ALL and S-ALL patients **(G–I)**. The data were from the comparison of diagnostic B-ALL samples.

The lncRNA expression profile could clearly distinguish R-ALL patients from S-ALL patients *via* PCA plot analysis (**Figure 1A**) and showed a good inter-consistent trend in R-ALL patients and S-ALL patients, respectively, by heatmap analysis (**Figure 1B**). Then, a total of 759 upregulated and 579 downregulated lncRNAs were identified in R-ALL patients compared with S-ALL patients (**Figure 1C**).

Besides, the miRNA expression profile also distinguished R-ALL patients from S-ALL patients *via* PCA plot analysis (**Figure 1D**) and exhibited an acceptable inter-consistent trend by heatmap analysis (**Figure 1E**), with 46 upregulated/29 downregulated miRNAs discovered in R-ALL patients compared to S-ALL patients (**Figure 1F**). Furthermore, the mRNA expression profile was also able to differentiate R-ALL patients from S-ALL patients (**Figure 1G**), and disclosed an inter-consistent trend by heatmap analysis (**Figure 1H**), with 676 upregulated/944 downregulated mRNAs uncovered in R-ALL patients compared to S-ALL patients (**Figure 1I**). In addition, the detailed information of each dysregulated lncRNA, miRNA, and mRNA is exhibited in **Supplementary Tables 1–3**, respectively.

### Identification of lncRNA DUXAP8/miR-29a/*PIK3CA* Network and Downstream PI3K-AKT Pathway

**Figure 2:**
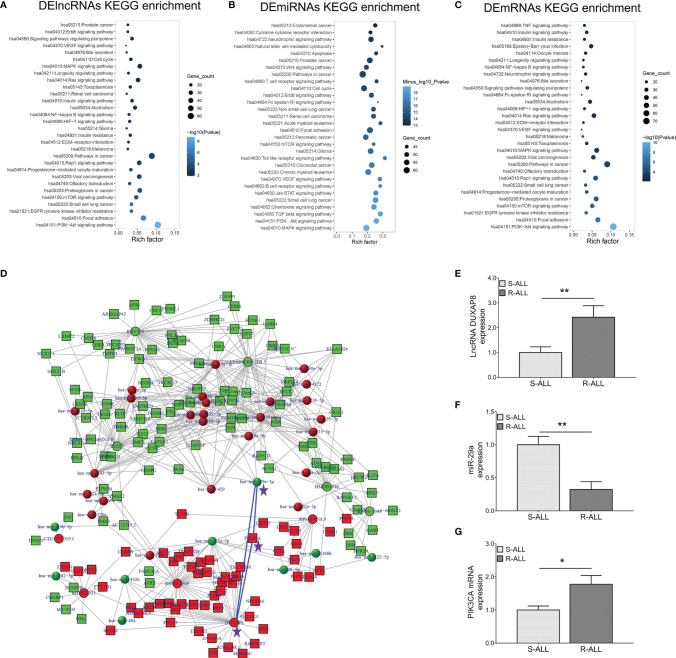
lncRNA–miRNA–mRNA co-expression network and RT-qPCR validation. KEGG **(A)** enrichment analysis according to DElncRNAs; KEGG **(B)** enrichment analysis according to DEmiRNAs; KEGG **(C)** enrichment analysis according to DEmRNAs. LncRNA–miRNA–mRNA co-expression network based on dysregulated genes between R-ALL and S-ALL patients **(D)**. RT-qPCR detection data of lncRNA DUXAP8 **(E)**, miR-29a **(F)**, and *PIK3CA* **(G)** expressions between R-ALL and S-ALL patients.

KEGG enrichment analyses of dysregulated lncRNA (**Figure 2A**), miRNA (**Figure 2B**), and mRNA (**Figure 2C**) expression profiles were carried out, which observed that they were all closely related to the PI3K-AKT pathway. Inspiringly, a comprehensive ceRNA network was established based on dysregulated lncRNA, miRNA, and mRNA expression profiles, which discovered that the lncRNA DUXAP8/miR-29a/*PIK3CA* network was a key component (**Figure 2D**). What is more, *PIK3CA* is the key component involved in the PI3K-AKT pathway, which is closely engaged in ALL pathogenesis and treatment sensitivity (25–27). Subsequently, RT-qPCR was performed to verify their expressions, which also demonstrated that lncRNA DUXAP8 was upregulated (**Figure 2E**), miR-29a was downregulated (**Figure 2F**), and *PIK3CA* was overexpressed (**Figure 2G**) in R-ALL patients compared to S-ALL patients (all *p* < 0.05). Based on the abovementioned information, the lncRNA DUXAP8/miR-29a/*PIK3CA* network and downstream PI3K-AKT pathway were essential in chemotherapy resistance, then subsequent experiments were performed to explore and validate this issue.

### Effect of lncRNA DUXAP8 Modification on Dox-Resistant B-ALL Cells

Firstly, Dox resistance was confirmed in N6/ADR and 697/ADR cells (**Supplementary Figures 2A, B**), then upregulated lncRNA DUXAP8 and *PIK3CA* and downregulated miR-29a were observed in N6/ADR and 697/ADR cells (all *p* < 0.05, **Supplementary Figures 2C, D**).

**Figure 3:**
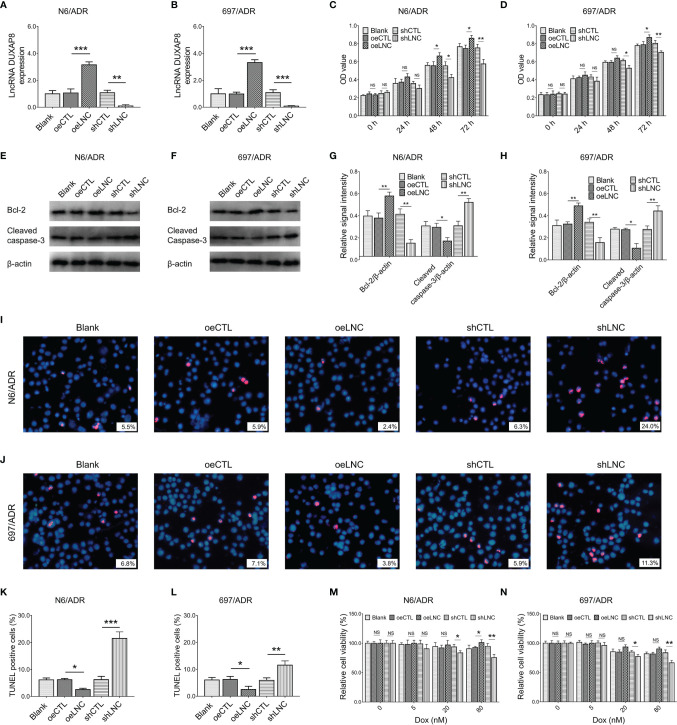
Cell behaviors after lncRNA DUXAP8 modification. LncRNA DUXAP8 **(A, B)**, cell proliferation by CCK-8 **(C, D)**, apoptotic marker expressions **(E–H)**, cell apoptosis rate **(I–L)**, and chemosensitivity to Dox **(M, N)** among blank, oeCTL, oeLNC, shCTL, and shLNC groups in N6/ADR cells and 697/ADR cells.

After confirmation of transfection efficiency (all *p* < 0.01, **Figures 3A, B**), lncRNA DUXAP8 overexpression was observed to promote cell proliferation in N6/ADR (**Figure 3C**) and 697/ADR (**Figure 3D**) cells (both *p* < 0.05). Then, lncRNA DUXAP8 overexpression increased Bcl-2 expression but repressed cleaved caspase-3 expression in N6/ADR (**Figures 3E, G** **)** and 697/ADR (**Figures 3F, H** **)** cells (all *p* < 0.05). In addition, lncRNA DUXAP8 overexpression reduced the cell apoptosis rate reflected by TUNEL-positive cell percentage in N6/ADR (**Figures 3I, K** **)** and 697/ADR (**Figures 3J, L** **)** cells (both *p* < 0.05). Besides, lncRNA DUXAP8 overexpression decreased Dox chemosensitivity in N6/ADR cells to some extent (**Figure 3M**) (*p* < 0.05) but did not affect Dox chemosensitivity in 697/ADR cells (**Figure 3N**) (*p* > 0.05). Inversely, lncRNA DUXAP8 knockdown exhibited the opposite effect to its overexpression in regulating the abovementioned cell functions; in particular, lncRNA DUXAP8 knockdown restored Dox chemosensitivity to some extent in N6/ADR (**Figure 3M**) and 697/ADR (**Figure 3N**) cells (all *p* < 0.05).

### Interaction Between lncRNA DUXAP8 and miR-29a

**Figure 4:**
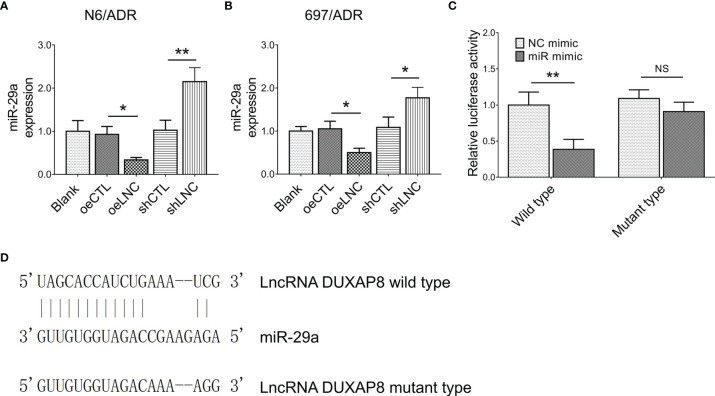
Interaction between lncRNA DUXAP8 and miR-29a. MiR-29a expression among blank, oeCTL, oeLNC, shCTL, and shLNC groups in N6/ADR cells and 697/ADR cells **(A, B)**. Luciferase reporter gene assay results **(C)** and binding site between lncRNA DUXAP8 and miR-29a **(D)**.

It was then observed that lncRNA DUXAP8 negatively regulated miR-29a expression in both N6/ADR (**Figure 4A**) and 697/ADR (**Figure 4B**) cells (all *p* < 0.05). By the Luciferase reporter gene assay with the designed binding sequence shown in **Figure 4C**, lncRNA DUXAP8 was found to be directly bound to miR-29a to regulate its expression (*p* < 0.01, **Figure 4D**).

### Effect of Modification of lncRNA DUXAP8 and miR-29a on Dox-Resistant B-ALL Cells

**Figure 5:**
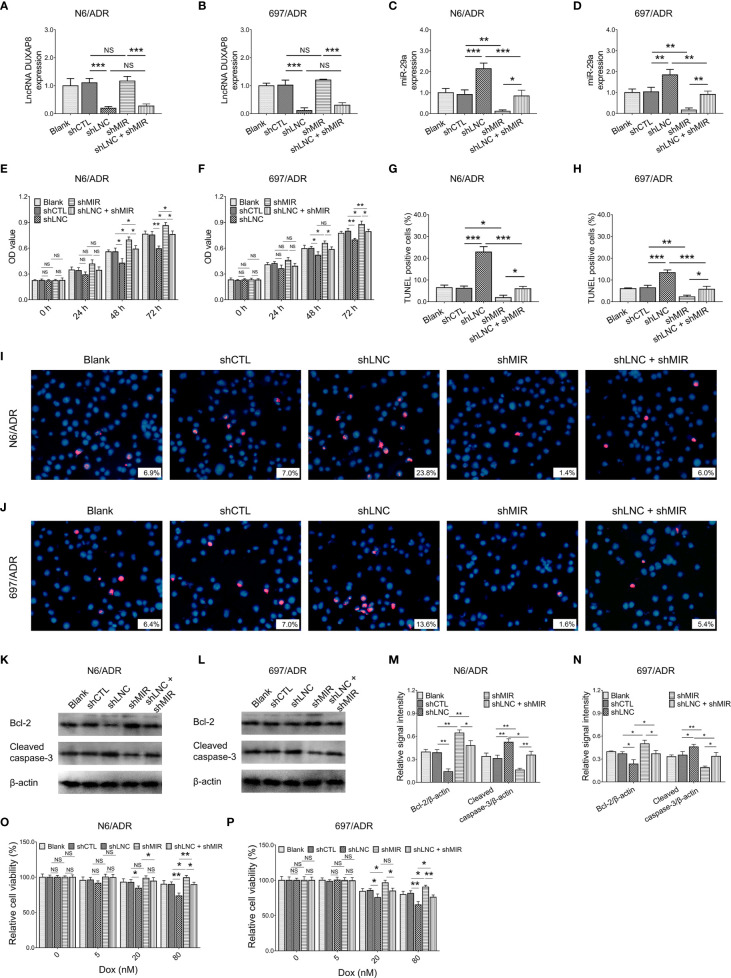
Cell behaviors after lncRNA DUXAP8 and miR-29a modification. LncRNA DUXAP8 **(A, B)**, miR-29a **(C, D)**, cell proliferation by CCK-8 **(E, F)**, cell apoptosis rate **(G–J)**, apoptotic marker expressions **(K–N)**, and chemosensitivity to Dox **(O, P)** among blank, shCTL, shLNC, shMIR, and shLNC +shMIR groups in N6/ADR cells and 697/ADR cells.

MiR-29a knockdown did not affect lncRNA DUXAP8 expression in both N6/ADR (**Figure 5A**) and 697/ADR (**Figure 5B**) cells (both *p* > 0.05), while lncRNA DUXAP8 knockdown increased miR-29a expression in both N6/ADR (**Figure 5C**) and 697/ADR (**Figure 5D**) cells (both *p* < 0.01). Subsequent experiments revealed that miR-29a knockdown enhanced cell proliferation in N6/ADR (**Figure 5E**) and 697/ADR (**Figure 5F**) cells (both *p* < 0.05); it also suppressed the cell apoptosis rate reflected by the TUNEL-positive cell percentage in N6/ADR (**Figures 5G, I** **)** and 697/ADR (**Figures 5H, J** **)** cells (both *p* < 0.05); then, it promoted Bcl-2 expression but reduced cleaved caspase-3 expression in N6/ADR (**Figures 5K, M** **)** and 697/ADR (**Figures 5L, N** **)** cells (all *p* < 0.05). Besides, miR-29a knockdown decreased Dox chemosensitivity in N6/ADR (**Figure 5O**) and 697/ADR (**Figure 5P**) cells (both *p* < 0.05).

Most notably, miR-29a knockdown compensated for the effects of lncRNA DUXAP8 knockdown on cell proliferation (**Figure 5E**), cell apoptosis rate reflected by TUNEL-positive cell percentage (**Figures 5G, I** **)**, apoptotic markers (**Figures 5K, M** **)**, and Dox chemosensitivity (**Figure 5O**) in N6/ADR cells (all *p* < 0.05). Similarly, miR-29a knockdown also compensated for the effects of lncRNA DUXAP8 knockdown on cell proliferation (**Figure 5F**), cell apoptosis rate reflected by TUNEL-positive cell percentage (**Figures 5H, J** **)**, apoptotic markers (**Figures 5L, N** **)**, and Dox chemosensitivity (**Figure 5P**) in 697/ADR cells (all *p* < 0.05).

### Interaction Among lncRNA DUXAP8, miR-29a, and *PIK3CA*

**Figure 6:**
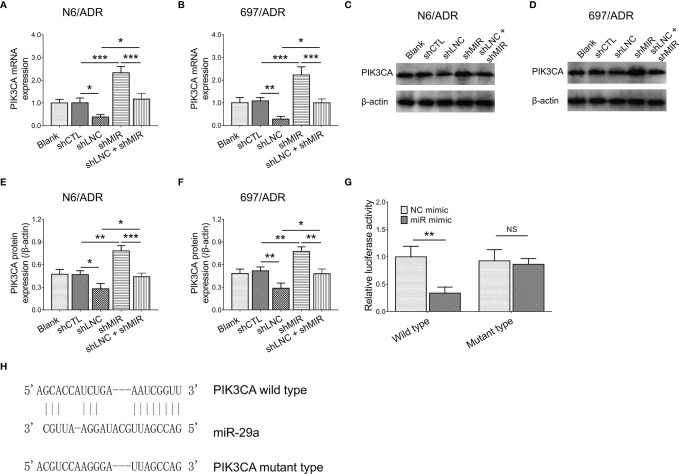
Interaction among lncRNA DUXAP8, miR-29a, and *PIK3CA*. *PIK3CA* expression among blank, shCTL, shLNC, shMIR, and shLNC +shMIR groups in N6/ADR cells and 697/ADR cells **(A–F)**. Luciferase reporter gene assay results **(G)** and binding site between miR-29a and *PIK3CA* **(H)**.

LncRNA DUXAP8 knockdown decreased the *PIK3CA* gene expression in N6/ADR (**Figure 6A**) and 697/ADR (**Figure 6B**) cells (both *p* < 0.05); it also repressed PIK3CA protein expression in N6/ADR (**Figures 6C, E** **)** and 697/ADR (**Figures 6D, F** **)** cells (both *p* < 0.05), whereas miR-29a knockdown increased *PIK3CA* gene expression in N6/ADR (**Figure 6A**) and 697/ADR (**Figure 6B**) cells (both *p* < 0.001), as well as elevated PIK3CA protein expression in N6/ADR (**Figures 6C, E** **)** and 697/ADR (**Figures 6D, F** **)** cells (both *p* < 0.01). Subsequent Luciferase reporter gene assay observed that miR-29a was directly bound to *PIK3CA* to regulate its expression (*p* < 0.01, **Figure 6G**), with the designed binding sequence shown in **Figure 6H**.

### Effect of Modification of miR-29a and *PIK3CA* on PI3K-AKT-mTOR Pathway

**Figure 7:**
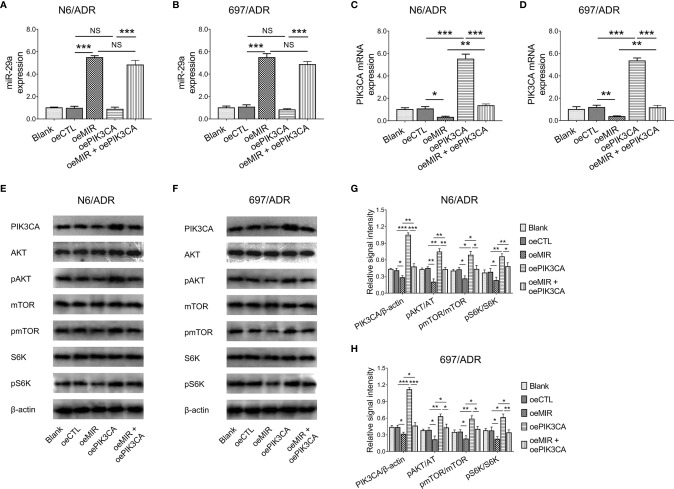
Interaction among miR-29a, *PIK3CA*, and PI3K-AKT-mTOR signaling. MiR-29a **(A, B)** and *PIK3CA* **(C, D)** expressions among blank, oeCTL, oeMIR, oePIK3CA, and oeMIR + oePIK3CA groups in N6/ADR cells and 697/ADR cells. PI3K-AKT-mTOR signaling among blank, oeCTL, oeMIR, oePIK3CA, and oeMIR + oePIK3CA groups in N6/ADR cells and 697/ADR cells **(E–H)**.

*PIK3CA* overexpression did not affect miR-29a expression in N6/ADR (**Figure 7A**) and 697/ADR (**Figure 7B**) cells (both *p* > 0.05), while miR-29a overexpression inhibited *PIK3CA* expression in N6/ADR (**Figure 7C**) and 697/ADR (**Figure 7D**) cells (both *p* < 0.05). It was then noted that miR-29a overexpression inactivated the PI3K-AKT-mTOR pathway in N6/ADR (**Figures 7E, G** **)** and 697/ADR (**Figures 7F, H** **)** cells (all *p* < 0.05), while *PIK3CA* overexpression activated the PI3K-AKT-mTOR pathway in N6/ADR (**Figures 7E, G** **)** and 697/ADR (**Figures 7F, H** **)** cells (all *p* < 0.05).

### Effect of Modification of miR-29a and *PIK3CA* on Dox-Resistant B-ALL Cells

**Figure 8:**
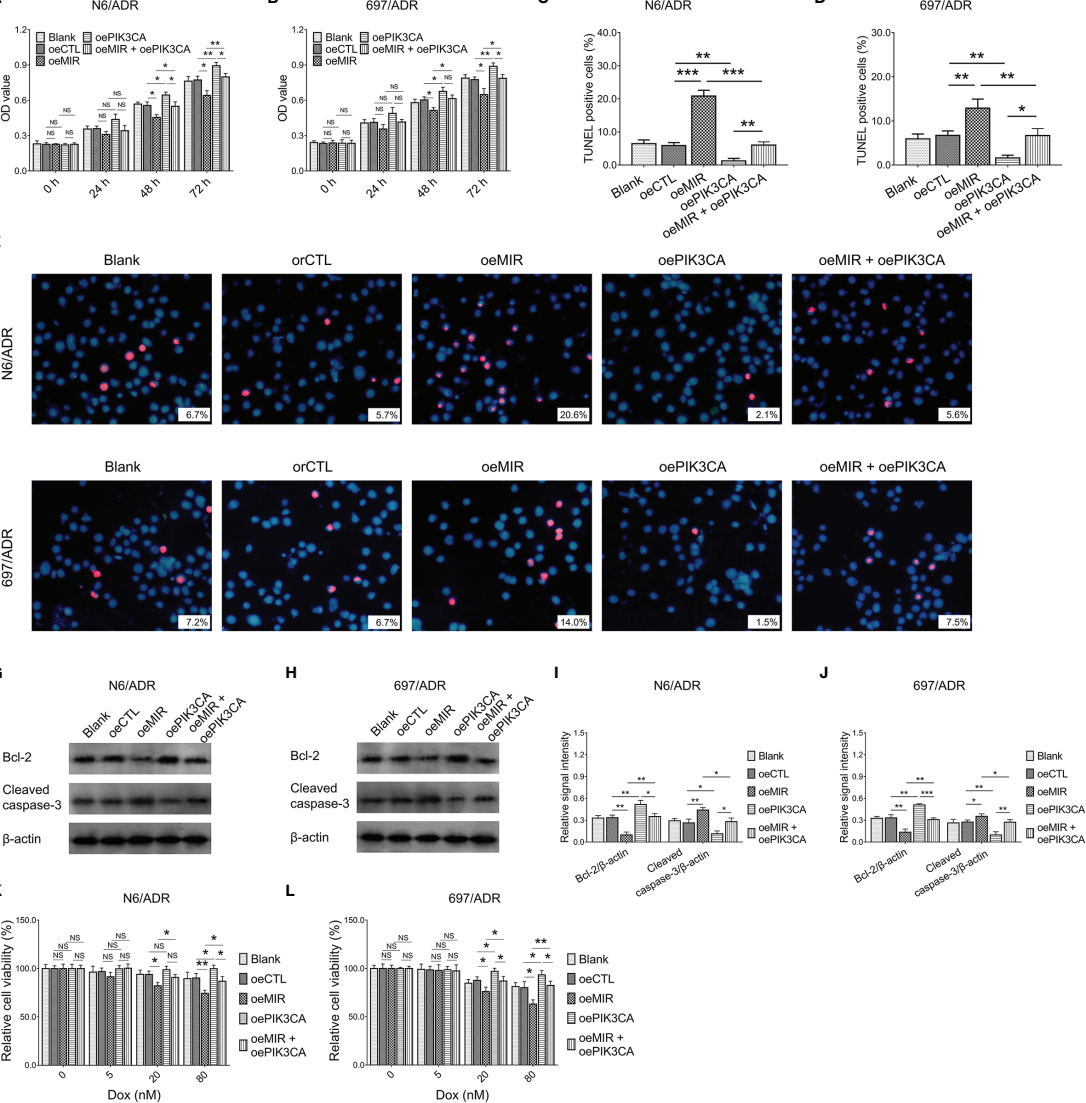
Cell behaviors after miR-29a and *PIK3CA* modification. Cell proliferation by CCK-8 **(A, B)**, cell apoptosis rate **(C–F)**, apoptotic marker expressions **(G–J)**, and chemosensitivity to Dox **(K, L)** among blank, oeCTL, oeMIR, oePIK3CA, and oeMIR + oePIK3CA groups in N6/ADR cells and 697/ADR cells.

MiR-29a overexpression decreased cell proliferation in N6/ADR (**Figure 8A**) and 697/ADR (**Figure 8B**) cells (both *p* < 0.05), while it increased the cell apoptosis rate reflected by the TUNEL-positive cell percentage in N6/ADR (**Figures 8C, E** **)** and 697/ADR (**Figures 8D, F** **)** cells (both *p* < 0.01). MiR-29a overexpression also inhibited Bcl-2 expression but promoted cleaved caspase-3 expression in N6/ADR (**Figures 8G, I** **)** and 697/ADR (**Figures 8H, J** **)** cells (all *p* < 0.05). Besides, miR-29a overexpression increased Dox chemosensitivity in N6/ADR (**Figure 8K**) and 697/ADR (**Figure 8L**) cells (both *p* < 0.05). However, *PIK3CA* overexpression showed the opposite effect as that of miR-29a overexpression in N6/ADR and 697/ADR cells (all *p* < 0.05).

It was inspiring that *PIK3CA* overexpression compensated for the effects of miR-29a overexpression on cell proliferation (**Figure 8A**), cell apoptosis rate reflected by the TUNEL-positive cell percentage (**Figures 8C, E** **)**, apoptotic markers (**Figures 8G, I** **)**, and Dox chemosensitivity (**Figure 8K**) in N6/ADR cells (all *p* < 0.05). Similarly, *PIK3CA* overexpression also compensated for the effects of miR-29a overexpression on cell proliferation (**Figure 8B**), cell apoptosis rate reflected by the TUNEL-positive cell percentage (**Figures 8D, F** **)**, apoptotic markers (**Figures 8H, J** **)**, and Dox chemosensitivity (**Figure 8L**) in 697/ADR cells (all *p* < 0.05).

In addition, *PIK3CA* overexpression plasmid or mTOR activator (MHY1485) was cocultured with lncRNA DUXAP8 knockdown plasmid to explore the role of the PI3K-AKT-mTOR pathway in the restored chemosensitivity by lncRNA DUXAP8 knockdown. Then, it was observed that PI3K-AKT-mTOR pathway activation decreased Dox chemosensitivity in N6/ADR (**Supplementary Figure 3A**) and 697/ADR cells (**Supplementary Figure 3B**) and also attenuated the effect of lncRNA DUXAP8 knockdown.

### LncRNA DUXAP8 Modification and Inotuzumab Ozogamicin on Dox-Resistant B-ALL Cells

**Figure 9:**
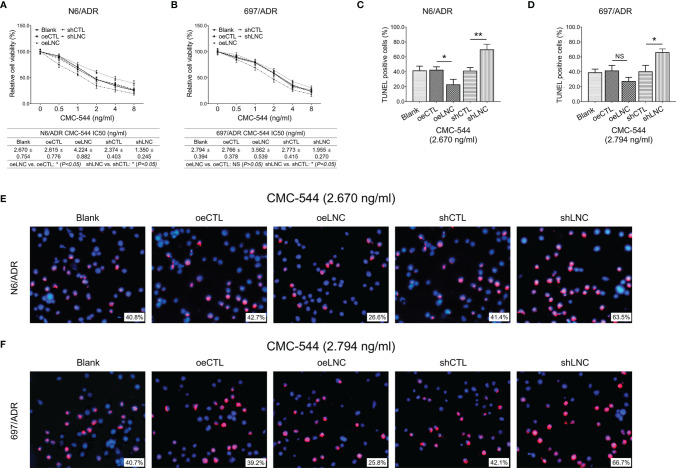
Cell behaviors after lncRNA DUXAP8 modification and inotuzumab ozogamicin treatment. Relative cell viability **(A, B)** and cell apoptosis rate **(C–F)** among blank, oeCTL, oeLNC, shCTL, and shLNC groups in inotuzumab ozogamicin treated N6/ADR cells and 697/ADR cells.

Inotuzumab ozogamicin is a novel drug recommended for the treatment of refractory B-ALL, so we further explored whether modification of lncRNA DUXAP8 affected inotuzumab ozogamicin treatment in Dox-resistant B-ALL cells. It was observed that lncRNA DUXAP8 overexpression increased the IC_50_ of inotuzumab ozogamicin in N6/ADR cells (**Figure 9A**) (*p* < 0.05), but not in 697/ADR cells (**Figure 9B**) (*p* > 0.05); it also decreased the cell apoptosis rate reflected by the TUNEL-positive cell percentage in inotuzumab ozogamicin-treated N6/ADR cells (**Figures 9C, E** **)** (*p* < 0.05), but not in 697/ADR cells (**Figures 9D, F** **)** (*p* > 0.05). Besides, lncRNA DUXAP8 knockdown decreased the IC_50_ of inotuzumab ozogamicin in N6/ADR cells (**Figure 9A**) and 697/ADR cells (**Figure 9B**) (both *p* < 0.05), which also enhanced the cell apoptosis rate reflected by the TUNEL-positive cell percentage in inotuzumab ozogamicin-treated N6/ADR cells (**Figures 9C, E** **)** and 697/ADR cells (**Figures 9D, F** **)** (both *p* < 0.05).

## Discussion

A previous secondary bioinformatics analysis involving The Cancer Genome Atlas (TCGA) data identifies 469 upregulated and 286 downregulated lncRNAs in ALL patients compared to controls (28). Another study analyzes the GSE67684 dataset from the Gene Expression Omnibus then validates the candidate lncRNAs by RT-qPCR, which observes 21 dysregulated lncRNAs in accordance with ALL patients compared to controls (29). However, no study has explored the aberrant pattern of the lncRNA profile between chemotherapy-resistant B-ALL patients and chemotherapy-sensitive patients. Our current study filled this gap, which observed that 1,338 lncRNAs, 75 miRNAs, and 1,620 mRNAs were dysregulated between chemotherapy-resistant B-ALL patients and chemotherapy-sensitive B-ALL patients. These data would propose new information on the interaction between genetics and chemotherapy resistance in B-ALL.

Since the concept of lncRNA is just initiated in a short time, in-depth studies of its mechanisms underlying ALL are very limited, and only a few studies have reported several specific lncRNAs that are involved in ALL pathogenesis, such as lncRNA MALAT1, lncRNA VPS9D1-AS1, and lncRNA TEX41 (18, 30, 31). However, few studies on lncRNA have been disclosed in the context of B-ALL chemotherapy resistance. LncRNA DUXAP8 is previously reported as an oncogene in several cancers. For instance, lncRNA DUXAP8 promotes neuroblastoma progression by regulating miR-29 mediated *NOL4L* and downstream Wnt/β-actin pathway (32); it also enhances colorectal cancer proliferation, migration, and invasion by regulating *EZH2* and *LSD1* (33). In terms of leukemia, only a related paper is available, which observes that lncRNA DUXAP8 regulates acute myeloid leukemia glycolysis and apoptosis through the Wnt/β-actin pathway (34). Furthermore, lncRNA DUXAP8 is also revealed as a regulator of an anticancer agent, such as a *PARP* inhibitor (35). However, no data in the aspect of lncRNA DUXAP8 in ALL are reported. In our present study, further bioinformatic analyses and RT-qPCR validation observed that lncRNA DUXAP8 was closely related to chemotherapy resistance in B-ALL patients; subsequent experiments also revealed that lncRNA DUXAP8 promotes cell proliferation and inhibits cell apoptosis, then targeting it restored the Dox chemosensitivity in chemoresistant B-ALL cell lines. The possible explanations were as follows: (1) lncRNA DUXAP8 regulated the miR-29a/*PIK3CA* network and downstream PI3K-AKT pathway, as shown by subsequent molecule mechanism experiments, to modify cell proliferation, apoptosis, and Dox chemosensitivity in B-ALL, and (2) lncRNA DUXAP8 activated several key carcinogenetic pathways such as Wnt/β-actin, AKT/mTOR, and WTAP/Fak, to regulate these cell functions (34, 36, 37).

MiR-29a is a well-known anti-oncogene in both solid tumor and hematological malignancies (38, 39). In leukemias, MiR-29a deficiency activates CD40 signaling/T-cell interaction to engage in chronic lymphocytic leukemia pathogenesis (40); meanwhile, it is greatly dysregulated in acute myeloid leukemia, chronic lymphocytic leukemia, and ALL patients (41–43). In the aspect of PI3K-AKT, it is a documented pathway involved in ALL development, progression, and even treatment sensitivity (25, 44, 45). In our present study, we observed that the lncRNA DUXAP8/miR-29a/*PIK3CA* network and downstream PI3K-AKT pathway were closely related to chemotherapy resistance in B-ALL patients. Then, comprehensive experiments were performed to demonstrate that the lncRNA DUXAP8/miR-29a/*PIK3CA* network regulates cell proliferation and apoptosis and targeting the network could restore the Dox chemosensitivity in chemoresistant B-ALL cell lines. The most possible explanation was further excavated, which observed that the effect of the lncRNA DUXAP8/miR-29a/*PIK3CA* network in chemoresistant B-ALL resulted from activation of downstream PI3K-AKT-mTOR signaling. This finding provided a novel target network for chemoresistant B-ALL, which was exciting.

For chemotherapy-refractory or relapsed B-ALL patients, salvage treatment options are lacking and urgently need investigation. Currently, inotuzumab ozogamicin, an anti-CD22 antibody–drug conjugate, achieves encouraging efficacy and acceptable tolerance as a salvage treatment regimen in refractory or relapsed B-ALL patients (46–50). In the current study, it was further discovered that targeting lncRNA DUXAP8 and inotuzumab ozogamicin had synergistic effects in killing chemoresistant B-ALL cells. These would provide a new train of thought regarding refractory B-ALL treatment.

Despite the interesting findings initiated in our present study, some limitations existed as well: firstly, the validation of expression of the lncRNA DUXAP8/miR-29a/*PIK3CA* network could be further conducted in a larger sample size of B-ALL patients. Secondly, targeting lncRNA DUXAP8 restored the Dox chemosensitivity in Dox-resistant B-ALL cell lines *via* miR-29a/*PIK3CA* and subsequent PI3K-AKT-mTOR signaling, while whether other potential mechanisms beyond this could be further investigated. Thirdly, overall changes induced by modification in lncRNA DUXAP8 were relatively minor, then the synergistic effect of modifying lnc DUXAP8 on inotuzumab ozogamicin was relatively minor and observed only in one tested cell line, so further validation experiments were needed in the future.

In conclusion, targeting the lncRNA DUXAP8/miR-29a/*PIK3CA* network not only exhibits a good killing effect on Dox-resistant B-ALL and restores Dox chemosensitivity *via* PI3K-AKT-mTOR signaling but also synergizes with inotuzumab ozogamicin. These imply the effectiveness of this network as a treatment target for chemotherapy-refractory B-ALL.

## Data Availability Statement

The original contributions presented in the study are publicly available. These data can be accessed using the following link/accession number: https://www.biosino.org/node/search; OEP003016.

## Ethics Statement

The studies involving human participants were reviewed and approved by the Ethics Committee of The Affiliated Hospital of Southwest Medical University. The patients/participants provided their written informed consent to participate in this study.

## Author Contributions

LZ and JT conceived and designed the study. LZ, SZ, XL, and JT collected and analyzed the data. LZ, TZ, and JT prepared the figures. LZ, SZ, and XL wrote the manuscript. TZ and JT edited the manuscript. All authors contributed to the article and approved the submitted version.

## Funding

This study was supported by Sichuan Science and Technology Program (No. 2019YFS0301), Doctor Research Foundation of The Affiliated Hospital of Southwest Medical University (No. 19032; No. 19079), The Key Research Project from Health and Family Planning Commission of Sichuan Province (No. 18ZD014), and the Applied Basic Research Project of Luzhou Science and Technology Bureau (2019LZXNYDJ54;2020LZXNYDJ48 and 2020-JYJ-50).

## Conflict of Interest

The authors declare that the research was conducted in the absence of any commercial or financial relationships that could be construed as a potential conflict of interest.

## Publisher’s Note

All claims expressed in this article are solely those of the authors and do not necessarily represent those of their affiliated organizations, or those of the publisher, the editors and the reviewers. Any product that may be evaluated in this article, or claim that may be made by its manufacturer, is not guaranteed or endorsed by the publisher.

## Supplementary Material

Supplementary Figure 1 Study flow chart.

Supplementary Figure 2 Expression of lncRNA DUXAP8, miR-29a and *PIK3CA* in N6/ADR and 697/ADR cells. Confirmation of the Dox resistance status of N6/ADR **(A)** and 697/ADR cells **(B)**. Expression of lncRNA DUXAP8, miR-29a and *PIK3CA* in N6/ADR and 697/ADR cells compared to normal NALM6 and 697 cells, respectively **(C, D).**

Supplementary Figure 3 Role of PI3K-AKT-mTOR pathway in this restored chemosensitivity. Relative cell viability among Blank, CTL, shLNC, oePIK3CA, shLNC+oePIK3CA, MHY1485, shLNC+MHY1485 groups in N6/ADR cells **(A)** and 697/ADR cells **(B)** under 0, 5, 20, 80 nM Dox treatment.
