# Supplementary figures and images for: Targeting the lncRNA DUXAP8/miR-29a/PIK3CA Network Restores Doxorubicin Chemosensitivity via PI3K-AKT-mTOR Signaling and Synergizes With Inotuzumab Ozogamicin in Chemotherapy-Resistant B-Cell Acute Lymphoblastic Leukemia
Source: Front Oncol. 2022 Mar 2;12:773601. doi: 10.3389/fonc.2022.773601 (PMC8924619; doi:10.3389/fonc.2022.773601)

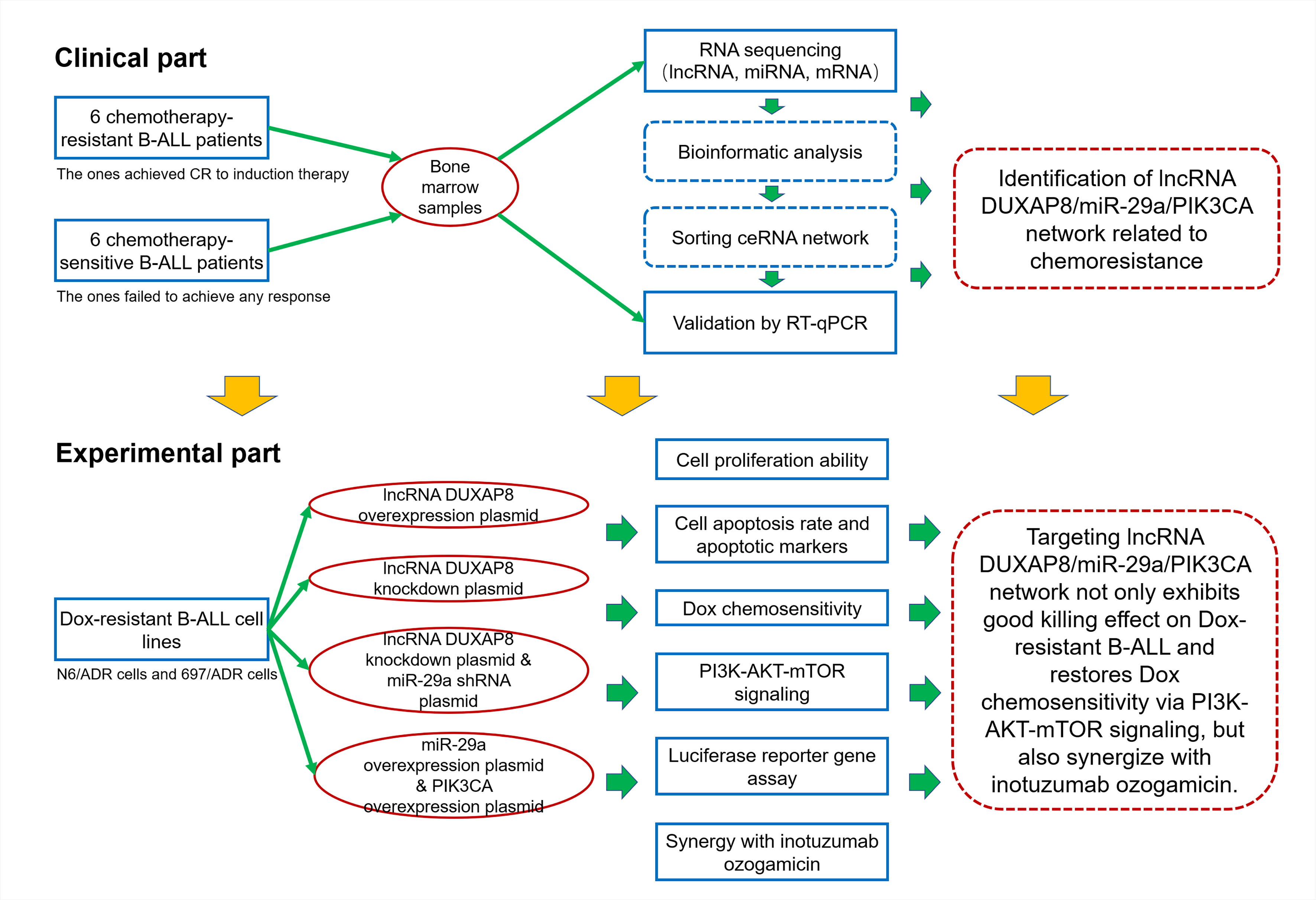

Supplement: Supplementary Figure 1 — Study flow chart. [file Image_1.tif]

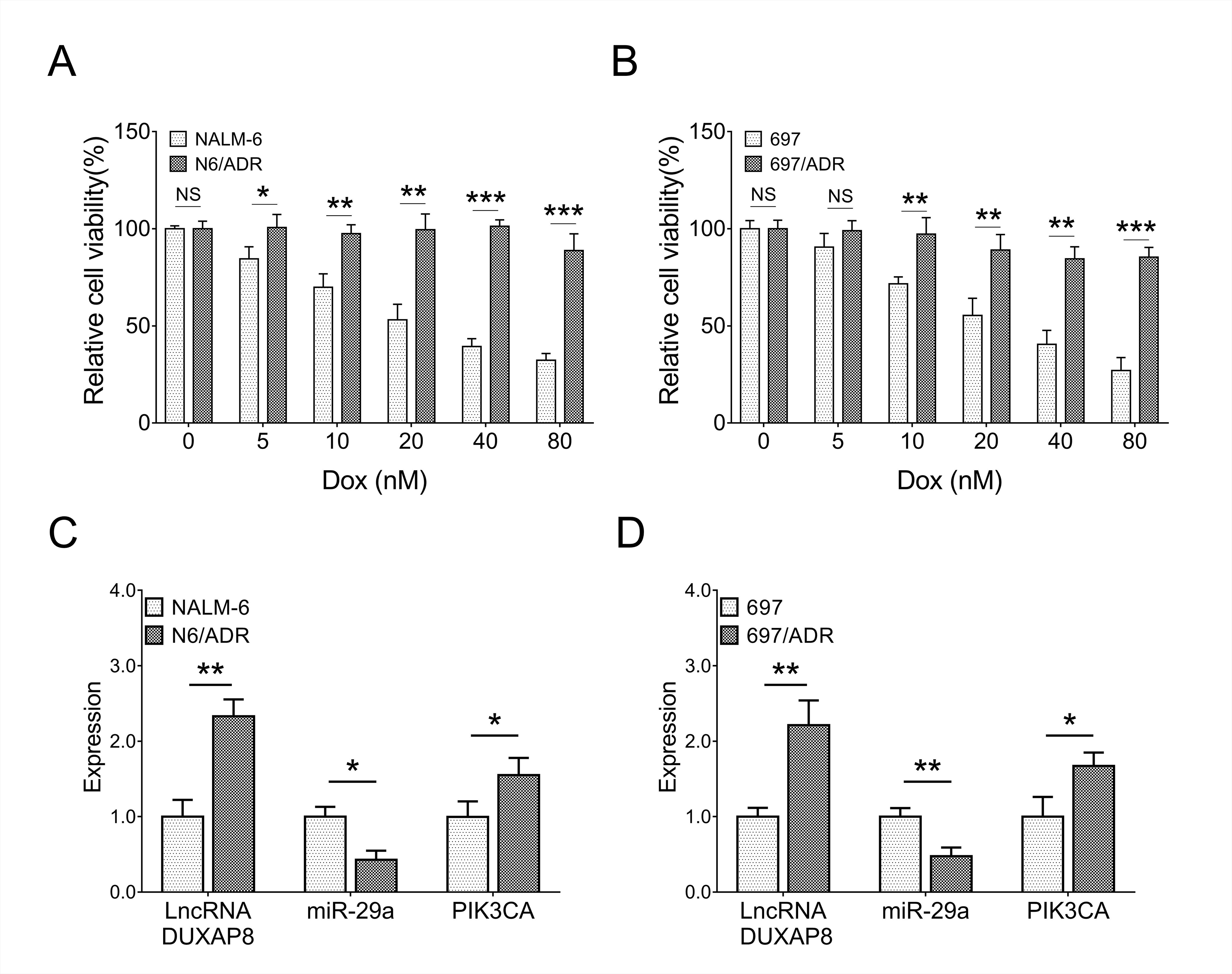

Supplement: Supplementary Figure 2 — Expression of lncRNA DUXAP8, miR-29a and PIK3CA in N6/ADR and 697/ADR cells. Confirmation of the Dox resistance status of N6/ADR (A) and 697/ADR cells (B). Expression of lncRNA DUXAP8, miR-29a and PIK3CA in N6/ADR and 697/ADR cells compared to normal NALM6 and 697 cells, respectively (C, D). [file Image_2.tif]

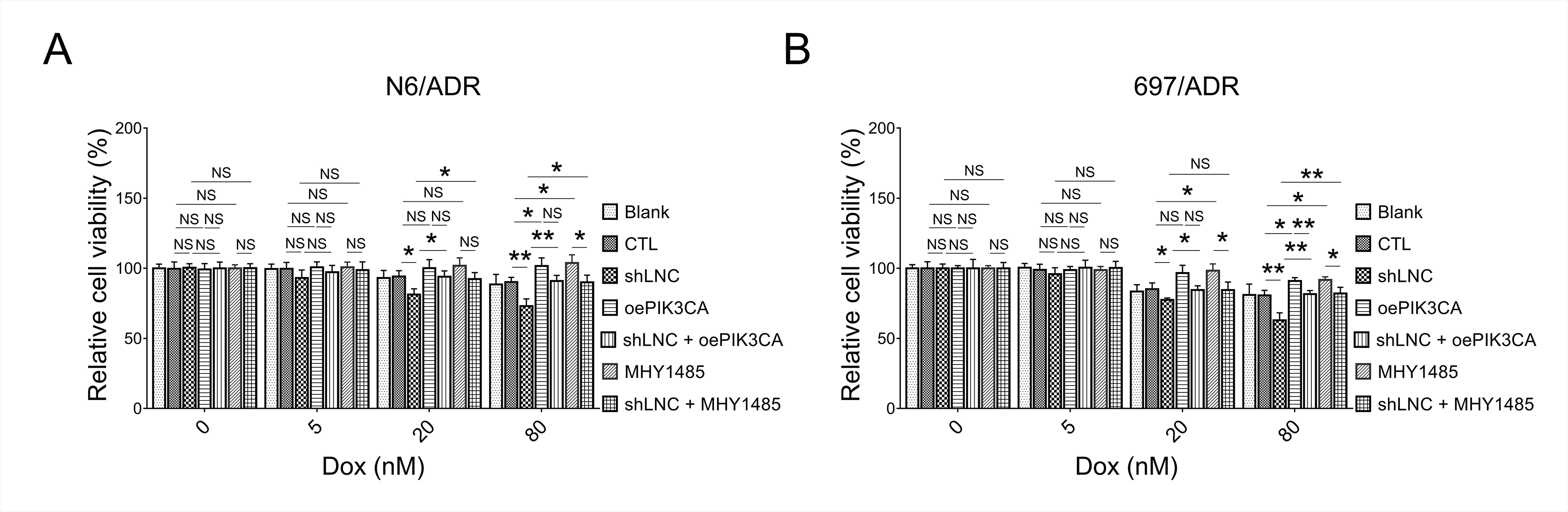

Supplement: Supplementary Figure 3 — Role of PI3K-AKT-mTOR pathway in this restored chemosensitivity. Relative cell viability among Blank, CTL, shLNC, oePIK3CA, shLNC+oePIK3CA, MHY1485, shLNC+MHY1485 groups in N6/ADR cells (A) and 697/ADR cells (B) under 0, 5, 20, 80 nM Dox treatment. [file Image_3.tif]

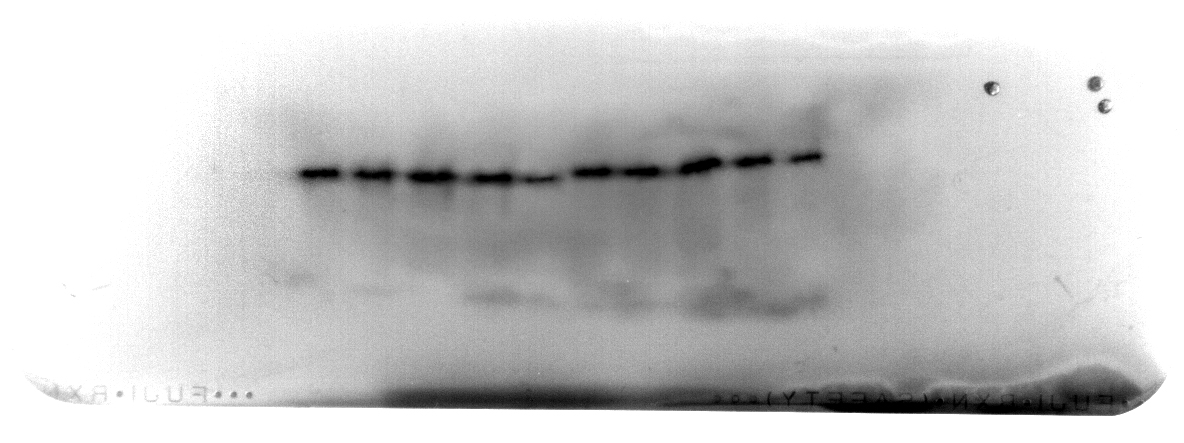

Supplement: Supplementary file 7 [file DataSheet_1.zip › Western Blot Original Image/Fig 3 Bcl-2.jpg]

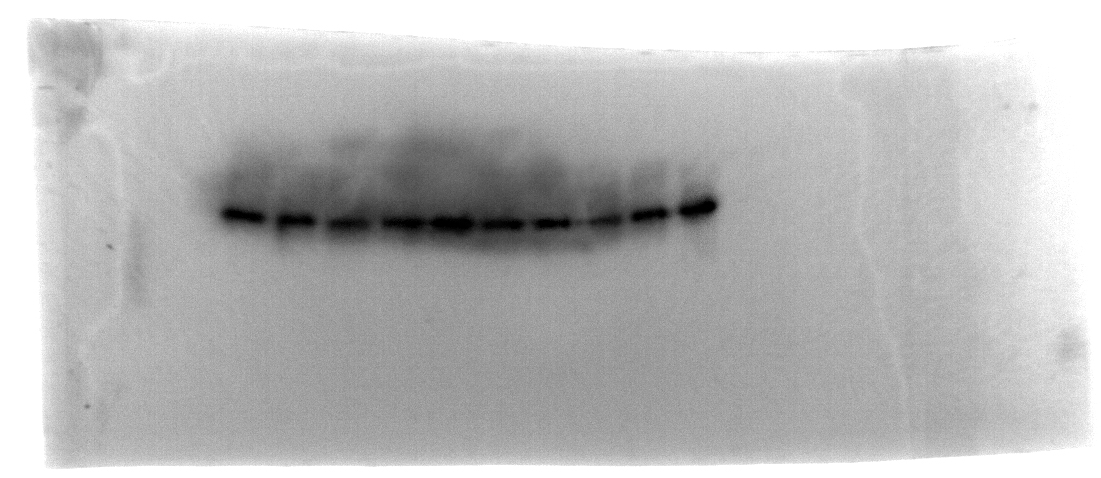

Supplement: Supplementary file 7 [file DataSheet_1.zip › Western Blot Original Image/Fig 3 cleaved caspase-3.jpg]

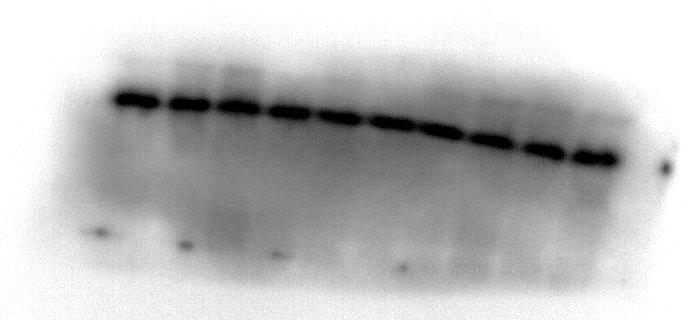

Supplement: Supplementary file 7 [file DataSheet_1.zip › Western Blot Original Image/Fig 3 a┬-actin.jpg]

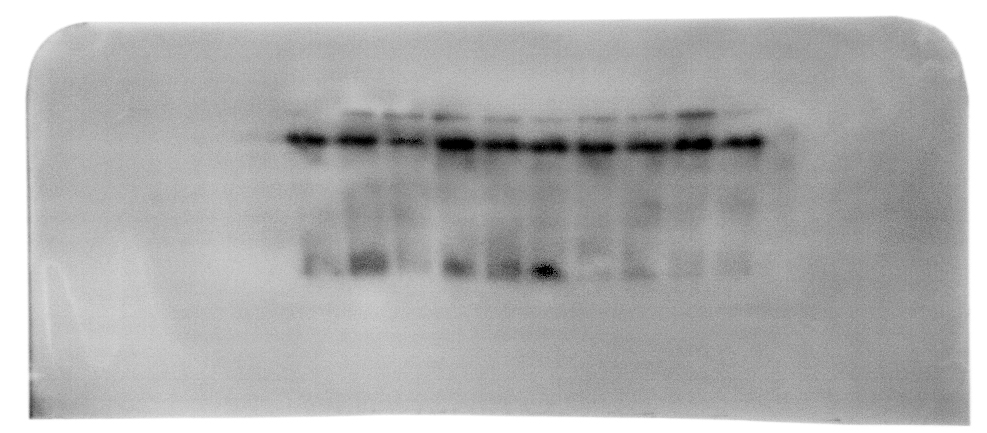

Supplement: Supplementary file 7 [file DataSheet_1.zip › Western Blot Original Image/Fig 5 Bcl-2.jpg]

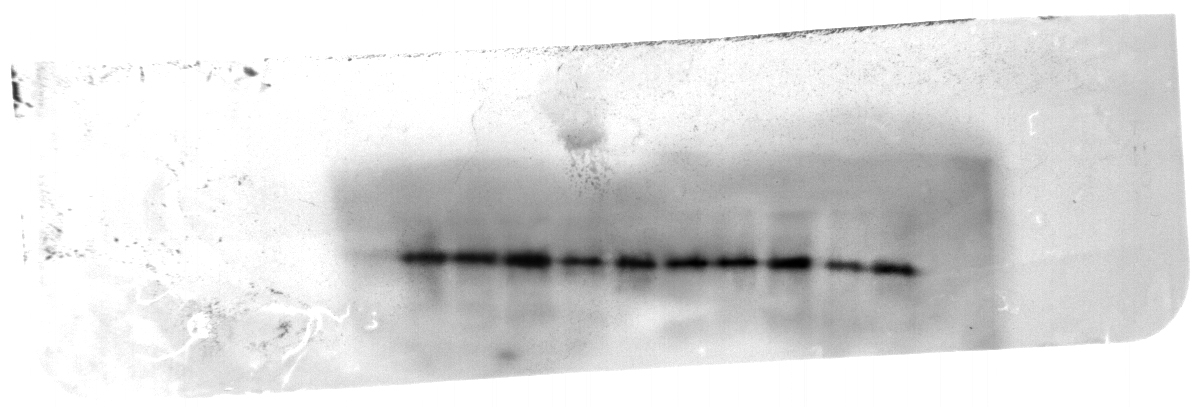

Supplement: Supplementary file 7 [file DataSheet_1.zip › Western Blot Original Image/Fig 5 cleaved caspase-3.jpg]

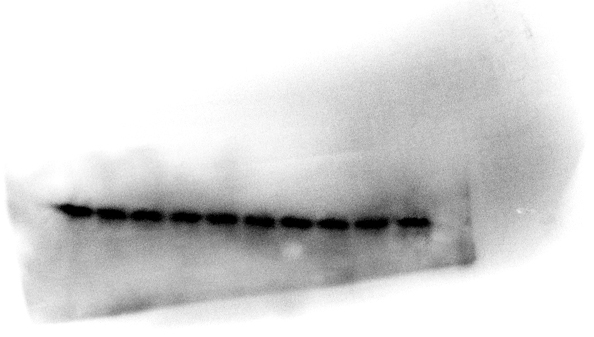

Supplement: Supplementary file 7 [file DataSheet_1.zip › Western Blot Original Image/Fig 5 a┬-actin.jpg]

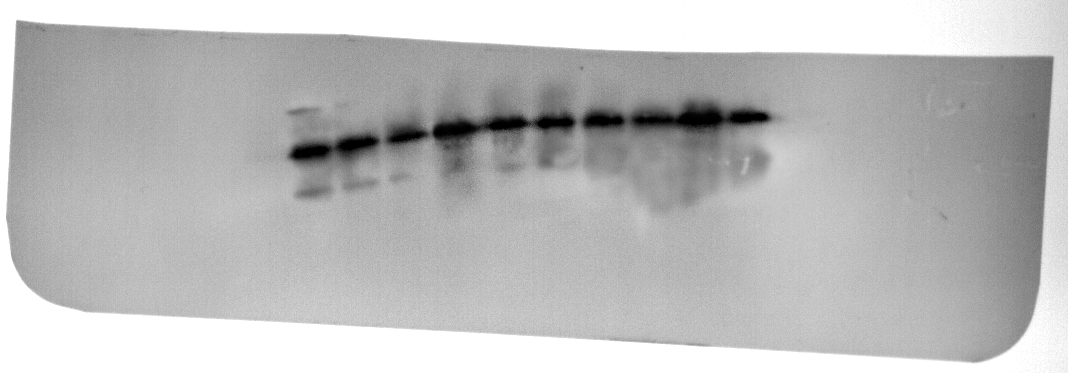

Supplement: Supplementary file 7 [file DataSheet_1.zip › Western Blot Original Image/Fig 6 PKI3CA.jpg]

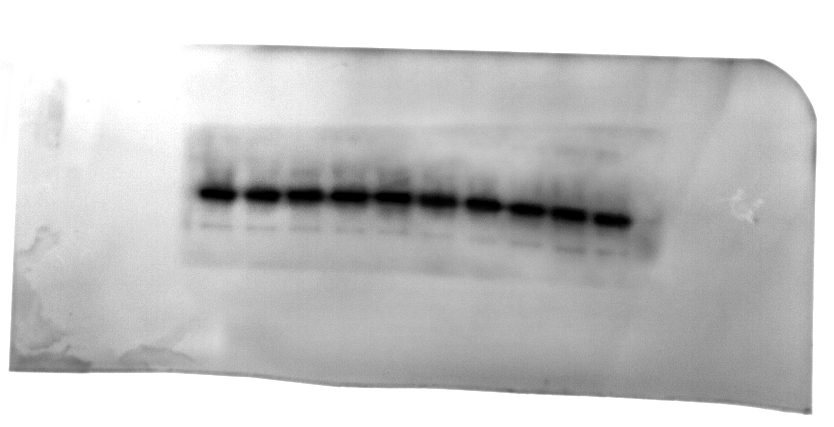

Supplement: Supplementary file 7 [file DataSheet_1.zip › Western Blot Original Image/Fig 6 a┬-actin.jpg]

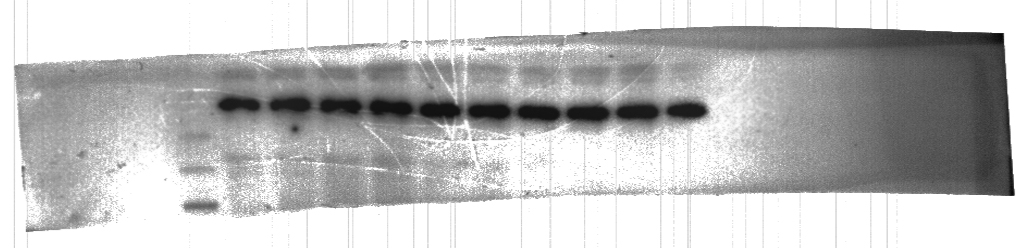

Supplement: Supplementary file 7 [file DataSheet_1.zip › Western Blot Original Image/Fig 7 AKT.jpg]

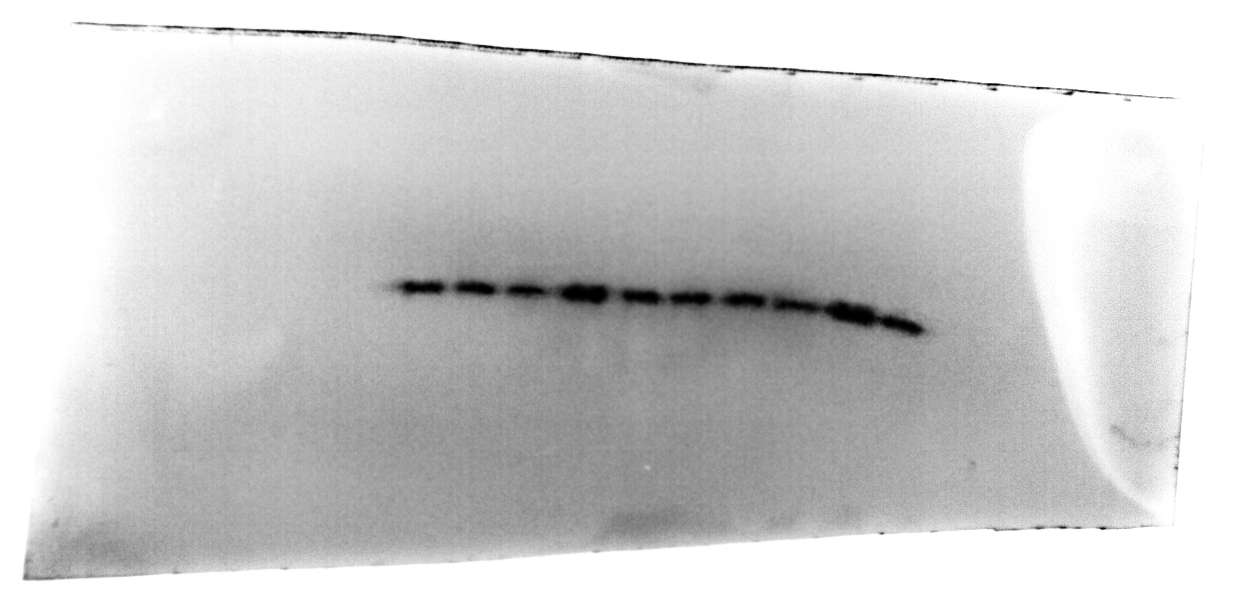

Supplement: Supplementary file 7 [file DataSheet_1.zip › Western Blot Original Image/Fig 7 PIK3CA.jpg]

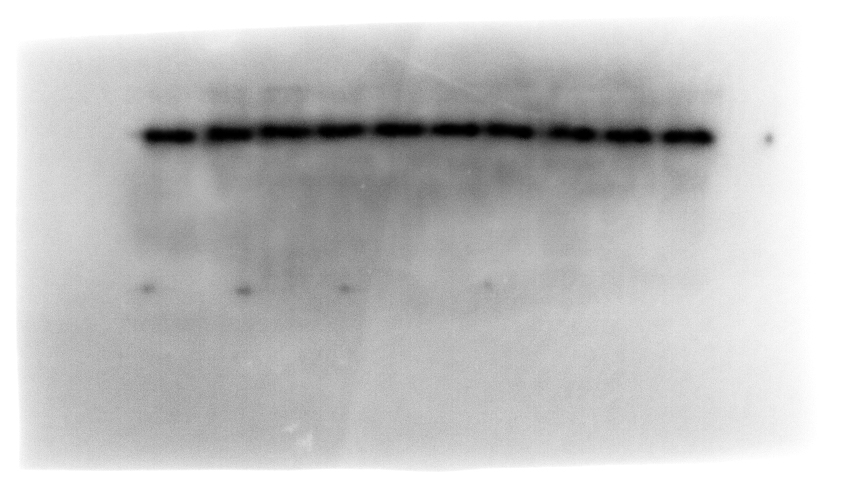

Supplement: Supplementary file 7 [file DataSheet_1.zip › Western Blot Original Image/Fig 7 S6K.jpg]

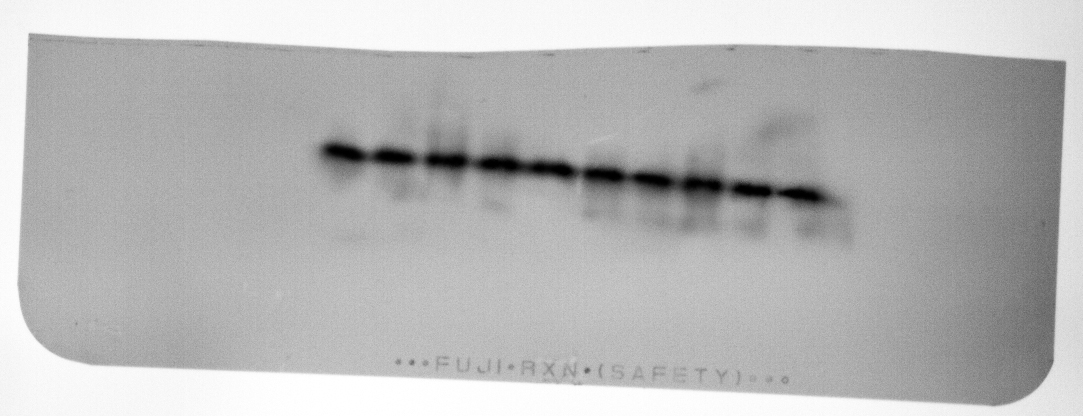

Supplement: Supplementary file 7 [file DataSheet_1.zip › Western Blot Original Image/Fig 7 mTOR.jpg]

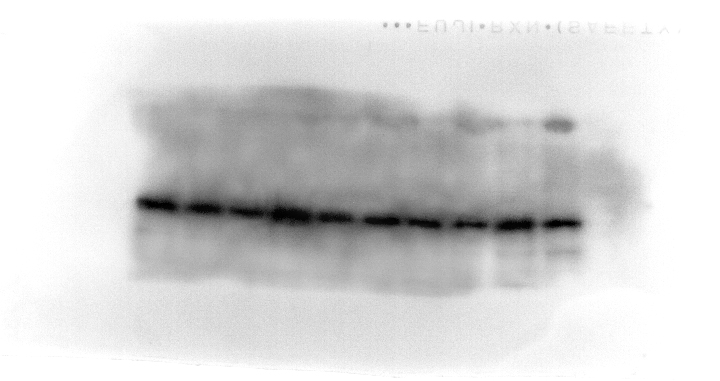

Supplement: Supplementary file 7 [file DataSheet_1.zip › Western Blot Original Image/Fig 7 pAKT.jpg]

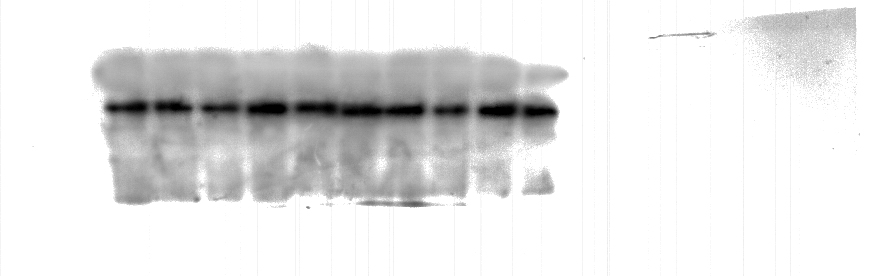

Supplement: Supplementary file 7 [file DataSheet_1.zip › Western Blot Original Image/Fig 7 pS6K.jpg]

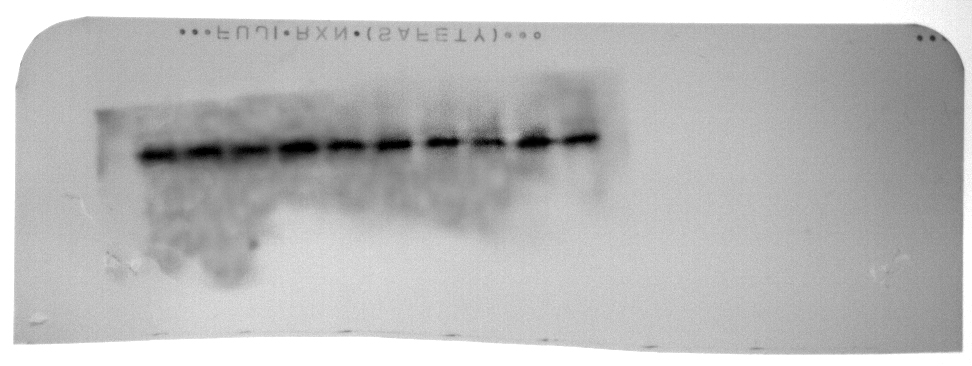

Supplement: Supplementary file 7 [file DataSheet_1.zip › Western Blot Original Image/Fig 7 pmTOR.jpg]

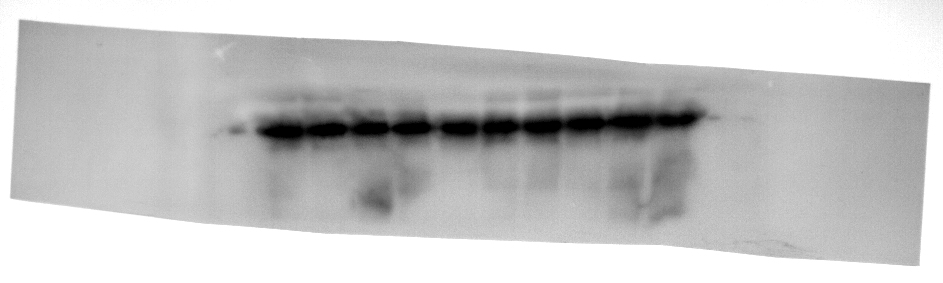

Supplement: Supplementary file 7 [file DataSheet_1.zip › Western Blot Original Image/Fig 7 a┬-actin.jpg]

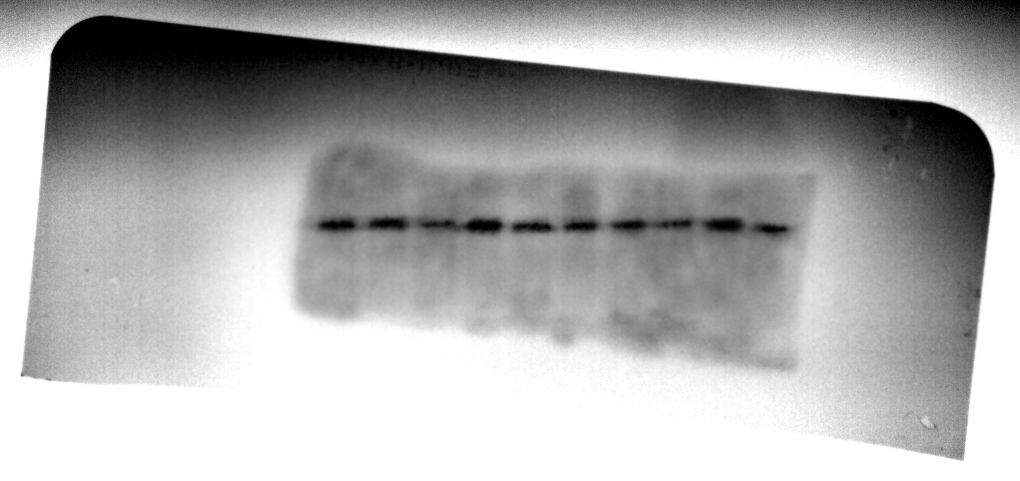

Supplement: Supplementary file 7 [file DataSheet_1.zip › Western Blot Original Image/Fig 8 Bcl-2.jpg]

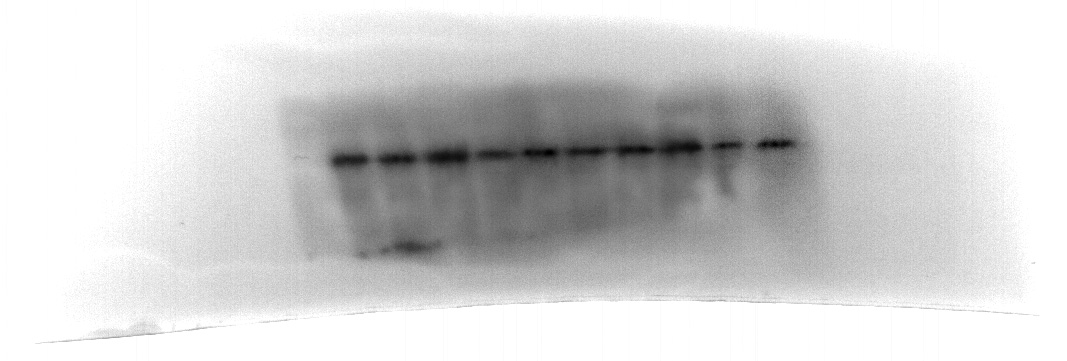

Supplement: Supplementary file 7 [file DataSheet_1.zip › Western Blot Original Image/Fig 8 cleaved caspse-3.jpg]

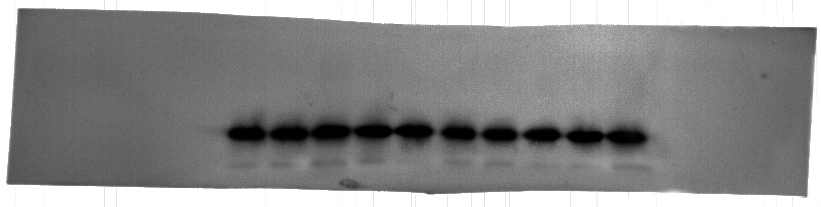

Supplement: Supplementary file 7 [file DataSheet_1.zip › Western Blot Original Image/Fig 8 a┬-actin.jpg]
