# Supplementary material for: Targeting the lncRNA DUXAP8/miR-29a/PIK3CA Network Restores Doxorubicin Chemosensitivity via PI3K-AKT-mTOR Signaling and Synergizes With Inotuzumab Ozogamicin in Chemotherapy-Resistant B-Cell Acute Lymphoblastic Leukemia
Source: Front Oncol. 2022 Mar 2;12:773601. doi: 10.3389/fonc.2022.773601 (PMC8924619; doi:10.3389/fonc.2022.773601)
Supplement: Supplementary file 4 [file Table_1.docx]

**Supplementary Table 1.** Dysregulated lncRNAs.

| Gene ID | Gene Symbol | Log_2_FoldChange | P | P_adj_ | Trend |
| --- | --- | --- | --- | --- | --- |
| ENSG00000157021 | FAM92A1P1 | 1.849463 | 5.77E-10 | 7.05E-06 | UP |
| ENSG00000166408 | OR5P1P | 1.056652 | 9.38E-07 | 0.000465 | UP |
| ENSG00000231976 | LINC00202-2 | 1.828239 | 1.50E-08 | 4.43E-05 | UP |
| ENSG00000174171 | RP11-23P13.6 | 1.322888 | 0.001548 | 0.030618 | UP |
| ENSG00000175147 | TMEM51-AS1 | 1.386395 | 1.96E-08 | 5.18E-05 | UP |
| ENSG00000176349 | AC110781.3 | 1.640607 | 6.16E-08 | 9.91E-05 | UP |
| ENSG00000178762 | HIST1H2BPS1 | 1.582683 | 5.52E-06 | 0.001269 | UP |
| ENSG00000178977 | LINC00324 | 1.473436 | 0.000302 | 0.012293 | UP |
| ENSG00000179066 | CTD-2527I21.15 | 1.823659 | 0.000417 | 0.014746 | UP |
| ENSG00000180539 | C9orf139 | 1.15381 | 5.73E-05 | 0.004796 | UP |
| ENSG00000181282 | OR5AK3P | 1.194294 | 2.46E-06 | 0.000803 | UP |
| ENSG00000182021 | RP11-381O7.3 | 1.247584 | 0.00018 | 0.009217 | UP |
| ENSG00000182057 | OGFRP1 | 1.198766 | 0.000824 | 0.021562 | UP |
| ENSG00000182109 | RP11-69E11.4 | 1.763839 | 2.87E-06 | 0.000877 | UP |
| ENSG00000182965 | NPM1P14 | 1.396806 | 6.30E-10 | 7.45E-06 | UP |
| ENSG00000183250 | LINC01547 | 1.147967 | 0.000275 | 0.011651 | UP |
| ENSG00000184441 | AP001062.7 | 1.349514 | 0.001033 | 0.02444 | UP |
| ENSG00000247373 | RP11-486O12.2 | 1.339881 | 0.000121 | 0.007337 | UP |
| ENSG00000186645 | SPDYE17 | 1.42884 | 1.59E-11 | 8.89E-07 | UP |
| ENSG00000186704 | DTX2P1 | 1.362485 | 5.01E-06 | 0.001202 | UP |
| ENSG00000187012 | LINC00207 | 1.576165 | 5.42E-12 | 4.93E-07 | UP |
| ENSG00000188185 | LINC00265 | 1.263879 | 4.57E-09 | 2.26E-05 | UP |
| ENSG00000188365 | AC092171.2 | 1.116034 | 0.000596 | 0.018023 | UP |
| ENSG00000189423 | USP32P3 | 1.264654 | 2.98E-12 | 3.49E-07 | UP |
| ENSG00000196566 | RP11-57C13.3 | 1.547499 | 4.22E-06 | 0.00109 | UP |
| ENSG00000197182 | MIRLET7BHG | 1.244679 | 0.001275 | 0.02746 | UP |
| ENSG00000197990 | ZNF734P | 1.279989 | 0.000125 | 0.007488 | UP |
| ENSG00000198618 | PPIAP22 | 1.913919 | 0.000172 | 0.008984 | UP |
| ENSG00000198788 | MUC2 | 2.00818 | 1.13E-11 | 7.27E-07 | UP |
| ENSG00000199282 | SNORA9 | 1.522558 | 2.44E-05 | 0.00296 | UP |
| ENSG00000199482 | RNU6-633P | 1.226381 | 8.29E-07 | 0.000434 | UP |
| ENSG00000199603 | RNU6-951P | 1.572109 | 1.04E-07 | 0.000134 | UP |
| ENSG00000199664 | RNU6-1266P | 1.104517 | 0.000135 | 0.007806 | UP |
| ENSG00000199857 | SNORD50 | 1.272573 | 2.10E-05 | 0.002722 | UP |
| ENSG00000200062 | RNU4-58P | 1.248759 | 0.000119 | 0.007268 | UP |
| ENSG00000200064 | RNY4P9 | 1.681259 | 3.35E-08 | 7.04E-05 | UP |
| ENSG00000200215 | SNORD113-6 | 2.179257 | 2.85E-06 | 0.000875 | UP |
| ENSG00000200774 | RNU6-478P | 1.246353 | 3.67E-11 | 1.44E-06 | UP |
| ENSG00000201793 | RN7SKP9 | 2.647085 | 3.49E-09 | 1.96E-05 | UP |
| ENSG00000202000 | RNU1-36P | 1.656013 | 4.12E-05 | 0.003979 | UP |
| ENSG00000202296 | RNU6-1335P | 1.246902 | 0.00261 | 0.040908 | UP |
| ENSG00000202427 | RNU6-744P | 1.255845 | 0.001929 | 0.034539 | UP |
| ENSG00000203799 | CCDC162P | 1.196543 | 0.000462 | 0.015583 | UP |
| ENSG00000205583 | STAG3L1 | 1.153042 | 0.003003 | 0.044254 | UP |
| ENSG00000205885 | C1RL-AS1 | 1.254477 | 1.04E-13 | 5.37E-08 | UP |
| ENSG00000206356 | RP11-93O17.2 | 1.250209 | 6.27E-08 | 0.0001 | UP |
| ENSG00000206760 | SNORA6 | 1.17789 | 8.82E-05 | 0.006157 | UP |
| ENSG00000206875 | RNU6-761P | 2.134417 | 0.000305 | 0.012358 | UP |
| ENSG00000206991 | RNU6-610P | 1.021121 | 0.001828 | 0.033562 | UP |
| ENSG00000207310 | RNU6-1127P | 1.097039 | 1.98E-07 | 0.000194 | UP |
| ENSG00000207327 | RNU6-883P | 1.522853 | 7.78E-11 | 2.23E-06 | UP |
| ENSG00000211770 | TRBJ2-6 | 1.455711 | 0.003663 | 0.049534 | UP |
| ENSG00000211771 | TRBJ2-7 | 1.580324 | 1.81E-06 | 0.000678 | UP |
| ENSG00000211857 | TRAJ32 | 1.025777 | 2.90E-05 | 0.003268 | UP |
| ENSG00000212460 | RNU6-460P | 1.588248 | 0.000125 | 0.007474 | UP |
| ENSG00000212952 | RP11-475I24.9 | 1.139186 | 0.001679 | 0.031984 | UP |
| ENSG00000213032 | NDUFA3P3 | 1.021483 | 2.58E-05 | 0.003055 | UP |
| ENSG00000213108 | BTF3L4P3 | 1.043035 | 2.13E-06 | 0.00074 | UP |
| ENSG00000213172 | RP1-228H13.2 | 2.328432 | 1.85E-06 | 0.000685 | UP |
| ENSG00000213216 | RP11-355O1.7 | 1.450663 | 0.000449 | 0.015382 | UP |
| ENSG00000213277 | MARCKSL1P1 | 1.263727 | 1.47E-06 | 0.000602 | UP |
| ENSG00000213279 | RP1-29C18.9 | 3.087615 | 4.41E-11 | 1.60E-06 | UP |
| ENSG00000213393 | RP11-546B8.1 | 1.068742 | 0.000963 | 0.023499 | UP |
| ENSG00000213536 | GNG5P1 | 1.60933 | 1.69E-09 | 1.31E-05 | UP |
| ENSG00000213730 | POLD2P1 | 1.430685 | 0.000153 | 0.008373 | UP |
| ENSG00000213731 | RAB5CP1 | 2.183397 | 3.82E-07 | 0.000279 | UP |
| ENSG00000213744 | RPS10P14 | 1.223667 | 0.001604 | 0.031227 | UP |
| ENSG00000213943 | KRT18P17 | 1.041243 | 4.31E-05 | 0.004076 | UP |
| ENSG00000214051 | ARF4P3 | 1.205997 | 2.78E-10 | 4.59E-06 | UP |
| ENSG00000214062 | RPL7P17 | 1.316877 | 6.20E-06 | 0.001359 | UP |
| ENSG00000214211 | CTAGE14P | 1.842299 | 1.56E-09 | 1.25E-05 | UP |
| ENSG00000214279 | SCART1 | 1.743413 | 8.38E-06 | 0.001619 | UP |
| ENSG00000214288 | HMGB3P13 | 1.665451 | 3.81E-05 | 0.003797 | UP |
| ENSG00000214359 | RPL18P10 | 1.251019 | 0.00018 | 0.009193 | UP |
| ENSG00000214578 | HMGN2P15 | 1.004369 | 0.001287 | 0.0276 | UP |
| ENSG00000214626 | POLR3DP1 | 1.320915 | 0.002869 | 0.043145 | UP |
| ENSG00000214650 | RP11-83B20.1 | 1.64656 | 0.000725 | 0.020046 | UP |
| ENSG00000214973 | CHCHD3P3 | 1.447908 | 0.001195 | 0.026515 | UP |
| ENSG00000215458 | AATBC | 1.412434 | 3.66E-13 | 1.09E-07 | UP |
| ENSG00000215769 | RP13-104F24.2 | 1.312735 | 0.00011 | 0.006963 | UP |
| ENSG00000215867 | KRT18P57 | 1.81312 | 3.59E-09 | 1.99E-05 | UP |
| ENSG00000216775 | RP1-152L7.5 | 1.235208 | 4.40E-13 | 1.20E-07 | UP |
| ENSG00000218073 | RP1-13D10.2 | 1.528426 | 8.17E-08 | 0.000117 | UP |
| ENSG00000218809 | RP1-229K20.5 | 1.13074 | 8.33E-06 | 0.001615 | UP |
| ENSG00000218976 | RP11-528A10.1 | 1.347268 | 0.00362 | 0.049193 | UP |
| ENSG00000218991 | CCNG1P1 | 1.398479 | 1.08E-07 | 0.000137 | UP |
| ENSG00000219712 | RP11-532F6.2 | 1.616131 | 5.81E-07 | 0.000353 | UP |
| ENSG00000219738 | XXbac-BPG34I8.3 | 1.313768 | 0.000256 | 0.011206 | UP |
| ENSG00000222094 | RNU2-65P | 1.920243 | 9.30E-05 | 0.00634 | UP |
| ENSG00000222371 | RN7SKP202 | 1.509311 | 0.001272 | 0.027433 | UP |
| ENSG00000222490 | RNU6-712P | 1.090077 | 0.000554 | 0.017321 | UP |
| ENSG00000222533 | RNU6-705P | 2.105865 | 8.21E-07 | 0.000431 | UP |
| ENSG00000222923 | RNU2-41P | 1.010237 | 3.57E-08 | 7.27E-05 | UP |
| ENSG00000223096 | RNU5E-9P | 1.437698 | 0.000581 | 0.017763 | UP |
| ENSG00000223581 | RP11-393K12.2 | 2.702401 | 5.72E-07 | 0.00035 | UP |
| ENSG00000223705 | NSUN5P1 | 1.03828 | 2.22E-05 | 0.002808 | UP |
| ENSG00000223725 | AC007879.5 | 1.373901 | 0.002611 | 0.040908 | UP |
| ENSG00000223850 | MYCNUT | 1.867421 | 1.27E-05 | 0.002057 | UP |
| ENSG00000223896 | CCNJP2 | 2.452088 | 1.66E-06 | 0.000644 | UP |
| ENSG00000223899 | SEC13P1 | 2.118539 | 7.63E-06 | 0.001533 | UP |
| ENSG00000223907 | LINC01226 | 2.179842 | 1.67E-05 | 0.002398 | UP |
| ENSG00000223995 | RPL32P35 | 1.340011 | 0.000208 | 0.010008 | UP |
| ENSG00000224025 | RP11-274B18.3 | 1.405607 | 0.002374 | 0.038832 | UP |
| ENSG00000224035 | SFPQP1 | 1.029557 | 9.09E-06 | 0.001698 | UP |
| ENSG00000224134 | AC004866.3 | 1.363212 | 3.13E-05 | 0.003403 | UP |
| ENSG00000224186 | C5orf66 | 1.324793 | 7.47E-06 | 0.001513 | UP |
| ENSG00000224203 | RPS23P10 | 1.322873 | 0.001422 | 0.029209 | UP |
| ENSG00000224261 | RPSAP18 | 1.892223 | 2.18E-05 | 0.002781 | UP |
| ENSG00000224353 | ACE3P | 1.067566 | 0.000986 | 0.023808 | UP |
| ENSG00000224448 | GS1-259H13.7 | 1.262829 | 0.001127 | 0.025688 | UP |
| ENSG00000224669 | RP11-561N12.1 | 2.07257 | 1.27E-05 | 0.002049 | UP |
| ENSG00000224675 | AC009227.2 | 1.428052 | 3.52E-08 | 7.22E-05 | UP |
| ENSG00000224727 | FCF1P7 | 1.370927 | 0.001995 | 0.035195 | UP |
| ENSG00000224791 | KRT18P39 | 1.610476 | 0.0015 | 0.030068 | UP |
| ENSG00000224888 | RP5-1142A6.2 | 1.008419 | 0.002093 | 0.036124 | UP |
| ENSG00000224975 | INE1 | 1.338497 | 2.95E-05 | 0.003298 | UP |
| ENSG00000225032 | RP11-228B15.4 | 1.437367 | 8.95E-06 | 0.001684 | UP |
| ENSG00000225092 | RP11-405O10.2 | 1.497005 | 1.21E-05 | 0.001995 | UP |
| ENSG00000225195 | RP11-338E21.2 | 1.548954 | 7.04E-07 | 0.000395 | UP |
| ENSG00000225213 | RP11-197M22.2 | 1.708294 | 2.09E-05 | 0.002714 | UP |
| ENSG00000225222 | CHCHD4P5 | 1.556821 | 0.001552 | 0.030669 | UP |
| ENSG00000225234 | TRAPPC12-AS1 | 1.064309 | 0.001269 | 0.027404 | UP |
| ENSG00000225300 | RP11-1086F11.1 | 1.576755 | 0.000119 | 0.00729 | UP |
| ENSG00000225399 | RP11-3B7.1 | 1.406811 | 0.000861 | 0.022081 | UP |
| ENSG00000225401 | TGIF2P1 | 1.183769 | 0.003201 | 0.045875 | UP |
| ENSG00000225803 | MRPS11P1 | 1.011768 | 0.001835 | 0.033621 | UP |
| ENSG00000225947 | RP11-313E4.1 | 2.033742 | 0.000345 | 0.01325 | UP |
| ENSG00000225982 | RP11-538D16.3 | 1.134421 | 0.00023 | 0.010571 | UP |
| ENSG00000226070 | RP11-552E20.4 | 1.338874 | 1.66E-05 | 0.002385 | UP |
| ENSG00000226144 | RPS27AP3 | 1.143169 | 0.000851 | 0.021952 | UP |
| ENSG00000226148 | SLC25A39P1 | 1.114374 | 0.000123 | 0.007414 | UP |
| ENSG00000226272 | ARHGAP26-AS1 | 1.301644 | 0.000234 | 0.010664 | UP |
| ENSG00000226284 | ARPC3P1 | 1.955068 | 0.000261 | 0.011332 | UP |
| ENSG00000226332 | RP11-157P1.4 | 1.315776 | 0.002488 | 0.039874 | UP |
| ENSG00000226340 | AC105402.3 | 1.049703 | 5.66E-11 | 1.85E-06 | UP |
| ENSG00000226396 | RP5-1056L3.3 | 1.193609 | 0.000655 | 0.018938 | UP |
| ENSG00000226411 | CTD-2526L21.2 | 1.143618 | 8.72E-07 | 0.000446 | UP |
| ENSG00000226421 | SLC25A5P5 | 1.069333 | 2.40E-05 | 0.002935 | UP |
| ENSG00000226450 | CYP2D8P | 1.401973 | 0.000105 | 0.006771 | UP |
| ENSG00000226498 | RPSAP21 | 1.201079 | 0.00303 | 0.044477 | UP |
| ENSG00000226533 | BTBD9-AS1 | 1.086726 | 0.000971 | 0.02361 | UP |
| ENSG00000226705 | SDCBPP1 | 2.225981 | 4.82E-05 | 0.004343 | UP |
| ENSG00000226746 | SMCR5 | 1.405027 | 0.002451 | 0.039559 | UP |
| ENSG00000226747 | AC007966.1 | 1.277148 | 1.95E-06 | 0.000705 | UP |
| ENSG00000226824 | RP4-756H11.3 | 1.879612 | 6.62E-06 | 0.001411 | UP |
| ENSG00000226972 | RPL12P19 | 1.21162 | 1.24E-05 | 0.002025 | UP |
| ENSG00000227032 | RP11-34E5.4 | 1.237718 | 6.96E-06 | 0.001452 | UP |
| ENSG00000227039 | ITGB2-AS1 | 1.140147 | 3.08E-06 | 0.000915 | UP |
| ENSG00000227155 | RP11-165F24.3 | 1.094097 | 3.39E-05 | 0.003559 | UP |
| ENSG00000227182 | VN1R28P | 1.426796 | 1.68E-11 | 9.19E-07 | UP |
| ENSG00000227187 | RP11-6C10.1 | 1.133488 | 6.16E-06 | 0.001354 | UP |
| ENSG00000227205 | PFN1P9 | 1.010208 | 1.88E-15 | 5.53E-09 | UP |
| ENSG00000227218 | RP11-203J24.8 | 1.055108 | 0.001659 | 0.031808 | UP |
| ENSG00000227456 | LINC00310 | 1.731058 | 0.001052 | 0.024698 | UP |
| ENSG00000227475 | RP11-810B23.1 | 1.044311 | 4.44E-06 | 0.001124 | UP |
| ENSG00000227508 | LINC01624 | 1.209552 | 0.000267 | 0.011466 | UP |
| ENSG00000227574 | RP13-1039J1.3 | 1.089083 | 2.52E-05 | 0.003017 | UP |
| ENSG00000227627 | RP1-101K10.6 | 2.08186 | 3.24E-09 | 1.88E-05 | UP |
| ENSG00000227702 | LINC00111 | 1.997901 | 0.000498 | 0.01628 | UP |
| ENSG00000227782 | CTC-529I10.2 | 1.018287 | 0.003645 | 0.049392 | UP |
| ENSG00000227920 | RP1-153P14.5 | 1.500209 | 0.000584 | 0.017806 | UP |
| ENSG00000227946 | AC007383.3 | 1.459229 | 1.96E-06 | 0.000706 | UP |
| ENSG00000228058 | RP11-552D4.1 | 1.212736 | 0.001273 | 0.027436 | UP |
| ENSG00000228257 | AC010141.6 | 2.215433 | 1.40E-14 | 1.77E-08 | UP |
| ENSG00000228264 | PSMD8P1 | 1.63448 | 1.11E-07 | 0.000139 | UP |
| ENSG00000228302 | RP11-186N15.3 | 1.197365 | 1.45E-08 | 4.33E-05 | UP |
| ENSG00000228432 | DHFRP2 | 1.867034 | 1.92E-10 | 3.76E-06 | UP |
| ENSG00000228473 | LIN28AP2 | 1.156051 | 1.28E-06 | 0.000555 | UP |
| ENSG00000228505 | AC011897.2 | 1.072651 | 1.98E-06 | 0.000709 | UP |
| ENSG00000228568 | AC006461.2 | 1.687438 | 4.82E-06 | 0.001175 | UP |
| ENSG00000228681 | AC008072.1 | 1.008494 | 0.000114 | 0.007121 | UP |
| ENSG00000228768 | AC003101.1 | 1.372062 | 1.18E-05 | 0.001969 | UP |
| ENSG00000228776 | RP11-319C21.1 | 1.360866 | 1.96E-07 | 0.000193 | UP |
| ENSG00000228783 | RP11-147I11.1 | 1.100482 | 3.43E-05 | 0.003581 | UP |
| ENSG00000228851 | RP11-1217F2.10 | 1.016295 | 0.001233 | 0.026979 | UP |
| ENSG00000228903 | RASA4CP | 1.12947 | 7.87E-06 | 0.001562 | UP |
| ENSG00000228981 | RP11-364L4.1 | 1.67373 | 8.24E-05 | 0.005918 | UP |
| ENSG00000229013 | AC083939.1 | 1.218963 | 4.14E-06 | 0.001078 | UP |
| ENSG00000229023 | AC067945.3 | 1.660774 | 0.000324 | 0.012797 | UP |
| ENSG00000229106 | BTBD6P1 | 1.339836 | 3.53E-05 | 0.003644 | UP |
| ENSG00000229151 | RP11-348F1.3 | 1.119342 | 0.00022 | 0.010297 | UP |
| ENSG00000229180 | GS1-124K5.11 | 1.219053 | 1.89E-05 | 0.002562 | UP |
| ENSG00000229533 | AC003986.5 | 1.286373 | 0.00056 | 0.017438 | UP |
| ENSG00000229569 | RP11-481G8.2 | 1.589573 | 6.31E-08 | 0.0001 | UP |
| ENSG00000229619 | MBNL1-AS1 | 1.09098 | 5.16E-07 | 0.00033 | UP |
| ENSG00000229687 | RP4-654H19.2 | 1.095053 | 1.99E-05 | 0.002637 | UP |
| ENSG00000229731 | RP11-501H19.2 | 1.739724 | 5.31E-05 | 0.0046 | UP |
| ENSG00000229791 | RP11-385E5.5 | 1.171906 | 0.000552 | 0.017297 | UP |
| ENSG00000229852 | RP11-398K22.12 | 1.296467 | 8.51E-06 | 0.001636 | UP |
| ENSG00000229875 | EIF1P1 | 1.639458 | 5.34E-08 | 9.12E-05 | UP |
| ENSG00000229893 | AC004549.6 | 1.035284 | 0.001504 | 0.030104 | UP |
| ENSG00000230018 | RP11-481H12.1 | 1.712214 | 5.05E-06 | 0.001207 | UP |
| ENSG00000230126 | FGF12-AS2 | 1.04867 | 2.59E-07 | 0.000224 | UP |
| ENSG00000230148 | HOXB-AS1 | 1.801797 | 0.000362 | 0.013615 | UP |
| ENSG00000230226 | RP4-697K14.3 | 1.38219 | 9.99E-14 | 5.25E-08 | UP |
| ENSG00000230299 | AC093162.3 | 1.545593 | 0.000239 | 0.010777 | UP |
| ENSG00000230359 | TPI1P2 | 1.258041 | 3.87E-05 | 0.003829 | UP |
| ENSG00000230365 | RP11-23D5.1 | 2.246845 | 2.78E-06 | 0.000862 | UP |
| ENSG00000230482 | ATP5G2P3 | 2.027531 | 2.28E-11 | 1.09E-06 | UP |
| ENSG00000230491 | RP13-228J13.10 | 1.487824 | 4.73E-06 | 0.001163 | UP |
| ENSG00000230542 | LINC00102 | 1.2251 | 2.62E-08 | 6.10E-05 | UP |
| ENSG00000230605 | MTCYBP6 | 1.216261 | 0.002258 | 0.037724 | UP |
| ENSG00000230638 | RP11-486B10.4 | 1.120924 | 4.45E-09 | 2.23E-05 | UP |
| ENSG00000230724 | LINC01001 | 1.01307 | 0.001579 | 0.030975 | UP |
| ENSG00000230749 | MEIS1-AS2 | 1.323174 | 2.95E-06 | 0.000892 | UP |
| ENSG00000230758 | SNAP23P | 1.916629 | 1.42E-08 | 4.28E-05 | UP |
| ENSG00000230815 | RP11-49O14.3 | 1.270782 | 0.001754 | 0.032779 | UP |
| ENSG00000230908 | XXyac-YX60D10.1 | 1.173909 | 0.000138 | 0.007912 | UP |
| ENSG00000230997 | RAB42P1 | 1.534737 | 2.61E-05 | 0.003073 | UP |
| ENSG00000231102 | RP11-298J23.5 | 1.038145 | 1.07E-05 | 0.001869 | UP |
| ENSG00000231103 | PRAMEF30P | 1.037398 | 2.06E-07 | 0.000198 | UP |
| ENSG00000231138 | RP11-391M7.3 | 1.45227 | 0.001118 | 0.025566 | UP |
| ENSG00000206195 | DUXAP8 | 3.209942 | 1.07E-09 | 1.00E-05 | UP |
| ENSG00000231312 | AC007246.3 | 1.341554 | 0.001416 | 0.029148 | UP |
| ENSG00000231460 | RP11-321L2.1 | 1.268183 | 6.27E-06 | 0.001368 | UP |
| ENSG00000231484 | RP11-548K12.12 | 1.338307 | 0.00054 | 0.017066 | UP |
| ENSG00000231552 | IGBP1P3 | 1.746104 | 1.61E-06 | 0.000633 | UP |
| ENSG00000231597 | AC007557.4 | 2.029803 | 1.85E-06 | 0.000685 | UP |
| ENSG00000231621 | AC013264.2 | 1.304989 | 0.000108 | 0.006878 | UP |
| ENSG00000231698 | AP002856.5 | 1.044978 | 0.000334 | 0.013011 | UP |
| ENSG00000231716 | CDY23P | 1.544095 | 1.53E-12 | 2.38E-07 | UP |
| ENSG00000231831 | MTHFD1P1 | 1.099553 | 0.000578 | 0.01772 | UP |
| ENSG00000231977 | RP5-963E22.4 | 1.627979 | 7.90E-09 | 3.07E-05 | UP |
| ENSG00000231987 | RP5-898J17.1 | 2.222567 | 1.40E-05 | 0.002174 | UP |
| ENSG00000232063 | RP11-307E17.8 | 1.164075 | 0.000352 | 0.01341 | UP |
| ENSG00000232254 | CSF2RBP1 | 1.510116 | 5.63E-05 | 0.004751 | UP |
| ENSG00000232354 | VIPR1-AS1 | 1.131066 | 2.77E-07 | 0.000231 | UP |
| ENSG00000232387 | SKA2P1 | 1.761056 | 7.35E-07 | 0.000405 | UP |
| ENSG00000232487 | RASA3-IT1 | 2.790129 | 6.36E-08 | 0.000101 | UP |
| ENSG00000232511 | OR2AH1P | 1.053371 | 0.000839 | 0.021779 | UP |
| ENSG00000232600 | TONSL-AS1 | 1.222692 | 1.30E-06 | 0.000561 | UP |
| ENSG00000232736 | AC007551.2 | 1.046275 | 0.000442 | 0.015232 | UP |
| ENSG00000232775 | BMS1P22 | 1.169081 | 6.44E-07 | 0.000375 | UP |
| ENSG00000232807 | RP11-536K7.3 | 1.323394 | 1.31E-06 | 0.000563 | UP |
| ENSG00000232832 | LMLN-AS1 | 1.748591 | 5.44E-06 | 0.00126 | UP |
| ENSG00000232881 | RPS10P21 | 1.556154 | 5.52E-05 | 0.004703 | UP |
| ENSG00000232888 | RPS11P5 | 1.145637 | 0.000309 | 0.012442 | UP |
| ENSG00000232896 | RP11-410K21.2 | 1.05029 | 0.000102 | 0.006679 | UP |
| ENSG00000232958 | AC091069.1 | 1.179114 | 9.80E-07 | 0.000477 | UP |
| ENSG00000233038 | AC011899.9 | 1.145265 | 6.81E-05 | 0.005293 | UP |
| ENSG00000233129 | RP5-837O21.2 | 1.111584 | 0.000909 | 0.022768 | UP |
| ENSG00000233175 | CTD-2020K17.3 | 1.510051 | 5.74E-06 | 0.001299 | UP |
| ENSG00000233178 | RP11-88I18.2 | 2.583852 | 2.74E-08 | 6.23E-05 | UP |
| ENSG00000233183 | RP3-468B3.2 | 1.375605 | 4.43E-10 | 6.09E-06 | UP |
| ENSG00000233406 | RP11-296A18.5 | 1.070258 | 0.000497 | 0.016255 | UP |
| ENSG00000233622 | CYP2T1P | 1.106049 | 3.55E-05 | 0.003652 | UP |
| ENSG00000233750 | CICP27 | 1.883391 | 8.63E-06 | 0.001649 | UP |
| ENSG00000233783 | AP001442.2 | 1.95583 | 1.21E-06 | 0.000539 | UP |
| ENSG00000233825 | RP11-135A24.4 | 1.441226 | 6.50E-05 | 0.005158 | UP |
| ENSG00000233845 | AC093732.1 | 1.051575 | 0.002671 | 0.041462 | UP |
| ENSG00000233850 | AC103563.8 | 1.123996 | 0.000495 | 0.016226 | UP |
| ENSG00000233895 | RP1-122P22.2 | 1.546545 | 0.000543 | 0.017133 | UP |
| ENSG00000233913 | CTC-575D19.1 | 3.075666 | 1.03E-12 | 1.93E-07 | UP |
| ENSG00000233937 | CTC-338M12.4 | 1.435975 | 3.28E-05 | 0.003497 | UP |
| ENSG00000233953 | AC009970.1 | 1.526462 | 6.55E-05 | 0.005183 | UP |
| ENSG00000234066 | TAS2R62P | 1.646531 | 0.002694 | 0.04164 | UP |
| ENSG00000234124 | CSN1S2AP | 1.024152 | 8.14E-05 | 0.005873 | UP |
| ENSG00000234139 | RP4-550H1.4 | 1.271635 | 2.68E-07 | 0.000228 | UP |
| ENSG00000234159 | RBPMSLP | 1.637188 | 1.25E-06 | 0.00055 | UP |
| ENSG00000234182 | RP11-118K6.2 | 1.650636 | 0.000177 | 0.009128 | UP |
| ENSG00000234270 | RPL36P20 | 1.175842 | 2.83E-05 | 0.00322 | UP |
| ENSG00000234273 | AC073071.1 | 1.458309 | 9.00E-08 | 0.000123 | UP |
| ENSG00000234293 | BACH1-IT3 | 1.69673 | 5.11E-05 | 0.004497 | UP |
| ENSG00000234379 | HMGB1P48 | 1.423282 | 1.82E-05 | 0.002505 | UP |
| ENSG00000234524 | RPL12P43 | 1.210316 | 0.000238 | 0.010761 | UP |
| ENSG00000234689 | LINC00444 | 1.288073 | 4.24E-09 | 2.17E-05 | UP |
| ENSG00000234737 | KRT18P15 | 2.834158 | 2.73E-07 | 0.00023 | UP |
| ENSG00000234742 | AC144530.1 | 1.184982 | 0.000737 | 0.020246 | UP |
| ENSG00000234743 | EIF5AP4 | 1.761872 | 3.62E-08 | 7.33E-05 | UP |
| ENSG00000234750 | CTD-2666L21.2 | 1.241239 | 0.00021 | 0.010056 | UP |
| ENSG00000234769 | WASH4P | 1.714249 | 0.000431 | 0.015025 | UP |
| ENSG00000234779 | RP11-62F24.2 | 1.001814 | 2.46E-08 | 5.90E-05 | UP |
| ENSG00000235111 | RP1-29C18.8 | 2.163209 | 4.15E-18 | 1.56E-10 | UP |
| ENSG00000235119 | RP11-9M16.2 | 1.372852 | 0.001767 | 0.032911 | UP |
| ENSG00000235217 | TSPY26P | 1.086917 | 0.000227 | 0.010503 | UP |
| ENSG00000235435 | AC064865.1 | 1.87565 | 2.68E-06 | 0.000842 | UP |
| ENSG00000235454 | HAUS6P3 | 1.417581 | 2.33E-05 | 0.002886 | UP |
| ENSG00000235499 | AC073046.25 | 1.522597 | 2.66E-07 | 0.000227 | UP |
| ENSG00000235579 | AC007283.4 | 1.154861 | 1.79E-07 | 0.000183 | UP |
| ENSG00000235615 | AJ239322.1 | 2.219447 | 6.66E-05 | 0.005228 | UP |
| ENSG00000235669 | AC004593.3 | 1.767431 | 9.22E-07 | 0.000461 | UP |
| ENSG00000235721 | AC013268.3 | 1.356355 | 1.38E-07 | 0.000157 | UP |
| ENSG00000235724 | AC009299.2 | 1.090781 | 0.000131 | 0.007671 | UP |
| ENSG00000235749 | RP11-634B7.4 | 1.348953 | 0.00013 | 0.007655 | UP |
| ENSG00000235785 | AL109767.1 | 1.572251 | 5.26E-07 | 0.000334 | UP |
| ENSG00000235827 | TUBB8P9 | 1.370547 | 0.002251 | 0.037665 | UP |
| ENSG00000235834 | RP1-60N8.1 | 2.639061 | 1.40E-08 | 4.26E-05 | UP |
| ENSG00000235947 | EGOT | 1.992366 | 2.01E-07 | 0.000195 | UP |
| ENSG00000236008 | AC011747.4 | 1.414517 | 1.54E-05 | 0.002284 | UP |
| ENSG00000236017 | ASMTL-AS1 | 1.798706 | 1.43E-05 | 0.002193 | UP |
| ENSG00000236018 | RP4-814D15.1 | 1.135186 | 0.002936 | 0.04373 | UP |
| ENSG00000236274 | RP4-728D4.3 | 1.98998 | 7.42E-05 | 0.005559 | UP |
| ENSG00000236277 | NIPA2P5 | 1.908758 | 2.06E-07 | 0.000198 | UP |
| ENSG00000236439 | RP11-175B9.3 | 1.726865 | 2.16E-07 | 0.000203 | UP |
| ENSG00000236449 | AC018890.6 | 1.129304 | 0.001481 | 0.029858 | UP |
| ENSG00000236564 | YWHAQP5 | 1.541002 | 2.38E-07 | 0.000214 | UP |
| ENSG00000236576 | RP11-22B10.3 | 1.303518 | 4.54E-07 | 0.000308 | UP |
| ENSG00000236756 | DNAJC9-AS1 | 1.066268 | 7.62E-07 | 0.000413 | UP |
| ENSG00000236779 | RP11-430C7.2 | 1.474196 | 1.53E-08 | 4.47E-05 | UP |
| ENSG00000236853 | OR2R1P | 1.481187 | 6.79E-05 | 0.005286 | UP |
| ENSG00000237017 | AC012314.8 | 1.29874 | 0.000693 | 0.019545 | UP |
| ENSG00000237065 | NANOGP4 | 1.148828 | 0.00075 | 0.02046 | UP |
| ENSG00000237111 | IGHJ3P | 1.381726 | 8.00E-06 | 0.001575 | UP |
| ENSG00000237186 | RP11-229A12.2 | 1.529769 | 0.0007 | 0.019652 | UP |
| ENSG00000237193 | RP11-275O4.4 | 1.363656 | 0.000405 | 0.014523 | UP |
| ENSG00000237399 | PITRM1-AS1 | 1.086431 | 0.0001 | 0.006609 | UP |
| ENSG00000237555 | RP5-862K6.4 | 1.487921 | 1.60E-07 | 0.000171 | UP |
| ENSG00000237716 | RP3-400B16.3 | 1.83804 | 2.88E-05 | 0.003256 | UP |
| ENSG00000237718 | AC009095.4 | 1.436284 | 7.90E-05 | 0.00577 | UP |
| ENSG00000237741 | AC002368.4 | 1.112365 | 8.41E-08 | 0.000119 | UP |
| ENSG00000237758 | BANF1P3 | 1.013726 | 0.001236 | 0.027008 | UP |
| ENSG00000237781 | RP11-54A4.2 | 1.944078 | 1.83E-10 | 3.66E-06 | UP |
| ENSG00000237892 | KLF7-IT1 | 1.012996 | 5.42E-05 | 0.004659 | UP |
| ENSG00000237929 | RPL31P3 | 1.056488 | 0.003571 | 0.048791 | UP |
| ENSG00000238094 | AC009892.5 | 1.122973 | 0.000981 | 0.02375 | UP |
| ENSG00000238109 | AC004893.10 | 1.337717 | 0.001922 | 0.034468 | UP |
| ENSG00000238160 | AC116366.5 | 1.052736 | 1.87E-06 | 0.000688 | UP |
| ENSG00000238609 | RNU7-94P | 1.363564 | 4.26E-06 | 0.001096 | UP |
| ENSG00000238697 | RNU6-261P | 1.53181 | 6.78E-09 | 2.82E-05 | UP |
| ENSG00000238959 | RNU7-19P | 1.004847 | 0.000345 | 0.01325 | UP |
| ENSG00000239043 | SNORD127 | 1.357036 | 0.000207 | 0.009967 | UP |
| ENSG00000239207 | GAPDHP39 | 1.628553 | 5.86E-05 | 0.004856 | UP |
| ENSG00000239415 | AP001469.9 | 1.163638 | 0.003132 | 0.045341 | UP |
| ENSG00000239470 | RP11-16F15.2 | 1.322497 | 0.001419 | 0.029181 | UP |
| ENSG00000239626 | RPSAP41 | 1.130509 | 0.001106 | 0.02543 | UP |
| ENSG00000239808 | RN7SL255P | 1.13457 | 2.51E-13 | 8.97E-08 | UP |
| ENSG00000239821 | RN7SL513P | 1.894821 | 4.09E-05 | 0.003963 | UP |
| ENSG00000239825 | RN7SL549P | 1.107299 | 0.000445 | 0.015296 | UP |
| ENSG00000240002 | YBX1P3 | 1.38443 | 0.000221 | 0.010316 | UP |
| ENSG00000240132 | ETF1P2 | 1.379428 | 9.17E-05 | 0.006296 | UP |
| ENSG00000240163 | RP11-745A24.1 | 1.656714 | 6.20E-05 | 0.005018 | UP |
| ENSG00000240219 | RP11-430C7.5 | 1.04751 | 0.002136 | 0.036536 | UP |
| ENSG00000240320 | AC006133.3 | 1.379189 | 5.35E-12 | 4.91E-07 | UP |
| ENSG00000240355 | AC004869.3 | 1.731865 | 3.59E-06 | 0.000998 | UP |
| ENSG00000240531 | RPL21P123 | 1.161594 | 0.002862 | 0.043096 | UP |
| ENSG00000240710 | RP11-430C7.4 | 1.433272 | 2.49E-06 | 0.000809 | UP |
| ENSG00000240793 | UBA52P8 | 2.393127 | 1.49E-06 | 0.000607 | UP |
| ENSG00000240853 | RN7SL328P | 2.848397 | 4.92E-09 | 2.34E-05 | UP |
| ENSG00000241015 | TPM3P9 | 1.465908 | 8.95E-06 | 0.001684 | UP |
| ENSG00000241269 | AC093620.5 | 1.491588 | 9.22E-05 | 0.006313 | UP |
| ENSG00000241429 | EEF1A1P25 | 1.746579 | 5.14E-06 | 0.00122 | UP |
| ENSG00000241438 | TDGF1P6 | 1.726836 | 0.000818 | 0.021482 | UP |
| ENSG00000241527 | CA15P1 | 1.166669 | 0.002791 | 0.042521 | UP |
| ENSG00000241546 | RP11-767L7.2 | 1.370647 | 0.002894 | 0.043368 | UP |
| ENSG00000241654 | RPL19P18 | 1.228007 | 5.45E-05 | 0.004672 | UP |
| ENSG00000241741 | RPL7AP30 | 2.419556 | 1.86E-06 | 0.000685 | UP |
| ENSG00000241890 | RPL13P4 | 1.485425 | 0.000951 | 0.023355 | UP |
| ENSG00000242048 | RP13-452N2.1 | 1.678486 | 5.72E-06 | 0.001296 | UP |
| ENSG00000242100 | RPL9P32 | 1.879701 | 3.17E-05 | 0.003429 | UP |
| ENSG00000242207 | HOXB-AS4 | 1.231375 | 1.32E-09 | 1.13E-05 | UP |
| ENSG00000242479 | RP11-383G6.4 | 1.136301 | 0.000792 | 0.021118 | UP |
| ENSG00000242588 | RP11-274B21.14 | 1.546183 | 1.15E-09 | 1.05E-05 | UP |
| ENSG00000242609 | RP11-398A8.1 | 2.816403 | 1.08E-07 | 0.000137 | UP |
| ENSG00000242687 | AC004893.11 | 1.396059 | 2.76E-12 | 3.35E-07 | UP |
| ENSG00000242729 | RP11-124G5.1 | 1.218503 | 0.000651 | 0.018885 | UP |
| ENSG00000242737 | RP11-562A8.1 | 1.546617 | 0.000719 | 0.019961 | UP |
| ENSG00000242853 | RN7SL749P | 1.347553 | 3.42E-05 | 0.003578 | UP |
| ENSG00000243049 | RN7SL33P | 1.357754 | 3.89E-06 | 0.00104 | UP |
| ENSG00000243179 | AC110769.3 | 1.107482 | 0.00333 | 0.046885 | UP |
| ENSG00000243313 | RN7SL285P | 1.944048 | 1.72E-05 | 0.002435 | UP |
| ENSG00000243388 | RPL3P3 | 1.517408 | 1.15E-06 | 0.000524 | UP |
| ENSG00000243398 | RN7SL141P | 1.219396 | 0.000263 | 0.011384 | UP |
| ENSG00000243508 | RP11-775J23.2 | 1.840505 | 0.00019 | 0.009499 | UP |
| ENSG00000243679 | RP11-274B21.3 | 1.60553 | 1.06E-07 | 0.000135 | UP |
| ENSG00000243914 | RPL5P14 | 1.173237 | 2.07E-06 | 0.000728 | UP |
| ENSG00000243964 | RPL23AP65 | 1.407255 | 5.73E-05 | 0.004794 | UP |
| ENSG00000244044 | RN7SL735P | 2.370964 | 2.15E-05 | 0.002757 | UP |
| ENSG00000244237 | RP11-1046B16.1 | 1.527085 | 0.000131 | 0.007687 | UP |
| ENSG00000244267 | RPL34P22 | 1.270568 | 7.04E-05 | 0.005394 | UP |
| ENSG00000244297 | RN7SL465P | 1.529803 | 1.12E-14 | 1.54E-08 | UP |
| ENSG00000273838 | RP3-333B15.5 | 1.147918 | 6.71E-05 | 0.005248 | UP |
| ENSG00000244556 | ODCP | 1.334618 | 0.000134 | 0.007777 | UP |
| ENSG00000244604 | RP11-713H12.1 | 1.042353 | 7.06E-06 | 0.001464 | UP |
| ENSG00000244625 | MIATNB | 1.008533 | 1.23E-06 | 0.000543 | UP |
| ENSG00000244642 | RN7SL396P | 3.079939 | 2.92E-08 | 6.48E-05 | UP |
| ENSG00000244674 | RPS3AP15 | 1.696898 | 8.33E-06 | 0.001615 | UP |
| ENSG00000244676 | AL109761.5 | 1.055971 | 4.54E-11 | 1.63E-06 | UP |
| ENSG00000244691 | RPL10AP1 | 1.358285 | 0.000483 | 0.01602 | UP |
| ENSG00000245522 | RP11-540A21.2 | 1.654831 | 0.001468 | 0.029714 | UP |
| ENSG00000245556 | SCAMP1-AS1 | 1.126207 | 0.00069 | 0.019508 | UP |
| ENSG00000246548 | RP11-7F17.5 | 1.766336 | 1.43E-07 | 0.00016 | UP |
| ENSG00000246575 | AC093162.5 | 1.257346 | 1.59E-07 | 0.00017 | UP |
| ENSG00000247151 | CSTF3-AS1 | 1.51369 | 8.98E-09 | 3.28E-05 | UP |
| ENSG00000247828 | TMEM161B-AS1 | 2.800798 | 8.52E-09 | 3.20E-05 | UP |
| ENSG00000247925 | RP3-510L9.1 | 1.244867 | 4.17E-05 | 0.004 | UP |
| ENSG00000248112 | RP11-78C3.1 | 1.418328 | 9.82E-08 | 0.00013 | UP |
| ENSG00000248180 | GAPDHP60 | 1.415393 | 1.07E-05 | 0.001869 | UP |
| ENSG00000248242 | RP11-556I14.2 | 1.486879 | 3.70E-06 | 0.001014 | UP |
| ENSG00000248283 | CCNL2P1 | 1.13007 | 0.001062 | 0.024845 | UP |
| ENSG00000248311 | CTD-2161F6.2 | 1.018145 | 1.31E-05 | 0.002087 | UP |
| ENSG00000248318 | RP11-713M15.1 | 1.511579 | 2.11E-08 | 5.41E-05 | UP |
| ENSG00000248492 | ZFAT-AS1 | 1.153958 | 2.40E-05 | 0.002931 | UP |
| ENSG00000248544 | CTB-47B11.3 | 1.039358 | 2.12E-05 | 0.002731 | UP |
| ENSG00000248554 | RP11-159F24.6 | 1.78085 | 0.000304 | 0.012336 | UP |
| ENSG00000248618 | ENPP7P3 | 1.260205 | 2.04E-06 | 0.000723 | UP |
| ENSG00000248673 | LINC01331 | 1.226869 | 2.84E-08 | 6.37E-05 | UP |
| ENSG00000248697 | TOX4P1 | 1.497022 | 3.89E-05 | 0.003836 | UP |
| ENSG00000248795 | RP11-173E2.1 | 1.24301 | 9.15E-07 | 0.000459 | UP |
| ENSG00000249099 | RP11-351N6.1 | 1.425431 | 2.07E-06 | 0.000729 | UP |
| ENSG00000249160 | RP11-1C1.5 | 2.111605 | 1.33E-05 | 0.002101 | UP |
| ENSG00000249175 | CTC-484P3.3 | 1.024789 | 0.000667 | 0.019136 | UP |
| ENSG00000249270 | KATNBL1P4 | 1.755354 | 3.33E-08 | 7.02E-05 | UP |
| ENSG00000249396 | RP11-1C1.4 | 1.59837 | 4.78E-05 | 0.004324 | UP |
| ENSG00000249820 | RP11-503I22.2 | 1.349734 | 3.21E-07 | 0.000253 | UP |
| ENSG00000249985 | RP11-468N14.8 | 1.713579 | 1.84E-15 | 5.48E-09 | UP |
| ENSG00000249988 | RP11-669M16.1 | 1.248268 | 8.08E-11 | 2.28E-06 | UP |
| ENSG00000250068 | RP11-576C12.1 | 1.505682 | 0.001241 | 0.027064 | UP |
| ENSG00000250223 | LINC01216 | 1.212062 | 2.23E-05 | 0.002812 | UP |
| ENSG00000250358 | RP11-159K7.2 | 1.069286 | 1.60E-06 | 0.000632 | UP |
| ENSG00000250362 | AC008592.5 | 2.115161 | 0.000225 | 0.010442 | UP |
| ENSG00000250504 | KRT18P51 | 1.192441 | 0.00246 | 0.039627 | UP |
| ENSG00000250539 | KRT8P33 | 1.256197 | 1.38E-06 | 0.00058 | UP |
| ENSG00000250574 | CTD-2275D10.2 | 1.992226 | 7.73E-07 | 0.000416 | UP |
| ENSG00000250604 | RP11-597D13.8 | 1.807938 | 1.72E-05 | 0.002436 | UP |
| ENSG00000250715 | RP11-432M8.19 | 1.107585 | 6.42E-06 | 0.001387 | UP |
| ENSG00000250733 | C8orf17 | 1.501016 | 0.000209 | 0.010029 | UP |
| ENSG00000251099 | CTD-2367A17.1 | 1.217604 | 1.01E-07 | 0.000132 | UP |
| ENSG00000251101 | RP11-1267H10.1 | 1.149494 | 0.000526 | 0.016811 | UP |
| ENSG00000251132 | RP11-438C19.2 | 1.92854 | 0.000148 | 0.008223 | UP |
| ENSG00000251323 | RP11-452H21.4 | 1.851537 | 6.52E-05 | 0.005168 | UP |
| ENSG00000251378 | AC096582.9 | 1.878365 | 1.14E-09 | 1.04E-05 | UP |
| ENSG00000251429 | RP11-597D13.7 | 1.630293 | 0.000138 | 0.007892 | UP |
| ENSG00000251441 | RTEL1P1 | 1.407609 | 4.49E-08 | 8.23E-05 | UP |
| ENSG00000251497 | RP11-197N18.7 | 1.382699 | 0.000191 | 0.009525 | UP |
| ENSG00000251667 | BRCC3P1 | 1.515844 | 0.000537 | 0.017022 | UP |
| ENSG00000251685 | UGT2B27P | 1.142941 | 0.002112 | 0.036301 | UP |
| ENSG00000251711 | RNU6-632P | 1.636782 | 1.56E-12 | 2.40E-07 | UP |
| ENSG00000251761 | RNU6-138P | 2.459482 | 3.24E-07 | 0.000254 | UP |
| ENSG00000251831 | RNU6-1114P | 2.434557 | 2.27E-06 | 0.000767 | UP |
| ENSG00000252008 | RNU6-927P | 1.272888 | 0.000327 | 0.012873 | UP |
| ENSG00000252013 | RNU4ATAC14P | 1.04521 | 4.99E-07 | 0.000325 | UP |
| ENSG00000252032 | RNU6-1281P | 2.178877 | 6.33E-14 | 4.21E-08 | UP |
| ENSG00000252151 | RNU6-1265P | 1.811162 | 2.99E-05 | 0.003321 | UP |
| ENSG00000252503 | RNU6-531P | 1.219249 | 1.90E-05 | 0.002567 | UP |
| ENSG00000252515 | RNU6-1171P | 1.008834 | 6.14E-05 | 0.004992 | UP |
| ENSG00000252772 | RNU6-714P | 1.340236 | 1.17E-05 | 0.001955 | UP |
| ENSG00000252782 | RNU6-341P | 1.195358 | 0.000144 | 0.008118 | UP |
| ENSG00000253269 | CTB-37A13.1 | 1.142972 | 0.000179 | 0.009191 | UP |
| ENSG00000253352 | TUG1 | 1.146217 | 1.85E-06 | 0.000684 | UP |
| ENSG00000253522 | MIR3142HG | 1.17483 | 0.001557 | 0.030719 | UP |
| ENSG00000253641 | LINCR-0001 | 1.459636 | 0.001389 | 0.028835 | UP |
| ENSG00000253649 | PRSS51 | 1.751114 | 1.17E-10 | 2.81E-06 | UP |
| ENSG00000253676 | TAGLN2P1 | 1.072235 | 9.61E-05 | 0.006465 | UP |
| ENSG00000253739 | RNU6-323P | 1.677254 | 0.000634 | 0.018631 | UP |
| ENSG00000253741 | CTD-2292P10.4 | 1.115207 | 0.000406 | 0.014534 | UP |
| ENSG00000254019 | RP11-10J21.3 | 2.525174 | 1.98E-08 | 5.22E-05 | UP |
| ENSG00000254020 | RP11-100L22.4 | 1.168446 | 2.42E-06 | 0.000796 | UP |
| ENSG00000254037 | RP11-775B15.3 | 1.596978 | 1.14E-05 | 0.001932 | UP |
| ENSG00000254153 | CTA-398F10.2 | 1.224829 | 0.003598 | 0.049011 | UP |
| ENSG00000254231 | CTD-2284J15.1 | 1.12942 | 0.002559 | 0.040484 | UP |
| ENSG00000254286 | RP11-89K10.1 | 1.839896 | 3.88E-06 | 0.001038 | UP |
| ENSG00000254425 | RP11-480O10.2 | 2.332732 | 3.35E-05 | 0.003535 | UP |
| ENSG00000254464 | OR4A3P | 2.270116 | 2.97E-05 | 0.003309 | UP |
| ENSG00000254644 | RP11-5A11.2 | 1.45794 | 0.00099 | 0.023845 | UP |
| ENSG00000254649 | RP11-452H21.2 | 1.269611 | 5.53E-05 | 0.004707 | UP |
| ENSG00000254670 | CTC-497E21.3 | 2.045378 | 3.76E-16 | 2.14E-09 | UP |
| ENSG00000254689 | RP11-354A14.1 | 1.034664 | 3.15E-05 | 0.003416 | UP |
| ENSG00000254704 | RP11-1036E20.7 | 1.026281 | 9.00E-06 | 0.00169 | UP |
| ENSG00000254713 | HNRNPA1P72 | 2.033926 | 5.45E-08 | 9.23E-05 | UP |
| ENSG00000254720 | RP11-583F24.3 | 2.03465 | 5.65E-05 | 0.004759 | UP |
| ENSG00000254731 | CTD-2005H7.1 | 1.924915 | 6.79E-10 | 7.79E-06 | UP |
| ENSG00000254825 | OR9G2P | 2.000088 | 7.59E-07 | 0.000412 | UP |
| ENSG00000254856 | NDUFA3P2 | 1.261043 | 0.001 | 0.023981 | UP |
| ENSG00000254907 | RP11-484D2.2 | 1.331647 | 0.001392 | 0.028862 | UP |
| ENSG00000254953 | RP11-100N3.2 | 2.128149 | 8.69E-07 | 0.000445 | UP |
| ENSG00000255055 | MTND1P35 | 3.133415 | 8.20E-09 | 3.13E-05 | UP |
| ENSG00000255158 | RP11-754B17.1 | 1.966064 | 4.25E-05 | 0.004044 | UP |
| ENSG00000255289 | RP11-91I20.1 | 1.259323 | 4.50E-06 | 0.001132 | UP |
| ENSG00000255585 | RP11-188C12.2 | 1.437255 | 2.99E-09 | 1.80E-05 | UP |
| ENSG00000255933 | RP11-495K9.5 | 1.457021 | 1.78E-05 | 0.002479 | UP |
| ENSG00000256159 | RP11-820K3.4 | 1.263409 | 0.000313 | 0.012556 | UP |
| ENSG00000256218 | RP11-1038A11.2 | 1.063478 | 1.01E-05 | 0.001805 | UP |
| ENSG00000256314 | RP11-338E21.3 | 1.301805 | 0.000108 | 0.006906 | UP |
| ENSG00000256331 | NIFKP3 | 1.369654 | 0.001133 | 0.025752 | UP |
| ENSG00000256465 | RP11-428G5.6 | 2.259818 | 0.000119 | 0.00729 | UP |
| ENSG00000256603 | RP11-667M19.2 | 1.693678 | 2.29E-10 | 4.12E-06 | UP |
| ENSG00000256746 | RP11-17G12.3 | 1.081111 | 0.003326 | 0.046851 | UP |
| ENSG00000256806 | C17orf100 | 1.083553 | 0.003186 | 0.045763 | UP |
| ENSG00000256925 | ADGRA1-AS1 | 1.885119 | 7.74E-08 | 0.000113 | UP |
| ENSG00000257069 | RP11-783K16.10 | 1.569257 | 4.34E-06 | 0.001109 | UP |
| ENSG00000257142 | RP11-536C10.19 | 1.189449 | 0.003458 | 0.047951 | UP |
| ENSG00000257183 | RP11-274M17.3 | 1.138273 | 0.000105 | 0.006779 | UP |
| ENSG00000257210 | NACAP3 | 1.059104 | 3.13E-06 | 0.000922 | UP |
| ENSG00000257220 | RP11-136F16.2 | 1.304564 | 2.34E-05 | 0.002892 | UP |
| ENSG00000257246 | PHBP19 | 1.460731 | 0.000121 | 0.00733 | UP |
| ENSG00000257294 | RP11-316A16.1 | 1.031691 | 3.27E-05 | 0.003493 | UP |
| ENSG00000280228 | RP11-67L14.1 | 1.048738 | 1.30E-07 | 0.000152 | UP |
| ENSG00000257893 | RP11-587P21.2 | 1.079944 | 1.08E-10 | 2.69E-06 | UP |
| ENSG00000257910 | RP11-461F16.3 | 1.038653 | 2.05E-05 | 0.002686 | UP |
| ENSG00000258439 | RP11-173A8.2 | 1.205005 | 0.000107 | 0.006871 | UP |
| ENSG00000258485 | SRMP2 | 1.608882 | 2.63E-09 | 1.68E-05 | UP |
| ENSG00000258494 | OR11J5P | 1.29816 | 0.001484 | 0.029882 | UP |
| ENSG00000258500 | CTD-2062F14.2 | 1.396869 | 5.82E-09 | 2.57E-05 | UP |
| ENSG00000258545 | RHOXF1-AS1 | 1.516277 | 0.001106 | 0.025432 | UP |
| ENSG00000258564 | OR4N1P | 1.469411 | 0.001032 | 0.02444 | UP |
| ENSG00000258591 | RP11-545M17.3 | 2.002017 | 2.18E-08 | 5.51E-05 | UP |
| ENSG00000258620 | RP11-362L22.1 | 2.156051 | 9.34E-05 | 0.006354 | UP |
| ENSG00000258730 | ITPK1-AS1 | 1.421066 | 5.79E-11 | 1.87E-06 | UP |
| ENSG00000258741 | RP11-386M24.4 | 1.462501 | 0.000287 | 0.011957 | UP |
| ENSG00000258781 | RP11-496I2.4 | 1.262828 | 0.001673 | 0.031932 | UP |
| ENSG00000258847 | CTD-2014B16.3 | 1.533776 | 3.03E-06 | 0.000905 | UP |
| ENSG00000258926 | RP11-47I22.1 | 1.053597 | 0.0019 | 0.034275 | UP |
| ENSG00000259079 | RP1-261D10.1 | 1.409054 | 0.000241 | 0.01085 | UP |
| ENSG00000259149 | RP11-313C4.1 | 1.699853 | 0.000312 | 0.012515 | UP |
| ENSG00000259202 | RP11-342M21.2 | 1.970541 | 7.77E-05 | 0.00571 | UP |
| ENSG00000259276 | RP11-815J21.3 | 1.522846 | 4.18E-05 | 0.004007 | UP |
| ENSG00000259294 | CTA-339C12.1 | 1.614798 | 1.22E-06 | 0.000541 | UP |
| ENSG00000259468 | RP11-1084A12.2 | 1.367614 | 0.000274 | 0.011634 | UP |
| ENSG00000259475 | RP11-654A16.3 | 1.354107 | 1.03E-05 | 0.001824 | UP |
| ENSG00000259688 | RP11-139F4.2 | 1.762658 | 9.58E-06 | 0.001751 | UP |
| ENSG00000259852 | IGHV1OR16-2 | 1.326011 | 0.001546 | 0.030605 | UP |
| ENSG00000259856 | RAB43P1 | 1.203926 | 0.000405 | 0.014516 | UP |
| ENSG00000259881 | RP11-830F9.5 | 1.566465 | 3.28E-05 | 0.003497 | UP |
| ENSG00000259891 | CTA-204B4.2 | 1.061408 | 0.001081 | 0.025108 | UP |
| ENSG00000259950 | RP11-170L3.4 | 1.325427 | 4.22E-09 | 2.17E-05 | UP |
| ENSG00000259962 | RP11-44F14.4 | 1.778809 | 4.16E-06 | 0.00108 | UP |
| ENSG00000260034 | LCMT1-AS2 | 1.060883 | 1.73E-05 | 0.002439 | UP |
| ENSG00000260103 | RP11-10O17.1 | 1.071406 | 0.000687 | 0.019449 | UP |
| ENSG00000260229 | RP11-391L3.5 | 2.2421 | 4.48E-12 | 4.41E-07 | UP |
| ENSG00000260252 | RP11-384M15.3 | 2.51904 | 7.64E-09 | 3.01E-05 | UP |
| ENSG00000260378 | CTD-2583P5.1 | 1.716421 | 2.96E-09 | 1.79E-05 | UP |
| ENSG00000260402 | RP11-56L13.1 | 1.112515 | 1.05E-06 | 0.000496 | UP |
| ENSG00000260427 | AGGF1P9 | 1.326075 | 2.81E-09 | 1.74E-05 | UP |
| ENSG00000260549 | MT1L | 1.363002 | 0.000996 | 0.023926 | UP |
| ENSG00000260600 | RP11-109D24.1 | 1.201566 | 2.20E-07 | 0.000205 | UP |
| ENSG00000260653 | RP11-114G11.5 | 1.11742 | 0.00261 | 0.040908 | UP |
| ENSG00000260807 | RP11-161M6.2 | 1.364122 | 7.26E-05 | 0.005491 | UP |
| ENSG00000260812 | RARRES2P7 | 1.038047 | 6.02E-05 | 0.00493 | UP |
| ENSG00000260908 | CTB-134H23.3 | 2.781726 | 2.63E-07 | 0.000226 | UP |
| ENSG00000260929 | RP11-327F22.1 | 1.29437 | 0.000602 | 0.018127 | UP |
| ENSG00000261064 | RP11-1000B6.3 | 1.607427 | 0.000545 | 0.017159 | UP |
| ENSG00000261090 | RP11-20G6.2 | 1.65419 | 5.61E-07 | 0.000346 | UP |
| ENSG00000261096 | RP11-690I21.2 | 1.858659 | 0.000277 | 0.01171 | UP |
| ENSG00000261183 | RP11-532F12.5 | 1.031319 | 2.10E-06 | 0.000732 | UP |
| ENSG00000261207 | LA16c-361A3.3 | 1.762124 | 4.06E-06 | 0.001066 | UP |
| ENSG00000261216 | RP11-166B2.5 | 1.554588 | 0.00122 | 0.026826 | UP |
| ENSG00000261222 | CTD-2006K23.1 | 1.204824 | 0.000108 | 0.006895 | UP |
| ENSG00000261226 | RP11-830F9.7 | 1.471967 | 3.93E-05 | 0.00386 | UP |
| ENSG00000261245 | RP11-120K18.3 | 1.925313 | 7.79E-08 | 0.000113 | UP |
| ENSG00000261294 | RP11-616M22.3 | 1.682259 | 6.26E-12 | 5.32E-07 | UP |
| ENSG00000261397 | LINC01177 | 1.377399 | 1.28E-05 | 0.002059 | UP |
| ENSG00000261419 | RP11-57A19.4 | 1.223373 | 4.61E-05 | 0.004229 | UP |
| ENSG00000261451 | RP11-981G7.1 | 1.161862 | 0.002937 | 0.043731 | UP |
| ENSG00000261471 | RP11-61F12.1 | 1.197795 | 1.21E-11 | 7.59E-07 | UP |
| ENSG00000261574 | RP1-168P16.2 | 1.348478 | 0.000197 | 0.009685 | UP |
| ENSG00000261811 | RP11-382N13.2 | 1.255798 | 0.000338 | 0.0131 | UP |
| ENSG00000261971 | MMP25-AS1 | 1.064699 | 4.64E-09 | 2.28E-05 | UP |
| ENSG00000262580 | RP11-334C17.5 | 2.122467 | 2.30E-08 | 5.68E-05 | UP |
| ENSG00000262668 | AJ003147.9 | 1.27288 | 0.002681 | 0.041542 | UP |
| ENSG00000262833 | RP11-28G8.1 | 1.370689 | 2.28E-05 | 0.002851 | UP |
| ENSG00000262884 | CTD-3060P21.1 | 1.095606 | 0.0004 | 0.014412 | UP |
| ENSG00000262905 | RP5-1029F21.2 | 1.784738 | 0.000103 | 0.006725 | UP |
| ENSG00000262943 | ALOX12P2 | 1.320434 | 9.06E-05 | 0.006251 | UP |
| ENSG00000263331 | CTC-508F8.1 | 1.573434 | 1.85E-06 | 0.000685 | UP |
| ENSG00000263368 | RP11-720N19.1 | 2.00589 | 0.000191 | 0.009541 | UP |
| ENSG00000263622 | RP11-389J22.3 | 1.474259 | 0.000808 | 0.021339 | UP |
| ENSG00000263657 | RP11-82O19.1 | 1.347943 | 1.24E-07 | 0.000148 | UP |
| ENSG00000263729 | RP11-746M1.8 | 1.638896 | 2.99E-06 | 0.000898 | UP |
| ENSG00000263883 | EEF1DP7 | 1.0266 | 0.000521 | 0.016709 | UP |
| ENSG00000264017 | RN7SL336P | 1.290622 | 0.002184 | 0.037026 | UP |
| ENSG00000264031 | ABHD15-AS1 | 1.900532 | 3.44E-06 | 0.000973 | UP |
| ENSG00000264270 | RP11-474I11.7 | 1.315514 | 0.000965 | 0.023528 | UP |
| ENSG00000264400 | RN7SL491P | 1.107993 | 0.000518 | 0.016653 | UP |
| ENSG00000264840 | RN7SL404P | 1.329365 | 3.78E-05 | 0.003787 | UP |
| ENSG00000264907 | PRELID3BP3 | 1.50664 | 6.24E-09 | 2.69E-05 | UP |
| ENSG00000264924 | RP11-799B12.2 | 2.055663 | 3.28E-06 | 0.000948 | UP |
| ENSG00000264943 | SH3GL1P2 | 1.4962 | 0.000858 | 0.02206 | UP |
| ENSG00000265114 | RP11-285M22.1 | 1.370996 | 5.16E-05 | 0.004524 | UP |
| ENSG00000265261 | RP11-162A12.3 | 1.312908 | 0.000782 | 0.020946 | UP |
| ENSG00000265469 | POLR3KP2 | 1.279607 | 5.51E-06 | 0.001269 | UP |
| ENSG00000266222 | RP11-433M22.1 | 1.561929 | 0.001933 | 0.034571 | UP |
| ENSG00000266237 | RP11-25D3.1 | 2.019223 | 2.19E-10 | 4.02E-06 | UP |
| ENSG00000266439 | RN7SL493P | 1.096013 | 6.52E-09 | 2.76E-05 | UP |
| ENSG00000266897 | AC005546.2 | 1.846458 | 2.61E-08 | 6.08E-05 | UP |
| ENSG00000266943 | RPSAP66 | 1.49658 | 0.000694 | 0.019567 | UP |
| ENSG00000266946 | MRPL37P1 | 1.339801 | 0.000162 | 0.008673 | UP |
| ENSG00000266965 | RP11-712P20.2 | 1.073828 | 0.000356 | 0.013498 | UP |
| ENSG00000266998 | RP11-936I5.1 | 1.129428 | 7.58E-06 | 0.001526 | UP |
| ENSG00000267016 | RP11-75C10.9 | 2.056083 | 5.86E-06 | 0.001315 | UP |
| ENSG00000267109 | CTD-2378E21.1 | 1.467783 | 6.63E-09 | 2.79E-05 | UP |
| ENSG00000267121 | CTD-2020K17.1 | 1.031959 | 4.74E-07 | 0.000316 | UP |
| ENSG00000267136 | RP11-53B2.3 | 1.424349 | 6.31E-08 | 0.0001 | UP |
| ENSG00000267141 | CTB-31O20.8 | 1.347178 | 1.48E-05 | 0.002238 | UP |
| ENSG00000267170 | CALM2P1 | 1.683782 | 0.001235 | 0.026997 | UP |
| ENSG00000267244 | CTB-31O20.4 | 1.257889 | 0.000457 | 0.015517 | UP |
| ENSG00000267322 | SNHG22 | 2.300978 | 1.15E-07 | 0.000141 | UP |
| ENSG00000267344 | CTB-39G8.3 | 1.704443 | 7.17E-08 | 0.000108 | UP |
| ENSG00000267369 | RP11-1094M14.8 | 1.031092 | 0.000458 | 0.015535 | UP |
| ENSG00000267405 | CTC-296K1.4 | 1.05524 | 0.001525 | 0.030349 | UP |
| ENSG00000267420 | RP11-527L4.6 | 1.449026 | 0.000328 | 0.012899 | UP |
| ENSG00000267454 | ZNF582-AS1 | 1.241044 | 4.18E-11 | 1.55E-06 | UP |
| ENSG00000267603 | LINC01028 | 1.406834 | 3.82E-06 | 0.00103 | UP |
| ENSG00000267632 | RP11-400F19.18 | 1.287276 | 6.79E-06 | 0.001433 | UP |
| ENSG00000267682 | CTD-3220F14.2 | 1.114903 | 0.001846 | 0.03373 | UP |
| ENSG00000267694 | RP11-691H4.4 | 1.374067 | 5.02E-07 | 0.000326 | UP |
| ENSG00000267695 | RP11-1030E3.1 | 1.099381 | 1.08E-05 | 0.001874 | UP |
| ENSG00000267702 | RP11-53B2.2 | 1.584865 | 2.05E-16 | 1.46E-09 | UP |
| ENSG00000267838 | AC008746.12 | 1.300018 | 0.0011 | 0.025352 | UP |
| ENSG00000267882 | RP4-569M23.5 | 1.17076 | 0.001818 | 0.033452 | UP |
| ENSG00000268119 | CTD-2561J22.5 | 1.074366 | 8.92E-07 | 0.000452 | UP |
| ENSG00000268218 | AC137932.4 | 1.043997 | 2.52E-05 | 0.003014 | UP |
| ENSG00000268322 | BNIP3P25 | 1.061801 | 2.30E-07 | 0.00021 | UP |
| ENSG00000268499 | CTB-102L5.8 | 1.339895 | 0.001276 | 0.027468 | UP |
| ENSG00000268535 | RP11-420K14.3 | 1.676535 | 5.08E-07 | 0.000328 | UP |
| ENSG00000268605 | CTB-50E14.4 | 1.253556 | 1.73E-05 | 0.00244 | UP |
| ENSG00000268659 | RP11-310J24.3 | 1.884144 | 6.82E-05 | 0.005299 | UP |
| ENSG00000268894 | PLCE1-AS1 | 2.316907 | 5.23E-06 | 0.001232 | UP |
| ENSG00000269256 | RP11-325D15.2 | 1.836884 | 9.67E-07 | 0.000473 | UP |
| ENSG00000269825 | CTD-3099C6.9 | 1.865874 | 2.12E-07 | 0.000201 | UP |
| ENSG00000269983 | RP11-497H16.9 | 1.489043 | 1.31E-05 | 0.002086 | UP |
| ENSG00000269985 | RP1-232P20.1 | 1.264329 | 0.000873 | 0.022258 | UP |
| ENSG00000270015 | RP11-540B6.6 | 1.142439 | 0.000244 | 0.010904 | UP |
| ENSG00000270072 | RP11-750H9.7 | 2.648407 | 4.78E-07 | 0.000317 | UP |
| ENSG00000270084 | GAS5-AS1 | 1.307112 | 1.01E-10 | 2.58E-06 | UP |
| ENSG00000270185 | IGHD1OR15-1B | 1.043624 | 0.003531 | 0.048507 | UP |
| ENSG00000270231 | NBPF8 | 1.068111 | 0.001382 | 0.028755 | UP |
| ENSG00000270252 | IGKV3OR2-5 | 1.545626 | 0.000203 | 0.009875 | UP |
| ENSG00000270381 | RP11-290D2.5 | 1.302315 | 1.01E-07 | 0.000132 | UP |
| ENSG00000270401 | RP11-812E19.14 | 1.35985 | 0.001972 | 0.034967 | UP |
| ENSG00000270462 | RP11-342K6.3 | 1.027593 | 0.000236 | 0.010722 | UP |
| ENSG00000270532 | PEBP1P2 | 1.312775 | 0.003136 | 0.045369 | UP |
| ENSG00000270702 | RP11-107F6.4 | 1.230541 | 0.000676 | 0.019275 | UP |
| ENSG00000270714 | MINOS1P2 | 1.570132 | 0.0002 | 0.009767 | UP |
| ENSG00000270832 | RP1-168P16.3 | 1.736671 | 0.000824 | 0.021563 | UP |
| ENSG00000270846 | RP11-674I16.1 | 1.523756 | 0.000516 | 0.016626 | UP |
| ENSG00000270850 | RP11-395N6.4 | 1.160996 | 7.72E-08 | 0.000113 | UP |
| ENSG00000270889 | RP11-609L3.1 | 1.189707 | 4.99E-06 | 0.001199 | UP |
| ENSG00000270894 | AC015849.13 | 1.793122 | 0.000199 | 0.009747 | UP |
| ENSG00000270954 | RPSAP75 | 1.537465 | 7.64E-06 | 0.001533 | UP |
| ENSG00000270983 | RP11-137J7.3 | 1.764969 | 1.22E-06 | 0.000542 | UP |
| ENSG00000271151 | RP11-394I13.2 | 1.108942 | 0.000203 | 0.009875 | UP |
| ENSG00000271180 | RP11-665C16.8 | 1.546479 | 0.000638 | 0.018695 | UP |
| ENSG00000271204 | RP11-138A9.1 | 1.066312 | 0.001775 | 0.032975 | UP |
| ENSG00000271349 | RP11-44N17.2 | 1.024372 | 0.00015 | 0.008296 | UP |
| ENSG00000271443 | RP11-541E12.1 | 1.513959 | 7.78E-09 | 3.04E-05 | UP |
| ENSG00000271454 | RP11-290L7.5 | 1.663722 | 0.001109 | 0.025463 | UP |
| ENSG00000271488 | RBM11P1 | 1.016555 | 3.34E-06 | 0.000957 | UP |
| ENSG00000271626 | RP11-3K24.2 | 1.456291 | 0.001288 | 0.027614 | UP |
| ENSG00000271997 | RP11-97O12.6 | 1.205306 | 0.000187 | 0.009425 | UP |
| ENSG00000272055 | RNU6-6P | 1.126827 | 4.24E-08 | 7.98E-05 | UP |
| ENSG00000272148 | RP11-195B17.1 | 1.707464 | 0.000498 | 0.01628 | UP |
| ENSG00000272150 | NBPF25P | 1.427773 | 0.00195 | 0.034731 | UP |
| ENSG00000272156 | RP11-477N3.1 | 1.338611 | 5.01E-08 | 8.78E-05 | UP |
| ENSG00000272195 | RP11-156E8.1 | 1.544507 | 0.001217 | 0.026795 | UP |
| ENSG00000272219 | CTB-181H17.1 | 1.424429 | 0.000133 | 0.00774 | UP |
| ENSG00000272505 | RP11-981G7.6 | 2.03464 | 0.000228 | 0.01051 | UP |
| ENSG00000272588 | RP11-440L14.4 | 1.263152 | 6.80E-10 | 7.79E-06 | UP |
| ENSG00000272754 | AL133245.2 | 1.170472 | 0.000411 | 0.014624 | UP |
| ENSG00000272767 | JMJD1C-AS1 | 2.046147 | 8.79E-08 | 0.000122 | UP |
| ENSG00000272797 | RP11-368I23.3 | 1.04577 | 6.20E-06 | 0.001358 | UP |
| ENSG00000272807 | RP11-260M2.1 | 1.228164 | 0.000639 | 0.018697 | UP |
| ENSG00000272814 | RP11-15I20.1 | 1.375851 | 0.000109 | 0.006936 | UP |
| ENSG00000272995 | RP11-362J17.1 | 1.127642 | 0.000413 | 0.014673 | UP |
| ENSG00000273006 | RP11-314C9.2 | 1.717284 | 2.18E-09 | 1.51E-05 | UP |
| ENSG00000273007 | RP11-170N16.3 | 2.613993 | 2.33E-07 | 0.000212 | UP |
| ENSG00000273153 | RP11-406H21.2 | 1.362196 | 1.36E-08 | 4.19E-05 | UP |
| ENSG00000273253 | RP3-402G11.26 | 1.826353 | 8.89E-18 | 2.47E-10 | UP |
| ENSG00000273284 | RP11-888D10.4 | 1.635296 | 9.29E-06 | 0.001721 | UP |
| ENSG00000273368 | RP11-376P6.3 | 1.542063 | 5.13E-07 | 0.00033 | UP |
| ENSG00000273520 | SPDYE8P | 1.389341 | 0.001226 | 0.026901 | UP |
| ENSG00000273537 | ZEB2_AS1_3 | 1.452754 | 0.001902 | 0.034283 | UP |
| ENSG00000273650 | CTD-3193K9.11 | 1.449872 | 0.002986 | 0.044118 | UP |
| ENSG00000273721 | RP11-745A24.3 | 1.737286 | 9.02E-07 | 0.000455 | UP |
| ENSG00000273729 | RP11-7F17.8 | 1.192943 | 2.67E-05 | 0.003114 | UP |
| ENSG00000273759 | RP4-563E14.1 | 1.008816 | 0.001432 | 0.029318 | UP |
| ENSG00000273851 | RP11-809H16.3 | 1.039815 | 0.000569 | 0.017581 | UP |
| ENSG00000273890 | RP11-603J24.21 | 1.15648 | 7.91E-08 | 0.000114 | UP |
| ENSG00000273908 | WT1-AS_7 | 1.19771 | 3.14E-06 | 0.000924 | UP |
| ENSG00000274028 | RP11-440D17.5 | 1.772599 | 4.01E-06 | 0.001058 | UP |
| ENSG00000274256 | RP1-229K20.8 | 1.443809 | 0.000103 | 0.006694 | UP |
| ENSG00000274272 | RP11-44M6.7 | 1.506314 | 0.000681 | 0.019349 | UP |
| ENSG00000274328 | RP11-239C9.1 | 1.493217 | 0.002026 | 0.035482 | UP |
| ENSG00000274591 | RP11-474P2.5 | 1.911108 | 0.000265 | 0.011416 | UP |
| ENSG00000274677 | RP11-505K9.5 | 1.971776 | 1.73E-05 | 0.002439 | UP |
| ENSG00000274864 | HOTAIRM1_3 | 2.420326 | 2.37E-10 | 4.20E-06 | UP |
| ENSG00000274963 | RN7SL600P | 1.207256 | 1.93E-08 | 5.13E-05 | UP |
| ENSG00000274995 | RP11-321F6.2 | 1.620111 | 0.000126 | 0.007509 | UP |
| ENSG00000275011 | RP11-599B13.9 | 1.027571 | 2.56E-05 | 0.003042 | UP |
| ENSG00000275178 | RP11-4B16.3 | 1.315719 | 5.91E-08 | 9.66E-05 | UP |
| ENSG00000275345 | RP11-697E2.11 | 1.286748 | 0.000168 | 0.008859 | UP |
| ENSG00000275437 | RP5-908M14.10 | 1.668091 | 0.000539 | 0.017056 | UP |
| ENSG00000275532 | CTB-58E17.3 | 1.658108 | 0.000144 | 0.008096 | UP |
| ENSG00000275557 | RP11-353N4.6 | 1.699684 | 4.63E-10 | 6.25E-06 | UP |
| ENSG00000275638 | RP11-16E23.5 | 1.154153 | 0.003208 | 0.045935 | UP |
| ENSG00000275759 | RP11-131L12.3 | 1.065288 | 0.003342 | 0.046984 | UP |
| ENSG00000275799 | AP001059.7 | 1.230122 | 2.62E-07 | 0.000225 | UP |
| ENSG00000275936 | RP1-278C19.8 | 1.508042 | 0.000199 | 0.009755 | UP |
| ENSG00000276250 | CTD-2349P21.12 | 1.456786 | 0.00041 | 0.014624 | UP |
| ENSG00000276354 | UPF3BP1 | 1.696885 | 3.01E-05 | 0.00333 | UP |
| ENSG00000276362 | RP11-241M13.2 | 1.411215 | 0.002143 | 0.036607 | UP |
| ENSG00000276399 | FLJ36000 | 1.077697 | 4.10E-05 | 0.003963 | UP |
| ENSG00000276473 | CTD-2026J24.1 | 1.509346 | 1.49E-06 | 0.000607 | UP |
| ENSG00000276531 | RP11-205A8.3 | 2.135309 | 1.35E-06 | 0.000574 | UP |
| ENSG00000276542 | RP11-115D19.2 | 1.161599 | 0.00014 | 0.007968 | UP |
| ENSG00000276570 | CTD-2587H24.14 | 1.796914 | 5.63E-05 | 0.004752 | UP |
| ENSG00000276840 | PMS2P10 | 1.410629 | 5.37E-05 | 0.004633 | UP |
| ENSG00000276845 | RP11-374M1.9 | 1.410691 | 1.46E-05 | 0.002224 | UP |
| ENSG00000277072 | STAG3L2 | 1.160854 | 2.95E-07 | 0.000241 | UP |
| ENSG00000277200 | RP11-74E22.8 | 1.045594 | 3.15E-06 | 0.000925 | UP |
| ENSG00000277452 | RN7SL473P | 1.006974 | 1.37E-05 | 0.002143 | UP |
| ENSG00000277561 | GOLGA8IP | 1.46631 | 0.000217 | 0.010231 | UP |
| ENSG00000278022 | RP11-35O15.2 | 1.602819 | 0.000114 | 0.007115 | UP |
| ENSG00000278301 | GRAMD4P3 | 1.141658 | 0.003015 | 0.044359 | UP |
| ENSG00000278346 | CTD-2267D19.7 | 1.433744 | 6.41E-06 | 0.001385 | UP |
| ENSG00000278434 | RP11-709D24.8 | 1.76442 | 8.46E-05 | 0.006008 | UP |
| ENSG00000278972 | CTD-3022L24.1 | 1.521929 | 1.47E-06 | 0.000603 | UP |
| ENSG00000279020 | C18orf15 | 1.991432 | 2.01E-06 | 0.000716 | UP |
| ENSG00000279078 | SND1-IT1 | 1.07723 | 0.000143 | 0.008061 | UP |
| ENSG00000279117 | CTD-2562J17.6 | 1.00491 | 2.40E-07 | 0.000215 | UP |
| ENSG00000231295 | RP4-797C5.2 | 1.961943 | 2.00E-07 | 0.000195 | UP |
| ENSG00000279129 | RP11-264L1.2 | 1.688382 | 3.40E-05 | 0.003565 | UP |
| ENSG00000279162 | CTD-3126B10.2 | 1.451234 | 8.42E-05 | 0.005992 | UP |
| ENSG00000279191 | RP11-803D5.1 | 1.037798 | 0.002915 | 0.043548 | UP |
| ENSG00000279217 | CTA-212A2.3 | 1.050003 | 0.001366 | 0.028591 | UP |
| ENSG00000279265 | AC000123.3 | 1.103974 | 4.00E-07 | 0.000287 | UP |
| ENSG00000279276 | CTA-345G4.1 | 1.659736 | 0.000694 | 0.019567 | UP |
| ENSG00000279294 | RP11-274A11.3 | 1.669011 | 3.64E-08 | 7.36E-05 | UP |
| ENSG00000279339 | CTD-2373H9.3 | 1.834402 | 7.20E-14 | 4.51E-08 | UP |
| ENSG00000279382 | RP11-449J21.3 | 1.473609 | 4.71E-06 | 0.00116 | UP |
| ENSG00000279418 | LINC00244 | 1.116404 | 0.000414 | 0.014691 | UP |
| ENSG00000279531 | RP11-184E9.4 | 1.304888 | 6.66E-06 | 0.001416 | UP |
| ENSG00000279557 | CTD-2210P15.3 | 2.195703 | 8.81E-11 | 2.40E-06 | UP |
| ENSG00000279620 | RP11-1102P22.2 | 2.100667 | 1.94E-06 | 0.000704 | UP |
| ENSG00000279621 | RP11-67A1.4 | 1.125023 | 2.88E-07 | 0.000237 | UP |
| ENSG00000279637 | RP11-196B3.1 | 1.535481 | 0.00036 | 0.013577 | UP |
| ENSG00000279652 | RP1-78B3.1 | 1.478279 | 8.78E-05 | 0.006144 | UP |
| ENSG00000279681 | RP11-274A11.5 | 1.623274 | 7.42E-05 | 0.005559 | UP |
| ENSG00000279759 | CTC-425O23.5 | 1.057798 | 0.000287 | 0.011957 | UP |
| ENSG00000279840 | RP1-228P16.9 | 1.652838 | 3.13E-05 | 0.003404 | UP |
| ENSG00000279891 | FLJ42393 | 2.1576 | 2.80E-08 | 6.31E-05 | UP |
| ENSG00000279901 | CTD-2270P14.2 | 1.066429 | 0.00033 | 0.012948 | UP |
| ENSG00000280020 | CTD-2095E4.4 | 2.226873 | 2.73E-06 | 0.000852 | UP |
| ENSG00000280033 | CTD-2095E4.3 | 1.152781 | 4.97E-05 | 0.004424 | UP |
| ENSG00000280035 | RP11-10J21.2 | 1.397805 | 9.92E-09 | 3.47E-05 | UP |
| ENSG00000280039 | RN7SKP23 | 1.609288 | 0.000628 | 0.018542 | UP |
| ENSG00000280054 | RP1-197B17.7 | 1.373608 | 2.31E-05 | 0.002873 | UP |
| ENSG00000280063 | RP11-295D4.3 | 1.578585 | 4.62E-07 | 0.000312 | UP |
| ENSG00000280109 | PLAC4 | 1.404743 | 3.62E-05 | 0.003698 | UP |
| ENSG00000280119 | AC093642.1 | 1.408532 | 0.002152 | 0.036701 | UP |
| ENSG00000280121 | CTD-3137H5.4 | 1.624255 | 1.37E-10 | 3.08E-06 | UP |
| ENSG00000280153 | RP11-876N24.3 | 1.067779 | 2.19E-05 | 0.002788 | UP |
| ENSG00000280164 | CH507-254M2.3 | 1.480687 | 3.78E-06 | 0.001025 | UP |
| ENSG00000280205 | RP11-504I13.2 | 2.36282 | 1.83E-17 | 3.75E-10 | UP |
| ENSG00000280213 | UCKL1-AS1 | 1.298645 | 0.002138 | 0.036556 | UP |
| ENSG00000280331 | RP11-545D22.1 | 1.48132 | 1.36E-06 | 0.000577 | UP |
| ENSG00000280332 | CTD-2013N17.6 | 1.310995 | 6.16E-05 | 0.004999 | UP |
| ENSG00000280408 | RP11-818O24.2 | 1.467782 | 8.45E-10 | 8.81E-06 | UP |
| ENSG00000280604 | PCBP3-OT1 | 2.270508 | 3.05E-06 | 0.000909 | UP |
| ENSG00000280703 | RP11-275E15.3 | 1.639217 | 0.000425 | 0.014913 | UP |
| ENSG00000280927 | CTBP1-AS | 1.220689 | 2.40E-06 | 0.000791 | UP |
| ENSG00000281100 | RP11-640L9.2 | 1.07915 | 0.001759 | 0.032837 | UP |
| ENSG00000281112 | AL355480.2 | 1.565154 | 4.14E-05 | 0.003985 | UP |
| ENSG00000281691 | RBM5-AS1 | 1.276056 | 0.000371 | 0.013809 | UP |
| ENSG00000282164 | PEG13 | 1.549226 | 7.02E-09 | 2.88E-05 | UP |
| ENSG00000282458 | WASH5P | 1.150588 | 5.26E-06 | 0.001236 | UP |
| ENSG00000282556 | RP11-304C12.5 | 1.311462 | 0.002133 | 0.036512 | UP |
| ENSG00000282787 | RP11-151A10.3 | 1.061154 | 2.87E-06 | 0.000877 | UP |
| ENSG00000282885 | RP11-596C23.6 | 1.38259 | 3.11E-10 | 4.92E-06 | UP |
| ENSG00000282916 | RP11-676F20.4 | 1.255839 | 4.40E-06 | 0.001118 | UP |
| ENSG00000188511 | C22orf34 | -1.26646 | 4.35E-09 | 2.20E-05 | DOWN |
| ENSG00000214659 | KRT8P26 | -1.11877 | 4.64E-10 | 6.25E-06 | DOWN |
| ENSG00000128692 | EIF2S2P4 | -1.06284 | 0.001109 | 0.025468 | DOWN |
| ENSG00000218870 | SLC25A6P6 | -1.86938 | 1.39E-11 | 8.23E-07 | DOWN |
| ENSG00000137970 | RPL7P9 | -1.82606 | 6.41E-14 | 4.22E-08 | DOWN |
| ENSG00000146677 | AC004453.8 | -1.09859 | 0.002603 | 0.040845 | DOWN |
| ENSG00000154874 | CCDC144B | -1.05814 | 2.61E-07 | 0.000225 | DOWN |
| ENSG00000154898 | CCDC144CP | -1.92786 | 5.53E-12 | 4.99E-07 | DOWN |
| ENSG00000161132 | XXbac-B444P24.10 | -1.13754 | 0.000815 | 0.021438 | DOWN |
| ENSG00000163364 | LINC01116 | -1.93533 | 0.002074 | 0.035954 | DOWN |
| ENSG00000165121 | RP11-213G2.3 | -1.08014 | 2.67E-08 | 6.15E-05 | DOWN |
| ENSG00000168852 | TPTE2P5 | -1.91631 | 0.000605 | 0.018167 | DOWN |
| ENSG00000260526 | RP11-73K9.2 | -2.71884 | 0.000368 | 0.013748 | DOWN |
| ENSG00000172912 | COX6B1P3 | -1.10099 | 9.96E-09 | 3.48E-05 | DOWN |
| ENSG00000173810 | PPIAP7 | -1.08461 | 5.41E-06 | 0.001255 | DOWN |
| ENSG00000175741 | RWDD4P2 | -1.18849 | 0.001501 | 0.030068 | DOWN |
| ENSG00000263120 | RP5-1107A17.4 | -1.24578 | 0.00326 | 0.046337 | DOWN |
| ENSG00000178081 | ULK4P3 | -1.18939 | 1.18E-08 | 3.85E-05 | DOWN |
| ENSG00000181214 | OR8G2P | -1.03296 | 1.17E-09 | 1.06E-05 | DOWN |
| ENSG00000181524 | RPL24P4 | -1.04473 | 2.26E-05 | 0.00284 | DOWN |
| ENSG00000181943 | OR4A21P | -1.05168 | 4.30E-06 | 0.001103 | DOWN |
| ENSG00000186594 | MIR22HG | -1.25962 | 2.41E-07 | 0.000215 | DOWN |
| ENSG00000187900 | OR5H7P | -1.48248 | 5.46E-05 | 0.004677 | DOWN |
| ENSG00000188403 | IGHV1OR15-9 | -1.1478 | 3.08E-10 | 4.89E-06 | DOWN |
| ENSG00000189212 | DPY19L2P1 | -1.09836 | 0.003347 | 0.047024 | DOWN |
| ENSG00000197332 | CTC-499B15.5 | -1.2448 | 3.85E-09 | 2.07E-05 | DOWN |
| ENSG00000198671 | RP11-168J19.2 | -2.47292 | 2.60E-11 | 1.18E-06 | DOWN |
| ENSG00000200488 | RN7SKP203 | -1.47209 | 2.58E-09 | 1.66E-05 | DOWN |
| ENSG00000200554 | RNU6-1020P | -1.26966 | 1.86E-07 | 0.000187 | DOWN |
| ENSG00000200645 | RNU6-1210P | -2.41015 | 4.74E-07 | 0.000316 | DOWN |
| ENSG00000201709 | RNU6-686P | -1.01399 | 3.14E-08 | 6.76E-05 | DOWN |
| ENSG00000202024 | RNU6-934P | -3.40938 | 9.19E-06 | 0.001709 | DOWN |
| ENSG00000202198 | RN7SK | -1.08027 | 2.04E-07 | 0.000197 | DOWN |
| ENSG00000202314 | SNORD6 | -1.25111 | 0.001161 | 0.026106 | DOWN |
| ENSG00000202337 | RNU6-8 | -1.09661 | 8.91E-09 | 3.27E-05 | DOWN |
| ENSG00000203644 | RP11-332M2.1 | -2.01974 | 1.52E-05 | 0.002273 | DOWN |
| ENSG00000204117 | RP4-640H8.2 | -1.01489 | 1.41E-07 | 0.000159 | DOWN |
| ENSG00000204929 | AC074391.1 | -1.04816 | 1.11E-05 | 0.001903 | DOWN |
| ENSG00000205184 | SLC10A5P1 | -1.36142 | 3.78E-06 | 0.001025 | DOWN |
| ENSG00000205267 | DGAT2L7P | -1.65349 | 3.25E-05 | 0.003482 | DOWN |
| ENSG00000205791 | LOH12CR2 | -1.04641 | 0.003432 | 0.04775 | DOWN |
| ENSG00000206062 | RP13-212L9.1 | -1.20439 | 1.50E-06 | 0.00061 | DOWN |
| ENSG00000206120 | EGFEM1P | -1.3483 | 0.00011 | 0.006963 | DOWN |
| ENSG00000206142 | KB-1183D5.13 | -1.56096 | 8.16E-11 | 2.29E-06 | DOWN |
| ENSG00000229881 | RP11-321E8.4 | -2.19581 | 3.00E-08 | 6.59E-05 | DOWN |
| ENSG00000206688 | SNORD116-18 | -1.05778 | 0.000232 | 0.010619 | DOWN |
| ENSG00000206802 | RNU6-926P | -1.05265 | 2.43E-07 | 0.000217 | DOWN |
| ENSG00000206896 | RNU6-1124P | -1.24126 | 4.78E-09 | 2.31E-05 | DOWN |
| ENSG00000206918 | RNU6-1181P | -1.01817 | 9.55E-05 | 0.006443 | DOWN |
| ENSG00000207248 | RNU6-1005P | -1.22555 | 0.000546 | 0.017167 | DOWN |
| ENSG00000207366 | RNU6-297P | -1.63786 | 1.02E-05 | 0.001808 | DOWN |
| ENSG00000210135 | MT-TN | -1.98641 | 5.17E-06 | 0.001223 | DOWN |
| ENSG00000210196 | MT-TP | -1.2583 | 0.001014 | 0.024168 | DOWN |
| ENSG00000211454 | AKR7L | -1.03365 | 1.70E-06 | 0.000651 | DOWN |
| ENSG00000211640 | IGLV6-57 | -2.29365 | 4.50E-08 | 8.24E-05 | DOWN |
| ENSG00000211648 | IGLV1-47 | -1.89593 | 0.000337 | 0.01307 | DOWN |
| ENSG00000211657 | IGLV3-32 | -1.20252 | 3.73E-06 | 0.001017 | DOWN |
| ENSG00000211695 | TRGV9 | -1.83602 | 0.003505 | 0.048326 | DOWN |
| ENSG00000211791 | TRAV13-2 | -1.20338 | 0.001856 | 0.033841 | DOWN |
| ENSG00000213036 | RP11-365D23.4 | -1.84658 | 2.22E-09 | 1.52E-05 | DOWN |
| ENSG00000213269 | AC004386.4 | -1.15473 | 5.52E-05 | 0.004703 | DOWN |
| ENSG00000213492 | NT5C3AP1 | -1.95506 | 2.69E-05 | 0.003126 | DOWN |
| ENSG00000213548 | AC005522.6 | -1.23007 | 8.04E-07 | 0.000426 | DOWN |
| ENSG00000213866 | YBX1P10 | -1.37758 | 0.001835 | 0.033621 | DOWN |
| ENSG00000213885 | RPL13AP7 | -1.67536 | 0.000354 | 0.013442 | DOWN |
| ENSG00000213935 | AC092610.12 | -1.88083 | 4.10E-16 | 2.25E-09 | DOWN |
| ENSG00000213976 | CTD-2561J22.2 | -1.65935 | 4.14E-05 | 0.003989 | DOWN |
| ENSG00000214106 | PAXIP1-AS2 | -2.02459 | 8.64E-12 | 6.30E-07 | DOWN |
| ENSG00000214194 | LINC00998 | -1.01878 | 2.69E-05 | 0.003125 | DOWN |
| ENSG00000214485 | RPL7P1 | -1.73641 | 1.36E-12 | 2.26E-07 | DOWN |
| ENSG00000215112 | FAM74A1 | -1.928 | 4.92E-08 | 8.69E-05 | DOWN |
| ENSG00000215149 | KRT18P32 | -1.48282 | 1.36E-06 | 0.000577 | DOWN |
| ENSG00000215151 | ABCD1P2 | -2.15382 | 8.34E-12 | 6.16E-07 | DOWN |
| ENSG00000215241 | RP11-266K4.9 | -1.91698 | 1.04E-16 | 9.71E-10 | DOWN |
| ENSG00000215417 | MIR17HG | -1.34041 | 1.37E-12 | 2.26E-07 | DOWN |
| ENSG00000215811 | BTNL10 | -1.19475 | 0.00046 | 0.015563 | DOWN |
| ENSG00000215887 | ZNF859P | -1.52635 | 2.29E-12 | 3.01E-07 | DOWN |
| ENSG00000216917 | RP5-988G15.1 | -1.71136 | 2.61E-14 | 2.50E-08 | DOWN |
| ENSG00000217195 | RP11-277I20.2 | -1.42339 | 0.00056 | 0.017438 | DOWN |
| ENSG00000217416 | ISCA1P1 | -1.71603 | 1.44E-06 | 0.000596 | DOWN |
| ENSG00000218227 | RP11-889L3.1 | -1.27704 | 0.001235 | 0.026997 | DOWN |
| ENSG00000218305 | CDC14C | -1.04378 | 0.002083 | 0.036028 | DOWN |
| ENSG00000218313 | RP11-393I2.2 | -1.14692 | 5.37E-06 | 0.00125 | DOWN |
| ENSG00000218890 | NUFIP1P | -1.15575 | 4.95E-10 | 6.43E-06 | DOWN |
| ENSG00000219102 | HNRNPA3P12 | -1.3013 | 7.72E-11 | 2.23E-06 | DOWN |
| ENSG00000219507 | FTH1P8 | -1.75334 | 0.003201 | 0.045875 | DOWN |
| ENSG00000219993 | RP11-288G3.3 | -1.46524 | 5.07E-08 | 8.84E-05 | DOWN |
| ENSG00000220212 | OR4F1P | -1.894 | 1.67E-12 | 2.49E-07 | DOWN |
| ENSG00000220598 | SSR1P1 | -2.20101 | 1.91E-15 | 5.53E-09 | DOWN |
| ENSG00000220695 | RP1-121G13.3 | -2.3306 | 7.12E-13 | 1.55E-07 | DOWN |
| ENSG00000222376 | RN7SKP152 | -1.66656 | 7.77E-11 | 2.23E-06 | DOWN |
| ENSG00000223298 | RNY3P8 | -2.94112 | 1.86E-14 | 2.07E-08 | DOWN |
| ENSG00000223550 | SNRPBP1 | -1.54112 | 0.002797 | 0.042556 | DOWN |
| ENSG00000223635 | RP4-613A2.1 | -2.00366 | 1.79E-11 | 9.52E-07 | DOWN |
| ENSG00000223804 | CH17-472G23.2 | -1.27026 | 0.000239 | 0.010787 | DOWN |
| ENSG00000224063 | AC007319.1 | -2.00006 | 0.000115 | 0.007143 | DOWN |
| ENSG00000224091 | AC104389.16 | -1.00174 | 1.27E-05 | 0.002056 | DOWN |
| ENSG00000224138 | AC000123.4 | -1.75912 | 2.51E-06 | 0.000813 | DOWN |
| ENSG00000224177 | LINC00570 | -1.0808 | 0.000756 | 0.020554 | DOWN |
| ENSG00000224224 | HAUS1P2 | -1.12919 | 1.57E-11 | 8.82E-07 | DOWN |
| ENSG00000224324 | THAP5P1 | -1.1173 | 0.000654 | 0.018923 | DOWN |
| ENSG00000224329 | LINC00297 | -1.24626 | 0.000193 | 0.009583 | DOWN |
| ENSG00000224337 | FAM8A3P | -1.27116 | 1.07E-06 | 0.000501 | DOWN |
| ENSG00000224509 | AC010884.1 | -1.61779 | 1.37E-08 | 4.22E-05 | DOWN |
| ENSG00000224517 | HTR2A-AS1 | -1.99275 | 3.12E-05 | 0.003402 | DOWN |
| ENSG00000224568 | AC096669.3 | -1.2895 | 4.84E-06 | 0.001178 | DOWN |
| ENSG00000224658 | RP11-631F7.1 | -1.24583 | 2.90E-13 | 9.61E-08 | DOWN |
| ENSG00000224661 | AC010907.5 | -1.23412 | 0.001092 | 0.025246 | DOWN |
| ENSG00000224739 | AC016735.1 | -2.07393 | 3.10E-06 | 0.000917 | DOWN |
| ENSG00000224826 | AC019109.1 | -1.03831 | 0.000145 | 0.008132 | DOWN |
| ENSG00000224884 | AC034187.2 | -1.04577 | 5.34E-07 | 0.000336 | DOWN |
| ENSG00000224945 | RP11-82L18.2 | -1.23245 | 2.93E-11 | 1.26E-06 | DOWN |
| ENSG00000225125 | RANP4 | -1.37025 | 0.001461 | 0.029648 | DOWN |
| ENSG00000225138 | CTD-2228K2.7 | -1.08358 | 5.18E-07 | 0.000331 | DOWN |
| ENSG00000225292 | RP11-57H14.3 | -1.89166 | 1.89E-06 | 0.000692 | DOWN |
| ENSG00000225385 | RP11-350E12.4 | -1.86699 | 0.000173 | 0.008995 | DOWN |
| ENSG00000225447 | RPS15AP10 | -1.42079 | 3.64E-07 | 0.000271 | DOWN |
| ENSG00000225964 | NRIR | -1.61714 | 9.96E-05 | 0.006591 | DOWN |
| ENSG00000225975 | LINC01534 | -1.37582 | 3.67E-05 | 0.003724 | DOWN |
| ENSG00000225981 | AC102953.4 | -1.582 | 1.26E-06 | 0.000552 | DOWN |
| ENSG00000226091 | LINC00937 | -1.57889 | 7.02E-09 | 2.88E-05 | DOWN |
| ENSG00000226188 | HNRNPA1P3 | -1.50191 | 3.34E-11 | 1.37E-06 | DOWN |
| ENSG00000226259 | GTF2H2B | -1.83572 | 2.61E-05 | 0.003073 | DOWN |
| ENSG00000226278 | PSPHP1 | -1.8038 | 1.69E-05 | 0.002412 | DOWN |
| ENSG00000226425 | RP11-348J12.2 | -1.12837 | 7.00E-06 | 0.001457 | DOWN |
| ENSG00000226481 | ACTR3BP2 | -1.98168 | 4.32E-07 | 0.0003 | DOWN |
| ENSG00000226751 | AF127936.5 | -1.06734 | 7.68E-05 | 0.005675 | DOWN |
| ENSG00000226806 | AC011893.3 | -1.05786 | 2.56E-05 | 0.003044 | DOWN |
| ENSG00000226831 | MED15P3 | -1.31178 | 3.36E-07 | 0.00026 | DOWN |
| ENSG00000226964 | RHEBP2 | -1.94641 | 4.44E-09 | 2.23E-05 | DOWN |
| ENSG00000226975 | AC006987.6 | -1.09838 | 6.43E-06 | 0.001387 | DOWN |
| ENSG00000227034 | RP11-234N17.1 | -1.6312 | 4.65E-06 | 0.001152 | DOWN |
| ENSG00000227043 | RP11-286M16.1 | -1.07371 | 1.56E-09 | 1.25E-05 | DOWN |
| ENSG00000227081 | RP11-543P15.1 | -2.10153 | 0.000762 | 0.020634 | DOWN |
| ENSG00000227352 | ARHGEF7-AS1 | -1.18632 | 8.07E-07 | 0.000427 | DOWN |
| ENSG00000227355 | RP11-162D16.2 | -1.13061 | 0.001213 | 0.026751 | DOWN |
| ENSG00000227388 | RP11-112J3.16 | -1.68043 | 4.61E-09 | 2.27E-05 | DOWN |
| ENSG00000227527 | RP11-223A3.1 | -1.27761 | 1.74E-05 | 0.002445 | DOWN |
| ENSG00000227573 | RP5-1166A24.1 | -2.08192 | 3.18E-12 | 3.63E-07 | DOWN |
| ENSG00000227687 | RP4-681L3.2 | -1.9205 | 3.78E-13 | 1.11E-07 | DOWN |
| ENSG00000227867 | TCEB1P11 | -1.25243 | 1.47E-09 | 1.20E-05 | DOWN |
| ENSG00000228039 | KB-1125A3.10 | -1.50292 | 3.34E-06 | 0.000957 | DOWN |
| ENSG00000228081 | RP11-385M4.2 | -1.84681 | 1.93E-10 | 3.76E-06 | DOWN |
| ENSG00000228195 | RPL5P27 | -1.03385 | 1.42E-06 | 0.00059 | DOWN |
| ENSG00000228232 | GAPDHP1 | -1.02944 | 0.001622 | 0.031421 | DOWN |
| ENSG00000228235 | AP001476.4 | -1.21753 | 0.00088 | 0.022342 | DOWN |
| ENSG00000228305 | AC016734.2 | -1.40588 | 0.001032 | 0.024432 | DOWN |
| ENSG00000228308 | LINC01209 | -1.46095 | 3.26E-17 | 5.06E-10 | DOWN |
| ENSG00000228397 | RP1-224A6.3 | -1.14492 | 0.003234 | 0.046117 | DOWN |
| ENSG00000228431 | ARL5AP3 | -1.26454 | 2.32E-07 | 0.000211 | DOWN |
| ENSG00000228436 | RP5-864K19.4 | -1.43408 | 0.000937 | 0.023167 | DOWN |
| ENSG00000228439 | TSTD3 | -3.05541 | 2.56E-11 | 1.17E-06 | DOWN |
| ENSG00000228489 | RPL21P50 | -1.36265 | 0.000259 | 0.011281 | DOWN |
| ENSG00000228523 | RP11-131O15.2 | -1.72414 | 1.08E-12 | 1.97E-07 | DOWN |
| ENSG00000228554 | AC004837.5 | -1.24709 | 0.000679 | 0.019324 | DOWN |
| ENSG00000228589 | SPCS2P4 | -1.65615 | 3.94E-06 | 0.001047 | DOWN |
| ENSG00000228645 | PHKG1P2 | -1.03486 | 4.11E-07 | 0.000291 | DOWN |
| ENSG00000228648 | RP11-568A7.2 | -1.57571 | 8.98E-08 | 0.000123 | DOWN |
| ENSG00000228869 | COX4I1P2 | -2.50089 | 8.67E-09 | 3.23E-05 | DOWN |
| ENSG00000228929 | RPS13P2 | -1.13998 | 0.001276 | 0.027468 | DOWN |
| ENSG00000229011 | LINC01038 | -1.59705 | 1.46E-06 | 0.000602 | DOWN |
| ENSG00000229066 | AC096649.3 | -1.14399 | 2.62E-12 | 3.27E-07 | DOWN |
| ENSG00000229090 | RP1-232L22__A.1 | -1.17651 | 3.63E-05 | 0.0037 | DOWN |
| ENSG00000229093 | OR51AB1P | -1.95144 | 0.000677 | 0.019294 | DOWN |
| ENSG00000229119 | CTB-63M22.1 | -1.14574 | 7.55E-07 | 0.000411 | DOWN |
| ENSG00000229282 | RP1-40E16.2 | -1.38676 | 2.08E-11 | 1.04E-06 | DOWN |
| ENSG00000229339 | RP11-193I22.2 | -2.17375 | 2.99E-15 | 7.12E-09 | DOWN |
| ENSG00000229425 | AJ006998.2 | -2.32826 | 4.61E-06 | 0.001145 | DOWN |
| ENSG00000229570 | GAPDHP58 | -2.11201 | 5.59E-05 | 0.004734 | DOWN |
| ENSG00000229729 | RP11-159G9.5 | -1.08572 | 3.29E-10 | 5.08E-06 | DOWN |
| ENSG00000206144 | RP11-400K9.2 | -2.30392 | 0.000952 | 0.023363 | DOWN |
| ENSG00000256020 | RP5-1154L15.2 | -2.19656 | 1.99E-13 | 7.86E-08 | DOWN |
| ENSG00000230084 | RP4-613B23.1 | -1.48443 | 4.73E-06 | 0.001163 | DOWN |
| ENSG00000230149 | RP3-508I15.19 | -1.12901 | 1.87E-05 | 0.002547 | DOWN |
| ENSG00000230325 | RP11-385F5.4 | -1.10691 | 9.83E-10 | 9.62E-06 | DOWN |
| ENSG00000230391 | RPSAP23 | -1.55014 | 1.56E-07 | 0.000169 | DOWN |
| ENSG00000230490 | RP11-141M1.3 | -2.05738 | 2.88E-13 | 9.60E-08 | DOWN |
| ENSG00000230590 | FTX | -1.44404 | 0.000219 | 0.010279 | DOWN |
| ENSG00000230817 | LINC01362 | -1.73654 | 6.85E-16 | 2.99E-09 | DOWN |
| ENSG00000230947 | AP000356.2 | -1.27592 | 1.83E-06 | 0.000681 | DOWN |
| ENSG00000230982 | DSTNP1 | -1.49816 | 1.11E-06 | 0.000512 | DOWN |
| ENSG00000231023 | LINC00326 | -1.36664 | 1.06E-17 | 2.64E-10 | DOWN |
| ENSG00000231464 | AC024937.4 | -1.29021 | 7.43E-08 | 0.00011 | DOWN |
| ENSG00000231507 | LINC01353 | -1.30019 | 7.33E-12 | 5.77E-07 | DOWN |
| ENSG00000231607 | DLEU2 | -1.08523 | 8.30E-10 | 8.73E-06 | DOWN |
| ENSG00000231615 | RP11-296O14.2 | -1.39703 | 0.000681 | 0.019349 | DOWN |
| ENSG00000231704 | AC004895.4 | -2.15167 | 1.69E-06 | 0.000649 | DOWN |
| ENSG00000231952 | DPY19L1P2 | -2.02257 | 1.94E-07 | 0.000192 | DOWN |
| ENSG00000232064 | USP9YP33 | -1.29108 | 0.000878 | 0.022323 | DOWN |
| ENSG00000232368 | FTLP2 | -2.66873 | 9.20E-06 | 0.00171 | DOWN |
| ENSG00000232379 | RP11-308D16.1 | -1.57744 | 1.80E-12 | 2.60E-07 | DOWN |
| ENSG00000232394 | AC090696.2 | -1.29066 | 9.04E-10 | 9.17E-06 | DOWN |
| ENSG00000232492 | NPM1P13 | -1.35625 | 0.000639 | 0.018695 | DOWN |
| ENSG00000232528 | RP4-673D20.3 | -1.03164 | 2.50E-05 | 0.003006 | DOWN |
| ENSG00000232557 | RP11-4M23.3 | -1.09991 | 0.000758 | 0.020574 | DOWN |
| ENSG00000232707 | AP1B1P2 | -1.38015 | 0.003282 | 0.046501 | DOWN |
| ENSG00000232768 | RP11-201O14.2 | -2.08063 | 4.98E-10 | 6.44E-06 | DOWN |
| ENSG00000232773 | RP11-1396O13.15 | -1.20824 | 3.28E-06 | 0.000948 | DOWN |
| ENSG00000232788 | AC078883.3 | -1.29274 | 4.94E-05 | 0.00441 | DOWN |
| ENSG00000233026 | MTCO1P5 | -1.5697 | 9.92E-12 | 6.80E-07 | DOWN |
| ENSG00000233125 | ACTBP12 | -1.72667 | 5.33E-06 | 0.001245 | DOWN |
| ENSG00000233219 | RP11-89N17.3 | -1.38082 | 6.51E-17 | 7.44E-10 | DOWN |
| ENSG00000233304 | LINC01346 | -1.28449 | 8.43E-08 | 0.000119 | DOWN |
| ENSG00000233328 | PFN1P1 | -1.48383 | 0.00168 | 0.031986 | DOWN |
| ENSG00000233384 | RP11-100E13.1 | -1.4132 | 3.91E-05 | 0.00385 | DOWN |
| ENSG00000233483 | CTD-2020K17.4 | -1.36726 | 4.45E-14 | 3.43E-08 | DOWN |
| ENSG00000233583 | RP4-635A23.3 | -1.31933 | 2.83E-06 | 0.000871 | DOWN |
| ENSG00000233659 | NDUFA5P4 | -1.09068 | 6.63E-06 | 0.001412 | DOWN |
| ENSG00000233844 | KCNQ5-IT1 | -1.44147 | 0.003631 | 0.049274 | DOWN |
| ENSG00000233866 | LA16c-4G1.3 | -1.11773 | 3.50E-07 | 0.000266 | DOWN |
| ENSG00000234183 | AC004854.4 | -1.38131 | 1.41E-06 | 0.000587 | DOWN |
| ENSG00000234223 | AC003988.1 | -1.41557 | 8.43E-05 | 0.005995 | DOWN |
| ENSG00000234340 | AP000705.8 | -2.48008 | 0.001548 | 0.030622 | DOWN |
| ENSG00000234418 | RP11-560I19.1 | -1.43026 | 0.000111 | 0.006994 | DOWN |
| ENSG00000234456 | MAGI2-AS3 | -2.01329 | 0.000988 | 0.023827 | DOWN |
| ENSG00000234484 | RP1-55C23.7 | -1.04367 | 1.16E-05 | 0.001951 | DOWN |
| ENSG00000234518 | PTGES3P1 | -1.14387 | 0.000109 | 0.00693 | DOWN |
| ENSG00000234645 | YWHAEP5 | -1.019 | 0.00073 | 0.020131 | DOWN |
| ENSG00000234883 | MIR155HG | -1.377 | 0.00067 | 0.019169 | DOWN |
| ENSG00000234962 | LINC00700 | -1.5071 | 0.000216 | 0.010214 | DOWN |
| ENSG00000234996 | RP11-480I12.9 | -1.01047 | 2.28E-06 | 0.000769 | DOWN |
| ENSG00000235032 | BMP7-AS1 | -1.75468 | 9.32E-06 | 0.001724 | DOWN |
| ENSG00000235077 | AC073842.19 | -1.52296 | 0.000455 | 0.015465 | DOWN |
| ENSG00000235136 | RP13-146A14.1 | -1.1367 | 0.002111 | 0.036294 | DOWN |
| ENSG00000235208 | RPL7AL3 | -1.20151 | 5.60E-12 | 5.02E-07 | DOWN |
| ENSG00000235296 | AC137723.5 | -1.06497 | 0.000997 | 0.023939 | DOWN |
| ENSG00000235369 | RPL36AP15 | -1.79728 | 9.81E-09 | 3.46E-05 | DOWN |
| ENSG00000235609 | AF127936.9 | -1.69329 | 3.93E-06 | 0.001046 | DOWN |
| ENSG00000235726 | AC010148.1 | -1.04719 | 3.31E-06 | 0.000952 | DOWN |
| ENSG00000235811 | RP11-510N19.3 | -1.45202 | 1.60E-06 | 0.000632 | DOWN |
| ENSG00000235859 | AC006978.6 | -1.33811 | 3.08E-10 | 4.89E-06 | DOWN |
| ENSG00000235884 | LINC00941 | -1.04002 | 0.000217 | 0.010233 | DOWN |
| ENSG00000235992 | GRAMD4P2 | -1.43835 | 3.72E-14 | 3.10E-08 | DOWN |
| ENSG00000236030 | LINC01036 | -1.61759 | 1.34E-05 | 0.002116 | DOWN |
| ENSG00000236160 | GS1-541M1.2 | -1.58168 | 2.65E-06 | 0.000839 | DOWN |
| ENSG00000236187 | GJA6P | -1.10381 | 5.11E-07 | 0.000329 | DOWN |
| ENSG00000236278 | PEBP1P3 | -1.6454 | 3.46E-09 | 1.95E-05 | DOWN |
| ENSG00000236301 | MRGPRG-AS1 | -1.89522 | 6.97E-05 | 0.005366 | DOWN |
| ENSG00000236451 | AC067956.1 | -1.39518 | 1.56E-13 | 6.84E-08 | DOWN |
| ENSG00000236701 | RP11-549A6.1 | -1.03692 | 4.27E-09 | 2.18E-05 | DOWN |
| ENSG00000236733 | RP11-203L2.4 | -1.50118 | 2.98E-07 | 0.000242 | DOWN |
| ENSG00000236852 | RP11-3D23.1 | -1.58784 | 1.93E-10 | 3.76E-06 | DOWN |
| ENSG00000236863 | RPL23AP23 | -2.49971 | 1.12E-10 | 2.74E-06 | DOWN |
| ENSG00000236922 | LINC01378 | -1.70193 | 1.58E-12 | 2.40E-07 | DOWN |
| ENSG00000237004 | ZNRF2P1 | -1.34004 | 0.000842 | 0.021823 | DOWN |
| ENSG00000237169 | RPL12P27 | -1.9746 | 3.67E-09 | 2.02E-05 | DOWN |
| ENSG00000237197 | IGHD1-7 | -1.18552 | 1.07E-10 | 2.67E-06 | DOWN |
| ENSG00000237296 | SMG1P1 | -1.10637 | 0.000489 | 0.016122 | DOWN |
| ENSG00000237350 | CDC42P6 | -1.04591 | 0.001093 | 0.025258 | DOWN |
| ENSG00000237371 | RP13-152O15.5 | -1.23923 | 2.29E-05 | 0.002859 | DOWN |
| ENSG00000237490 | RP13-926M18.1 | -1.2161 | 0.003563 | 0.048743 | DOWN |
| ENSG00000237576 | AC097495.2 | -1.1829 | 0.002081 | 0.03601 | DOWN |
| ENSG00000237720 | AC011995.1 | -1.39227 | 0.000941 | 0.023208 | DOWN |
| ENSG00000237782 | RP1-192P9.1 | -1.66925 | 1.36E-12 | 2.26E-07 | DOWN |
| ENSG00000237852 | RP4-630A11.3 | -1.83865 | 5.69E-06 | 0.001292 | DOWN |
| ENSG00000237877 | LINC01473 | -1.51389 | 1.79E-05 | 0.002482 | DOWN |
| ENSG00000238120 | LINC01589 | -1.19339 | 6.47E-13 | 1.48E-07 | DOWN |
| ENSG00000238195 | CTA-503F6.2 | -1.29894 | 1.13E-05 | 0.001921 | DOWN |
| ENSG00000238284 | LINC01448 | -1.62733 | 0.002532 | 0.040284 | DOWN |
| ENSG00000238616 | RNU6-300P | -1.64012 | 7.25E-08 | 0.000109 | DOWN |
| ENSG00000239219 | RP11-379K17.4 | -1.19646 | 9.04E-06 | 0.001693 | DOWN |
| ENSG00000239393 | CTD-2301A4.1 | -1.39556 | 5.84E-13 | 1.40E-07 | DOWN |
| ENSG00000239532 | RP11-9D8.1 | -1.29085 | 1.07E-05 | 0.001871 | DOWN |
| ENSG00000240151 | RN7SL826P | -1.89381 | 4.34E-07 | 0.000301 | DOWN |
| ENSG00000240152 | RP11-16N2.1 | -1.06598 | 3.44E-10 | 5.21E-06 | DOWN |
| ENSG00000240210 | RP11-204K16.1 | -1.52882 | 7.41E-05 | 0.005557 | DOWN |
| ENSG00000240376 | RP11-36C20.1 | -1.23061 | 2.14E-07 | 0.000202 | DOWN |
| ENSG00000240435 | RPS12P27 | -1.25365 | 1.99E-09 | 1.43E-05 | DOWN |
| ENSG00000240450 | CSPG4P1Y | -1.14974 | 4.00E-08 | 7.72E-05 | DOWN |
| ENSG00000240452 | MTCO1P29 | -2.30177 | 2.61E-16 | 1.70E-09 | DOWN |
| ENSG00000240590 | RPSAP48 | -1.99134 | 2.95E-17 | 4.86E-10 | DOWN |
| ENSG00000240666 | MME-AS1 | -2.33212 | 5.79E-05 | 0.004828 | DOWN |
| ENSG00000240869 | RN7SL128P | -1.35482 | 1.08E-06 | 0.000504 | DOWN |
| ENSG00000240870 | RPL19P14 | -1.51387 | 5.13E-06 | 0.00122 | DOWN |
| ENSG00000241211 | IQCJ-SCHIP1-AS1 | -1.42436 | 1.11E-09 | 1.02E-05 | DOWN |
| ENSG00000241461 | RN7SL182P | -1.04539 | 0.00028 | 0.011785 | DOWN |
| ENSG00000241772 | AC092620.2 | -1.75999 | 1.89E-10 | 3.73E-06 | DOWN |
| ENSG00000241899 | TPT1P3 | -2.01061 | 7.57E-16 | 3.14E-09 | DOWN |
| ENSG00000241959 | RN7SL76P | -1.62013 | 6.33E-07 | 0.000371 | DOWN |
| ENSG00000241985 | WWTR1-IT1 | -1.47179 | 2.40E-08 | 5.82E-05 | DOWN |
| ENSG00000242029 | RP11-457K10.1 | -1.13159 | 1.86E-05 | 0.002541 | DOWN |
| ENSG00000242341 | RN7SL646P | -1.16523 | 5.42E-09 | 2.48E-05 | DOWN |
| ENSG00000242391 | RP4-631H13.2 | -1.84658 | 4.03E-13 | 1.15E-07 | DOWN |
| ENSG00000242520 | MAGEA5 | -1.00641 | 0.000528 | 0.016855 | DOWN |
| ENSG00000242756 | RHOT1P3 | -1.82603 | 6.18E-08 | 9.93E-05 | DOWN |
| ENSG00000242931 | RPL7P49 | -1.09283 | 0.001307 | 0.027848 | DOWN |
| ENSG00000243260 | RN7SL558P | -1.44045 | 0.000177 | 0.009133 | DOWN |
| ENSG00000243273 | RP11-166N6.2 | -1.8462 | 5.04E-07 | 0.000327 | DOWN |
| ENSG00000243838 | PSMC1P7 | -1.06868 | 0.00056 | 0.017438 | DOWN |
| ENSG00000244000 | AC006366.3 | -1.40137 | 8.82E-12 | 6.35E-07 | DOWN |
| ENSG00000244089 | HMGB1P30 | -3.29608 | 1.03E-09 | 9.83E-06 | DOWN |
| ENSG00000244194 | RN7SL218P | -2.56342 | 5.73E-09 | 2.56E-05 | DOWN |
| ENSG00000244313 | RP11-425L10.1 | -1.69809 | 2.83E-11 | 1.24E-06 | DOWN |
| ENSG00000244346 | RP11-531F16.3 | -1.36394 | 0.000131 | 0.007676 | DOWN |
| ENSG00000244381 | RP11-3K16.1 | -1.00036 | 6.28E-07 | 0.00037 | DOWN |
| ENSG00000244720 | RP11-402J7.2 | -1.66646 | 3.74E-09 | 2.04E-05 | DOWN |
| ENSG00000244743 | RP11-8P13.1 | -1.36803 | 4.46E-05 | 0.004153 | DOWN |
| ENSG00000245970 | KB-1208A12.3 | -1.09924 | 0.003427 | 0.047707 | DOWN |
| ENSG00000246273 | SBF2-AS1 | -1.6774 | 0.000453 | 0.015428 | DOWN |
| ENSG00000246448 | RP13-578N3.3 | -1.74435 | 0.000149 | 0.008268 | DOWN |
| ENSG00000247095 | MIR210HG | -1.90524 | 0.000884 | 0.022409 | DOWN |
| ENSG00000248307 | LINC00616 | -1.02244 | 0.000487 | 0.01609 | DOWN |
| ENSG00000248396 | TOMM22P4 | -1.22588 | 1.06E-05 | 0.001853 | DOWN |
| ENSG00000248428 | CTC-551A13.1 | -1.01623 | 1.01E-05 | 0.001801 | DOWN |
| ENSG00000248538 | RP11-10A14.5 | -1.05817 | 2.31E-07 | 0.000211 | DOWN |
| ENSG00000248645 | RP11-366M4.6 | -1.33435 | 1.53E-05 | 0.002275 | DOWN |
| ENSG00000248834 | MARK2P5 | -1.13699 | 3.29E-09 | 1.90E-05 | DOWN |
| ENSG00000249138 | SLED1 | -1.3924 | 1.20E-10 | 2.84E-06 | DOWN |
| ENSG00000249216 | RP11-227F19.5 | -1.06796 | 0.000665 | 0.019104 | DOWN |
| ENSG00000249249 | AC010226.4 | -1.19091 | 0.000565 | 0.017502 | DOWN |
| ENSG00000249405 | RP11-317O24.1 | -1.45256 | 1.31E-12 | 2.21E-07 | DOWN |
| ENSG00000249584 | RP11-478P10.1 | -1.01142 | 8.11E-08 | 0.000116 | DOWN |
| ENSG00000249668 | KRT18P56 | -1.06315 | 9.80E-09 | 3.46E-05 | DOWN |
| ENSG00000249675 | RP11-217C7.1 | -1.36623 | 2.66E-08 | 6.14E-05 | DOWN |
| ENSG00000249679 | RP11-279O9.4 | -1.01845 | 2.21E-10 | 4.04E-06 | DOWN |
| ENSG00000249741 | RP11-673E1.3 | -1.33038 | 0.001013 | 0.024168 | DOWN |
| ENSG00000249746 | RP11-254I22.3 | -1.00495 | 5.59E-05 | 0.004736 | DOWN |
| ENSG00000249828 | RP11-1281K21.2 | -1.09124 | 1.56E-12 | 2.40E-07 | DOWN |
| ENSG00000249835 | VCAN-AS1 | -1.67974 | 1.79E-05 | 0.002483 | DOWN |
| ENSG00000249855 | EEF1A1P19 | -1.15143 | 5.67E-08 | 9.44E-05 | DOWN |
| ENSG00000250107 | CACNA1G-AS1 | -1.23307 | 2.04E-05 | 0.002674 | DOWN |
| ENSG00000250320 | CTD-2269F5.1 | -1.50613 | 2.31E-10 | 4.14E-06 | DOWN |
| ENSG00000250471 | GMPSP1 | -1.4083 | 2.18E-09 | 1.51E-05 | DOWN |
| ENSG00000250567 | CTD-2154H6.1 | -1.42149 | 1.14E-05 | 0.001933 | DOWN |
| ENSG00000251182 | RP11-617I14.1 | -1.36689 | 2.27E-13 | 8.49E-08 | DOWN |
| ENSG00000251234 | PSMA2P2 | -1.58414 | 0.000167 | 0.008827 | DOWN |
| ENSG00000251359 | WWC2-AS2 | -1.47349 | 3.84E-09 | 2.07E-05 | DOWN |
| ENSG00000251432 | RP11-420A23.1 | -1.09097 | 2.99E-05 | 0.003324 | DOWN |
| ENSG00000251600 | RP11-673E1.1 | -2.30175 | 4.37E-08 | 8.12E-05 | DOWN |
| ENSG00000251741 | RNU4ATAC13P | -1.2057 | 4.35E-08 | 8.09E-05 | DOWN |
| ENSG00000251804 | RNU6-1294P | -1.42977 | 5.97E-12 | 5.20E-07 | DOWN |
| ENSG00000251952 | RNU6-1219P | -1.31051 | 6.21E-06 | 0.00136 | DOWN |
| ENSG00000251973 | RNU6-473P | -1.01605 | 2.15E-06 | 0.000743 | DOWN |
| ENSG00000252028 | RN7SKP52 | -1.29734 | 9.95E-12 | 6.80E-07 | DOWN |
| ENSG00000252283 | Vault | -1.10469 | 2.51E-05 | 0.00301 | DOWN |
| ENSG00000252297 | RNU6-875P | -1.65321 | 1.12E-08 | 3.73E-05 | DOWN |
| ENSG00000252400 | RNU6-1291P | -1.01589 | 4.75E-08 | 8.51E-05 | DOWN |
| ENSG00000252767 | RNU6-250P | -1.26775 | 1.04E-09 | 9.90E-06 | DOWN |
| ENSG00000253106 | RP11-158K1.3 | -1.15746 | 1.17E-08 | 3.84E-05 | DOWN |
| ENSG00000253225 | RP11-1057N3.2 | -1.83998 | 6.13E-12 | 5.27E-07 | DOWN |
| ENSG00000253235 | RP11-434I12.4 | -1.34143 | 6.35E-06 | 0.001378 | DOWN |
| ENSG00000253363 | RP11-962G15.1 | -1.164 | 1.77E-07 | 0.000182 | DOWN |
| ENSG00000253377 | RP11-566H8.3 | -1.05481 | 0.003426 | 0.047703 | DOWN |
| ENSG00000253381 | RP11-359E19.1 | -1.44701 | 4.13E-11 | 1.54E-06 | DOWN |
| ENSG00000253418 | SNX18P27 | -1.06007 | 0.000138 | 0.007912 | DOWN |
| ENSG00000253422 | CTB-47B8.4 | -2.53473 | 9.70E-18 | 2.53E-10 | DOWN |
| ENSG00000253483 | CTC-756D1.1 | -1.76575 | 1.86E-06 | 0.000686 | DOWN |
| ENSG00000253562 | CTD-2340D6.2 | -1.17961 | 1.09E-11 | 7.16E-07 | DOWN |
| ENSG00000253568 | RP11-386D6.3 | -1.1386 | 3.74E-11 | 1.45E-06 | DOWN |
| ENSG00000253639 | SUMO2P18 | -1.18302 | 0.000118 | 0.007235 | DOWN |
| ENSG00000253656 | KB-1568E2.1 | -1.22812 | 2.51E-05 | 0.00301 | DOWN |
| ENSG00000253875 | RP11-16P20.3 | -1.31829 | 5.51E-08 | 9.28E-05 | DOWN |
| ENSG00000253915 | MAPRE1P1 | -2.8934 | 4.83E-17 | 6.40E-10 | DOWN |
| ENSG00000253955 | CTB-33O18.3 | -1.95044 | 0.000563 | 0.017484 | DOWN |
| ENSG00000254274 | TDGF1P5 | -1.34214 | 1.37E-10 | 3.08E-06 | DOWN |
| ENSG00000254372 | RP11-343P9.1 | -1.24384 | 4.02E-17 | 5.74E-10 | DOWN |
| ENSG00000254492 | KB-1073A2.1 | -1.26031 | 2.85E-11 | 1.24E-06 | DOWN |
| ENSG00000254495 | AP000487.4 | -1.21489 | 7.19E-06 | 0.001479 | DOWN |
| ENSG00000254518 | RP11-347H15.4 | -1.50663 | 6.92E-05 | 0.005346 | DOWN |
| ENSG00000254562 | LINC01493 | -1.03157 | 3.54E-08 | 7.24E-05 | DOWN |
| ENSG00000254574 | RP11-429J17.4 | -1.02418 | 2.28E-05 | 0.002851 | DOWN |
| ENSG00000254606 | RP11-22P4.2 | -1.21689 | 9.51E-08 | 0.000127 | DOWN |
| ENSG00000254650 | RP11-665E10.5 | -1.00786 | 6.97E-06 | 0.001454 | DOWN |
| ENSG00000254680 | RP11-265D17.2 | -1.32577 | 5.26E-07 | 0.000334 | DOWN |
| ENSG00000254721 | RP11-805J14.5 | -1.03993 | 9.50E-06 | 0.001743 | DOWN |
| ENSG00000254777 | AC022182.1 | -1.06783 | 1.68E-10 | 3.47E-06 | DOWN |
| ENSG00000254780 | RP11-793I11.1 | -1.18864 | 0.000243 | 0.010885 | DOWN |
| ENSG00000255138 | GLTPP1 | -1.43746 | 0.000429 | 0.014983 | DOWN |
| ENSG00000255160 | RP11-428C19.5 | -1.02027 | 1.33E-09 | 1.14E-05 | DOWN |
| ENSG00000255198 | SNHG9 | -1.66222 | 1.76E-06 | 0.000666 | DOWN |
| ENSG00000255216 | RP11-831A10.1 | -1.38564 | 3.02E-09 | 1.80E-05 | DOWN |
| ENSG00000255295 | RP11-745I13.1 | -1.26618 | 1.49E-06 | 0.000607 | DOWN |
| ENSG00000255870 | RP11-667M19.5 | -1.05703 | 1.01E-05 | 0.001804 | DOWN |
| ENSG00000255882 | RP11-290C10.1 | -2.11474 | 4.58E-06 | 0.001141 | DOWN |
| ENSG00000230006 | ANKRD36BP2 | -2.48415 | 0.00145 | 0.029534 | DOWN |
| ENSG00000256039 | RP11-291B21.2 | -1.88317 | 9.37E-07 | 0.000465 | DOWN |
| ENSG00000256152 | RP11-463O12.3 | -1.10714 | 6.34E-08 | 0.0001 | DOWN |
| ENSG00000256304 | CCDC150P1 | -1.83205 | 0.000211 | 0.010071 | DOWN |
| ENSG00000256385 | UBE2NP1 | -2.1641 | 2.51E-08 | 5.96E-05 | DOWN |
| ENSG00000256512 | RP11-860B13.3 | -1.55845 | 7.67E-13 | 1.61E-07 | DOWN |
| ENSG00000256637 | RP11-76I14.1 | -1.16785 | 3.83E-07 | 0.00028 | DOWN |
| ENSG00000256704 | SDCCAG3P1 | -1.63099 | 7.31E-12 | 5.77E-07 | DOWN |
| ENSG00000257109 | OR4F28P | -1.65433 | 6.40E-16 | 2.89E-09 | DOWN |
| ENSG00000257167 | TMPO-AS1 | -1.56231 | 3.04E-05 | 0.003351 | DOWN |
| ENSG00000257764 | RP11-1143G9.4 | -1.37041 | 2.30E-05 | 0.002862 | DOWN |
| ENSG00000257818 | C1GALT1P1 | -1.70976 | 2.28E-05 | 0.002853 | DOWN |
| ENSG00000258056 | RP11-644F5.11 | -1.12969 | 0.000147 | 0.008209 | DOWN |
| ENSG00000258088 | RP11-114H23.2 | -2.59837 | 0.00224 | 0.037566 | DOWN |
| ENSG00000258131 | RP11-541G9.1 | -1.64912 | 0.002632 | 0.041106 | DOWN |
| ENSG00000258175 | RP11-412H8.2 | -1.22963 | 6.46E-07 | 0.000376 | DOWN |
| ENSG00000258216 | RP11-654D12.2 | -1.01962 | 3.00E-05 | 0.003325 | DOWN |
| ENSG00000258376 | RP4-647C14.2 | -1.01223 | 1.83E-06 | 0.000681 | DOWN |
| ENSG00000258407 | RP11-300J18.2 | -2.32436 | 0.000102 | 0.006691 | DOWN |
| ENSG00000258419 | RP11-588P7.1 | -1.0811 | 3.64E-05 | 0.003709 | DOWN |
| ENSG00000258443 | RP3-414A15.11 | -1.6183 | 0.000255 | 0.011185 | DOWN |
| ENSG00000258455 | RP11-665C16.5 | -1.66854 | 2.63E-06 | 0.000834 | DOWN |
| ENSG00000258457 | RP11-298I3.4 | -1.72991 | 0.002352 | 0.038616 | DOWN |
| ENSG00000258895 | CTD-2643K12.1 | -1.63151 | 4.98E-09 | 2.36E-05 | DOWN |
| ENSG00000258981 | COX5AP2 | -1.00519 | 0.000368 | 0.013733 | DOWN |
| ENSG00000259106 | RP11-242P2.2 | -1.44014 | 3.46E-13 | 1.05E-07 | DOWN |
| ENSG00000259137 | RP11-305B6.1 | -1.49831 | 6.59E-07 | 0.00038 | DOWN |
| ENSG00000259285 | CTD-2330J20.2 | -1.51754 | 4.18E-08 | 7.91E-05 | DOWN |
| ENSG00000259393 | RP11-313H3.1 | -1.75736 | 9.93E-12 | 6.80E-07 | DOWN |
| ENSG00000259600 | RP11-925D8.3 | -2.01784 | 0.0003 | 0.012254 | DOWN |
| ENSG00000259604 | RP11-66B24.1 | -1.36521 | 1.18E-08 | 3.84E-05 | DOWN |
| ENSG00000259628 | RP11-467H10.2 | -1.47719 | 1.87E-11 | 9.77E-07 | DOWN |
| ENSG00000259706 | HSP90B2P | -1.74534 | 4.81E-08 | 8.57E-05 | DOWN |
| ENSG00000259737 | RP11-475A13.1 | -1.38892 | 5.54E-05 | 0.00471 | DOWN |
| ENSG00000259774 | RP11-182J1.13 | -1.10064 | 0.000493 | 0.016192 | DOWN |
| ENSG00000259781 | RP11-673C5.1 | -1.37195 | 3.76E-06 | 0.001021 | DOWN |
| ENSG00000259793 | RP11-400N9.1 | -1.16245 | 7.60E-07 | 0.000412 | DOWN |
| ENSG00000259822 | RP11-1292F20.1 | -1.15927 | 0.001671 | 0.031932 | DOWN |
| ENSG00000259889 | RP11-315F22.1 | -1.17025 | 3.48E-12 | 3.81E-07 | DOWN |
| ENSG00000259924 | RP11-16E23.4 | -1.78664 | 8.82E-11 | 2.40E-06 | DOWN |
| ENSG00000259939 | RP11-77H9.5 | -1.46021 | 1.14E-06 | 0.000521 | DOWN |
| ENSG00000259986 | RP11-382A20.4 | -1.41204 | 0.00195 | 0.034731 | DOWN |
| ENSG00000260171 | RP11-95H11.1 | -1.04523 | 1.82E-08 | 4.95E-05 | DOWN |
| ENSG00000260493 | RP11-219B4.7 | -1.03313 | 0.000976 | 0.023686 | DOWN |
| ENSG00000260589 | STAM-AS1 | -1.62394 | 0.000275 | 0.011666 | DOWN |
| ENSG00000260629 | BGLT3 | -1.71863 | 1.76E-06 | 0.000666 | DOWN |
| ENSG00000260641 | RP11-1299A16.3 | -1.69564 | 2.30E-07 | 0.00021 | DOWN |
| ENSG00000260702 | LA16c-349E11.1 | -1.29404 | 4.33E-08 | 8.08E-05 | DOWN |
| ENSG00000260740 | AC026471.6 | -1.69208 | 7.62E-13 | 1.61E-07 | DOWN |
| ENSG00000260773 | RP11-352G18.2 | -1.74994 | 1.35E-07 | 0.000155 | DOWN |
| ENSG00000260828 | HMGB3P32 | -1.07938 | 7.58E-07 | 0.000412 | DOWN |
| ENSG00000260920 | RP1-228H13.5 | -1.18755 | 0.00224 | 0.037562 | DOWN |
| ENSG00000260932 | RP11-483P21.6 | -1.09698 | 3.46E-08 | 7.16E-05 | DOWN |
| ENSG00000261033 | RP11-209D14.2 | -1.29098 | 0.000785 | 0.020997 | DOWN |
| ENSG00000261113 | RP11-141O15.1 | -1.65241 | 5.93E-16 | 2.80E-09 | DOWN |
| ENSG00000261127 | RP11-17M15.2 | -2.00099 | 5.69E-13 | 1.39E-07 | DOWN |
| ENSG00000261270 | RP11-325K4.3 | -1.51926 | 3.77E-07 | 0.000277 | DOWN |
| ENSG00000261458 | RP11-787D11.1 | -1.30391 | 3.32E-11 | 1.37E-06 | DOWN |
| ENSG00000261541 | RP11-626K17.3 | -1.62738 | 1.90E-11 | 9.86E-07 | DOWN |
| ENSG00000261775 | RP11-10O17.3 | -1.03474 | 0.000751 | 0.020483 | DOWN |
| ENSG00000262172 | CTD-2529O21.1 | -1.43755 | 1.50E-06 | 0.000609 | DOWN |
| ENSG00000262294 | RP11-1260E13.2 | -1.27534 | 0.000121 | 0.007337 | DOWN |
| ENSG00000262339 | RP11-1197K16.2 | -1.63865 | 3.89E-05 | 0.003836 | DOWN |
| ENSG00000263011 | RP11-473M20.11 | -1.84586 | 5.75E-16 | 2.77E-09 | DOWN |
| ENSG00000263199 | RP11-372K20.1 | -1.17464 | 4.13E-06 | 0.001077 | DOWN |
| ENSG00000263232 | ATP5A1P3 | -1.79648 | 6.12E-06 | 0.001349 | DOWN |
| ENSG00000263482 | ANTXRLP1 | -1.1878 | 0.00025 | 0.011057 | DOWN |
| ENSG00000263503 | RP11-707O23.5 | -1.15864 | 2.30E-07 | 0.000211 | DOWN |
| ENSG00000263934 | SNORD3A | -1.94193 | 0.001201 | 0.026591 | DOWN |
| ENSG00000264293 | RN7SL657P | -1.13166 | 1.46E-05 | 0.002224 | DOWN |
| ENSG00000264340 | RP11-231E4.3 | -1.01383 | 0.000635 | 0.018645 | DOWN |
| ENSG00000264608 | RP11-192H23.8 | -1.46057 | 4.22E-05 | 0.004029 | DOWN |
| ENSG00000264706 | RN7SL217P | -1.13704 | 1.44E-10 | 3.17E-06 | DOWN |
| ENSG00000264750 | RP11-277J6.2 | -1.78925 | 7.98E-05 | 0.005802 | DOWN |
| ENSG00000264920 | RP11-6N17.4 | -1.34538 | 3.35E-10 | 5.12E-06 | DOWN |
| ENSG00000264940 | SNORD3C | -1.58023 | 0.000202 | 0.009849 | DOWN |
| ENSG00000265182 | SRP72P1 | -2.67405 | 0.001761 | 0.032853 | DOWN |
| ENSG00000265185 | SNORD3B-1 | -1.57986 | 0.002269 | 0.037828 | DOWN |
| ENSG00000265257 | RP11-21J18.1 | -1.77195 | 1.54E-10 | 3.28E-06 | DOWN |
| ENSG00000265554 | RP11-419J16.1 | -1.46597 | 2.52E-08 | 5.97E-05 | DOWN |
| ENSG00000265735 | RN7SL5P | -1.14967 | 0.001628 | 0.031478 | DOWN |
| ENSG00000265946 | RP11-752P2.2 | -1.01648 | 1.65E-07 | 0.000174 | DOWN |
| ENSG00000266050 | RP11-822E23.6 | -1.22694 | 7.15E-10 | 8.03E-06 | DOWN |
| ENSG00000266128 | RN7SL366P | -1.73319 | 0.000939 | 0.02319 | DOWN |
| ENSG00000266166 | RN7SL557P | -1.04538 | 1.24E-07 | 0.000148 | DOWN |
| ENSG00000266283 | RP11-627G18.1 | -1.13869 | 0.002956 | 0.043877 | DOWN |
| ENSG00000266308 | RN7SL510P | -1.54524 | 3.21E-08 | 6.85E-05 | DOWN |
| ENSG00000266397 | RP11-737O24.1 | -1.53923 | 5.15E-06 | 0.001222 | DOWN |
| ENSG00000266477 | RN7SL616P | -1.68424 | 0.000109 | 0.00693 | DOWN |
| ENSG00000266497 | RP11-995C19.2 | -1.52532 | 1.03E-05 | 0.001829 | DOWN |
| ENSG00000267027 | AC011524.2 | -2.02118 | 1.90E-05 | 0.00257 | DOWN |
| ENSG00000267218 | AC005336.5 | -1.53418 | 3.86E-05 | 0.003822 | DOWN |
| ENSG00000267233 | HNRNPA3P16 | -1.3562 | 7.55E-10 | 8.28E-06 | DOWN |
| ENSG00000267396 | RP11-845C23.3 | -1.17217 | 0.001761 | 0.032858 | DOWN |
| ENSG00000267551 | AC005264.2 | -1.36821 | 4.19E-09 | 2.17E-05 | DOWN |
| ENSG00000267571 | AC104532.4 | -1.26395 | 2.49E-09 | 1.62E-05 | DOWN |
| ENSG00000267643 | RP11-64C12.4 | -1.34187 | 2.39E-08 | 5.82E-05 | DOWN |
| ENSG00000267659 | LINC01482 | -1.38119 | 2.55E-06 | 0.000819 | DOWN |
| ENSG00000267731 | RP11-147L13.8 | -1.20786 | 0.001163 | 0.026122 | DOWN |
| ENSG00000267750 | RUNDC3A-AS1 | -1.47576 | 4.17E-05 | 0.003999 | DOWN |
| ENSG00000268475 | CTC-435M10.6 | -1.13747 | 5.47E-09 | 2.49E-05 | DOWN |
| ENSG00000268486 | ABC7-42418200C9.1 | -1.23031 | 3.61E-06 | 0.000999 | DOWN |
| ENSG00000268560 | CTD-2332E11.2 | -1.27136 | 1.72E-09 | 1.32E-05 | DOWN |
| ENSG00000268568 | AC007228.9 | -1.05294 | 4.53E-11 | 1.63E-06 | DOWN |
| ENSG00000268823 | CTC-457E21.6 | -1.44765 | 9.67E-11 | 2.53E-06 | DOWN |
| ENSG00000268845 | RP11-1137G4.3 | -1.73031 | 1.17E-12 | 2.07E-07 | DOWN |
| ENSG00000269001 | ZNF818P | -1.44441 | 4.10E-08 | 7.83E-05 | DOWN |
| ENSG00000269050 | CTC-360G5.6 | -1.56315 | 2.00E-15 | 5.66E-09 | DOWN |
| ENSG00000269069 | CTC-471F3.5 | -1.45824 | 4.63E-08 | 8.39E-05 | DOWN |
| ENSG00000269289 | CTB-92J24.3 | -1.0056 | 2.71E-07 | 0.000229 | DOWN |
| ENSG00000269506 | RP11-571I18.5 | -1.47148 | 0.00171 | 0.032308 | DOWN |
| ENSG00000269887 | RP11-477H21.2 | -1.6071 | 0.000818 | 0.021482 | DOWN |
| ENSG00000269893 | SNHG8 | -1.16082 | 2.38E-05 | 0.002919 | DOWN |
| ENSG00000269899 | RP11-589N15.2 | -1.07014 | 0.001161 | 0.026106 | DOWN |
| ENSG00000270110 | RP5-1139B12.4 | -1.12094 | 3.84E-06 | 0.001033 | DOWN |
| ENSG00000270402 | CTC-412M14.5 | -1.29067 | 0.001877 | 0.034041 | DOWN |
| ENSG00000270689 | BUD13P1 | -1.45998 | 1.82E-13 | 7.48E-08 | DOWN |
| ENSG00000270718 | RP11-421H10.2 | -1.04996 | 3.92E-07 | 0.000283 | DOWN |
| ENSG00000270748 | IGKV2OR2-1 | -1.77571 | 0.00202 | 0.035443 | DOWN |
| ENSG00000270764 | CTB-152G17.5 | -1.08561 | 3.61E-05 | 0.003689 | DOWN |
| ENSG00000270849 | CHCHD2P3 | -1.11123 | 2.59E-12 | 3.25E-07 | DOWN |
| ENSG00000270872 | SRGAP2D | -1.68646 | 1.89E-05 | 0.002559 | DOWN |
| ENSG00000270960 | RP11-366M4.18 | -1.39193 | 2.16E-11 | 1.06E-06 | DOWN |
| ENSG00000271287 | BCRP9 | -1.52152 | 3.30E-06 | 0.000951 | DOWN |
| ENSG00000279494 | RP11-494O16.4 | -2.17659 | 7.74E-12 | 5.94E-07 | DOWN |
| ENSG00000271347 | RP11-701H24.7 | -2.1154 | 4.51E-06 | 0.001134 | DOWN |
| ENSG00000271496 | SNRPGP20 | -1.76733 | 0.001789 | 0.033129 | DOWN |
| ENSG00000271554 | RP4-665N4.8 | -1.09661 | 0.000717 | 0.019919 | DOWN |
| ENSG00000271588 | LARP7P1 | -1.32874 | 3.89E-09 | 2.08E-05 | DOWN |
| ENSG00000271612 | HSPE1P14 | -1.44628 | 1.05E-06 | 0.000496 | DOWN |
| ENSG00000271646 | RP11-326I11.3 | -1.60001 | 7.09E-12 | 5.69E-07 | DOWN |
| ENSG00000271736 | RP11-85G21.3 | -1.92861 | 3.35E-06 | 0.000959 | DOWN |
| ENSG00000271893 | RP11-762E8.1 | -1.36951 | 0.001491 | 0.029961 | DOWN |
| ENSG00000272054 | RP11-423P10.2 | -1.27708 | 0.001114 | 0.025528 | DOWN |
| ENSG00000272248 | RP3-406P24.4 | -1.88189 | 4.85E-09 | 2.32E-05 | DOWN |
| ENSG00000272446 | RP1-225E12.3 | -1.0696 | 8.49E-08 | 0.000119 | DOWN |
| ENSG00000272579 | RP11-101E13.5 | -1.44058 | 3.86E-09 | 2.07E-05 | DOWN |
| ENSG00000272825 | LL21NC02-1C16.2 | -1.00748 | 0.000333 | 0.013008 | DOWN |
| ENSG00000272888 | LINC01578 | -1.49347 | 6.54E-14 | 4.25E-08 | DOWN |
| ENSG00000272917 | RP11-705C15.5 | -1.13637 | 9.86E-09 | 3.47E-05 | DOWN |
| ENSG00000273248 | RP11-399K21.13 | -1.55049 | 9.77E-09 | 3.45E-05 | DOWN |
| ENSG00000273271 | AP000254.8 | -1.06818 | 0.002522 | 0.040195 | DOWN |
| ENSG00000273437 | RP11-434H6.7 | -1.06212 | 0.000925 | 0.022989 | DOWN |
| ENSG00000274173 | RP4-568C11.4 | -2.10746 | 5.83E-07 | 0.000354 | DOWN |
| ENSG00000274312 | RP11-167B3.2 | -1.05817 | 2.06E-10 | 3.90E-06 | DOWN |
| ENSG00000274422 | LL22NC03-2H8.5 | -3.6311 | 5.42E-14 | 3.85E-08 | DOWN |
| ENSG00000274457 | AC000041.10 | -1.36472 | 2.69E-07 | 0.000229 | DOWN |
| ENSG00000274667 | RP11-31H5.3 | -1.64876 | 7.61E-10 | 8.30E-06 | DOWN |
| ENSG00000274691 | RP11-310E22.6 | -1.48631 | 0.000598 | 0.01805 | DOWN |
| ENSG00000274758 | RP1-59D14.10 | -1.59446 | 0.000293 | 0.012092 | DOWN |
| ENSG00000274987 | RP11-295I5.3 | -1.74184 | 7.09E-07 | 0.000397 | DOWN |
| ENSG00000275197 | RP11-295I5.4 | -1.68673 | 0.000278 | 0.011738 | DOWN |
| ENSG00000275418 | RP11-126O1.6 | -1.24384 | 4.55E-05 | 0.004199 | DOWN |
| ENSG00000275538 | RNVU1-19 | -2.08204 | 8.18E-05 | 0.005893 | DOWN |
| ENSG00000275852 | RP13-379L11.4 | -2.02169 | 1.62E-06 | 0.000636 | DOWN |
| ENSG00000276182 | RP11-872J21.5 | -1.77626 | 2.79E-05 | 0.003194 | DOWN |
| ENSG00000276229 | Six3os1_3 | -1.25154 | 0.000477 | 0.015879 | DOWN |
| ENSG00000276487 | RP11-417O18.2 | -1.52944 | 1.64E-05 | 0.002369 | DOWN |
| ENSG00000276577 | RNU6-467P | -1.27417 | 1.16E-07 | 0.000142 | DOWN |
| ENSG00000276691 | RP5-1057I20.5 | -1.1093 | 1.86E-09 | 1.38E-05 | DOWN |
| ENSG00000276842 | RP11-713N11.6 | -1.32686 | 0.001627 | 0.031472 | DOWN |
| ENSG00000277341 | RNU6-489P | -1.69335 | 6.09E-17 | 7.28E-10 | DOWN |
| ENSG00000277444 | ZEB2_AS1_4 | -1.5996 | 0.002533 | 0.040286 | DOWN |
| ENSG00000277527 | AC007251.2 | -2.46511 | 2.67E-07 | 0.000228 | DOWN |
| ENSG00000277624 | RP11-374M1.11 | -1.34846 | 1.28E-08 | 4.03E-05 | DOWN |
| ENSG00000277879 | RP11-129M16.4 | -1.3748 | 1.79E-05 | 0.002482 | DOWN |
| ENSG00000278001 | RP11-157L3.11 | -1.11507 | 1.08E-12 | 1.97E-07 | DOWN |
| ENSG00000278238 | RP11-245D16.4 | -2.01315 | 1.53E-07 | 0.000167 | DOWN |
| ENSG00000278376 | RP11-158I9.8 | -1.22605 | 0.000337 | 0.01307 | DOWN |
| ENSG00000278577 | RP11-340I6.11 | -1.23641 | 4.39E-08 | 8.13E-05 | DOWN |
| ENSG00000278582 | RP11-1H8.6 | -1.21059 | 3.31E-05 | 0.003513 | DOWN |
| ENSG00000278626 | RP11-467L19.16 | -1.35342 | 1.19E-08 | 3.86E-05 | DOWN |
| ENSG00000278797 | LLNLR-276E7.1 | -1.56228 | 0.001808 | 0.033348 | DOWN |
| ENSG00000279045 | RP11-643G5.7 | -1.24581 | 7.56E-06 | 0.001524 | DOWN |
| ENSG00000279059 | RP11-257O5.2 | -1.02768 | 0.001783 | 0.033065 | DOWN |
| ENSG00000279175 | CTA-414D7.1 | -1.00226 | 0.000148 | 0.008221 | DOWN |
| ENSG00000279184 | RP3-323A16.1 | -1.73416 | 0.000247 | 0.010993 | DOWN |
| ENSG00000279204 | RP11-175K6.2 | -1.59023 | 4.30E-08 | 8.05E-05 | DOWN |
| ENSG00000279325 | RP11-24H2.3 | -1.42592 | 8.86E-08 | 0.000122 | DOWN |
| ENSG00000279400 | CTD-2353F22.2 | -1.31593 | 4.62E-12 | 4.49E-07 | DOWN |
| ENSG00000271317 | IGHD4OR15-4A | -2.85455 | 3.73E-06 | 0.001018 | DOWN |
| ENSG00000279532 | CTB-96E2.6 | -1.36081 | 1.37E-06 | 0.000578 | DOWN |
| ENSG00000279561 | RP11-4L24.4 | -1.3481 | 1.11E-05 | 0.001904 | DOWN |
| ENSG00000279636 | LINC00216 | -1.4555 | 3.16E-08 | 6.78E-05 | DOWN |
| ENSG00000279675 | RP11-454H19.2 | -1.13854 | 1.36E-07 | 0.000155 | DOWN |
| ENSG00000279679 | RP11-278J6.5 | -1.59736 | 0.000628 | 0.018542 | DOWN |
| ENSG00000279734 | RP11-675P14.2 | -1.71025 | 7.49E-09 | 2.99E-05 | DOWN |
| ENSG00000279821 | RP11-1334A24.5 | -1.69233 | 1.15E-10 | 2.79E-06 | DOWN |
| ENSG00000279845 | RP11-6L6.4 | -2.45889 | 3.19E-15 | 7.38E-09 | DOWN |
| ENSG00000279917 | RP11-360N9.2 | -1.03653 | 2.41E-08 | 5.84E-05 | DOWN |
| ENSG00000280096 | RP11-294N21.2 | -1.91703 | 7.48E-09 | 2.98E-05 | DOWN |
| ENSG00000280272 | RP3-446N13.4 | -1.13932 | 2.62E-10 | 4.45E-06 | DOWN |
| ENSG00000237700 | PRAMEF33P | -1.15478 | 0.003458 | 0.047951 | DOWN |
| ENSG00000280326 | RP11-642A1.1 | -1.64667 | 7.27E-12 | 5.76E-07 | DOWN |
| ENSG00000280378 | RP4-758J24.6 | -1.80618 | 8.69E-06 | 0.001655 | DOWN |
| ENSG00000280752 | LINC00850 | -1.4792 | 2.71E-09 | 1.70E-05 | DOWN |
| ENSG00000280852 | CTD-2319I12.10 | -1.10786 | 0.000714 | 0.019882 | DOWN |
| ENSG00000281106 | LINC00282 | -1.15846 | 6.13E-06 | 0.00135 | DOWN |
| ENSG00000281406 | BLACAT1 | -1.9498 | 2.44E-08 | 5.87E-05 | DOWN |
| ENSG00000281469 | RP11-567F11.1 | -1.07933 | 0.000298 | 0.012209 | DOWN |
| ENSG00000281477 | RP11-223J6.2 | -1.7945 | 7.60E-07 | 0.000412 | DOWN |
| ENSG00000281732 | CTA-299D3.8 | -1.06689 | 0.003602 | 0.049042 | DOWN |
| ENSG00000282304 | RP11-1223D19.3 | -1.4186 | 0.002144 | 0.036621 | DOWN |
| ENSG00000282939 | TRBV7-2 | -1.17026 | 3.76E-05 | 0.003774 | DOWN |
| ENSG00000283118 | RP11-107E5.4 | -1.06376 | 2.09E-05 | 0.002714 | DOWN |
