# Supplementary material for: Targeting the lncRNA DUXAP8/miR-29a/PIK3CA Network Restores Doxorubicin Chemosensitivity via PI3K-AKT-mTOR Signaling and Synergizes With Inotuzumab Ozogamicin in Chemotherapy-Resistant B-Cell Acute Lymphoblastic Leukemia
Source: Front Oncol. 2022 Mar 2;12:773601. doi: 10.3389/fonc.2022.773601 (PMC8924619; doi:10.3389/fonc.2022.773601)
Supplement: Supplementary file 5 [file Table_2.docx]

**Supplementary Table 2.** Dysregulated miRNAs.

| Gene ID | Log_2_FoldChange | P | P_adj_ | Trend |
| --- | --- | --- | --- | --- |
| hsa-let-7a-5p | 2.616523 | 0.013131 | 0.079337 | UP |
| hsa-miR-199a-5p | 2.240786 | 0.023046 | 0.115071 | UP |
| hsa-miR-19a-3p | 4.508441 | 1.25E-07 | 7.50E-06 | UP |
| hsa-miR-17-5p | 3.836591 | 0.000711 | 0.009651 | UP |
| hsa-miR-361-3p | 3.454983 | 0.000176 | 0.003337 | UP |
| hsa-miR-18a-3p | 3.287695 | 0.000248 | 0.004355 | UP |
| hsa-miR-221-3p | 3.122777 | 0.002598 | 0.022699 | UP |
| hsa-miR-769-5p | 3.09049 | 2.56E-07 | 1.31E-05 | UP |
| hsa-let-7e-5p | 2.904624 | 0.001817 | 0.017419 | UP |
| hsa-miR-320a | 2.865718 | 0.00344 | 0.029095 | UP |
| hsa-miR-346 | 2.684241 | 0.000321 | 0.005371 | UP |
| hsa-miR-429 | 2.608166 | 4.32E-06 | 0.000141 | UP |
| hsa-miR-95-3p | 2.492561 | 7.70E-06 | 0.000241 | UP |
| hsa-miR-4272 | 2.422861 | 0.00112 | 0.014308 | UP |
| hsa-miR-204-5p | 2.416436 | 0.001225 | 0.015186 | UP |
| hsa-miR-16-5p | 2.349806 | 0.003986 | 0.032943 | UP |
| hsa-miR-124-3p | 2.32579 | 0.002037 | 0.018677 | UP |
| hsa-miR-142-3p | 2.28937 | 0.005249 | 0.03973 | UP |
| hsa-miR-6788-5p | 1.941192 | 0.008968 | 0.062604 | UP |
| hsa-miR-24-2-5p | 1.815906 | 0.034869 | 0.148349 | UP |
| hsa-miR-210-5p | 1.775259 | 0.000101 | 0.002081 | UP |
| hsa-miR-451a | 1.624491 | 0.001517 | 0.017047 | UP |
| hsa-miR-505-3p | 1.577886 | 1.13E-08 | 9.06E-07 | UP |
| hsa-miR-93-5p | 1.574669 | 0.026608 | 0.124772 | UP |
| hsa-miR-139-5p | 1.559386 | 6.15E-05 | 0.001383 | UP |
| hsa-miR-302b-3p | 1.550792 | 0.028889 | 0.129014 | UP |
| hsa-miR-218-5p | 1.371602 | 4.69E-07 | 2.11E-05 | UP |
| hsa-miR-181c-3p | 1.367975 | 0.008661 | 0.061052 | UP |
| hsa-miR-338-3p | 1.357851 | 1.36E-10 | 2.44E-08 | UP |
| hsa-miR-365a-3p | 1.352579 | 9.59E-05 | 0.002027 | UP |
| hsa-miR-215-5p | 1.30508 | 1.42E-07 | 7.84E-06 | UP |
| hsa-miR-210-3p | 1.304611 | 4.58E-09 | 4.70E-07 | UP |
| hsa-miR-633 | 1.245028 | 2.62E-06 | 9.42E-05 | UP |
| hsa-miR-549a | 1.242961 | 0.00693 | 0.050327 | UP |
| hsa-miR-874-3p | 1.233075 | 3.39E-06 | 0.000116 | UP |
| hsa-miR-302a-3p | 1.226869 | 8.70E-10 | 1.04E-07 | UP |
| hsa-miR-1-3p | 1.198261 | 0.010899 | 0.070597 | UP |
| hsa-miR-181c-5p | 1.189742 | 2.11E-06 | 8.00E-05 | UP |
| hsa-miR-26a-5p | 1.178198 | 0.000835 | 0.011113 | UP |
| hsa-miR-222-3p | 1.1128 | 0.008358 | 0.059497 | UP |
| hsa-miR-155-5p | 1.080357 | 3.27E-08 | 2.14E-06 | UP |
| hsa-miR-30b-5p | 1.077781 | 0.017254 | 0.097576 | UP |
| hsa-miR-24-3p | 1.071313 | 2.12E-05 | 0.000545 | UP |
| hsa-miR-106b-5p | 1.031778 | 4.41E-05 | 0.001023 | UP |
| hsa-miR-29b-3p | 1.019239 | 3.06E-10 | 4.41E-08 | UP |
| hsa-let-7c-5p | 1.003383 | 0.028153 | 0.127306 | UP |
| hsa-miR-652-3p | -1.01384 | 3.93E-05 | 0.000941 | DOWN |
| hsa-miR-484 | -1.02859 | 2.85E-07 | 1.37E-05 | DOWN |
| hsa-miR-6734-3p | -1.03138 | 0.009996 | 0.066158 | DOWN |
| hsa-miR-376a-3p | -1.04185 | 0.002365 | 0.021257 | DOWN |
| hsa-miR-101-3p | -1.14713 | 0.015975 | 0.091159 | DOWN |
| hsa-miR-99a-5p | -1.15861 | 0.000274 | 0.004695 | DOWN |
| hsa-miR-92a-3p | -1.1613 | 0.026326 | 0.124772 | DOWN |
| hsa-miR-296-3p | -1.18518 | 0.01308 | 0.079337 | DOWN |
| hsa-miR-98-5p | -1.19036 | 0.039594 | 0.15904 | DOWN |
| hsa-miR-130b-3p | -1.19726 | 0.000197 | 0.003628 | DOWN |
| hsa-miR-520h | -1.2387 | 0.015165 | 0.087934 | DOWN |
| hsa-miR-302c-3p | -1.31653 | 0.001695 | 0.017419 | DOWN |
| hsa-miR-423-3p | -1.44434 | 9.72E-09 | 8.74E-07 | DOWN |
| hsa-miR-615-3p | -1.4461 | 0.001379 | 0.016053 | DOWN |
| hsa-miR-4326 | -1.46851 | 0.042994 | 0.168003 | DOWN |
| hsa-miR-147a | -1.47761 | 1.13E-05 | 0.000324 | DOWN |
| hsa-miR-5582-5p | -1.47843 | 0.001011 | 0.01322 | DOWN |
| hsa-miR-1260b | -1.49005 | 0.006488 | 0.047601 | DOWN |
| hsa-miR-205-5p | -1.57867 | 0.014054 | 0.08351 | DOWN |
| hsa-miR-15b-5p | -1.62505 | 0.019392 | 0.10405 | DOWN |
| hsa-miR-28-5p | -1.63947 | 0.01992 | 0.104538 | DOWN |
| hsa-miR-122-5p | -1.78971 | 0.000589 | 0.008463 | DOWN |
| hsa-miR-200c-3p | -1.93731 | 0.009289 | 0.063114 | DOWN |
| hsa-miR-125a-5p | -1.95139 | 1.53E-06 | 6.49E-05 | DOWN |
| hsa-miR-182-5p | -2.13125 | 0.013343 | 0.079945 | DOWN |
| hsa-miR-4669 | -2.51951 | 0.015381 | 0.08847 | DOWN |
| hsa-miR-29a-3p | -2.5604 | 0.001384 | 0.016053 | DOWN |
| hsa-miR-181d-5p | -2.64991 | 0.000586 | 0.008463 | DOWN |
| hsa-miR-15a-5p | -3.19923 | 0.001714 | 0.017419 | DOWN |
