# Supplementary material for: Targeting the lncRNA DUXAP8/miR-29a/PIK3CA Network Restores Doxorubicin Chemosensitivity via PI3K-AKT-mTOR Signaling and Synergizes With Inotuzumab Ozogamicin in Chemotherapy-Resistant B-Cell Acute Lymphoblastic Leukemia
Source: Front Oncol. 2022 Mar 2;12:773601. doi: 10.3389/fonc.2022.773601 (PMC8924619; doi:10.3389/fonc.2022.773601)
Supplement: Supplementary file 6 [file Table_3.docx]

**Supplementary Table 3.** Dysregulated mRNAs.

| Gene ID | Gene Symbol | Log_2_FoldChange | P | P_adj_ | Trend |
| --- | --- | --- | --- | --- | --- |
| ENSG00000157106 | SMG1 | 3.368881 | 2.94E-12 | 3.48E-07 | UP |
| ENSG00000136869 | TLR4 | 2.877979 | 1.16E-10 | 2.79E-06 | UP |
| ENSG00000112981 | NME5 | 2.854957 | 0.000662 | 0.019056 | UP |
| ENSG00000184956 | MUC6 | 2.839585 | 5.24E-09 | 2.43E-05 | UP |
| ENSG00000165548 | TMEM63C | 2.774112 | 1.62E-08 | 4.63E-05 | UP |
| ENSG00000008517 | IL32 | 2.575692 | 4.84E-06 | 0.001178 | UP |
| ENSG00000134317 | GRHL1 | 2.522134 | 5.92E-07 | 0.000356 | UP |
| ENSG00000229833 | PET100 | 2.478041 | 1.10E-06 | 0.000509 | UP |
| ENSG00000114738 | MAPKAPK3 | 2.473216 | 2.49E-07 | 0.000219 | UP |
| ENSG00000257923 | CUX1 | 2.455492 | 1.10E-07 | 0.000138 | UP |
| ENSG00000139194 | RBP5 | 2.436508 | 5.64E-14 | 3.94E-08 | UP |
| ENSG00000141505 | ASGR1 | 2.433181 | 3.61E-08 | 7.32E-05 | UP |
| ENSG00000197119 | SLC25A29 | 2.389127 | 2.11E-10 | 3.94E-06 | UP |
| ENSG00000187553 | CYP26C1 | 2.350354 | 4.01E-07 | 0.000287 | UP |
| ENSG00000010327 | STAB1 | 2.32605 | 3.60E-15 | 7.88E-09 | UP |
| ENSG00000115942 | ORC2 | 2.300643 | 2.79E-08 | 6.30E-05 | UP |
| ENSG00000102524 | TNFSF13B | 2.218414 | 0.000132 | 0.007712 | UP |
| ENSG00000128218 | VPREB3 | 2.215159 | 4.07E-09 | 2.13E-05 | UP |
| ENSG00000183837 | PNMA3 | 2.186059 | 0.00317 | 0.045642 | UP |
| ENSG00000149600 | COMMD7 | 2.167999 | 6.74E-09 | 2.81E-05 | UP |
| ENSG00000280151 | AC026248.1 | 2.156398 | 1.57E-06 | 0.000625 | UP |
| ENSG00000099949 | LZTR1 | 2.137805 | 7.57E-09 | 3.00E-05 | UP |
| ENSG00000157020 | SEC13 | 2.132793 | 5.07E-08 | 8.84E-05 | UP |
| ENSG00000131482 | G6PC | 2.092815 | 8.85E-09 | 3.26E-05 | UP |
| ENSG00000221855 | TAS2R41 | 2.080818 | 0.000471 | 0.015759 | UP |
| ENSG00000141579 | ZNF750 | 2.071816 | 2.03E-05 | 0.002671 | UP |
| ENSG00000119772 | DNMT3A | 2.063927 | 0.001061 | 0.024834 | UP |
| ENSG00000185070 | FLRT2 | 2.039329 | 2.58E-10 | 4.41E-06 | UP |
| ENSG00000114767 | RRP9 | 2.033723 | 1.38E-07 | 0.000157 | UP |
| ENSG00000158023 | WDR66 | 2.021269 | 0.000817 | 0.021475 | UP |
| ENSG00000169032 | MAP2K1 | 2.018482 | 7.84E-07 | 0.000419 | UP |
| ENSG00000128271 | ADORA2A | 2.017919 | 2.15E-05 | 0.002758 | UP |
| ENSG00000111790 | FGFR1OP2 | 2.017376 | 1.34E-05 | 0.002115 | UP |
| ENSG00000187796 | CARD9 | 2.015664 | 2.67E-12 | 3.30E-07 | UP |
| ENSG00000168131 | OR2B2 | 2.014634 | 2.35E-06 | 0.000782 | UP |
| ENSG00000270672 | MTRNR2L6 | 2.010575 | 6.38E-11 | 1.98E-06 | UP |
| ENSG00000139323 | POC1B | 1.994394 | 4.53E-07 | 0.000308 | UP |
| ENSG00000039523 | FAM65A | 1.988493 | 0.000103 | 0.006716 | UP |
| ENSG00000131650 | KREMEN2 | 1.981153 | 5.85E-05 | 0.004852 | UP |
| ENSG00000122862 | SRGN | 1.962604 | 6.15E-06 | 0.001352 | UP |
| ENSG00000182718 | ANXA2 | 1.952475 | 0.000102 | 0.006694 | UP |
| ENSG00000166436 | TRIM66 | 1.949753 | 6.78E-12 | 5.55E-07 | UP |
| ENSG00000143382 | ADAMTSL4 | 1.94889 | 8.10E-14 | 4.77E-08 | UP |
| ENSG00000049089 | COL9A2 | 1.942653 | 4.04E-07 | 0.000288 | UP |
| ENSG00000154274 | C4orf19 | 1.934672 | 3.60E-06 | 0.000999 | UP |
| ENSG00000177570 | SAMD12 | 1.932539 | 0.000143 | 0.008057 | UP |
| ENSG00000166833 | NAV2 | 1.927545 | 2.73E-06 | 0.000852 | UP |
| ENSG00000158748 | HTR6 | 1.924181 | 0.002581 | 0.040652 | UP |
| ENSG00000177169 | ULK1 | 1.922441 | 1.47E-05 | 0.002229 | UP |
| ENSG00000178021 | TSPYL6 | 1.921256 | 0.000135 | 0.007788 | UP |
| ENSG00000240053 | LY6G5B | 1.920393 | 0.000125 | 0.007476 | UP |
| ENSG00000151650 | VENTX | 1.909557 | 1.23E-09 | 1.08E-05 | UP |
| ENSG00000126895 | AVPR2 | 1.896487 | 2.08E-10 | 3.91E-06 | UP |
| ENSG00000137809 | ITGA11 | 1.892887 | 1.16E-06 | 0.000525 | UP |
| ENSG00000160781 | PAQR6 | 1.865778 | 3.42E-05 | 0.003574 | UP |
| ENSG00000213658 | LAT | 1.863842 | 2.92E-06 | 0.000887 | UP |
| ENSG00000165457 | FOLR2 | 1.861954 | 0.000502 | 0.016346 | UP |
| ENSG00000119048 | UBE2B | 1.835991 | 6.77E-11 | 2.05E-06 | UP |
| ENSG00000182253 | SYNM | 1.823093 | 0.000164 | 0.008732 | UP |
| ENSG00000162409 | PRKAA2 | 1.819944 | 6.11E-08 | 9.87E-05 | UP |
| ENSG00000101812 | H2BFM | 1.815211 | 1.73E-05 | 0.002439 | UP |
| ENSG00000170049 | KCNAB3 | 1.814599 | 0.000407 | 0.014545 | UP |
| ENSG00000107281 | NPDC1 | 1.805902 | 0.000626 | 0.018523 | UP |
| ENSG00000126464 | PRR12 | 1.803643 | 1.25E-05 | 0.002038 | UP |
| ENSG00000146063 | TRIM41 | 1.802163 | 3.78E-07 | 0.000278 | UP |
| ENSG00000178623 | GPR35 | 1.801522 | 1.02E-08 | 3.54E-05 | UP |
| ENSG00000167615 | LENG8 | 1.796476 | 9.06E-09 | 3.30E-05 | UP |
| ENSG00000258539 | RP11-12J10.3 | 1.796092 | 0.000257 | 0.011234 | UP |
| ENSG00000142731 | PLK4 | 1.795198 | 9.49E-05 | 0.006417 | UP |
| ENSG00000261832 | RP11-435I10.4 | 1.79473 | 1.12E-05 | 0.001915 | UP |
| ENSG00000107859 | PITX3 | 1.787148 | 1.44E-08 | 4.32E-05 | UP |
| ENSG00000169083 | AR | 1.775072 | 2.13E-07 | 0.000202 | UP |
| ENSG00000130653 | PNPLA7 | 1.770888 | 2.00E-09 | 1.43E-05 | UP |
| ENSG00000160752 | FDPS | 1.762706 | 8.86E-09 | 3.26E-05 | UP |
| ENSG00000197122 | SRC | 1.761969 | 1.06E-05 | 0.001861 | UP |
| ENSG00000197461 | PDGFA | 1.755884 | 0.000214 | 0.010143 | UP |
| ENSG00000176533 | GNG7 | 1.753728 | 7.30E-05 | 0.005511 | UP |
| ENSG00000164253 | WDR41 | 1.738422 | 0.000143 | 0.008057 | UP |
| ENSG00000112576 | CCND3 | 1.738359 | 5.78E-08 | 9.55E-05 | UP |
| ENSG00000183044 | ABAT | 1.737348 | 0.000218 | 0.010267 | UP |
| ENSG00000053747 | LAMA3 | 1.735147 | 0.003022 | 0.044417 | UP |
| ENSG00000158636 | C11orf30 | 1.727215 | 0.000101 | 0.00664 | UP |
| ENSG00000134072 | CAMK1 | 1.727181 | 7.08E-06 | 0.001465 | UP |
| ENSG00000185101 | ANO9 | 1.726965 | 5.75E-09 | 2.56E-05 | UP |
| ENSG00000178038 | ALS2CL | 1.720016 | 1.00E-10 | 2.57E-06 | UP |
| ENSG00000144130 | NT5DC4 | 1.719982 | 0.000713 | 0.019871 | UP |
| ENSG00000077238 | IL4R | 1.71675 | 0.000299 | 0.012229 | UP |
| ENSG00000196126 | HLA-DRB1 | 1.716232 | 4.99E-07 | 0.000325 | UP |
| ENSG00000023445 | BIRC3 | 1.715708 | 7.77E-12 | 5.95E-07 | UP |
| ENSG00000103056 | SMPD3 | 1.708517 | 1.12E-08 | 3.73E-05 | UP |
| ENSG00000215529 | EFCAB8 | 1.705543 | 3.17E-06 | 0.000928 | UP |
| ENSG00000280080 | U51561.1 | 1.705144 | 6.06E-11 | 1.92E-06 | UP |
| ENSG00000181830 | SLC35C1 | 1.699851 | 0.000708 | 0.019802 | UP |
| ENSG00000120049 | KCNIP2 | 1.697981 | 9.23E-05 | 0.006315 | UP |
| ENSG00000109685 | WHSC1 | 1.696826 | 6.19E-09 | 2.68E-05 | UP |
| ENSG00000084754 | HADHA | 1.69156 | 1.29E-12 | 2.21E-07 | UP |
| ENSG00000100288 | CHKB | 1.690619 | 0.000376 | 0.013895 | UP |
| ENSG00000124766 | SOX4 | 1.690618 | 3.28E-14 | 2.87E-08 | UP |
| ENSG00000188153 | COL4A5 | 1.68199 | 5.93E-07 | 0.000357 | UP |
| ENSG00000164050 | PLXNB1 | 1.680772 | 0.000314 | 0.012572 | UP |
| ENSG00000074964 | ARHGEF10L | 1.679907 | 3.89E-09 | 2.08E-05 | UP |
| ENSG00000174233 | ADCY6 | 1.672089 | 0.00022 | 0.010295 | UP |
| ENSG00000178404 | CEP295NL | 1.668368 | 1.58E-13 | 6.86E-08 | UP |
| ENSG00000154764 | WNT7A | 1.667081 | 0.002548 | 0.040395 | UP |
| ENSG00000189056 | RELN | 1.662626 | 6.10E-05 | 0.004969 | UP |
| ENSG00000080854 | IGSF9B | 1.660507 | 1.62E-05 | 0.002353 | UP |
| ENSG00000122126 | OCRL | 1.660395 | 1.48E-05 | 0.002239 | UP |
| ENSG00000116032 | GRIN3B | 1.657625 | 0.00018 | 0.009197 | UP |
| ENSG00000255587 | RAB44 | 1.655494 | 5.81E-09 | 2.57E-05 | UP |
| ENSG00000180357 | ZNF609 | 1.655175 | 0.000244 | 0.010904 | UP |
| ENSG00000173482 | PTPRM | 1.65203 | 3.29E-05 | 0.003498 | UP |
| ENSG00000174945 | AMZ1 | 1.62512 | 1.46E-05 | 0.002224 | UP |
| ENSG00000141837 | CACNA1A | 1.620829 | 0.000691 | 0.019524 | UP |
| ENSG00000083720 | OXCT1 | 1.617005 | 0.000545 | 0.017159 | UP |
| ENSG00000179256 | SMCO3 | 1.615198 | 6.91E-05 | 0.005343 | UP |
| ENSG00000121879 | PIK3CA | 1.605892 | 3.18E-12 | 3.63E-07 | UP |
| ENSG00000163467 | TSACC | 1.605036 | 1.20E-05 | 0.001988 | UP |
| ENSG00000165424 | ZCCHC24 | 1.604838 | 1.11E-05 | 0.001901 | UP |
| ENSG00000176495 | OR5AN1 | 1.598583 | 1.63E-08 | 4.63E-05 | UP |
| ENSG00000128052 | KDR | 1.593804 | 1.64E-06 | 0.00064 | UP |
| ENSG00000147465 | STAR | 1.588386 | 0.000329 | 0.012915 | UP |
| ENSG00000214688 | C10orf105 | 1.587911 | 8.36E-07 | 0.000436 | UP |
| ENSG00000008710 | PKD1 | 1.587675 | 5.64E-11 | 1.85E-06 | UP |
| ENSG00000164171 | ITGA2 | 1.586363 | 0.000207 | 0.009971 | UP |
| ENSG00000141458 | NPC1 | 1.585419 | 0.002401 | 0.039075 | UP |
| ENSG00000243696 | RP5-966M1.6 | 1.584018 | 0.000579 | 0.017737 | UP |
| ENSG00000119535 | CSF3R | 1.57977 | 7.62E-08 | 0.000112 | UP |
| ENSG00000161642 | ZNF385A | 1.576486 | 9.72E-07 | 0.000475 | UP |
| ENSG00000100376 | FAM118A | 1.571592 | 3.37E-05 | 0.003547 | UP |
| ENSG00000259522 | RP11-468E2.2 | 1.569972 | 8.13E-09 | 3.12E-05 | UP |
| ENSG00000182580 | EPHB3 | 1.56753 | 0.00119 | 0.02645 | UP |
| ENSG00000174038 | C9orf131 | 1.558101 | 4.63E-07 | 0.000312 | UP |
| ENSG00000164093 | PITX2 | 1.556051 | 0.002461 | 0.039629 | UP |
| ENSG00000171793 | CTPS1 | 1.554539 | 3.87E-05 | 0.003828 | UP |
| ENSG00000118260 | CREB1 | 1.552669 | 2.12E-05 | 0.002735 | UP |
| ENSG00000176182 | MYPOP | 1.552502 | 0.000221 | 0.01032 | UP |
| ENSG00000180815 | MAP3K15 | 1.55097 | 1.68E-05 | 0.002406 | UP |
| ENSG00000147606 | SLC26A7 | 1.550948 | 0.000284 | 0.011888 | UP |
| ENSG00000130511 | SSBP4 | 1.550305 | 3.35E-08 | 7.04E-05 | UP |
| ENSG00000131398 | KCNC3 | 1.547106 | 3.83E-05 | 0.003811 | UP |
| ENSG00000185386 | MAPK11 | 1.54697 | 0.000168 | 0.00887 | UP |
| ENSG00000151923 | TIAL1 | 1.545491 | 5.78E-12 | 5.10E-07 | UP |
| ENSG00000186919 | ZACN | 1.545192 | 6.61E-06 | 0.00141 | UP |
| ENSG00000168071 | CCDC88B | 1.542978 | 1.23E-09 | 1.08E-05 | UP |
| ENSG00000103168 | TAF1C | 1.542687 | 1.07E-09 | 1.00E-05 | UP |
| ENSG00000100883 | SRP54 | 1.540334 | 3.13E-09 | 1.85E-05 | UP |
| ENSG00000188996 | HUS1B | 1.539965 | 0.000338 | 0.013087 | UP |
| ENSG00000168116 | KIAA1586 | 1.539327 | 1.05E-09 | 9.94E-06 | UP |
| ENSG00000134780 | DAGLA | 1.536496 | 0.001049 | 0.024663 | UP |
| ENSG00000117983 | MUC5B | 1.535213 | 0.001114 | 0.025529 | UP |
| ENSG00000166526 | ZNF3 | 1.534273 | 2.28E-05 | 0.002851 | UP |
| ENSG00000266714 | MYO15B | 1.530848 | 4.73E-10 | 6.29E-06 | UP |
| ENSG00000153885 | KCTD15 | 1.529027 | 2.86E-09 | 1.75E-05 | UP |
| ENSG00000203896 | LIME1 | 1.527833 | 3.03E-06 | 0.000906 | UP |
| ENSG00000151694 | ADAM17 | 1.525873 | 0.001243 | 0.027093 | UP |
| ENSG00000162390 | ACOT11 | 1.52388 | 4.88E-07 | 0.000321 | UP |
| ENSG00000166741 | NNMT | 1.522759 | 4.13E-05 | 0.003979 | UP |
| ENSG00000187695 | RP11-723O4.6 | 1.51949 | 3.61E-06 | 0.000999 | UP |
| ENSG00000100596 | SPTLC2 | 1.515339 | 2.66E-14 | 2.52E-08 | UP |
| ENSG00000131558 | EXOC4 | 1.512646 | 8.26E-10 | 8.72E-06 | UP |
| ENSG00000184983 | NDUFA6 | 1.512559 | 2.51E-05 | 0.00301 | UP |
| ENSG00000119771 | KLHL29 | 1.510792 | 1.69E-07 | 0.000177 | UP |
| ENSG00000171368 | TPPP | 1.508101 | 2.44E-06 | 0.000799 | UP |
| ENSG00000173452 | TMEM196 | 1.507666 | 1.11E-08 | 3.72E-05 | UP |
| ENSG00000049768 | FOXP3 | 1.507203 | 0.000575 | 0.017681 | UP |
| ENSG00000100285 | NEFH | 1.506157 | 0.002209 | 0.037275 | UP |
| ENSG00000177873 | ZNF619 | 1.50609 | 0.000564 | 0.017491 | UP |
| ENSG00000179832 | MROH1 | 1.505581 | 1.33E-11 | 8.04E-07 | UP |
| ENSG00000232810 | TNF | 1.505013 | 7.43E-06 | 0.001509 | UP |
| ENSG00000105711 | SCN1B | 1.502823 | 0.000302 | 0.012301 | UP |
| ENSG00000176153 | GPX2 | 1.502729 | 3.95E-07 | 0.000284 | UP |
| ENSG00000139344 | AMDHD1 | 1.499322 | 9.37E-05 | 0.006367 | UP |
| ENSG00000186174 | BCL9L | 1.498194 | 2.06E-06 | 0.000727 | UP |
| ENSG00000148935 | GAS2 | 1.496387 | 5.45E-09 | 2.49E-05 | UP |
| ENSG00000120253 | NUP43 | 1.49498 | 1.84E-06 | 0.000682 | UP |
| ENSG00000185899 | TAS2R60 | 1.494904 | 0.000122 | 0.007389 | UP |
| ENSG00000117308 | GALE | 1.49396 | 2.53E-06 | 0.000815 | UP |
| ENSG00000157992 | KRTCAP3 | 1.493814 | 0.000742 | 0.020326 | UP |
| ENSG00000075303 | SLC25A40 | 1.493633 | 0.000295 | 0.012153 | UP |
| ENSG00000116824 | CD2 | 1.49197 | 1.83E-05 | 0.002517 | UP |
| ENSG00000164916 | FOXK1 | 1.491863 | 3.90E-08 | 7.62E-05 | UP |
| ENSG00000271383 | NBPF19 | 1.49166 | 0.000696 | 0.019596 | UP |
| ENSG00000070669 | ASNS | 1.491233 | 0.000417 | 0.014754 | UP |
| ENSG00000180964 | TCEAL8 | 1.491018 | 2.28E-05 | 0.002852 | UP |
| ENSG00000250510 | GPR162 | 1.483391 | 3.69E-07 | 0.000274 | UP |
| ENSG00000105963 | ADAP1 | 1.480375 | 3.79E-07 | 0.000278 | UP |
| ENSG00000161980 | POLR3K | 1.479876 | 3.69E-07 | 0.000274 | UP |
| ENSG00000143434 | SEMA6C | 1.477529 | 5.63E-05 | 0.004751 | UP |
| ENSG00000155265 | GOLGA7B | 1.477041 | 1.77E-05 | 0.002475 | UP |
| ENSG00000169174 | PCSK9 | 1.476634 | 5.64E-09 | 2.54E-05 | UP |
| ENSG00000203485 | INF2 | 1.475728 | 4.19E-07 | 0.000295 | UP |
| ENSG00000109072 | VTN | 1.475262 | 0.000547 | 0.017187 | UP |
| ENSG00000144152 | FBLN7 | 1.474044 | 9.71E-06 | 0.001765 | UP |
| ENSG00000100726 | TELO2 | 1.472539 | 3.85E-07 | 0.00028 | UP |
| ENSG00000149639 | SOGA1 | 1.471263 | 7.71E-07 | 0.000416 | UP |
| ENSG00000168874 | ATOH8 | 1.470423 | 0.000841 | 0.021816 | UP |
| ENSG00000122203 | KIAA1191 | 1.470281 | 8.78E-06 | 0.001666 | UP |
| ENSG00000187498 | COL4A1 | 1.470114 | 0.001523 | 0.030336 | UP |
| ENSG00000004399 | PLXND1 | 1.468588 | 1.52E-08 | 4.45E-05 | UP |
| ENSG00000130254 | SAFB2 | 1.468474 | 0.000192 | 0.009552 | UP |
| ENSG00000258839 | MC1R | 1.466519 | 7.49E-05 | 0.005588 | UP |
| ENSG00000183773 | AIFM3 | 1.465141 | 7.19E-05 | 0.005463 | UP |
| ENSG00000144331 | ZNF385B | 1.462296 | 0.001169 | 0.026204 | UP |
| ENSG00000243716 | NPIPB5 | 1.460183 | 7.88E-07 | 0.000421 | UP |
| ENSG00000160193 | WDR4 | 1.458957 | 2.03E-09 | 1.44E-05 | UP |
| ENSG00000236737 | GAGE12B | 1.458426 | 4.91E-06 | 0.001188 | UP |
| ENSG00000156427 | FGF18 | 1.45752 | 0.001408 | 0.02905 | UP |
| ENSG00000179532 | DNHD1 | 1.456109 | 1.73E-13 | 7.27E-08 | UP |
| ENSG00000168546 | GFRA2 | 1.452187 | 0.000554 | 0.017321 | UP |
| ENSG00000198691 | ABCA4 | 1.45208 | 4.63E-09 | 2.27E-05 | UP |
| ENSG00000132003 | ZSWIM4 | 1.451101 | 1.11E-07 | 0.000139 | UP |
| ENSG00000169710 | FASN | 1.45075 | 2.96E-06 | 0.000894 | UP |
| ENSG00000140093 | SERPINA10 | 1.450363 | 0.000648 | 0.01883 | UP |
| ENSG00000171208 | NETO2 | 1.450108 | 1.15E-08 | 3.80E-05 | UP |
| ENSG00000148840 | PPRC1 | 1.449135 | 0.000104 | 0.006756 | UP |
| ENSG00000188152 | NUTM2G | 1.448996 | 1.00E-06 | 0.000483 | UP |
| ENSG00000117115 | PADI2 | 1.448818 | 4.29E-05 | 0.004064 | UP |
| ENSG00000140365 | COMMD4 | 1.448093 | 0.002748 | 0.042123 | UP |
| ENSG00000165138 | ANKS6 | 1.447818 | 2.77E-12 | 3.35E-07 | UP |
| ENSG00000136859 | ANGPTL2 | 1.447539 | 6.11E-07 | 0.000364 | UP |
| ENSG00000130827 | PLXNA3 | 1.447063 | 1.12E-06 | 0.000515 | UP |
| ENSG00000173611 | SCAI | 1.444912 | 0.002755 | 0.04218 | UP |
| ENSG00000275342 | SGK223 | 1.443577 | 2.68E-10 | 4.50E-06 | UP |
| ENSG00000095209 | TMEM38B | 1.443504 | 8.45E-05 | 0.006005 | UP |
| ENSG00000183087 | GAS6 | 1.443188 | 8.57E-09 | 3.21E-05 | UP |
| ENSG00000076928 | ARHGEF1 | 1.443161 | 6.70E-11 | 2.04E-06 | UP |
| ENSG00000136997 | MYC | 1.441594 | 0.000113 | 0.007075 | UP |
| ENSG00000141252 | VPS53 | 1.439876 | 2.17E-06 | 0.000748 | UP |
| ENSG00000134955 | SLC37A2 | 1.439481 | 3.13E-08 | 6.75E-05 | UP |
| ENSG00000163945 | UVSSA | 1.439408 | 1.39E-12 | 2.28E-07 | UP |
| ENSG00000169228 | RAB24 | 1.439326 | 2.88E-06 | 0.000878 | UP |
| ENSG00000188064 | WNT7B | 1.431699 | 1.88E-06 | 0.00069 | UP |
| ENSG00000140836 | ZFHX3 | 1.429874 | 0.000218 | 0.010262 | UP |
| ENSG00000180921 | FAM83H | 1.429854 | 0.000254 | 0.011173 | UP |
| ENSG00000179046 | TRIML2 | 1.428458 | 2.00E-06 | 0.000714 | UP |
| ENSG00000010810 | FYN | 1.428222 | 0.000624 | 0.018487 | UP |
| ENSG00000176253 | OR4K13 | 1.427642 | 4.91E-08 | 8.69E-05 | UP |
| ENSG00000233954 | UQCRHL | 1.426651 | 0.000584 | 0.017806 | UP |
| ENSG00000111886 | GABRR2 | 1.42613 | 5.86E-05 | 0.004855 | UP |
| ENSG00000133105 | RXFP2 | 1.425787 | 0.000177 | 0.009138 | UP |
| ENSG00000090006 | LTBP4 | 1.425779 | 4.63E-06 | 0.001149 | UP |
| ENSG00000161036 | LRWD1 | 1.425558 | 5.66E-09 | 2.54E-05 | UP |
| ENSG00000100650 | SRSF5 | 1.425191 | 0.000644 | 0.018777 | UP |
| ENSG00000135953 | MFSD9 | 1.423429 | 5.53E-08 | 9.29E-05 | UP |
| ENSG00000135835 | KIAA1614 | 1.42294 | 3.68E-05 | 0.003729 | UP |
| ENSG00000181963 | OR52K2 | 1.422723 | 4.03E-12 | 4.14E-07 | UP |
| ENSG00000134812 | GIF | 1.418191 | 6.73E-06 | 0.001424 | UP |
| ENSG00000198930 | CSAG1 | 1.41553 | 0.000613 | 0.018295 | UP |
| ENSG00000186866 | POFUT2 | 1.4153 | 3.24E-07 | 0.000254 | UP |
| ENSG00000261732 | LA16c-431H6.6 | 1.412223 | 1.03E-11 | 6.94E-07 | UP |
| ENSG00000204520 | MICA | 1.411601 | 3.40E-06 | 0.000967 | UP |
| ENSG00000187122 | SLIT1 | 1.410478 | 3.65E-06 | 0.001007 | UP |
| ENSG00000261150 | EPPK1 | 1.410292 | 2.11E-10 | 3.94E-06 | UP |
| ENSG00000147896 | IFNK | 1.408264 | 0.000153 | 0.008388 | UP |
| ENSG00000151304 | SRFBP1 | 1.407379 | 9.94E-08 | 0.000131 | UP |
| ENSG00000140807 | NKD1 | 1.405132 | 3.67E-09 | 2.02E-05 | UP |
| ENSG00000186766 | FOXI2 | 1.404192 | 3.76E-11 | 1.45E-06 | UP |
| ENSG00000114200 | BCHE | 1.398888 | 1.62E-06 | 0.000636 | UP |
| ENSG00000141519 | CCDC40 | 1.39887 | 0.002104 | 0.036237 | UP |
| ENSG00000159199 | ATP5G1 | 1.398835 | 0.002826 | 0.042792 | UP |
| ENSG00000183530 | PRR14L | 1.396291 | 9.67E-07 | 0.000473 | UP |
| ENSG00000140400 | MAN2C1 | 1.395823 | 1.59E-09 | 1.26E-05 | UP |
| ENSG00000177189 | RPS6KA3 | 1.395095 | 4.72E-07 | 0.000315 | UP |
| ENSG00000138435 | CHRNA1 | 1.394962 | 7.75E-05 | 0.005706 | UP |
| ENSG00000160972 | PPP1R16A | 1.393348 | 0.000245 | 0.01093 | UP |
| ENSG00000075826 | SEC31B | 1.389301 | 1.10E-06 | 0.000511 | UP |
| ENSG00000075399 | VPS9D1 | 1.38853 | 2.65E-05 | 0.003103 | UP |
| ENSG00000071242 | RPS6KA2 | 1.386912 | 1.52E-10 | 3.26E-06 | UP |
| ENSG00000188747 | NOXA1 | 1.38678 | 4.64E-07 | 0.000312 | UP |
| ENSG00000008394 | MGST1 | 1.382886 | 3.21E-05 | 0.003455 | UP |
| ENSG00000065989 | PDE4A | 1.38068 | 0.001272 | 0.027433 | UP |
| ENSG00000019485 | PRDM11 | 1.379021 | 0.002226 | 0.037445 | UP |
| ENSG00000145335 | SNCA | 1.378734 | 0.000297 | 0.012201 | UP |
| ENSG00000063169 | GLTSCR1 | 1.37827 | 0.000281 | 0.011804 | UP |
| ENSG00000079337 | RAPGEF3 | 1.377046 | 3.78E-08 | 7.50E-05 | UP |
| ENSG00000131969 | ABHD12B | 1.375086 | 3.46E-05 | 0.003601 | UP |
| ENSG00000185324 | CDK10 | 1.372069 | 1.05E-10 | 2.65E-06 | UP |
| ENSG00000150594 | ADRA2A | 1.371905 | 0.000462 | 0.015596 | UP |
| ENSG00000153531 | ADPRHL1 | 1.371905 | 0.000961 | 0.023485 | UP |
| ENSG00000125247 | TMTC4 | 1.370737 | 1.46E-09 | 1.20E-05 | UP |
| ENSG00000136295 | TTYH3 | 1.370322 | 6.60E-06 | 0.00141 | UP |
| ENSG00000175766 | EIF4E1B | 1.369505 | 4.32E-09 | 2.20E-05 | UP |
| ENSG00000150967 | ABCB9 | 1.368709 | 0.002077 | 0.035975 | UP |
| ENSG00000121764 | HCRTR1 | 1.368668 | 0.000174 | 0.00903 | UP |
| ENSG00000148400 | NOTCH1 | 1.367157 | 2.19E-08 | 5.53E-05 | UP |
| ENSG00000212935 | KRTAP10-3 | 1.363907 | 0.000537 | 0.017016 | UP |
| ENSG00000181652 | ATG9B | 1.36228 | 0.001392 | 0.028867 | UP |
| ENSG00000205765 | C5orf51 | 1.362212 | 1.60E-06 | 0.000631 | UP |
| ENSG00000197774 | EME2 | 1.361629 | 4.53E-07 | 0.000308 | UP |
| ENSG00000146830 | GIGYF1 | 1.360883 | 8.85E-14 | 4.89E-08 | UP |
| ENSG00000175471 | MCTP1 | 1.358921 | 2.91E-06 | 0.000885 | UP |
| ENSG00000173421 | CCDC36 | 1.357635 | 7.14E-05 | 0.005437 | UP |
| ENSG00000117266 | CDK18 | 1.356839 | 0.001502 | 0.030083 | UP |
| ENSG00000177595 | PIDD1 | 1.355647 | 2.61E-05 | 0.003073 | UP |
| ENSG00000197046 | SIGLEC15 | 1.354509 | 4.46E-05 | 0.004153 | UP |
| ENSG00000219435 | TEX40 | 1.354057 | 7.01E-13 | 1.54E-07 | UP |
| ENSG00000174898 | CATSPERD | 1.350504 | 3.31E-06 | 0.000952 | UP |
| ENSG00000171552 | BCL2L1 | 1.348773 | 0.002638 | 0.041157 | UP |
| ENSG00000213949 | ITGA1 | 1.348688 | 0.00065 | 0.018863 | UP |
| ENSG00000100299 | ARSA | 1.348287 | 1.15E-05 | 0.001939 | UP |
| ENSG00000108578 | BLMH | 1.347986 | 0.001647 | 0.031681 | UP |
| ENSG00000117676 | RPS6KA1 | 1.347627 | 2.43E-05 | 0.002951 | UP |
| ENSG00000134259 | NGF | 1.346194 | 1.25E-07 | 0.000148 | UP |
| ENSG00000167874 | TMEM88 | 1.34546 | 0.000387 | 0.014133 | UP |
| ENSG00000070614 | NDST1 | 1.344272 | 6.79E-10 | 7.79E-06 | UP |
| ENSG00000089820 | ARHGAP4 | 1.33994 | 4.34E-08 | 8.08E-05 | UP |
| ENSG00000122068 | FYTTD1 | 1.331938 | 3.03E-05 | 0.003344 | UP |
| ENSG00000107290 | SETX | 1.331458 | 2.44E-09 | 1.60E-05 | UP |
| ENSG00000135519 | KCNH3 | 1.331446 | 0.000129 | 0.007629 | UP |
| ENSG00000167548 | KMT2D | 1.330579 | 1.78E-08 | 4.89E-05 | UP |
| ENSG00000005961 | ITGA2B | 1.33027 | 4.12E-08 | 7.84E-05 | UP |
| ENSG00000168032 | ENTPD3 | 1.329841 | 0.001665 | 0.031873 | UP |
| ENSG00000175711 | B3GNTL1 | 1.329827 | 3.47E-07 | 0.000265 | UP |
| ENSG00000163145 | C1QTNF7 | 1.328402 | 0.001585 | 0.031034 | UP |
| ENSG00000118777 | ABCG2 | 1.326014 | 3.80E-05 | 0.003797 | UP |
| ENSG00000139835 | GRTP1 | 1.325582 | 0.000463 | 0.015605 | UP |
| ENSG00000130950 | NUTM2F | 1.32555 | 7.57E-08 | 0.000112 | UP |
| ENSG00000188603 | CLN3 | 1.3252 | 1.67E-06 | 0.000645 | UP |
| ENSG00000271425 | NBPF10 | 1.323976 | 1.78E-05 | 0.002477 | UP |
| ENSG00000177706 | FAM20C | 1.323717 | 5.54E-05 | 0.004713 | UP |
| ENSG00000261594 | TPBGL | 1.323403 | 0.000878 | 0.022323 | UP |
| ENSG00000180758 | GPR157 | 1.322789 | 0.001166 | 0.026174 | UP |
| ENSG00000144589 | STK11IP | 1.320009 | 4.59E-10 | 6.22E-06 | UP |
| ENSG00000138892 | TTLL8 | 1.316367 | 0.002768 | 0.042295 | UP |
| ENSG00000126215 | XRCC3 | 1.315298 | 3.41E-09 | 1.94E-05 | UP |
| ENSG00000143801 | PSEN2 | 1.314702 | 9.51E-06 | 0.001743 | UP |
| ENSG00000079432 | CIC | 1.312758 | 3.85E-06 | 0.001034 | UP |
| ENSG00000185056 | C5orf47 | 1.311523 | 7.63E-14 | 4.65E-08 | UP |
| ENSG00000198889 | DCAF12L1 | 1.310633 | 0.000731 | 0.020154 | UP |
| ENSG00000240583 | AQP1 | 1.310545 | 0.001238 | 0.027034 | UP |
| ENSG00000011600 | TYROBP | 1.309049 | 0.001216 | 0.026781 | UP |
| ENSG00000159251 | ACTC1 | 1.306672 | 5.29E-07 | 0.000334 | UP |
| ENSG00000058866 | DGKG | 1.30085 | 0.00013 | 0.00766 | UP |
| ENSG00000171223 | JUNB | 1.300711 | 0.002024 | 0.035471 | UP |
| ENSG00000220201 | ZGLP1 | 1.300447 | 4.82E-11 | 1.69E-06 | UP |
| ENSG00000140090 | SLC24A4 | 1.300355 | 1.65E-07 | 0.000174 | UP |
| ENSG00000185215 | TNFAIP2 | 1.299745 | 3.53E-08 | 7.23E-05 | UP |
| ENSG00000179695 | OR6C2 | 1.299176 | 3.49E-05 | 0.003617 | UP |
| ENSG00000186815 | TPCN1 | 1.299074 | 6.07E-12 | 5.24E-07 | UP |
| ENSG00000119714 | GPR68 | 1.297679 | 2.06E-08 | 5.33E-05 | UP |
| ENSG00000172830 | SSH3 | 1.296653 | 3.34E-05 | 0.003534 | UP |
| ENSG00000142627 | EPHA2 | 1.295379 | 0.002974 | 0.044019 | UP |
| ENSG00000197530 | MIB2 | 1.294489 | 1.25E-08 | 3.97E-05 | UP |
| ENSG00000139112 | GABARAPL1 | 1.294441 | 1.28E-05 | 0.002062 | UP |
| ENSG00000117461 | PIK3R3 | 1.293995 | 0.000183 | 0.009285 | UP |
| ENSG00000205903 | ZNF316 | 1.291944 | 6.32E-08 | 0.0001 | UP |
| ENSG00000110492 | MDK | 1.291685 | 1.09E-09 | 1.01E-05 | UP |
| ENSG00000173327 | MAP3K11 | 1.290299 | 7.32E-09 | 2.94E-05 | UP |
| ENSG00000105221 | AKT2 | 1.289577 | 1.71E-07 | 0.000178 | UP |
| ENSG00000141540 | TTYH2 | 1.289015 | 1.60E-09 | 1.27E-05 | UP |
| ENSG00000106069 | CHN2 | 1.28894 | 3.94E-12 | 4.09E-07 | UP |
| ENSG00000172765 | TMCC1 | 1.286334 | 0.000153 | 0.008375 | UP |
| ENSG00000139645 | ANKRD52 | 1.284238 | 2.38E-08 | 5.81E-05 | UP |
| ENSG00000099958 | DERL3 | 1.283638 | 6.22E-05 | 0.005026 | UP |
| ENSG00000174529 | TMEM81 | 1.281344 | 1.78E-05 | 0.002477 | UP |
| ENSG00000053438 | NNAT | 1.279583 | 2.12E-07 | 0.000201 | UP |
| ENSG00000170962 | PDGFD | 1.279097 | 3.75E-05 | 0.003771 | UP |
| ENSG00000110400 | PVRL1 | 1.278118 | 3.12E-05 | 0.003403 | UP |
| ENSG00000206013 | IFITM5 | 1.278014 | 0.000564 | 0.017497 | UP |
| ENSG00000061273 | HDAC7 | 1.277774 | 6.20E-07 | 0.000367 | UP |
| ENSG00000167525 | PROCA1 | 1.277352 | 3.81E-05 | 0.003802 | UP |
| ENSG00000129911 | KLF16 | 1.277197 | 5.19E-05 | 0.004538 | UP |
| ENSG00000105287 | PRKD2 | 1.275695 | 7.96E-06 | 0.001571 | UP |
| ENSG00000163795 | ZNF513 | 1.275528 | 3.70E-06 | 0.001014 | UP |
| ENSG00000001084 | GCLC | 1.275661 | 5.32E-06 | 0.001245 | UP |
| ENSG00000136830 | FAM129B | 1.27552 | 1.05E-05 | 0.001851 | UP |
| ENSG00000165804 | ZNF219 | 1.274738 | 1.56E-06 | 0.000622 | UP |
| ENSG00000109927 | TECTA | 1.274004 | 2.76E-07 | 0.000231 | UP |
| ENSG00000162458 | FBLIM1 | 1.273962 | 1.52E-11 | 8.67E-07 | UP |
| ENSG00000151952 | TMEM132D | 1.272884 | 0.001604 | 0.031227 | UP |
| ENSG00000179833 | SERTAD2 | 1.27248 | 3.88E-07 | 0.000282 | UP |
| ENSG00000170075 | GPR37L1 | 1.271708 | 3.62E-05 | 0.003697 | UP |
| ENSG00000100359 | SGSM3 | 1.269998 | 7.56E-12 | 5.86E-07 | UP |
| ENSG00000024862 | CCDC28A | 1.269717 | 0.000272 | 0.011585 | UP |
| ENSG00000125730 | C3 | 1.269346 | 0.001944 | 0.034695 | UP |
| ENSG00000259529 | RP11-468E2.4 | 1.269313 | 5.81E-05 | 0.004835 | UP |
| ENSG00000183570 | PCBP3 | 1.267847 | 0.000177 | 0.009125 | UP |
| ENSG00000104205 | SGK3 | 1.267489 | 4.20E-08 | 7.94E-05 | UP |
| ENSG00000176299 | OR4M1 | 1.267468 | 0.003542 | 0.048595 | UP |
| ENSG00000226174 | TEX22 | 1.265099 | 2.29E-05 | 0.002856 | UP |
| ENSG00000162882 | HAAO | 1.263975 | 9.10E-06 | 0.001699 | UP |
| ENSG00000214511 | HIGD1C | 1.263701 | 4.49E-06 | 0.001132 | UP |
| ENSG00000167987 | VPS37C | 1.263084 | 4.23E-09 | 2.17E-05 | UP |
| ENSG00000051180 | RAD51 | 1.262213 | 1.07E-12 | 1.97E-07 | UP |
| ENSG00000102886 | GDPD3 | 1.261876 | 5.68E-09 | 2.55E-05 | UP |
| ENSG00000135436 | FAM186B | 1.26026 | 0.000298 | 0.012216 | UP |
| ENSG00000134697 | GNL2 | 1.257768 | 8.57E-14 | 4.83E-08 | UP |
| ENSG00000101076 | HNF4A | 1.256805 | 0.001672 | 0.031932 | UP |
| ENSG00000169026 | MFSD7 | 1.25609 | 0.000252 | 0.011101 | UP |
| ENSG00000091592 | NLRP1 | 1.255741 | 4.71E-10 | 6.28E-06 | UP |
| ENSG00000136383 | ALPK3 | 1.254693 | 0.000458 | 0.015524 | UP |
| ENSG00000174004 | NRROS | 1.253729 | 5.91E-06 | 0.001322 | UP |
| ENSG00000133392 | MYH11 | 1.252388 | 4.73E-11 | 1.67E-06 | UP |
| ENSG00000107807 | TLX1 | 1.246678 | 1.43E-06 | 0.000594 | UP |
| ENSG00000141258 | SGSM2 | 1.244579 | 2.18E-11 | 1.06E-06 | UP |
| ENSG00000129219 | PLD2 | 1.244251 | 1.84E-08 | 4.98E-05 | UP |
| ENSG00000118898 | PPL | 1.243151 | 4.92E-12 | 4.67E-07 | UP |
| ENSG00000132359 | RAP1GAP2 | 1.24271 | 8.69E-07 | 0.000445 | UP |
| ENSG00000143537 | ADAM15 | 1.241151 | 4.66E-05 | 0.00426 | UP |
| ENSG00000196700 | ZNF512B | 1.240358 | 0.00027 | 0.011551 | UP |
| ENSG00000144228 | SPOPL | 1.239667 | 0.00011 | 0.006975 | UP |
| ENSG00000141096 | DPEP3 | 1.239546 | 0.000234 | 0.010666 | UP |
| ENSG00000118894 | EEF2KMT | 1.238753 | 4.26E-05 | 0.004047 | UP |
| ENSG00000033122 | LRRC7 | 1.236687 | 1.25E-07 | 0.000148 | UP |
| ENSG00000144619 | CNTN4 | 1.235835 | 0.0016 | 0.03118 | UP |
| ENSG00000171163 | ZNF692 | 1.235664 | 1.45E-07 | 0.000161 | UP |
| ENSG00000119608 | PROX2 | 1.234967 | 0.000527 | 0.016825 | UP |
| ENSG00000162341 | TPCN2 | 1.233634 | 5.90E-07 | 0.000356 | UP |
| ENSG00000112414 | ADGRG6 | 1.231647 | 2.81E-09 | 1.74E-05 | UP |
| ENSG00000101126 | ADNP | 1.231254 | 0.00019 | 0.009493 | UP |
| ENSG00000162522 | KIAA1522 | 1.230174 | 0.000238 | 0.010777 | UP |
| ENSG00000001629 | ANKIB1 | 1.229247 | 0.00193 | 0.034552 | UP |
| ENSG00000146197 | SCUBE3 | 1.228666 | 3.06E-06 | 0.00091 | UP |
| ENSG00000163126 | ANKRD23 | 1.225234 | 0.002301 | 0.038133 | UP |
| ENSG00000105810 | CDK6 | 1.225066 | 0.001567 | 0.030834 | UP |
| ENSG00000187533 | PRR27 | 1.224749 | 1.28E-05 | 0.002058 | UP |
| ENSG00000274443 | C8orf89 | 1.224344 | 0.00068 | 0.019335 | UP |
| ENSG00000072315 | TRPC5 | 1.22368 | 0.002059 | 0.035786 | UP |
| ENSG00000169607 | CKAP2L | 1.222821 | 0.000282 | 0.011832 | UP |
| ENSG00000161281 | COX7A1 | 1.220995 | 9.70E-10 | 9.56E-06 | UP |
| ENSG00000198467 | TPM2 | 1.218655 | 0.000472 | 0.015784 | UP |
| ENSG00000161847 | RAVER1 | 1.218085 | 0.000387 | 0.014146 | UP |
| ENSG00000149499 | EML3 | 1.217963 | 4.94E-07 | 0.000323 | UP |
| ENSG00000102738 | MRPS31 | 1.217947 | 0.00204 | 0.035617 | UP |
| ENSG00000196924 | FLNA | 1.217917 | 1.60E-08 | 4.59E-05 | UP |
| ENSG00000101220 | C20orf27 | 1.216772 | 1.64E-05 | 0.002375 | UP |
| ENSG00000125089 | SH3TC1 | 1.216 | 3.65E-08 | 7.36E-05 | UP |
| ENSG00000253305 | PCDHGB6 | 1.21512 | 1.46E-06 | 0.0006 | UP |
| ENSG00000188981 | MSANTD1 | 1.214971 | 0.003589 | 0.048945 | UP |
| ENSG00000070388 | FGF22 | 1.212775 | 2.93E-05 | 0.003284 | UP |
| ENSG00000103024 | NME3 | 1.210751 | 0.000209 | 0.010033 | UP |
| ENSG00000146826 | C7orf43 | 1.210123 | 8.44E-09 | 3.18E-05 | UP |
| ENSG00000159842 | ABR | 1.209873 | 4.03E-09 | 2.12E-05 | UP |
| ENSG00000114853 | ZBTB47 | 1.209435 | 0.000356 | 0.013492 | UP |
| ENSG00000108602 | ALDH3A1 | 1.207818 | 8.84E-05 | 0.006166 | UP |
| ENSG00000165752 | STK32C | 1.207457 | 1.74E-05 | 0.002451 | UP |
| ENSG00000124574 | ABCC10 | 1.202774 | 1.80E-08 | 4.92E-05 | UP |
| ENSG00000186868 | MAPT | 1.202195 | 4.53E-06 | 0.001136 | UP |
| ENSG00000129204 | USP6 | 1.201691 | 1.68E-08 | 4.73E-05 | UP |
| ENSG00000269113 | TRABD2B | 1.197502 | 1.01E-05 | 0.001801 | UP |
| ENSG00000269743 | SLC25A53 | 1.197064 | 0.000116 | 0.007182 | UP |
| ENSG00000184574 | LPAR5 | 1.196329 | 0.00046 | 0.015563 | UP |
| ENSG00000023839 | ABCC2 | 1.196189 | 0.000264 | 0.011408 | UP |
| ENSG00000106331 | PAX4 | 1.195654 | 0.000432 | 0.015041 | UP |
| ENSG00000277611 | RP1-138B7.6 | 1.195547 | 3.44E-05 | 0.003589 | UP |
| ENSG00000011451 | WIZ | 1.192242 | 1.73E-05 | 0.002445 | UP |
| ENSG00000061938 | TNK2 | 1.191827 | 1.97E-07 | 0.000194 | UP |
| ENSG00000160111 | CPAMD8 | 1.191755 | 0.000122 | 0.007389 | UP |
| ENSG00000111961 | SASH1 | 1.190939 | 3.95E-07 | 0.000285 | UP |
| ENSG00000141577 | CEP131 | 1.190145 | 3.23E-05 | 0.003464 | UP |
| ENSG00000140548 | ZNF710 | 1.189153 | 1.27E-06 | 0.000553 | UP |
| ENSG00000268089 | GABRQ | 1.188389 | 5.81E-06 | 0.001309 | UP |
| ENSG00000091986 | CCDC80 | 1.187214 | 0.003153 | 0.045505 | UP |
| ENSG00000108175 | ZMIZ1 | 1.186914 | 3.77E-08 | 7.48E-05 | UP |
| ENSG00000102032 | RENBP | 1.186469 | 0.00015 | 0.008297 | UP |
| ENSG00000143858 | SYT2 | 1.186468 | 0.001608 | 0.031264 | UP |
| ENSG00000135723 | FHOD1 | 1.186243 | 7.29E-08 | 0.000109 | UP |
| ENSG00000184012 | TMPRSS2 | 1.185244 | 2.99E-11 | 1.28E-06 | UP |
| ENSG00000106070 | GRB10 | 1.185231 | 0.000515 | 0.016616 | UP |
| ENSG00000139970 | RTN1 | 1.183507 | 1.22E-07 | 0.000147 | UP |
| ENSG00000054967 | RELT | 1.181341 | 2.21E-09 | 1.52E-05 | UP |
| ENSG00000141380 | SS18 | 1.180948 | 0.000408 | 0.014582 | UP |
| ENSG00000172638 | EFEMP2 | 1.177929 | 0.000621 | 0.018439 | UP |
| ENSG00000179583 | CIITA | 1.177329 | 1.51E-10 | 3.25E-06 | UP |
| ENSG00000182405 | PGBD4 | 1.175713 | 1.45E-06 | 0.000598 | UP |
| ENSG00000181523 | SGSH | 1.174106 | 4.43E-06 | 0.001123 | UP |
| ENSG00000006638 | TBXA2R | 1.172838 | 0.001611 | 0.0313 | UP |
| ENSG00000173581 | CCDC106 | 1.170061 | 0.000139 | 0.007929 | UP |
| ENSG00000112972 | HMGCS1 | 1.16973 | 0.001113 | 0.025524 | UP |
| ENSG00000133247 | SUV420H2 | 1.169557 | 9.80E-06 | 0.001775 | UP |
| ENSG00000152669 | CCNO | 1.169098 | 3.85E-05 | 0.003819 | UP |
| ENSG00000136051 | KIAA1033 | 1.166489 | 2.24E-09 | 1.53E-05 | UP |
| ENSG00000155846 | PPARGC1B | 1.16636 | 9.07E-09 | 3.30E-05 | UP |
| ENSG00000100554 | ATP6V1D | 1.163153 | 1.68E-05 | 0.002403 | UP |
| ENSG00000140326 | CDAN1 | 1.162418 | 3.49E-11 | 1.40E-06 | UP |
| ENSG00000167700 | MFSD3 | 1.161048 | 0.000131 | 0.007689 | UP |
| ENSG00000205502 | C2CD4B | 1.160659 | 0.001229 | 0.026925 | UP |
| ENSG00000060558 | GNA15 | 1.159952 | 0.000238 | 0.010774 | UP |
| ENSG00000058453 | CROCC | 1.159713 | 1.02E-05 | 0.001809 | UP |
| ENSG00000112787 | FBRSL1 | 1.159003 | 1.00E-04 | 0.006607 | UP |
| ENSG00000052723 | SIKE1 | 1.154926 | 0.000797 | 0.021187 | UP |
| ENSG00000160796 | NBEAL2 | 1.154643 | 3.15E-07 | 0.00025 | UP |
| ENSG00000187144 | SPATA21 | 1.154011 | 0.002543 | 0.040363 | UP |
| ENSG00000186895 | FGF3 | 1.153499 | 0.000375 | 0.013868 | UP |
| ENSG00000002330 | BAD | 1.153195 | 0.000433 | 0.015055 | UP |
| ENSG00000137154 | RPS6 | 1.152797 | 1.52E-06 | 0.000614 | UP |
| ENSG00000186198 | SLC51B | 1.152128 | 0.000191 | 0.009529 | UP |
| ENSG00000276581 | SPATA31A5 | 1.14993 | 0.001868 | 0.033946 | UP |
| ENSG00000064225 | ST3GAL6 | 1.149916 | 5.88E-07 | 0.000356 | UP |
| ENSG00000116906 | GNPAT | 1.149738 | 1.43E-06 | 0.000594 | UP |
| ENSG00000139223 | ANP32D | 1.149458 | 1.15E-07 | 0.000141 | UP |
| ENSG00000167528 | ZNF641 | 1.147096 | 9.87E-05 | 0.00656 | UP |
| ENSG00000176903 | PNMA1 | 1.146622 | 0.000154 | 0.008401 | UP |
| ENSG00000105321 | CCDC9 | 1.145265 | 0.000576 | 0.017702 | UP |
| ENSG00000176058 | TPRN | 1.142406 | 0.000374 | 0.013868 | UP |
| ENSG00000029559 | IBSP | 1.141977 | 9.28E-05 | 0.006335 | UP |
| ENSG00000116213 | WRAP73 | 1.141446 | 4.45E-10 | 6.09E-06 | UP |
| ENSG00000100068 | LRP5L | 1.141182 | 7.17E-08 | 0.000108 | UP |
| ENSG00000149792 | MRPL49 | 1.139557 | 0.000179 | 0.009168 | UP |
| ENSG00000139631 | CSAD | 1.139254 | 1.19E-09 | 1.07E-05 | UP |
| ENSG00000171217 | CLDN20 | 1.138034 | 4.97E-05 | 0.004421 | UP |
| ENSG00000176248 | ANAPC2 | 1.137351 | 2.44E-08 | 5.87E-05 | UP |
| ENSG00000066379 | ZNRD1 | 1.136807 | 0.002973 | 0.044014 | UP |
| ENSG00000154079 | SDHAF4 | 1.136743 | 2.17E-06 | 0.000748 | UP |
| ENSG00000107159 | CA9 | 1.134823 | 2.22E-07 | 0.000206 | UP |
| ENSG00000215041 | NEURL4 | 1.132158 | 2.97E-08 | 6.54E-05 | UP |
| ENSG00000130508 | PXDN | 1.132109 | 0.000221 | 0.010313 | UP |
| ENSG00000151006 | PRSS53 | 1.131371 | 0.000247 | 0.010993 | UP |
| ENSG00000182557 | SPNS3 | 1.130756 | 0.003533 | 0.048521 | UP |
| ENSG00000127125 | PPCS | 1.13001 | 0.000281 | 0.011797 | UP |
| ENSG00000170786 | SDR16C5 | 1.128359 | 0.003487 | 0.048178 | UP |
| ENSG00000153558 | FBXL2 | 1.128023 | 2.30E-05 | 0.002866 | UP |
| ENSG00000197081 | IGF2R | 1.125572 | 6.94E-11 | 2.08E-06 | UP |
| ENSG00000101280 | ANGPT4 | 1.125359 | 0.000339 | 0.013112 | UP |
| ENSG00000112996 | MRPS30 | 1.125106 | 0.00289 | 0.043332 | UP |
| ENSG00000092969 | TGFB2 | 1.124911 | 0.000361 | 0.0136 | UP |
| ENSG00000072182 | ASIC4 | 1.124587 | 4.52E-06 | 0.001135 | UP |
| ENSG00000130479 | MAP1S | 1.123916 | 9.86E-05 | 0.006559 | UP |
| ENSG00000109063 | MYH3 | 1.121963 | 0.000114 | 0.007105 | UP |
| ENSG00000135929 | CYP27A1 | 1.121788 | 0.002153 | 0.03671 | UP |
| ENSG00000139055 | ERP27 | 1.121505 | 0.00023 | 0.010568 | UP |
| ENSG00000214491 | SEC14L6 | 1.119309 | 0.001172 | 0.026233 | UP |
| ENSG00000173531 | MST1 | 1.118774 | 0.002458 | 0.03961 | UP |
| ENSG00000162576 | MXRA8 | 1.118084 | 0.000347 | 0.01329 | UP |
| ENSG00000113319 | RASGRF2 | 1.117361 | 8.19E-05 | 0.005895 | UP |
| ENSG00000182378 | PLCXD1 | 1.116633 | 0.000687 | 0.019449 | UP |
| ENSG00000162552 | WNT4 | 1.116454 | 0.00019 | 0.009492 | UP |
| ENSG00000197632 | SERPINB2 | 1.114227 | 6.17E-07 | 0.000366 | UP |
| ENSG00000038382 | TRIO | 1.113555 | 7.88E-09 | 3.07E-05 | UP |
| ENSG00000108984 | MAP2K6 | 1.11286 | 0.000632 | 0.018597 | UP |
| ENSG00000103042 | SLC38A7 | 1.110917 | 8.98E-05 | 0.006222 | UP |
| ENSG00000120235 | IFNA6 | 1.110265 | 7.59E-10 | 8.30E-06 | UP |
| ENSG00000169925 | BRD3 | 1.108808 | 2.01E-09 | 1.44E-05 | UP |
| ENSG00000068831 | RASGRP2 | 1.108749 | 8.93E-09 | 3.28E-05 | UP |
| ENSG00000079313 | REXO1 | 1.108566 | 5.10E-06 | 0.001215 | UP |
| ENSG00000025039 | RRAGD | 1.108121 | 0.001228 | 0.026923 | UP |
| ENSG00000244414 | CFHR1 | 1.107811 | 4.77E-09 | 2.31E-05 | UP |
| ENSG00000160214 | RRP1 | 1.10773 | 2.73E-07 | 0.00023 | UP |
| ENSG00000110042 | DTX4 | 1.107715 | 4.17E-05 | 0.004 | UP |
| ENSG00000161202 | DVL3 | 1.106973 | 0.001053 | 0.024721 | UP |
| ENSG00000115221 | ITGB6 | 1.106156 | 9.27E-07 | 0.000462 | UP |
| ENSG00000168140 | VASN | 1.105812 | 0.000108 | 0.006887 | UP |
| ENSG00000205356 | TECPR1 | 1.104337 | 1.02E-07 | 0.000133 | UP |
| ENSG00000196912 | ANKRD36B | 1.103727 | 7.91E-05 | 0.005772 | UP |
| ENSG00000040199 | PHLPP2 | 1.103716 | 3.12E-07 | 0.000249 | UP |
| ENSG00000146094 | DOK3 | 1.103599 | 0.000274 | 0.011639 | UP |
| ENSG00000204839 | MROH6 | 1.103533 | 0.00037 | 0.013781 | UP |
| ENSG00000088854 | C20orf194 | 1.103379 | 1.39E-06 | 0.000583 | UP |
| ENSG00000099821 | POLRMT | 1.102665 | 9.26E-06 | 0.001717 | UP |
| ENSG00000139625 | MAP3K12 | 1.101071 | 4.73E-06 | 0.001163 | UP |
| ENSG00000149115 | TNKS1BP1 | 1.101056 | 0.000656 | 0.018947 | UP |
| ENSG00000139890 | REM2 | 1.100204 | 5.88E-07 | 0.000356 | UP |
| ENSG00000124104 | SNX21 | 1.100036 | 0.000119 | 0.007283 | UP |
| ENSG00000164620 | RELL2 | 1.099529 | 0.000466 | 0.015666 | UP |
| ENSG00000101384 | JAG1 | 1.099019 | 0.002546 | 0.040378 | UP |
| ENSG00000085185 | BCORL1 | 1.09821 | 1.35E-05 | 0.002125 | UP |
| ENSG00000006453 | BAIAP2L1 | 1.097796 | 0.000178 | 0.009162 | UP |
| ENSG00000186881 | OR13F1 | 1.094814 | 1.04E-06 | 0.000495 | UP |
| ENSG00000089558 | KCNH4 | 1.094811 | 0.000923 | 0.022958 | UP |
| ENSG00000142208 | AKT1 | 1.092214 | 4.13E-06 | 0.001077 | UP |
| ENSG00000196588 | MKL1 | 1.090837 | 5.01E-09 | 2.36E-05 | UP |
| ENSG00000132329 | RAMP1 | 1.089982 | 6.30E-06 | 0.001371 | UP |
| ENSG00000139174 | PRICKLE1 | 1.08992 | 0.002676 | 0.04149 | UP |
| ENSG00000187105 | HEATR4 | 1.088936 | 0.000452 | 0.015423 | UP |
| ENSG00000104332 | SFRP1 | 1.088504 | 7.14E-10 | 8.03E-06 | UP |
| ENSG00000178718 | RPP25 | 1.088366 | 0.001883 | 0.034096 | UP |
| ENSG00000131467 | PSME3 | 1.087027 | 4.74E-07 | 0.000316 | UP |
| ENSG00000164889 | SLC4A2 | 1.0857 | 3.69E-06 | 0.001013 | UP |
| ENSG00000111254 | AKAP3 | 1.084871 | 3.78E-06 | 0.001025 | UP |
| ENSG00000181698 | OR5T1 | 1.084346 | 7.25E-10 | 8.09E-06 | UP |
| ENSG00000138079 | SLC3A1 | 1.082376 | 0.000119 | 0.00727 | UP |
| ENSG00000132694 | ARHGEF11 | 1.080009 | 1.94E-06 | 0.000703 | UP |
| ENSG00000145730 | PAM | 1.078473 | 1.93E-05 | 0.002593 | UP |
| ENSG00000015153 | YAF2 | 1.07764 | 0.000182 | 0.009259 | UP |
| ENSG00000132361 | CLUH | 1.076533 | 0.000107 | 0.006857 | UP |
| ENSG00000226479 | TMEM185B | 1.076475 | 2.61E-06 | 0.000831 | UP |
| ENSG00000214021 | TTLL3 | 1.076258 | 1.97E-11 | 1.01E-06 | UP |
| ENSG00000146192 | FGD2 | 1.07611 | 6.53E-13 | 1.48E-07 | UP |
| ENSG00000078814 | MYH7B | 1.075452 | 2.22E-06 | 0.000758 | UP |
| ENSG00000122547 | EEPD1 | 1.074445 | 1.31E-06 | 0.000565 | UP |
| ENSG00000215029 | TCP11X2 | 1.073689 | 6.13E-09 | 2.66E-05 | UP |
| ENSG00000068650 | ATP11A | 1.072895 | 1.05E-11 | 6.99E-07 | UP |
| ENSG00000117528 | ABCD3 | 1.072782 | 0.001023 | 0.024307 | UP |
| ENSG00000197841 | ZNF181 | 1.072153 | 1.04E-06 | 0.000493 | UP |
| ENSG00000100413 | POLR3H | 1.071439 | 3.96E-05 | 0.00388 | UP |
| ENSG00000005007 | UPF1 | 1.069818 | 5.66E-09 | 2.54E-05 | UP |
| ENSG00000149016 | TUT1 | 1.066817 | 4.39E-05 | 0.004118 | UP |
| ENSG00000115282 | TTC31 | 1.066057 | 3.45E-07 | 0.000264 | UP |
| ENSG00000239605 | C2orf61 | 1.064614 | 0.001349 | 0.028391 | UP |
| ENSG00000161960 | EIF4A1 | 1.064466 | 6.37E-08 | 0.000101 | UP |
| ENSG00000274736 | CCL23 | 1.06416 | 0.00021 | 0.010053 | UP |
| ENSG00000160191 | PDE9A | 1.063506 | 0.000592 | 0.017959 | UP |
| ENSG00000160293 | VAV2 | 1.062995 | 1.48E-06 | 0.000605 | UP |
| ENSG00000164070 | HSPA4L | 1.061874 | 0.002528 | 0.040248 | UP |
| ENSG00000057149 | SERPINB3 | 1.060962 | 0.00014 | 0.00797 | UP |
| ENSG00000136205 | TNS3 | 1.060255 | 3.37E-09 | 1.92E-05 | UP |
| ENSG00000282872 | RP11-96L14.8 | 1.060096 | 0.000318 | 0.012651 | UP |
| ENSG00000141564 | RPTOR | 1.059997 | 0.000603 | 0.018149 | UP |
| ENSG00000103249 | CLCN7 | 1.059953 | 9.89E-06 | 0.001784 | UP |
| ENSG00000244607 | CCDC13 | 1.058295 | 0.001352 | 0.028423 | UP |
| ENSG00000111452 | ADGRD1 | 1.057725 | 0.001228 | 0.026922 | UP |
| ENSG00000187961 | KLHL17 | 1.057438 | 0.000963 | 0.023507 | UP |
| ENSG00000197114 | ZGPAT | 1.056714 | 2.34E-05 | 0.002892 | UP |
| ENSG00000127415 | IDUA | 1.056401 | 0.00058 | 0.01775 | UP |
| ENSG00000161653 | NAGS | 1.056289 | 0.001369 | 0.028619 | UP |
| ENSG00000123454 | DBH | 1.055577 | 0.002826 | 0.042792 | UP |
| ENSG00000087077 | TRIP6 | 1.053875 | 0.000325 | 0.012817 | UP |
| ENSG00000148459 | PDSS1 | 1.053226 | 0.003569 | 0.048783 | UP |
| ENSG00000173567 | ADGRF3 | 1.053226 | 0.000204 | 0.009888 | UP |
| ENSG00000168056 | LTBP3 | 1.052767 | 5.94E-05 | 0.004896 | UP |
| ENSG00000111321 | LTBR | 1.052755 | 0.000102 | 0.006691 | UP |
| ENSG00000167522 | ANKRD11 | 1.05215 | 1.49E-11 | 8.59E-07 | UP |
| ENSG00000197093 | GAL3ST4 | 1.050633 | 0.000297 | 0.012201 | UP |
| ENSG00000186522 | 10-Sep | 1.047183 | 0.000923 | 0.022958 | UP |
| ENSG00000143554 | SLC27A3 | 1.045442 | 0.000138 | 0.007901 | UP |
| ENSG00000105355 | PLIN3 | 1.044681 | 9.71E-05 | 0.006507 | UP |
| ENSG00000280410 | AC104073.2 | 1.043472 | 3.46E-07 | 0.000265 | UP |
| ENSG00000177508 | IRX3 | 1.043464 | 0.001636 | 0.031566 | UP |
| ENSG00000124181 | PLCG1 | 1.042992 | 1.54E-07 | 0.000167 | UP |
| ENSG00000155254 | MARVELD1 | 1.042779 | 0.000616 | 0.018347 | UP |
| ENSG00000184524 | CEND1 | 1.041349 | 0.00026 | 0.011305 | UP |
| ENSG00000128159 | TUBGCP6 | 1.040678 | 9.98E-07 | 0.000482 | UP |
| ENSG00000126522 | ASL | 1.039539 | 9.14E-06 | 0.001704 | UP |
| ENSG00000116990 | MYCL | 1.038628 | 1.12E-05 | 0.001917 | UP |
| ENSG00000107957 | SH3PXD2A | 1.038028 | 0.001465 | 0.029695 | UP |
| ENSG00000124313 | IQSEC2 | 1.037537 | 0.003112 | 0.045196 | UP |
| ENSG00000185000 | DGAT1 | 1.036753 | 5.38E-08 | 9.16E-05 | UP |
| ENSG00000159788 | RGS12 | 1.036141 | 7.33E-07 | 0.000404 | UP |
| ENSG00000107263 | RAPGEF1 | 1.035514 | 3.47E-07 | 0.000265 | UP |
| ENSG00000014914 | MTMR11 | 1.035063 | 0.001268 | 0.027399 | UP |
| ENSG00000173678 | SPDYE2B | 1.034012 | 0.000117 | 0.007213 | UP |
| ENSG00000077044 | DGKD | 1.033004 | 2.10E-07 | 0.0002 | UP |
| ENSG00000175727 | MLXIP | 1.031195 | 2.49E-07 | 0.000219 | UP |
| ENSG00000106609 | TMEM248 | 1.031098 | 8.17E-07 | 0.00043 | UP |
| ENSG00000107551 | RASSF4 | 1.030865 | 4.37E-05 | 0.004111 | UP |
| ENSG00000163472 | TMEM79 | 1.030534 | 0.000607 | 0.018211 | UP |
| ENSG00000105639 | JAK3 | 1.030202 | 2.89E-08 | 6.44E-05 | UP |
| ENSG00000173137 | ADCK5 | 1.030112 | 4.19E-05 | 0.004012 | UP |
| ENSG00000168237 | GLYCTK | 1.028987 | 1.01E-06 | 0.000485 | UP |
| ENSG00000145016 | RUBCN | 1.028523 | 9.33E-10 | 9.34E-06 | UP |
| ENSG00000119685 | TTLL5 | 1.026268 | 3.40E-06 | 0.000967 | UP |
| ENSG00000144136 | SLC20A1 | 1.025354 | 0.000424 | 0.014897 | UP |
| ENSG00000155229 | MMS19 | 1.025268 | 1.20E-07 | 0.000145 | UP |
| ENSG00000231861 | OR5K2 | 1.02411 | 4.40E-07 | 0.000303 | UP |
| ENSG00000078808 | SDF4 | 1.023743 | 2.57E-05 | 0.003047 | UP |
| ENSG00000171603 | CLSTN1 | 1.023679 | 3.87E-07 | 0.000281 | UP |
| ENSG00000118369 | USP35 | 1.021477 | 5.28E-07 | 0.000334 | UP |
| ENSG00000117713 | ARID1A | 1.020355 | 3.33E-07 | 0.000259 | UP |
| ENSG00000089351 | GRAMD1A | 1.020349 | 1.02E-05 | 0.001808 | UP |
| ENSG00000183828 | NUDT14 | 1.020262 | 0.001159 | 0.026101 | UP |
| ENSG00000176273 | SLC35G1 | 1.01965 | 0.000133 | 0.007737 | UP |
| ENSG00000068024 | HDAC4 | 1.019458 | 1.99E-10 | 3.83E-06 | UP |
| ENSG00000260097 | SPDYE6 | 1.018922 | 0.001376 | 0.028699 | UP |
| ENSG00000166598 | HSP90B1 | 1.017534 | 0.001286 | 0.02759 | UP |
| ENSG00000105519 | CAPS | 1.016471 | 0.000134 | 0.007758 | UP |
| ENSG00000168781 | PPIP5K1 | 1.016003 | 0.001662 | 0.031835 | UP |
| ENSG00000099338 | CATSPERG | 1.015579 | 3.83E-05 | 0.003809 | UP |
| ENSG00000124602 | UNC5CL | 1.014223 | 0.000319 | 0.012673 | UP |
| ENSG00000179364 | PACS2 | 1.012836 | 1.01E-06 | 0.000485 | UP |
| ENSG00000101407 | TTI1 | 1.012101 | 9.37E-08 | 0.000126 | UP |
| ENSG00000154803 | FLCN | 1.012048 | 0.001149 | 0.025958 | UP |
| ENSG00000130717 | UCK1 | 1.011363 | 0.000652 | 0.018886 | UP |
| ENSG00000233436 | BTBD18 | 1.009844 | 0.000313 | 0.012553 | UP |
| ENSG00000107105 | ELAVL2 | 1.007206 | 0.001672 | 0.031932 | UP |
| ENSG00000134245 | WNT2B | 1.007008 | 8.61E-05 | 0.006075 | UP |
| ENSG00000162944 | RFTN2 | 1.006337 | 0.002009 | 0.035341 | UP |
| ENSG00000188522 | FAM83G | 1.005387 | 0.001459 | 0.029628 | UP |
| ENSG00000015133 | CCDC88C | 1.004933 | 2.84E-09 | 1.75E-05 | UP |
| ENSG00000153823 | PID1 | 1.00314 | 0.001289 | 0.027617 | UP |
| ENSG00000140006 | WDR89 | 1.002743 | 7.16E-05 | 0.005449 | UP |
| ENSG00000121769 | FABP3 | 1.002523 | 0.001834 | 0.033615 | UP |
| ENSG00000186318 | BACE1 | 1.00209 | 3.49E-07 | 0.000266 | UP |
| ENSG00000162650 | ATXN7L2 | 1.001896 | 0.001076 | 0.02504 | UP |
| ENSG00000139636 | LMBR1L | 1.000804 | 1.72E-08 | 4.79E-05 | UP |
| ENSG00000085563 | ABCB1 | 1.000453 | 0.00168 | 0.031986 | UP |
| ENSG00000176641 | RNF152 | 1.000082 | 0.000807 | 0.021332 | UP |
| ENSG00000175894 | TSPEAR | -1.00004 | 0.002574 | 0.040608 | DOWN |
| ENSG00000135919 | SERPINE2 | -1.00031 | 0.003084 | 0.044966 | DOWN |
| ENSG00000205189 | ZBTB10 | -1.0004 | 3.00E-05 | 0.003324 | DOWN |
| ENSG00000125629 | INSIG2 | -1.00043 | 1.49E-05 | 0.002244 | DOWN |
| ENSG00000100815 | TRIP11 | -1.00063 | 5.90E-11 | 1.89E-06 | DOWN |
| ENSG00000114127 | XRN1 | -1.00111 | 5.68E-05 | 0.00477 | DOWN |
| ENSG00000187855 | ASCL4 | -1.00154 | 3.24E-07 | 0.000254 | DOWN |
| ENSG00000118849 | RARRES1 | -1.00268 | 2.21E-06 | 0.000755 | DOWN |
| ENSG00000152219 | ARL14EP | -1.00286 | 2.55E-07 | 0.000222 | DOWN |
| ENSG00000147894 | C9orf72 | -1.00293 | 2.22E-05 | 0.002809 | DOWN |
| ENSG00000133657 | ATP13A3 | -1.00347 | 4.93E-10 | 6.43E-06 | DOWN |
| ENSG00000171612 | SLC25A33 | -1.00376 | 0.000467 | 0.015687 | DOWN |
| ENSG00000181704 | YIPF6 | -1.00521 | 0.000434 | 0.015078 | DOWN |
| ENSG00000109536 | FRG1 | -1.00535 | 5.10E-07 | 0.000328 | DOWN |
| ENSG00000166575 | TMEM135 | -1.0055 | 1.13E-07 | 0.000141 | DOWN |
| ENSG00000122870 | BICC1 | -1.0056 | 1.30E-05 | 0.002075 | DOWN |
| ENSG00000108506 | INTS2 | -1.00588 | 0.002025 | 0.035476 | DOWN |
| ENSG00000166130 | IKBIP | -1.00607 | 2.23E-07 | 0.000207 | DOWN |
| ENSG00000198440 | ZNF583 | -1.00634 | 9.11E-05 | 0.006269 | DOWN |
| ENSG00000180035 | ZNF48 | -1.00639 | 1.53E-08 | 4.47E-05 | DOWN |
| ENSG00000120992 | LYPLA1 | -1.00651 | 1.78E-06 | 0.00067 | DOWN |
| ENSG00000100664 | EIF5 | -1.00669 | 1.52E-05 | 0.002269 | DOWN |
| ENSG00000115520 | COQ10B | -1.00689 | 6.36E-07 | 0.000372 | DOWN |
| ENSG00000179387 | ELMOD2 | -1.0071 | 2.08E-07 | 0.000199 | DOWN |
| ENSG00000135972 | MRPS9 | -1.00731 | 2.06E-10 | 3.90E-06 | DOWN |
| ENSG00000167306 | MYO5B | -1.00752 | 0.000349 | 0.013333 | DOWN |
| ENSG00000109743 | BST1 | -1.00801 | 3.07E-10 | 4.89E-06 | DOWN |
| ENSG00000247595 | SPTY2D1-AS1 | -1.00801 | 0.002111 | 0.036295 | DOWN |
| ENSG00000136942 | RPL35 | -1.00858 | 0.002079 | 0.036003 | DOWN |
| ENSG00000086598 | TMED2 | -1.00868 | 1.21E-08 | 3.90E-05 | DOWN |
| ENSG00000165409 | TSHR | -1.00902 | 4.80E-09 | 2.31E-05 | DOWN |
| ENSG00000142892 | PIGK | -1.00918 | 1.39E-05 | 0.002163 | DOWN |
| ENSG00000174600 | CMKLR1 | -1.00941 | 0.000667 | 0.019127 | DOWN |
| ENSG00000146476 | ARMT1 | -1.00943 | 6.73E-12 | 5.54E-07 | DOWN |
| ENSG00000069345 | DNAJA2 | -1.01014 | 3.22E-05 | 0.003459 | DOWN |
| ENSG00000145824 | CXCL14 | -1.01093 | 1.88E-07 | 0.000188 | DOWN |
| ENSG00000139679 | LPAR6 | -1.01153 | 5.48E-06 | 0.001264 | DOWN |
| ENSG00000122873 | CISD1 | -1.01207 | 6.86E-07 | 0.000389 | DOWN |
| ENSG00000113525 | IL5 | -1.01272 | 4.94E-05 | 0.00441 | DOWN |
| ENSG00000128585 | MKLN1 | -1.01291 | 1.69E-06 | 0.00065 | DOWN |
| ENSG00000102409 | BEX4 | -1.01306 | 2.18E-08 | 5.51E-05 | DOWN |
| ENSG00000120063 | GNA13 | -1.01312 | 1.38E-06 | 0.000582 | DOWN |
| ENSG00000145901 | TNIP1 | -1.01355 | 8.02E-10 | 8.57E-06 | DOWN |
| ENSG00000102781 | KATNAL1 | -1.01372 | 1.23E-06 | 0.000544 | DOWN |
| ENSG00000143147 | GPR161 | -1.01388 | 0.001117 | 0.025566 | DOWN |
| ENSG00000102595 | UGGT2 | -1.01433 | 6.49E-05 | 0.005155 | DOWN |
| ENSG00000196268 | ZNF493 | -1.0144 | 3.68E-09 | 2.02E-05 | DOWN |
| ENSG00000019144 | PHLDB1 | -1.01472 | 5.33E-10 | 6.71E-06 | DOWN |
| ENSG00000133773 | CCDC59 | -1.01612 | 9.18E-07 | 0.00046 | DOWN |
| ENSG00000125257 | ABCC4 | -1.01623 | 0.000255 | 0.011181 | DOWN |
| ENSG00000115966 | ATF2 | -1.01656 | 2.64E-11 | 1.19E-06 | DOWN |
| ENSG00000125691 | RPL23 | -1.01679 | 0.001038 | 0.024513 | DOWN |
| ENSG00000123411 | IKZF4 | -1.01695 | 2.92E-07 | 0.000239 | DOWN |
| ENSG00000182899 | RPL35A | -1.01732 | 0.000274 | 0.011629 | DOWN |
| ENSG00000147050 | KDM6A | -1.01746 | 6.12E-14 | 4.14E-08 | DOWN |
| ENSG00000113407 | TARS | -1.01809 | 2.47E-06 | 0.000805 | DOWN |
| ENSG00000140694 | PARN | -1.01951 | 1.81E-09 | 1.36E-05 | DOWN |
| ENSG00000204614 | TRIM40 | -1.01953 | 0.000102 | 0.006694 | DOWN |
| ENSG00000221944 | TIGD1 | -1.02006 | 0.000124 | 0.007429 | DOWN |
| ENSG00000106701 | FSD1L | -1.02014 | 1.28E-05 | 0.002063 | DOWN |
| ENSG00000150316 | CWC15 | -1.0203 | 8.58E-09 | 3.21E-05 | DOWN |
| ENSG00000113391 | FAM172A | -1.02042 | 3.87E-08 | 7.59E-05 | DOWN |
| ENSG00000151332 | MBIP | -1.02084 | 1.15E-05 | 0.001944 | DOWN |
| ENSG00000133119 | RFC3 | -1.02153 | 0.000319 | 0.012675 | DOWN |
| ENSG00000198791 | CNOT7 | -1.0216 | 8.39E-09 | 3.17E-05 | DOWN |
| ENSG00000172671 | ZFAND4 | -1.02163 | 3.54E-06 | 0.00099 | DOWN |
| ENSG00000100578 | KIAA0586 | -1.02198 | 1.38E-10 | 3.09E-06 | DOWN |
| ENSG00000112303 | VNN2 | -1.02217 | 2.89E-06 | 0.000881 | DOWN |
| ENSG00000173597 | SULT1B1 | -1.0234 | 0.001874 | 0.034003 | DOWN |
| ENSG00000136122 | BORA | -1.02344 | 2.14E-06 | 0.000741 | DOWN |
| ENSG00000111266 | DUSP16 | -1.02383 | 6.26E-06 | 0.001366 | DOWN |
| ENSG00000204323 | SMIM5 | -1.02475 | 1.08E-06 | 0.000503 | DOWN |
| ENSG00000278705 | HIST1H4B | -1.02517 | 2.24E-08 | 5.60E-05 | DOWN |
| ENSG00000143751 | SDE2 | -1.02548 | 0.000614 | 0.018321 | DOWN |
| ENSG00000175548 | ALG10B | -1.02593 | 2.08E-06 | 0.000729 | DOWN |
| ENSG00000111845 | PAK1IP1 | -1.02604 | 3.11E-07 | 0.000248 | DOWN |
| ENSG00000046651 | OFD1 | -1.02622 | 0.000886 | 0.022427 | DOWN |
| ENSG00000129317 | PUS7L | -1.02697 | 4.32E-11 | 1.58E-06 | DOWN |
| ENSG00000172331 | BPGM | -1.02775 | 2.14E-05 | 0.002751 | DOWN |
| ENSG00000115540 | MOB4 | -1.02792 | 2.05E-05 | 0.002681 | DOWN |
| ENSG00000108733 | PEX12 | -1.02796 | 7.07E-05 | 0.005408 | DOWN |
| ENSG00000169884 | WNT10B | -1.02892 | 4.72E-05 | 0.004292 | DOWN |
| ENSG00000095574 | IKZF5 | -1.02899 | 0.001253 | 0.027207 | DOWN |
| ENSG00000101639 | CEP192 | -1.02922 | 4.28E-06 | 0.001099 | DOWN |
| ENSG00000237440 | ZNF737 | -1.02922 | 1.17E-07 | 0.000143 | DOWN |
| ENSG00000276410 | HIST1H2BB | -1.02945 | 3.89E-08 | 7.60E-05 | DOWN |
| ENSG00000112309 | B3GAT2 | -1.03004 | 0.000632 | 0.018608 | DOWN |
| ENSG00000128829 | EIF2AK4 | -1.03087 | 2.18E-10 | 4.02E-06 | DOWN |
| ENSG00000103569 | AQP9 | -1.03126 | 5.62E-07 | 0.000347 | DOWN |
| ENSG00000164329 | PAPD4 | -1.03148 | 2.46E-07 | 0.000218 | DOWN |
| ENSG00000214517 | PPME1 | -1.03205 | 2.05E-05 | 0.002683 | DOWN |
| ENSG00000150779 | TIMM8B | -1.03225 | 0.000303 | 0.012315 | DOWN |
| ENSG00000120742 | SERP1 | -1.03279 | 2.48E-09 | 1.62E-05 | DOWN |
| ENSG00000103423 | DNAJA3 | -1.0342 | 1.35E-05 | 0.002119 | DOWN |
| ENSG00000122787 | AKR1D1 | -1.03424 | 1.23E-10 | 2.87E-06 | DOWN |
| ENSG00000171928 | TVP23B | -1.03451 | 1.96E-06 | 0.000706 | DOWN |
| ENSG00000115828 | QPCT | -1.03521 | 1.67E-06 | 0.000645 | DOWN |
| ENSG00000156467 | UQCRB | -1.03529 | 1.47E-07 | 0.000163 | DOWN |
| ENSG00000178974 | FBXO34 | -1.03599 | 2.45E-08 | 5.89E-05 | DOWN |
| ENSG00000134371 | CDC73 | -1.03615 | 4.24E-08 | 7.98E-05 | DOWN |
| ENSG00000083642 | PDS5B | -1.03672 | 1.20E-05 | 0.001987 | DOWN |
| ENSG00000156735 | BAG4 | -1.03684 | 5.47E-08 | 9.24E-05 | DOWN |
| ENSG00000136146 | MED4 | -1.03711 | 1.71E-05 | 0.002431 | DOWN |
| ENSG00000101003 | GINS1 | -1.03733 | 3.74E-05 | 0.003767 | DOWN |
| ENSG00000137947 | GTF2B | -1.03746 | 6.51E-13 | 1.48E-07 | DOWN |
| ENSG00000136536 | 7-Mar | -1.03757 | 2.18E-11 | 1.06E-06 | DOWN |
| ENSG00000126267 | COX6B1 | -1.03765 | 2.51E-06 | 0.000813 | DOWN |
| ENSG00000177932 | ZNF354C | -1.03822 | 2.55E-08 | 6.02E-05 | DOWN |
| ENSG00000176658 | MYO1D | -1.04005 | 0.000375 | 0.013872 | DOWN |
| ENSG00000111727 | HCFC2 | -1.04088 | 2.03E-06 | 0.000722 | DOWN |
| ENSG00000154813 | DPH3 | -1.04166 | 3.74E-08 | 7.45E-05 | DOWN |
| ENSG00000113851 | CRBN | -1.04237 | 3.90E-10 | 5.63E-06 | DOWN |
| ENSG00000206177 | HBM | -1.04333 | 2.97E-05 | 0.003312 | DOWN |
| ENSG00000102145 | GATA1 | -1.04339 | 2.87E-05 | 0.003245 | DOWN |
| ENSG00000137462 | TLR2 | -1.04397 | 4.38E-09 | 2.21E-05 | DOWN |
| ENSG00000212127 | TAS2R14 | -1.04503 | 0.002813 | 0.042694 | DOWN |
| ENSG00000155508 | CNOT8 | -1.04581 | 1.25E-11 | 7.73E-07 | DOWN |
| ENSG00000110031 | LPXN | -1.04604 | 0.001624 | 0.031446 | DOWN |
| ENSG00000120137 | PANK3 | -1.04692 | 2.67E-05 | 0.003114 | DOWN |
| ENSG00000116171 | SCP2 | -1.04718 | 1.88E-07 | 0.000188 | DOWN |
| ENSG00000104047 | DTWD1 | -1.0472 | 4.95E-10 | 6.43E-06 | DOWN |
| ENSG00000111666 | CHPT1 | -1.04933 | 0.000459 | 0.015543 | DOWN |
| ENSG00000130513 | GDF15 | -1.05007 | 0.002044 | 0.035649 | DOWN |
| ENSG00000146648 | EGFR | -1.05009 | 3.81E-05 | 0.003797 | DOWN |
| ENSG00000185885 | IFITM1 | -1.05023 | 0.000129 | 0.007613 | DOWN |
| ENSG00000090612 | ZNF268 | -1.05075 | 0.001452 | 0.02955 | DOWN |
| ENSG00000116209 | TMEM59 | -1.05102 | 1.98E-14 | 2.12E-08 | DOWN |
| ENSG00000172379 | ARNT2 | -1.05124 | 1.49E-09 | 1.22E-05 | DOWN |
| ENSG00000047365 | ARAP2 | -1.05221 | 0.000163 | 0.008698 | DOWN |
| ENSG00000127081 | ZNF484 | -1.05243 | 0.000133 | 0.007754 | DOWN |
| ENSG00000086300 | SNX10 | -1.05273 | 4.10E-08 | 7.83E-05 | DOWN |
| ENSG00000162929 | KIAA1841 | -1.0538 | 1.75E-08 | 4.83E-05 | DOWN |
| ENSG00000175279 | APITD1 | -1.05532 | 1.38E-08 | 4.23E-05 | DOWN |
| ENSG00000172201 | ID4 | -1.05545 | 2.79E-05 | 0.003193 | DOWN |
| ENSG00000143476 | DTL | -1.05589 | 3.36E-06 | 0.00096 | DOWN |
| ENSG00000154727 | GABPA | -1.05703 | 3.81E-09 | 2.06E-05 | DOWN |
| ENSG00000221886 | ZBED8 | -1.05734 | 0.000981 | 0.023749 | DOWN |
| ENSG00000032219 | ARID4A | -1.05764 | 2.60E-08 | 6.08E-05 | DOWN |
| ENSG00000184661 | CDCA2 | -1.05868 | 4.15E-05 | 0.003991 | DOWN |
| ENSG00000196417 | ZNF765 | -1.05896 | 1.31E-05 | 0.002085 | DOWN |
| ENSG00000135018 | UBQLN1 | -1.05906 | 1.06E-07 | 0.000135 | DOWN |
| ENSG00000146263 | MMS22L | -1.0593 | 1.82E-07 | 0.000185 | DOWN |
| ENSG00000131469 | RPL27 | -1.05981 | 0.000271 | 0.01156 | DOWN |
| ENSG00000139610 | CELA1 | -1.06018 | 0.000104 | 0.006754 | DOWN |
| ENSG00000019991 | HGF | -1.06089 | 0.000664 | 0.019081 | DOWN |
| ENSG00000083099 | LYRM2 | -1.06109 | 2.84E-08 | 6.37E-05 | DOWN |
| ENSG00000179083 | FAM133A | -1.06116 | 2.14E-07 | 0.000202 | DOWN |
| ENSG00000175445 | LPL | -1.06181 | 7.76E-08 | 0.000113 | DOWN |
| ENSG00000132912 | DCTN4 | -1.06216 | 0.00015 | 0.008296 | DOWN |
| ENSG00000162980 | ARL5A | -1.06325 | 1.39E-08 | 4.24E-05 | DOWN |
| ENSG00000116752 | BCAS2 | -1.06384 | 1.87E-05 | 0.002548 | DOWN |
| ENSG00000160216 | AGPAT3 | -1.06457 | 4.83E-09 | 2.32E-05 | DOWN |
| ENSG00000182220 | ATP6AP2 | -1.06471 | 1.60E-06 | 0.00063 | DOWN |
| ENSG00000085224 | ATRX | -1.06556 | 6.06E-06 | 0.001342 | DOWN |
| ENSG00000163319 | MRPS18C | -1.06695 | 1.09E-09 | 1.02E-05 | DOWN |
| ENSG00000160716 | CHRNB2 | -1.06729 | 9.39E-05 | 0.006372 | DOWN |
| ENSG00000211450 | C11orf31 | -1.06769 | 0.001219 | 0.026815 | DOWN |
| ENSG00000129128 | SPCS3 | -1.06774 | 1.50E-10 | 3.25E-06 | DOWN |
| ENSG00000163312 | HELQ | -1.06823 | 4.64E-09 | 2.28E-05 | DOWN |
| ENSG00000171791 | BCL2 | -1.0684 | 2.51E-08 | 5.96E-05 | DOWN |
| ENSG00000163754 | GYG1 | -1.07084 | 0.000265 | 0.011424 | DOWN |
| ENSG00000175324 | LSM1 | -1.0719 | 1.53E-06 | 0.000614 | DOWN |
| ENSG00000256771 | ZNF253 | -1.07211 | 5.69E-07 | 0.000349 | DOWN |
| ENSG00000103490 | PYCARD | -1.07221 | 0.001453 | 0.02956 | DOWN |
| ENSG00000156103 | MMP16 | -1.07231 | 2.61E-07 | 0.000225 | DOWN |
| ENSG00000085831 | TTC39A | -1.07264 | 2.47E-07 | 0.000218 | DOWN |
| ENSG00000147853 | AK3 | -1.07306 | 5.67E-05 | 0.004768 | DOWN |
| ENSG00000187699 | C2orf88 | -1.07309 | 0.001431 | 0.029308 | DOWN |
| ENSG00000162444 | RBP7 | -1.07355 | 0.000426 | 0.01493 | DOWN |
| ENSG00000078098 | FAP | -1.07493 | 8.87E-07 | 0.000451 | DOWN |
| ENSG00000121152 | NCAPH | -1.07562 | 1.76E-05 | 0.002461 | DOWN |
| ENSG00000109756 | RAPGEF2 | -1.07608 | 5.64E-06 | 0.001286 | DOWN |
| ENSG00000076258 | FMO4 | -1.07623 | 8.41E-05 | 0.005991 | DOWN |
| ENSG00000097033 | SH3GLB1 | -1.07637 | 3.92E-09 | 2.08E-05 | DOWN |
| ENSG00000111142 | METAP2 | -1.07693 | 2.98E-09 | 1.79E-05 | DOWN |
| ENSG00000135218 | CD36 | -1.07733 | 8.59E-06 | 0.001644 | DOWN |
| ENSG00000112139 | MDGA1 | -1.0781 | 3.04E-05 | 0.003348 | DOWN |
| ENSG00000139163 | ETNK1 | -1.07939 | 4.49E-09 | 2.24E-05 | DOWN |
| ENSG00000198435 | NRARP | -1.08054 | 0.001903 | 0.034284 | DOWN |
| ENSG00000108839 | ALOX12 | -1.08055 | 2.41E-05 | 0.002938 | DOWN |
| ENSG00000169504 | CLIC4 | -1.0806 | 1.81E-05 | 0.002503 | DOWN |
| ENSG00000171396 | KRTAP4-4 | -1.08184 | 1.11E-11 | 7.20E-07 | DOWN |
| ENSG00000163563 | MNDA | -1.08186 | 0.000147 | 0.008204 | DOWN |
| ENSG00000197128 | ZNF772 | -1.08247 | 1.83E-12 | 2.63E-07 | DOWN |
| ENSG00000151247 | EIF4E | -1.08255 | 2.07E-07 | 0.000199 | DOWN |
| ENSG00000171532 | NEUROD2 | -1.08302 | 0.000498 | 0.016274 | DOWN |
| ENSG00000133731 | IMPA1 | -1.0839 | 1.38E-08 | 4.23E-05 | DOWN |
| ENSG00000125266 | EFNB2 | -1.08467 | 7.21E-05 | 0.005467 | DOWN |
| ENSG00000126775 | ATG14 | -1.08529 | 6.58E-06 | 0.001406 | DOWN |
| ENSG00000124587 | PEX6 | -1.0854 | 2.69E-05 | 0.003125 | DOWN |
| ENSG00000029993 | HMGB3 | -1.08583 | 0.000372 | 0.013832 | DOWN |
| ENSG00000066557 | LRRC40 | -1.08807 | 4.42E-08 | 8.16E-05 | DOWN |
| ENSG00000188313 | PLSCR1 | -1.08825 | 0.000117 | 0.007212 | DOWN |
| ENSG00000257017 | HP | -1.08826 | 1.09E-06 | 0.000507 | DOWN |
| ENSG00000185947 | ZNF267 | -1.08866 | 7.59E-13 | 1.61E-07 | DOWN |
| ENSG00000122591 | FAM126A | -1.08892 | 2.37E-10 | 4.20E-06 | DOWN |
| ENSG00000073921 | PICALM | -1.08937 | 1.59E-09 | 1.26E-05 | DOWN |
| ENSG00000254004 | ZNF260 | -1.08964 | 1.24E-05 | 0.002026 | DOWN |
| ENSG00000161888 | SPC24 | -1.08983 | 0.000157 | 0.008531 | DOWN |
| ENSG00000085231 | AK6 | -1.09041 | 0.000186 | 0.009373 | DOWN |
| ENSG00000181315 | ZNF322 | -1.09052 | 2.78E-05 | 0.003188 | DOWN |
| ENSG00000131831 | RAI2 | -1.09067 | 0.000545 | 0.017159 | DOWN |
| ENSG00000162692 | VCAM1 | -1.09067 | 5.60E-05 | 0.004737 | DOWN |
| ENSG00000147592 | LACTB2 | -1.09178 | 6.14E-05 | 0.004992 | DOWN |
| ENSG00000171202 | TMEM126A | -1.09268 | 4.68E-06 | 0.001157 | DOWN |
| ENSG00000169251 | NMD3 | -1.09308 | 4.68E-09 | 2.28E-05 | DOWN |
| ENSG00000250264 | XXbac-BPG246D15.9 | -1.09323 | 1.01E-09 | 9.77E-06 | DOWN |
| ENSG00000120533 | ENY2 | -1.09391 | 4.58E-15 | 9.04E-09 | DOWN |
| ENSG00000179476 | C14orf28 | -1.09442 | 5.07E-05 | 0.004479 | DOWN |
| ENSG00000131845 | ZNF304 | -1.0945 | 1.96E-09 | 1.42E-05 | DOWN |
| ENSG00000166801 | FAM111A | -1.09476 | 9.16E-07 | 0.000459 | DOWN |
| ENSG00000124098 | FAM210B | -1.09528 | 0.00015 | 0.008278 | DOWN |
| ENSG00000104432 | IL7 | -1.096 | 0.000147 | 0.008187 | DOWN |
| ENSG00000047056 | WDR37 | -1.09703 | 4.30E-14 | 3.37E-08 | DOWN |
| ENSG00000107758 | PPP3CB | -1.09765 | 9.95E-07 | 0.000482 | DOWN |
| ENSG00000109270 | LAMTOR3 | -1.09777 | 3.64E-06 | 0.001005 | DOWN |
| ENSG00000174842 | GLMN | -1.09777 | 0.00039 | 0.014208 | DOWN |
| ENSG00000021574 | SPAST | -1.09788 | 2.51E-11 | 1.16E-06 | DOWN |
| ENSG00000204388 | HSPA1B | -1.09865 | 0.002906 | 0.043472 | DOWN |
| ENSG00000173451 | THAP2 | -1.09968 | 3.62E-07 | 0.000271 | DOWN |
| ENSG00000152484 | USP12 | -1.10031 | 2.74E-05 | 0.003164 | DOWN |
| ENSG00000143933 | CALM2 | -1.10093 | 5.46E-06 | 0.001262 | DOWN |
| ENSG00000178605 | GTPBP6 | -1.10093 | 2.14E-05 | 0.002752 | DOWN |
| ENSG00000213047 | DENND1B | -1.10107 | 1.44E-08 | 4.32E-05 | DOWN |
| ENSG00000126777 | KTN1 | -1.10141 | 1.07E-06 | 0.000501 | DOWN |
| ENSG00000184786 | TCTE3 | -1.10168 | 4.79E-05 | 0.004327 | DOWN |
| ENSG00000205155 | PSENEN | -1.10213 | 1.34E-07 | 0.000154 | DOWN |
| ENSG00000101782 | RIOK3 | -1.10342 | 0.000455 | 0.015465 | DOWN |
| ENSG00000076554 | TPD52 | -1.1046 | 7.80E-05 | 0.005726 | DOWN |
| ENSG00000260861 | RP4-576H24.4 | -1.1049 | 2.31E-09 | 1.55E-05 | DOWN |
| ENSG00000104756 | KCTD9 | -1.10492 | 1.15E-06 | 0.000523 | DOWN |
| ENSG00000111843 | TMEM14C | -1.10566 | 0.0004 | 0.014423 | DOWN |
| ENSG00000109819 | PPARGC1A | -1.10646 | 1.06E-09 | 9.98E-06 | DOWN |
| ENSG00000275052 | PPP4R3B | -1.10658 | 2.40E-09 | 1.58E-05 | DOWN |
| ENSG00000164466 | SFXN1 | -1.1069 | 0.000987 | 0.023822 | DOWN |
| ENSG00000177485 | ZBTB33 | -1.10692 | 4.94E-09 | 2.35E-05 | DOWN |
| ENSG00000198839 | ZNF277 | -1.10709 | 2.81E-13 | 9.56E-08 | DOWN |
| ENSG00000166710 | B2M | -1.10742 | 1.90E-12 | 2.68E-07 | DOWN |
| ENSG00000134294 | SLC38A2 | -1.1075 | 1.32E-08 | 4.12E-05 | DOWN |
| ENSG00000180257 | ZNF816 | -1.10808 | 1.02E-05 | 0.001811 | DOWN |
| ENSG00000109576 | AADAT | -1.11022 | 1.01E-07 | 0.000132 | DOWN |
| ENSG00000109458 | GAB1 | -1.11036 | 1.58E-05 | 0.002322 | DOWN |
| ENSG00000163220 | S100A9 | -1.1106 | 9.56E-05 | 0.006443 | DOWN |
| ENSG00000166037 | CEP57 | -1.11086 | 3.73E-11 | 1.45E-06 | DOWN |
| ENSG00000134419 | RPS15A | -1.111 | 0.00049 | 0.016133 | DOWN |
| ENSG00000180817 | PPA1 | -1.11249 | 0.000205 | 0.009928 | DOWN |
| ENSG00000186908 | ZDHHC17 | -1.11356 | 3.75E-07 | 0.000276 | DOWN |
| ENSG00000158863 | FAM160B2 | -1.11492 | 1.94E-09 | 1.41E-05 | DOWN |
| ENSG00000170631 | ZNF16 | -1.11496 | 0.000518 | 0.01665 | DOWN |
| ENSG00000106689 | LHX2 | -1.11536 | 0.000645 | 0.018783 | DOWN |
| ENSG00000152256 | PDK1 | -1.11552 | 1.07E-07 | 0.000136 | DOWN |
| ENSG00000137871 | ZNF280D | -1.11601 | 1.45E-08 | 4.33E-05 | DOWN |
| ENSG00000197976 | AKAP17A | -1.11639 | 5.01E-06 | 0.001202 | DOWN |
| ENSG00000186130 | ZBTB6 | -1.11648 | 1.51E-06 | 0.00061 | DOWN |
| ENSG00000239642 | MEIKIN | -1.11697 | 5.83E-08 | 9.59E-05 | DOWN |
| ENSG00000103534 | TMC5 | -1.11741 | 0.001727 | 0.032491 | DOWN |
| ENSG00000112245 | PTP4A1 | -1.11814 | 6.80E-07 | 0.000387 | DOWN |
| ENSG00000142669 | SH3BGRL3 | -1.11873 | 1.27E-07 | 0.00015 | DOWN |
| ENSG00000273542 | HIST1H4K | -1.11915 | 1.49E-05 | 0.002243 | DOWN |
| ENSG00000159263 | SIM2 | -1.11968 | 8.07E-17 | 8.48E-10 | DOWN |
| ENSG00000181781 | ODF3L2 | -1.12055 | 6.97E-09 | 2.87E-05 | DOWN |
| ENSG00000164209 | SLC25A46 | -1.12111 | 3.94E-12 | 4.09E-07 | DOWN |
| ENSG00000100916 | BRMS1L | -1.1213 | 0.002247 | 0.037623 | DOWN |
| ENSG00000142453 | CARM1 | -1.12216 | 1.60E-08 | 4.59E-05 | DOWN |
| ENSG00000171757 | LRRC34 | -1.12365 | 0.000148 | 0.008221 | DOWN |
| ENSG00000013288 | MAN2B2 | -1.12464 | 4.69E-10 | 6.27E-06 | DOWN |
| ENSG00000116221 | MRPL37 | -1.12509 | 2.54E-07 | 0.000222 | DOWN |
| ENSG00000005469 | CROT | -1.12587 | 1.57E-12 | 2.40E-07 | DOWN |
| ENSG00000198924 | DCLRE1A | -1.12761 | 3.71E-06 | 0.001015 | DOWN |
| ENSG00000242419 | PCDHGC4 | -1.12861 | 2.50E-05 | 0.003005 | DOWN |
| ENSG00000163156 | SCNM1 | -1.12897 | 8.24E-06 | 0.001605 | DOWN |
| ENSG00000197479 | PCDHB11 | -1.12897 | 2.21E-05 | 0.002805 | DOWN |
| ENSG00000213186 | TRIM59 | -1.13004 | 9.59E-08 | 0.000128 | DOWN |
| ENSG00000075790 | BCAP29 | -1.13103 | 1.30E-05 | 0.002081 | DOWN |
| ENSG00000113460 | BRIX1 | -1.13118 | 3.13E-08 | 6.75E-05 | DOWN |
| ENSG00000183130 | OR2T11 | -1.13133 | 9.40E-07 | 0.000466 | DOWN |
| ENSG00000196437 | ZNF569 | -1.13257 | 2.63E-08 | 6.11E-05 | DOWN |
| ENSG00000164346 | NSA2 | -1.13354 | 8.43E-06 | 0.001625 | DOWN |
| ENSG00000017427 | IGF1 | -1.13519 | 5.06E-07 | 0.000327 | DOWN |
| ENSG00000101266 | CSNK2A1 | -1.13568 | 1.70E-06 | 0.000651 | DOWN |
| ENSG00000163827 | LRRC2 | -1.13569 | 0.001377 | 0.028702 | DOWN |
| ENSG00000115138 | POMC | -1.1357 | 0.002292 | 0.038056 | DOWN |
| ENSG00000028116 | VRK2 | -1.1358 | 5.85E-08 | 9.61E-05 | DOWN |
| ENSG00000154114 | TBCEL | -1.13586 | 2.36E-05 | 0.002903 | DOWN |
| ENSG00000173660 | UQCRH | -1.13664 | 3.18E-09 | 1.87E-05 | DOWN |
| ENSG00000169398 | PTK2 | -1.13702 | 1.02E-09 | 9.77E-06 | DOWN |
| ENSG00000112159 | MDN1 | -1.13802 | 6.72E-09 | 2.81E-05 | DOWN |
| ENSG00000164953 | TMEM67 | -1.13827 | 1.64E-05 | 0.002369 | DOWN |
| ENSG00000110696 | C11orf58 | -1.13866 | 3.50E-07 | 0.000266 | DOWN |
| ENSG00000115758 | ODC1 | -1.13987 | 7.75E-07 | 0.000417 | DOWN |
| ENSG00000141232 | TOB1 | -1.14052 | 8.84E-06 | 0.001672 | DOWN |
| ENSG00000119446 | RBM18 | -1.14214 | 2.46E-06 | 0.000803 | DOWN |
| ENSG00000197601 | FAR1 | -1.14223 | 6.84E-07 | 0.000389 | DOWN |
| ENSG00000136856 | SLC2A8 | -1.14347 | 1.08E-09 | 1.01E-05 | DOWN |
| ENSG00000205730 | ITPRIPL2 | -1.14354 | 1.88E-10 | 3.73E-06 | DOWN |
| ENSG00000163501 | IHH | -1.14357 | 7.04E-05 | 0.005396 | DOWN |
| ENSG00000182698 | RESP18 | -1.14395 | 0.00116 | 0.026103 | DOWN |
| ENSG00000138468 | SENP7 | -1.14436 | 5.43E-05 | 0.004661 | DOWN |
| ENSG00000184304 | PRKD1 | -1.14468 | 4.81E-06 | 0.001175 | DOWN |
| ENSG00000006042 | TMEM98 | -1.14596 | 9.07E-06 | 0.001697 | DOWN |
| ENSG00000184611 | KCNH7 | -1.14639 | 0.003213 | 0.045974 | DOWN |
| ENSG00000048405 | ZNF800 | -1.1465 | 9.38E-11 | 2.48E-06 | DOWN |
| ENSG00000101161 | PRPF6 | -1.1469 | 2.21E-07 | 0.000206 | DOWN |
| ENSG00000078061 | ARAF | -1.14746 | 1.59E-06 | 0.00063 | DOWN |
| ENSG00000134056 | MRPS36 | -1.14803 | 3.65E-09 | 2.01E-05 | DOWN |
| ENSG00000203814 | HIST2H2BF | -1.14813 | 6.20E-13 | 1.45E-07 | DOWN |
| ENSG00000165704 | HPRT1 | -1.14885 | 3.26E-05 | 0.003483 | DOWN |
| ENSG00000096872 | IFT74 | -1.15014 | 7.07E-08 | 0.000108 | DOWN |
| ENSG00000166527 | CLEC4D | -1.15021 | 8.43E-05 | 0.005996 | DOWN |
| ENSG00000139350 | NEDD1 | -1.15119 | 0.000163 | 0.008707 | DOWN |
| ENSG00000118564 | FBXL5 | -1.1515 | 1.90E-07 | 0.000189 | DOWN |
| ENSG00000130517 | PGPEP1 | -1.15151 | 3.91E-08 | 7.62E-05 | DOWN |
| ENSG00000149212 | SESN3 | -1.153 | 9.64E-08 | 0.000128 | DOWN |
| ENSG00000064933 | PMS1 | -1.15368 | 3.58E-11 | 1.42E-06 | DOWN |
| ENSG00000097046 | CDC7 | -1.15539 | 3.28E-07 | 0.000256 | DOWN |
| ENSG00000197444 | OGDHL | -1.15643 | 4.57E-05 | 0.004212 | DOWN |
| ENSG00000187514 | PTMA | -1.15693 | 8.56E-05 | 0.006053 | DOWN |
| ENSG00000163029 | SMC6 | -1.15769 | 7.11E-08 | 0.000108 | DOWN |
| ENSG00000197782 | ZNF780A | -1.15805 | 6.81E-09 | 2.82E-05 | DOWN |
| ENSG00000198918 | RPL39 | -1.15817 | 3.15E-10 | 4.95E-06 | DOWN |
| ENSG00000138604 | GLCE | -1.15818 | 1.11E-09 | 1.02E-05 | DOWN |
| ENSG00000165025 | SYK | -1.1614 | 1.95E-09 | 1.41E-05 | DOWN |
| ENSG00000111605 | CPSF6 | -1.16214 | 5.31E-11 | 1.79E-06 | DOWN |
| ENSG00000152455 | SUV39H2 | -1.16226 | 3.61E-07 | 0.000271 | DOWN |
| ENSG00000109929 | SC5D | -1.1628 | 5.22E-08 | 8.99E-05 | DOWN |
| ENSG00000221837 | KRTAP10-9 | -1.16306 | 5.51E-05 | 0.004703 | DOWN |
| ENSG00000107537 | PHYH | -1.16402 | 0.000741 | 0.020307 | DOWN |
| ENSG00000103150 | MLYCD | -1.1642 | 0.000814 | 0.021423 | DOWN |
| ENSG00000155097 | ATP6V1C1 | -1.16643 | 4.05E-08 | 7.79E-05 | DOWN |
| ENSG00000154640 | BTG3 | -1.16645 | 1.10E-05 | 0.001896 | DOWN |
| ENSG00000068878 | PSME4 | -1.16688 | 0.000171 | 0.008954 | DOWN |
| ENSG00000110888 | CAPRIN2 | -1.16746 | 0.000102 | 0.006664 | DOWN |
| ENSG00000104231 | ZFAND1 | -1.16798 | 4.77E-09 | 2.31E-05 | DOWN |
| ENSG00000155100 | OTUD6B | -1.16971 | 3.71E-08 | 7.42E-05 | DOWN |
| ENSG00000012048 | BRCA1 | -1.1698 | 1.98E-07 | 0.000194 | DOWN |
| ENSG00000182141 | ZNF708 | -1.17073 | 1.43E-07 | 0.00016 | DOWN |
| ENSG00000259494 | MRPL46 | -1.17136 | 0.000149 | 0.008266 | DOWN |
| ENSG00000109832 | DDX25 | -1.1724 | 2.56E-07 | 0.000223 | DOWN |
| ENSG00000146278 | PNRC1 | -1.17312 | 1.71E-07 | 0.000178 | DOWN |
| ENSG00000221870 | TMEM257 | -1.17318 | 0.000244 | 0.01092 | DOWN |
| ENSG00000135049 | AGTPBP1 | -1.17421 | 4.07E-09 | 2.13E-05 | DOWN |
| ENSG00000134248 | LAMTOR5 | -1.17521 | 0.000102 | 0.006664 | DOWN |
| ENSG00000143977 | SNRPG | -1.17613 | 9.49E-05 | 0.006415 | DOWN |
| ENSG00000147905 | ZCCHC7 | -1.17662 | 5.75E-09 | 2.56E-05 | DOWN |
| ENSG00000111261 | MANSC1 | -1.17693 | 2.57E-07 | 0.000223 | DOWN |
| ENSG00000197933 | ZNF823 | -1.17752 | 2.49E-05 | 0.002994 | DOWN |
| ENSG00000172264 | MACROD2 | -1.17803 | 0.000144 | 0.008098 | DOWN |
| ENSG00000115514 | TXNDC9 | -1.17822 | 4.48E-09 | 2.24E-05 | DOWN |
| ENSG00000165682 | CLEC1B | -1.17994 | 0.000653 | 0.018901 | DOWN |
| ENSG00000109736 | MFSD10 | -1.18102 | 3.47E-10 | 5.24E-06 | DOWN |
| ENSG00000163320 | CGGBP1 | -1.18169 | 1.01E-07 | 0.000132 | DOWN |
| ENSG00000169258 | GPRIN1 | -1.18278 | 0.001983 | 0.035071 | DOWN |
| ENSG00000113389 | NPR3 | -1.18304 | 2.37E-05 | 0.002916 | DOWN |
| ENSG00000136897 | MRPL50 | -1.18305 | 1.55E-08 | 4.50E-05 | DOWN |
| ENSG00000013441 | CLK1 | -1.18306 | 4.63E-06 | 0.001149 | DOWN |
| ENSG00000156831 | NSMCE2 | -1.18319 | 3.70E-08 | 7.41E-05 | DOWN |
| ENSG00000151065 | DCP1B | -1.18355 | 1.68E-11 | 9.19E-07 | DOWN |
| ENSG00000184166 | OR1D2 | -1.18488 | 0.000453 | 0.015428 | DOWN |
| ENSG00000168803 | ADAL | -1.18607 | 3.57E-09 | 1.99E-05 | DOWN |
| ENSG00000005249 | PRKAR2B | -1.18754 | 0.000102 | 0.006689 | DOWN |
| ENSG00000025156 | HSF2 | -1.18909 | 1.46E-05 | 0.002224 | DOWN |
| ENSG00000126858 | RHOT1 | -1.18921 | 1.47E-08 | 4.37E-05 | DOWN |
| ENSG00000198873 | GRK5 | -1.19014 | 0.000217 | 0.010231 | DOWN |
| ENSG00000132688 | NES | -1.19069 | 4.60E-08 | 8.36E-05 | DOWN |
| ENSG00000050327 | ARHGEF5 | -1.19095 | 4.57E-09 | 2.26E-05 | DOWN |
| ENSG00000150630 | VEGFC | -1.19273 | 1.18E-11 | 7.46E-07 | DOWN |
| ENSG00000124126 | PREX1 | -1.19352 | 3.75E-08 | 7.46E-05 | DOWN |
| ENSG00000117595 | IRF6 | -1.19472 | 1.77E-05 | 0.002474 | DOWN |
| ENSG00000258653 | RP5-1021I20.4 | -1.19488 | 9.42E-10 | 9.39E-06 | DOWN |
| ENSG00000134146 | DPH6 | -1.19553 | 2.28E-08 | 5.67E-05 | DOWN |
| ENSG00000186314 | PRELID2 | -1.19621 | 1.58E-06 | 0.000627 | DOWN |
| ENSG00000144959 | NCEH1 | -1.1963 | 0.00014 | 0.007952 | DOWN |
| ENSG00000106537 | TSPAN13 | -1.19752 | 1.64E-05 | 0.002375 | DOWN |
| ENSG00000146243 | IRAK1BP1 | -1.19774 | 4.71E-06 | 0.00116 | DOWN |
| ENSG00000188761 | BCL2L15 | -1.2001 | 0.000135 | 0.007806 | DOWN |
| ENSG00000057663 | ATG5 | -1.20086 | 8.25E-12 | 6.14E-07 | DOWN |
| ENSG00000076043 | REXO2 | -1.20122 | 6.75E-08 | 0.000105 | DOWN |
| ENSG00000255837 | TAS2R20 | -1.20153 | 0.003481 | 0.048134 | DOWN |
| ENSG00000116266 | STXBP3 | -1.20224 | 7.37E-10 | 8.16E-06 | DOWN |
| ENSG00000101236 | RNF24 | -1.20289 | 4.94E-06 | 0.001192 | DOWN |
| ENSG00000156711 | MAPK13 | -1.2038 | 0.001179 | 0.026323 | DOWN |
| ENSG00000137575 | SDCBP | -1.20453 | 2.96E-07 | 0.000241 | DOWN |
| ENSG00000156411 | C14orf2 | -1.20538 | 5.20E-07 | 0.000332 | DOWN |
| ENSG00000108813 | DLX4 | -1.20545 | 0.001184 | 0.026381 | DOWN |
| ENSG00000172172 | MRPL13 | -1.20554 | 7.94E-09 | 3.08E-05 | DOWN |
| ENSG00000136603 | SKIL | -1.20607 | 6.68E-09 | 2.80E-05 | DOWN |
| ENSG00000164163 | ABCE1 | -1.20676 | 2.37E-07 | 0.000213 | DOWN |
| ENSG00000123297 | TSFM | -1.20709 | 1.34E-06 | 0.000573 | DOWN |
| ENSG00000269343 | ZNF587B | -1.20715 | 1.18E-10 | 2.82E-06 | DOWN |
| ENSG00000180628 | PCGF5 | -1.20725 | 4.74E-05 | 0.004299 | DOWN |
| ENSG00000188816 | HMX2 | -1.20746 | 1.23E-06 | 0.000544 | DOWN |
| ENSG00000162399 | BSND | -1.20813 | 3.17E-13 | 1.01E-07 | DOWN |
| ENSG00000145425 | RPS3A | -1.21008 | 1.96E-05 | 0.002614 | DOWN |
| ENSG00000008952 | SEC62 | -1.21124 | 1.28E-06 | 0.000557 | DOWN |
| ENSG00000109686 | SH3D19 | -1.21171 | 1.74E-09 | 1.33E-05 | DOWN |
| ENSG00000167232 | ZNF91 | -1.21183 | 5.78E-07 | 0.000352 | DOWN |
| ENSG00000176782 | DEFB104A | -1.21351 | 8.32E-12 | 6.16E-07 | DOWN |
| ENSG00000197980 | LEKR1 | -1.21367 | 0.002836 | 0.042881 | DOWN |
| ENSG00000105245 | NUMBL | -1.21446 | 1.53E-07 | 0.000166 | DOWN |
| ENSG00000162775 | RBM15 | -1.21472 | 2.11E-06 | 0.000735 | DOWN |
| ENSG00000169508 | GPR183 | -1.21505 | 3.70E-11 | 1.45E-06 | DOWN |
| ENSG00000136021 | SCYL2 | -1.21923 | 9.95E-10 | 9.69E-06 | DOWN |
| ENSG00000122958 | VPS26A | -1.22099 | 1.83E-06 | 0.00068 | DOWN |
| ENSG00000164078 | MST1R | -1.22185 | 2.29E-08 | 5.68E-05 | DOWN |
| ENSG00000143183 | TMCO1 | -1.22323 | 1.27E-07 | 0.00015 | DOWN |
| ENSG00000131127 | ZNF141 | -1.22366 | 3.84E-06 | 0.001032 | DOWN |
| ENSG00000164603 | C7orf60 | -1.22573 | 5.22E-13 | 1.33E-07 | DOWN |
| ENSG00000132963 | POMP | -1.22578 | 4.71E-14 | 3.55E-08 | DOWN |
| ENSG00000009950 | MLXIPL | -1.22622 | 7.29E-09 | 2.94E-05 | DOWN |
| ENSG00000118523 | CTGF | -1.22661 | 1.80E-05 | 0.002496 | DOWN |
| ENSG00000115760 | BIRC6 | -1.22818 | 8.05E-09 | 3.11E-05 | DOWN |
| ENSG00000204435 | CSNK2B | -1.22838 | 0.000732 | 0.020165 | DOWN |
| ENSG00000151229 | SLC2A13 | -1.22876 | 0.001659 | 0.031816 | DOWN |
| ENSG00000174021 | GNG5 | -1.22988 | 1.92E-13 | 7.70E-08 | DOWN |
| ENSG00000004846 | ABCB5 | -1.23039 | 3.27E-06 | 0.000947 | DOWN |
| ENSG00000174951 | FUT1 | -1.23105 | 0.00116 | 0.026103 | DOWN |
| ENSG00000132465 | JCHAIN | -1.23269 | 3.13E-05 | 0.003404 | DOWN |
| ENSG00000108312 | UBTF | -1.23397 | 1.99E-06 | 0.000712 | DOWN |
| ENSG00000172469 | MANEA | -1.23409 | 2.39E-09 | 1.58E-05 | DOWN |
| ENSG00000104408 | EIF3E | -1.23437 | 2.47E-15 | 6.31E-09 | DOWN |
| ENSG00000134265 | NAPG | -1.23442 | 5.81E-08 | 9.57E-05 | DOWN |
| ENSG00000133393 | FOPNL | -1.23584 | 3.15E-07 | 0.00025 | DOWN |
| ENSG00000241945 | PWP2 | -1.23691 | 2.29E-07 | 0.00021 | DOWN |
| ENSG00000165733 | BMS1 | -1.23815 | 2.35E-10 | 4.19E-06 | DOWN |
| ENSG00000173442 | EHBP1L1 | -1.23989 | 3.37E-05 | 0.003548 | DOWN |
| ENSG00000112115 | IL17A | -1.24094 | 5.78E-13 | 1.40E-07 | DOWN |
| ENSG00000153044 | CENPH | -1.24222 | 6.40E-05 | 0.005118 | DOWN |
| ENSG00000164066 | INTU | -1.24279 | 3.91E-05 | 0.003852 | DOWN |
| ENSG00000112742 | TTK | -1.24299 | 4.78E-07 | 0.000317 | DOWN |
| ENSG00000083937 | CHMP2B | -1.24312 | 3.20E-10 | 4.99E-06 | DOWN |
| ENSG00000185652 | NTF3 | -1.24611 | 8.15E-07 | 0.000429 | DOWN |
| ENSG00000074842 | MYDGF | -1.2468 | 1.28E-06 | 0.000556 | DOWN |
| ENSG00000163291 | PAQR3 | -1.24826 | 9.89E-06 | 0.001784 | DOWN |
| ENSG00000165487 | MICU2 | -1.24913 | 2.08E-09 | 1.46E-05 | DOWN |
| ENSG00000176105 | YES1 | -1.24981 | 2.63E-09 | 1.68E-05 | DOWN |
| ENSG00000169877 | AHSP | -1.24998 | 1.64E-05 | 0.002369 | DOWN |
| ENSG00000174405 | LIG4 | -1.25042 | 1.09E-08 | 3.68E-05 | DOWN |
| ENSG00000182512 | GLRX5 | -1.25257 | 2.66E-06 | 0.000839 | DOWN |
| ENSG00000168876 | ANKRD49 | -1.25724 | 2.86E-08 | 6.40E-05 | DOWN |
| ENSG00000166479 | TMX3 | -1.25885 | 7.87E-08 | 0.000114 | DOWN |
| ENSG00000115947 | ORC4 | -1.26099 | 5.96E-13 | 1.42E-07 | DOWN |
| ENSG00000120952 | PRAMEF2 | -1.26151 | 2.13E-06 | 0.000739 | DOWN |
| ENSG00000186566 | GPATCH8 | -1.26151 | 1.76E-08 | 4.86E-05 | DOWN |
| ENSG00000198804 | MT-CO1 | -1.26241 | 7.41E-07 | 0.000407 | DOWN |
| ENSG00000123975 | CKS2 | -1.26368 | 6.43E-06 | 0.001387 | DOWN |
| ENSG00000170515 | PA2G4 | -1.26399 | 2.64E-10 | 4.46E-06 | DOWN |
| ENSG00000168175 | MAPK1IP1L | -1.26472 | 5.57E-06 | 0.001276 | DOWN |
| ENSG00000146587 | RBAK | -1.26689 | 2.13E-10 | 3.96E-06 | DOWN |
| ENSG00000147400 | CETN2 | -1.26812 | 2.95E-06 | 0.000891 | DOWN |
| ENSG00000101972 | STAG2 | -1.26834 | 3.82E-12 | 4.04E-07 | DOWN |
| ENSG00000124172 | ATP5E | -1.26905 | 5.74E-11 | 1.86E-06 | DOWN |
| ENSG00000100614 | PPM1A | -1.26922 | 1.30E-05 | 0.002079 | DOWN |
| ENSG00000173598 | NUDT4 | -1.27001 | 3.11E-05 | 0.003392 | DOWN |
| ENSG00000134779 | TPGS2 | -1.27236 | 8.71E-06 | 0.001658 | DOWN |
| ENSG00000156970 | BUB1B | -1.27238 | 1.96E-06 | 0.000707 | DOWN |
| ENSG00000179271 | GADD45GIP1 | -1.27259 | 1.68E-12 | 2.49E-07 | DOWN |
| ENSG00000198168 | SVIP | -1.27351 | 3.30E-12 | 3.70E-07 | DOWN |
| ENSG00000183735 | TBK1 | -1.27448 | 2.31E-09 | 1.55E-05 | DOWN |
| ENSG00000109184 | DCUN1D4 | -1.27617 | 3.21E-09 | 1.87E-05 | DOWN |
| ENSG00000165813 | CCDC186 | -1.27857 | 9.77E-12 | 6.77E-07 | DOWN |
| ENSG00000204186 | ZDBF2 | -1.27893 | 7.33E-07 | 0.000404 | DOWN |
| ENSG00000155506 | LARP1 | -1.28088 | 1.46E-12 | 2.33E-07 | DOWN |
| ENSG00000057935 | MTA3 | -1.28141 | 3.44E-07 | 0.000264 | DOWN |
| ENSG00000272104 | XXcos-LUCA11.5 | -1.28164 | 7.38E-05 | 0.005547 | DOWN |
| ENSG00000165985 | C1QL3 | -1.28285 | 0.000942 | 0.023216 | DOWN |
| ENSG00000137648 | TMPRSS4 | -1.28287 | 0.001638 | 0.031583 | DOWN |
| ENSG00000157766 | ACAN | -1.28315 | 5.69E-07 | 0.000349 | DOWN |
| ENSG00000163806 | SPDYA | -1.28527 | 0.000275 | 0.011658 | DOWN |
| ENSG00000187118 | CMC1 | -1.28555 | 0.000116 | 0.007182 | DOWN |
| ENSG00000108406 | DHX40 | -1.28593 | 2.88E-05 | 0.003251 | DOWN |
| ENSG00000257108 | NHLRC4 | -1.28736 | 1.94E-14 | 2.11E-08 | DOWN |
| ENSG00000123610 | TNFAIP6 | -1.28763 | 1.97E-06 | 0.000708 | DOWN |
| ENSG00000140319 | SRP14 | -1.28781 | 1.31E-07 | 0.000152 | DOWN |
| ENSG00000196352 | CD55 | -1.28839 | 4.37E-05 | 0.004111 | DOWN |
| ENSG00000188672 | RHCE | -1.28845 | 0.000458 | 0.015524 | DOWN |
| ENSG00000113638 | TTC33 | -1.28852 | 5.84E-09 | 2.58E-05 | DOWN |
| ENSG00000127184 | COX7C | -1.28862 | 6.44E-05 | 0.005134 | DOWN |
| ENSG00000034677 | RNF19A | -1.28876 | 2.72E-06 | 0.000851 | DOWN |
| ENSG00000171806 | METTL18 | -1.28984 | 3.52E-07 | 0.000267 | DOWN |
| ENSG00000107890 | ANKRD26 | -1.29022 | 1.77E-09 | 1.34E-05 | DOWN |
| ENSG00000101624 | CEP76 | -1.29118 | 5.45E-07 | 0.00034 | DOWN |
| ENSG00000183291 | 15-Sep | -1.29164 | 9.37E-12 | 6.60E-07 | DOWN |
| ENSG00000204592 | HLA-E | -1.29165 | 3.51E-11 | 1.41E-06 | DOWN |
| ENSG00000119013 | NDUFB3 | -1.29246 | 8.73E-09 | 3.24E-05 | DOWN |
| ENSG00000120942 | UBIAD1 | -1.29254 | 0.000486 | 0.016067 | DOWN |
| ENSG00000156171 | DRAM2 | -1.29331 | 8.11E-14 | 4.77E-08 | DOWN |
| ENSG00000147649 | MTDH | -1.29459 | 3.11E-11 | 1.31E-06 | DOWN |
| ENSG00000107562 | CXCL12 | -1.29468 | 1.91E-08 | 5.11E-05 | DOWN |
| ENSG00000088986 | DYNLL1 | -1.29478 | 0.000765 | 0.020692 | DOWN |
| ENSG00000124279 | FASTKD3 | -1.29502 | 1.52E-11 | 8.67E-07 | DOWN |
| ENSG00000088179 | PTPN4 | -1.2953 | 1.79E-08 | 4.91E-05 | DOWN |
| ENSG00000056050 | C4orf27 | -1.29553 | 9.96E-05 | 0.006591 | DOWN |
| ENSG00000183010 | PYCR1 | -1.29576 | 8.00E-07 | 0.000424 | DOWN |
| ENSG00000198901 | PRC1 | -1.29624 | 1.38E-09 | 1.16E-05 | DOWN |
| ENSG00000120662 | MTRF1 | -1.29719 | 7.43E-11 | 2.18E-06 | DOWN |
| ENSG00000009844 | VTA1 | -1.29961 | 3.56E-11 | 1.42E-06 | DOWN |
| ENSG00000144395 | CCDC150 | -1.29977 | 1.42E-05 | 0.002183 | DOWN |
| ENSG00000196636 | SDHAF3 | -1.29981 | 2.48E-08 | 5.93E-05 | DOWN |
| ENSG00000129071 | MBD4 | -1.29991 | 1.32E-11 | 7.99E-07 | DOWN |
| ENSG00000277157 | HIST1H4D | -1.30059 | 1.49E-12 | 2.35E-07 | DOWN |
| ENSG00000175348 | TMEM9B | -1.30137 | 1.56E-05 | 0.002302 | DOWN |
| ENSG00000156509 | FBXO43 | -1.30242 | 0.001356 | 0.028463 | DOWN |
| ENSG00000153993 | SEMA3D | -1.30344 | 8.24E-08 | 0.000117 | DOWN |
| ENSG00000112237 | CCNC | -1.30489 | 9.48E-11 | 2.50E-06 | DOWN |
| ENSG00000122565 | CBX3 | -1.30776 | 2.98E-14 | 2.71E-08 | DOWN |
| ENSG00000123080 | CDKN2C | -1.30877 | 3.48E-06 | 0.000979 | DOWN |
| ENSG00000152944 | MED21 | -1.30934 | 5.37E-08 | 9.15E-05 | DOWN |
| ENSG00000007372 | PAX6 | -1.30956 | 8.50E-05 | 0.006024 | DOWN |
| ENSG00000279238 | AL138706.2 | -1.31072 | 0.00329 | 0.046561 | DOWN |
| ENSG00000122691 | TWIST1 | -1.31191 | 5.05E-08 | 8.82E-05 | DOWN |
| ENSG00000139971 | C14orf37 | -1.3124 | 0.001262 | 0.027326 | DOWN |
| ENSG00000106009 | BRAT1 | -1.31352 | 9.71E-06 | 0.001765 | DOWN |
| ENSG00000041982 | TNC | -1.31685 | 5.88E-07 | 0.000356 | DOWN |
| ENSG00000149527 | PLCH2 | -1.31702 | 0.001608 | 0.03126 | DOWN |
| ENSG00000156017 | CARNMT1 | -1.31848 | 1.49E-12 | 2.35E-07 | DOWN |
| ENSG00000141682 | PMAIP1 | -1.31851 | 8.66E-05 | 0.006096 | DOWN |
| ENSG00000204682 | CASC10 | -1.3212 | 8.40E-09 | 3.17E-05 | DOWN |
| ENSG00000169714 | CNBP | -1.32138 | 8.96E-07 | 0.000453 | DOWN |
| ENSG00000118276 | B4GALT6 | -1.32296 | 0.000187 | 0.009415 | DOWN |
| ENSG00000101057 | MYBL2 | -1.32329 | 2.65E-09 | 1.68E-05 | DOWN |
| ENSG00000187240 | DYNC2H1 | -1.32404 | 2.98E-06 | 0.000896 | DOWN |
| ENSG00000112210 | RAB23 | -1.32476 | 2.11E-09 | 1.48E-05 | DOWN |
| ENSG00000180855 | ZNF443 | -1.32538 | 0.002694 | 0.04164 | DOWN |
| ENSG00000149548 | CCDC15 | -1.32628 | 0.0001 | 0.006617 | DOWN |
| ENSG00000167447 | SMG8 | -1.32675 | 2.80E-06 | 0.000865 | DOWN |
| ENSG00000101745 | ANKRD12 | -1.32743 | 5.61E-13 | 1.39E-07 | DOWN |
| ENSG00000182287 | AP1S2 | -1.32837 | 1.30E-06 | 0.000562 | DOWN |
| ENSG00000181856 | SLC2A4 | -1.32942 | 0.00023 | 0.010571 | DOWN |
| ENSG00000115128 | SF3B6 | -1.32976 | 1.87E-07 | 0.000188 | DOWN |
| ENSG00000277632 | CCL3 | -1.33108 | 7.29E-05 | 0.005507 | DOWN |
| ENSG00000065548 | ZC3H15 | -1.33157 | 9.82E-11 | 2.55E-06 | DOWN |
| ENSG00000213780 | GTF2H4 | -1.33159 | 8.29E-10 | 8.73E-06 | DOWN |
| ENSG00000134028 | ADAMDEC1 | -1.3332 | 4.21E-06 | 0.001088 | DOWN |
| ENSG00000189266 | PNRC2 | -1.33464 | 9.89E-14 | 5.24E-08 | DOWN |
| ENSG00000196792 | STRN3 | -1.33485 | 1.11E-13 | 5.58E-08 | DOWN |
| ENSG00000123485 | HJURP | -1.33497 | 0.000333 | 0.012998 | DOWN |
| ENSG00000120337 | TNFSF18 | -1.33545 | 5.42E-11 | 1.81E-06 | DOWN |
| ENSG00000160179 | ABCG1 | -1.33641 | 2.32E-11 | 1.10E-06 | DOWN |
| ENSG00000010017 | RANBP9 | -1.33806 | 5.46E-07 | 0.000341 | DOWN |
| ENSG00000170881 | RNF139 | -1.33839 | 3.75E-14 | 3.10E-08 | DOWN |
| ENSG00000117586 | TNFSF4 | -1.33861 | 1.37E-10 | 3.08E-06 | DOWN |
| ENSG00000198719 | DLL1 | -1.33921 | 0.000101 | 0.00664 | DOWN |
| ENSG00000153814 | JAZF1 | -1.3396 | 0.000168 | 0.008854 | DOWN |
| ENSG00000119922 | IFIT2 | -1.33982 | 0.001288 | 0.027614 | DOWN |
| ENSG00000060982 | BCAT1 | -1.34191 | 3.51E-08 | 7.22E-05 | DOWN |
| ENSG00000118922 | KLF12 | -1.34283 | 7.45E-05 | 0.005573 | DOWN |
| ENSG00000069869 | NEDD4 | -1.34365 | 8.71E-14 | 4.86E-08 | DOWN |
| ENSG00000164976 | KIAA1161 | -1.34425 | 1.31E-13 | 6.20E-08 | DOWN |
| ENSG00000127995 | CASD1 | -1.34527 | 2.76E-09 | 1.72E-05 | DOWN |
| ENSG00000174695 | TMEM167A | -1.34665 | 8.38E-11 | 2.33E-06 | DOWN |
| ENSG00000067221 | STOML1 | -1.34853 | 7.67E-10 | 8.34E-06 | DOWN |
| ENSG00000139793 | MBNL2 | -1.34904 | 1.43E-07 | 0.00016 | DOWN |
| ENSG00000113811 | SELK | -1.3499 | 3.20E-09 | 1.87E-05 | DOWN |
| ENSG00000128590 | DNAJB9 | -1.35056 | 1.89E-10 | 3.73E-06 | DOWN |
| ENSG00000138398 | PPIG | -1.35075 | 1.24E-09 | 1.09E-05 | DOWN |
| ENSG00000128710 | HOXD10 | -1.35204 | 9.77E-07 | 0.000476 | DOWN |
| ENSG00000197050 | ZNF420 | -1.3535 | 1.90E-09 | 1.40E-05 | DOWN |
| ENSG00000121749 | TBC1D15 | -1.35619 | 7.53E-13 | 1.61E-07 | DOWN |
| ENSG00000100744 | GSKIP | -1.35739 | 7.80E-11 | 2.23E-06 | DOWN |
| ENSG00000129810 | SGOL1 | -1.3574 | 2.37E-08 | 5.79E-05 | DOWN |
| ENSG00000185689 | C6orf201 | -1.35749 | 5.10E-10 | 6.54E-06 | DOWN |
| ENSG00000102384 | CENPI | -1.36094 | 1.42E-08 | 4.28E-05 | DOWN |
| ENSG00000129515 | SNX6 | -1.3627 | 2.92E-18 | 1.29E-10 | DOWN |
| ENSG00000274641 | HIST1H2BO | -1.36271 | 2.47E-10 | 4.29E-06 | DOWN |
| ENSG00000064989 | CALCRL | -1.36495 | 5.24E-05 | 0.004564 | DOWN |
| ENSG00000149089 | APIP | -1.36573 | 7.25E-12 | 5.76E-07 | DOWN |
| ENSG00000139324 | TMTC3 | -1.36644 | 6.95E-12 | 5.63E-07 | DOWN |
| ENSG00000127720 | METTL25 | -1.36887 | 6.91E-18 | 2.12E-10 | DOWN |
| ENSG00000135111 | TBX3 | -1.37095 | 2.19E-08 | 5.54E-05 | DOWN |
| ENSG00000279131 | AC069063.1 | -1.37193 | 0.003111 | 0.045193 | DOWN |
| ENSG00000141446 | ESCO1 | -1.37235 | 1.92E-13 | 7.70E-08 | DOWN |
| ENSG00000117533 | VAMP4 | -1.37355 | 3.36E-08 | 7.04E-05 | DOWN |
| ENSG00000160208 | RRP1B | -1.37487 | 2.31E-07 | 0.000211 | DOWN |
| ENSG00000004897 | CDC27 | -1.37493 | 1.54E-06 | 0.000617 | DOWN |
| ENSG00000112333 | NR2E1 | -1.37562 | 2.29E-09 | 1.55E-05 | DOWN |
| ENSG00000115607 | IL18RAP | -1.37563 | 1.10E-05 | 0.001895 | DOWN |
| ENSG00000123552 | USP45 | -1.37732 | 7.73E-09 | 3.04E-05 | DOWN |
| ENSG00000105520 | PLPPR2 | -1.37856 | 1.36E-07 | 0.000155 | DOWN |
| ENSG00000116473 | RAP1A | -1.38143 | 1.62E-14 | 1.93E-08 | DOWN |
| ENSG00000278272 | HIST1H3C | -1.38297 | 1.28E-07 | 0.00015 | DOWN |
| ENSG00000137274 | BPHL | -1.38313 | 8.09E-13 | 1.66E-07 | DOWN |
| ENSG00000140030 | GPR65 | -1.38318 | 8.56E-08 | 0.00012 | DOWN |
| ENSG00000132432 | SEC61G | -1.38374 | 3.44E-12 | 3.78E-07 | DOWN |
| ENSG00000204116 | CHIC1 | -1.38636 | 1.70E-05 | 0.002421 | DOWN |
| ENSG00000108039 | XPNPEP1 | -1.38754 | 0.001002 | 0.023996 | DOWN |
| ENSG00000175893 | ZDHHC21 | -1.38903 | 9.10E-10 | 9.20E-06 | DOWN |
| ENSG00000166128 | RAB8B | -1.39078 | 5.03E-15 | 9.49E-09 | DOWN |
| ENSG00000134057 | CCNB1 | -1.39183 | 1.08E-11 | 7.11E-07 | DOWN |
| ENSG00000234545 | FAM133B | -1.39191 | 6.20E-09 | 2.68E-05 | DOWN |
| ENSG00000221977 | OR4E2 | -1.39495 | 2.28E-13 | 8.49E-08 | DOWN |
| ENSG00000128708 | HAT1 | -1.3954 | 1.62E-08 | 4.63E-05 | DOWN |
| ENSG00000198482 | ZNF808 | -1.39634 | 0.000997 | 0.023941 | DOWN |
| ENSG00000164398 | ACSL6 | -1.39648 | 6.71E-05 | 0.005248 | DOWN |
| ENSG00000142875 | PRKACB | -1.39742 | 4.29E-07 | 0.000299 | DOWN |
| ENSG00000162374 | ELAVL4 | -1.39768 | 0.000177 | 0.009121 | DOWN |
| ENSG00000112175 | BMP5 | -1.39819 | 7.75E-07 | 0.000417 | DOWN |
| ENSG00000166012 | TAF1D | -1.39898 | 2.96E-12 | 3.49E-07 | DOWN |
| ENSG00000167524 | SGK494 | -1.39985 | 8.00E-07 | 0.000424 | DOWN |
| ENSG00000139734 | DIAPH3 | -1.3999 | 2.45E-08 | 5.89E-05 | DOWN |
| ENSG00000163832 | ELP6 | -1.40076 | 8.46E-17 | 8.65E-10 | DOWN |
| ENSG00000143369 | ECM1 | -1.4008 | 0.000639 | 0.018699 | DOWN |
| ENSG00000124207 | CSE1L | -1.40146 | 1.39E-14 | 1.77E-08 | DOWN |
| ENSG00000185379 | RAD51D | -1.40201 | 7.09E-05 | 0.00542 | DOWN |
| ENSG00000047597 | XK | -1.40278 | 0.000777 | 0.020885 | DOWN |
| ENSG00000150456 | N6AMT2 | -1.40312 | 6.77E-07 | 0.000387 | DOWN |
| ENSG00000105835 | NAMPT | -1.40693 | 4.28E-09 | 2.19E-05 | DOWN |
| ENSG00000123219 | CENPK | -1.40714 | 4.88E-07 | 0.000321 | DOWN |
| ENSG00000148926 | ADM | -1.40726 | 8.42E-11 | 2.33E-06 | DOWN |
| ENSG00000254685 | FPGT | -1.40799 | 6.59E-12 | 5.47E-07 | DOWN |
| ENSG00000169255 | B3GALNT1 | -1.40826 | 2.80E-06 | 0.000865 | DOWN |
| ENSG00000006634 | DBF4 | -1.40854 | 9.39E-09 | 3.37E-05 | DOWN |
| ENSG00000178074 | C2orf69 | -1.40913 | 1.95E-09 | 1.41E-05 | DOWN |
| ENSG00000132141 | CCT6B | -1.41009 | 0.001169 | 0.026203 | DOWN |
| ENSG00000077254 | USP33 | -1.4104 | 4.10E-09 | 2.14E-05 | DOWN |
| ENSG00000172995 | ARPP21 | -1.41086 | 1.21E-06 | 0.00054 | DOWN |
| ENSG00000169330 | KIAA1024 | -1.41135 | 5.94E-05 | 0.004896 | DOWN |
| ENSG00000101096 | NFATC2 | -1.41298 | 2.39E-12 | 3.09E-07 | DOWN |
| ENSG00000168283 | BMI1 | -1.41329 | 1.98E-07 | 0.000194 | DOWN |
| ENSG00000197969 | VPS13A | -1.41362 | 4.42E-10 | 6.08E-06 | DOWN |
| ENSG00000138759 | FRAS1 | -1.41549 | 4.03E-10 | 5.74E-06 | DOWN |
| ENSG00000094975 | SUCO | -1.41574 | 3.60E-10 | 5.35E-06 | DOWN |
| ENSG00000163507 | KIAA1524 | -1.41661 | 3.98E-11 | 1.51E-06 | DOWN |
| ENSG00000247746 | USP51 | -1.41763 | 8.24E-14 | 4.79E-08 | DOWN |
| ENSG00000087586 | AURKA | -1.41802 | 7.63E-09 | 3.01E-05 | DOWN |
| ENSG00000145757 | SPATA9 | -1.41951 | 0.000961 | 0.023485 | DOWN |
| ENSG00000145911 | N4BP3 | -1.4197 | 4.12E-07 | 0.000292 | DOWN |
| ENSG00000238074 | TSPY9P | -1.4202 | 6.27E-12 | 5.32E-07 | DOWN |
| ENSG00000155329 | ZCCHC10 | -1.42064 | 2.72E-10 | 4.54E-06 | DOWN |
| ENSG00000106052 | TAX1BP1 | -1.42127 | 4.81E-10 | 6.34E-06 | DOWN |
| ENSG00000168216 | LMBRD1 | -1.42133 | 2.19E-15 | 5.92E-09 | DOWN |
| ENSG00000136982 | DSCC1 | -1.42393 | 1.32E-05 | 0.002093 | DOWN |
| ENSG00000157557 | ETS2 | -1.42477 | 5.35E-11 | 1.80E-06 | DOWN |
| ENSG00000100442 | FKBP3 | -1.42884 | 3.32E-10 | 5.11E-06 | DOWN |
| ENSG00000088325 | TPX2 | -1.43494 | 1.63E-09 | 1.28E-05 | DOWN |
| ENSG00000189430 | NCR1 | -1.43557 | 0.0028 | 0.042574 | DOWN |
| ENSG00000126226 | PCID2 | -1.43739 | 2.70E-08 | 6.19E-05 | DOWN |
| ENSG00000241973 | PI4KA | -1.43926 | 1.91E-12 | 2.69E-07 | DOWN |
| ENSG00000130164 | LDLR | -1.43952 | 1.86E-07 | 0.000187 | DOWN |
| ENSG00000111725 | PRKAB1 | -1.44141 | 0.002687 | 0.041596 | DOWN |
| ENSG00000203965 | EFCAB7 | -1.44153 | 1.80E-06 | 0.000675 | DOWN |
| ENSG00000161249 | DMKN | -1.44307 | 1.41E-05 | 0.002176 | DOWN |
| ENSG00000198938 | MT-CO3 | -1.44315 | 1.55E-06 | 0.000621 | DOWN |
| ENSG00000102057 | KCND1 | -1.44516 | 0.000263 | 0.011385 | DOWN |
| ENSG00000224470 | ATXN1L | -1.44874 | 0.00013 | 0.007648 | DOWN |
| ENSG00000196911 | KPNA5 | -1.44957 | 1.22E-09 | 1.08E-05 | DOWN |
| ENSG00000181562 | EDDM3A | -1.45086 | 2.15E-13 | 8.22E-08 | DOWN |
| ENSG00000163513 | TGFBR2 | -1.45219 | 0.00034 | 0.013144 | DOWN |
| ENSG00000276903 | HIST1H2AL | -1.4531 | 4.79E-05 | 0.004327 | DOWN |
| ENSG00000187372 | PCDHB13 | -1.45416 | 9.09E-08 | 0.000124 | DOWN |
| ENSG00000255221 | CARD17 | -1.45737 | 0.000154 | 0.008428 | DOWN |
| ENSG00000172139 | SLC9C1 | -1.45856 | 0.000308 | 0.012435 | DOWN |
| ENSG00000148481 | FAM188A | -1.46085 | 8.49E-14 | 4.82E-08 | DOWN |
| ENSG00000197134 | ZNF257 | -1.46113 | 0.000101 | 0.006626 | DOWN |
| ENSG00000174485 | DENND4A | -1.46134 | 1.74E-11 | 9.38E-07 | DOWN |
| ENSG00000156136 | DCK | -1.46367 | 1.70E-07 | 0.000177 | DOWN |
| ENSG00000198826 | ARHGAP11A | -1.46489 | 2.28E-07 | 0.00021 | DOWN |
| ENSG00000188549 | C15orf52 | -1.46548 | 2.50E-07 | 0.00022 | DOWN |
| ENSG00000072201 | LNX1 | -1.46638 | 0.002948 | 0.043809 | DOWN |
| ENSG00000180016 | OR1E1 | -1.46651 | 1.72E-09 | 1.32E-05 | DOWN |
| ENSG00000161813 | LARP4 | -1.46686 | 4.04E-15 | 8.37E-09 | DOWN |
| ENSG00000149231 | CCDC82 | -1.4712 | 9.37E-10 | 9.36E-06 | DOWN |
| ENSG00000118971 | CCND2 | -1.47149 | 1.30E-10 | 2.99E-06 | DOWN |
| ENSG00000157827 | FMNL2 | -1.47219 | 9.65E-13 | 1.86E-07 | DOWN |
| ENSG00000185917 | SETD4 | -1.4745 | 2.40E-07 | 0.000215 | DOWN |
| ENSG00000196747 | HIST1H2AI | -1.47487 | 9.00E-08 | 0.000123 | DOWN |
| ENSG00000110330 | BIRC2 | -1.47606 | 2.14E-08 | 5.45E-05 | DOWN |
| ENSG00000111711 | GOLT1B | -1.48012 | 7.86E-14 | 4.71E-08 | DOWN |
| ENSG00000092931 | MFSD11 | -1.48023 | 0.000259 | 0.011272 | DOWN |
| ENSG00000135655 | USP15 | -1.48064 | 1.18E-15 | 4.11E-09 | DOWN |
| ENSG00000109332 | UBE2D3 | -1.48257 | 2.06E-10 | 3.90E-06 | DOWN |
| ENSG00000114346 | ECT2 | -1.48261 | 7.73E-10 | 8.37E-06 | DOWN |
| ENSG00000160691 | SHC1 | -1.48406 | 6.66E-05 | 0.005228 | DOWN |
| ENSG00000262560 | RP11-296A16.1 | -1.48449 | 4.76E-15 | 9.21E-09 | DOWN |
| ENSG00000116750 | UCHL5 | -1.48622 | 5.58E-10 | 6.90E-06 | DOWN |
| ENSG00000116489 | CAPZA1 | -1.48705 | 2.43E-14 | 2.40E-08 | DOWN |
| ENSG00000066739 | ATG2B | -1.488 | 2.08E-11 | 1.04E-06 | DOWN |
| ENSG00000167083 | GNGT2 | -1.48825 | 0.000116 | 0.007199 | DOWN |
| ENSG00000119888 | EPCAM | -1.49132 | 1.46E-11 | 8.50E-07 | DOWN |
| ENSG00000156384 | SFR1 | -1.49255 | 1.66E-09 | 1.29E-05 | DOWN |
| ENSG00000117724 | CENPF | -1.49407 | 2.04E-07 | 0.000197 | DOWN |
| ENSG00000164342 | TLR3 | -1.49429 | 1.41E-07 | 0.000159 | DOWN |
| ENSG00000151414 | NEK7 | -1.49481 | 4.98E-14 | 3.66E-08 | DOWN |
| ENSG00000174226 | SNX31 | -1.49532 | 7.93E-15 | 1.27E-08 | DOWN |
| ENSG00000196240 | OR2T2 | -1.49645 | 3.86E-10 | 5.59E-06 | DOWN |
| ENSG00000205413 | SAMD9 | -1.49737 | 1.10E-11 | 7.18E-07 | DOWN |
| ENSG00000169299 | PGM2 | -1.50006 | 1.08E-10 | 2.68E-06 | DOWN |
| ENSG00000143546 | S100A8 | -1.50205 | 2.42E-05 | 0.002948 | DOWN |
| ENSG00000080824 | HSP90AA1 | -1.50237 | 1.45E-09 | 1.20E-05 | DOWN |
| ENSG00000151725 | CENPU | -1.5048 | 7.91E-08 | 0.000114 | DOWN |
| ENSG00000104219 | ZDHHC2 | -1.5054 | 3.40E-13 | 1.04E-07 | DOWN |
| ENSG00000278637 | HIST1H4A | -1.50548 | 9.31E-18 | 2.51E-10 | DOWN |
| ENSG00000104043 | ATP8B4 | -1.50778 | 1.09E-05 | 0.00189 | DOWN |
| ENSG00000145354 | CISD2 | -1.50802 | 1.22E-08 | 3.92E-05 | DOWN |
| ENSG00000137601 | NEK1 | -1.51104 | 2.31E-17 | 4.25E-10 | DOWN |
| ENSG00000168228 | ZCCHC4 | -1.51194 | 5.52E-08 | 9.29E-05 | DOWN |
| ENSG00000003436 | TFPI | -1.51445 | 0.000114 | 0.007113 | DOWN |
| ENSG00000145949 | MYLK4 | -1.51528 | 0.00016 | 0.008607 | DOWN |
| ENSG00000198160 | MIER1 | -1.51586 | 8.46E-14 | 4.82E-08 | DOWN |
| ENSG00000102468 | HTR2A | -1.51625 | 0.000277 | 0.01171 | DOWN |
| ENSG00000110721 | CHKA | -1.51675 | 1.98E-17 | 3.89E-10 | DOWN |
| ENSG00000145623 | OSMR | -1.51713 | 0.002165 | 0.036823 | DOWN |
| ENSG00000213096 | ZNF254 | -1.51727 | 2.92E-10 | 4.74E-06 | DOWN |
| ENSG00000173679 | OR1L1 | -1.52064 | 5.14E-07 | 0.00033 | DOWN |
| ENSG00000111276 | CDKN1B | -1.52195 | 3.13E-13 | 1.01E-07 | DOWN |
| ENSG00000047621 | C12orf4 | -1.52229 | 2.10E-15 | 5.80E-09 | DOWN |
| ENSG00000158815 | FGF17 | -1.52267 | 2.85E-13 | 9.58E-08 | DOWN |
| ENSG00000110958 | PTGES3 | -1.52292 | 2.48E-06 | 0.000808 | DOWN |
| ENSG00000124333 | VAMP7 | -1.52319 | 3.51E-08 | 7.22E-05 | DOWN |
| ENSG00000196381 | ZNF781 | -1.52393 | 1.52E-05 | 0.002269 | DOWN |
| ENSG00000155542 | SETD9 | -1.52435 | 0.001277 | 0.027472 | DOWN |
| ENSG00000173674 | EIF1AX | -1.52551 | 9.74E-09 | 3.45E-05 | DOWN |
| ENSG00000072274 | TFRC | -1.52561 | 1.43E-08 | 4.30E-05 | DOWN |
| ENSG00000100479 | POLE2 | -1.52663 | 3.95E-08 | 7.67E-05 | DOWN |
| ENSG00000108771 | DHX58 | -1.52797 | 0.000546 | 0.017165 | DOWN |
| ENSG00000115507 | OTX1 | -1.52843 | 0.001827 | 0.033555 | DOWN |
| ENSG00000182359 | KBTBD3 | -1.53002 | 4.09E-08 | 7.83E-05 | DOWN |
| ENSG00000189043 | NDUFA4 | -1.53085 | 4.48E-13 | 1.21E-07 | DOWN |
| ENSG00000186063 | AIDA | -1.53203 | 1.29E-07 | 0.000151 | DOWN |
| ENSG00000143158 | MPC2 | -1.53322 | 2.36E-08 | 5.77E-05 | DOWN |
| ENSG00000101187 | SLCO4A1 | -1.53407 | 1.74E-09 | 1.33E-05 | DOWN |
| ENSG00000145242 | EPHA5 | -1.53681 | 3.13E-17 | 4.99E-10 | DOWN |
| ENSG00000164611 | PTTG1 | -1.54066 | 4.01E-09 | 2.11E-05 | DOWN |
| ENSG00000135744 | AGT | -1.54255 | 1.25E-09 | 1.09E-05 | DOWN |
| ENSG00000155090 | KLF10 | -1.54414 | 4.09E-09 | 2.14E-05 | DOWN |
| ENSG00000176208 | ATAD5 | -1.5477 | 2.89E-09 | 1.76E-05 | DOWN |
| ENSG00000124783 | SSR1 | -1.54983 | 6.22E-11 | 1.95E-06 | DOWN |
| ENSG00000073282 | TP63 | -1.55305 | 3.79E-15 | 8.09E-09 | DOWN |
| ENSG00000147604 | RPL7 | -1.55524 | 1.81E-07 | 0.000185 | DOWN |
| ENSG00000118520 | ARG1 | -1.55675 | 8.30E-08 | 0.000118 | DOWN |
| ENSG00000153140 | CETN3 | -1.55855 | 8.54E-11 | 2.35E-06 | DOWN |
| ENSG00000146350 | TBC1D32 | -1.55868 | 4.94E-10 | 6.43E-06 | DOWN |
| ENSG00000105374 | NKG7 | -1.56285 | 7.59E-06 | 0.001527 | DOWN |
| ENSG00000111231 | GPN3 | -1.56307 | 9.38E-13 | 1.83E-07 | DOWN |
| ENSG00000280671 | AC112719.2 | -1.56313 | 2.21E-12 | 2.95E-07 | DOWN |
| ENSG00000166435 | XRRA1 | -1.56326 | 2.55E-07 | 0.000222 | DOWN |
| ENSG00000136492 | BRIP1 | -1.56491 | 5.82E-13 | 1.40E-07 | DOWN |
| ENSG00000165996 | HACD1 | -1.56575 | 0.000595 | 0.01802 | DOWN |
| ENSG00000166483 | WEE1 | -1.56689 | 5.48E-18 | 1.85E-10 | DOWN |
| ENSG00000142856 | ITGB3BP | -1.56754 | 1.45E-12 | 2.33E-07 | DOWN |
| ENSG00000198836 | OPA1 | -1.57 | 3.80E-05 | 0.003796 | DOWN |
| ENSG00000135413 | LACRT | -1.57125 | 1.44E-13 | 6.55E-08 | DOWN |
| ENSG00000124802 | EEF1E1 | -1.57373 | 0.000105 | 0.006783 | DOWN |
| ENSG00000180425 | C11orf71 | -1.57791 | 6.26E-08 | 0.0001 | DOWN |
| ENSG00000164068 | RNF123 | -1.57941 | 8.79E-07 | 0.000448 | DOWN |
| ENSG00000170385 | SLC30A1 | -1.58014 | 4.13E-12 | 4.20E-07 | DOWN |
| ENSG00000156976 | EIF4A2 | -1.58557 | 1.10E-07 | 0.000138 | DOWN |
| ENSG00000187837 | HIST1H1C | -1.5897 | 1.17E-15 | 4.11E-09 | DOWN |
| ENSG00000162669 | HFM1 | -1.59099 | 8.05E-05 | 0.005836 | DOWN |
| ENSG00000135315 | CEP162 | -1.59644 | 1.71E-14 | 1.98E-08 | DOWN |
| ENSG00000183137 | CEP57L1 | -1.59668 | 2.77E-10 | 4.59E-06 | DOWN |
| ENSG00000113249 | HAVCR1 | -1.59669 | 1.13E-05 | 0.001925 | DOWN |
| ENSG00000184163 | FAM132A | -1.5973 | 6.48E-09 | 2.75E-05 | DOWN |
| ENSG00000142089 | IFITM3 | -1.59876 | 6.80E-08 | 0.000105 | DOWN |
| ENSG00000198712 | MT-CO2 | -1.59973 | 2.34E-06 | 0.00078 | DOWN |
| ENSG00000197019 | SERTAD1 | -1.60249 | 1.01E-09 | 9.77E-06 | DOWN |
| ENSG00000135452 | TSPAN31 | -1.60448 | 0.001265 | 0.027361 | DOWN |
| ENSG00000071794 | HLTF | -1.61069 | 7.11E-16 | 3.04E-09 | DOWN |
| ENSG00000144320 | KIAA1715 | -1.61118 | 2.83E-13 | 9.56E-08 | DOWN |
| ENSG00000023041 | ZDHHC6 | -1.6119 | 0.000367 | 0.01371 | DOWN |
| ENSG00000204131 | NHSL2 | -1.61338 | 1.36E-07 | 0.000155 | DOWN |
| ENSG00000204179 | PTPN20 | -1.61563 | 7.76E-08 | 0.000113 | DOWN |
| ENSG00000120256 | LRP11 | -1.61685 | 1.09E-07 | 0.000138 | DOWN |
| ENSG00000159307 | SCUBE1 | -1.62 | 4.31E-07 | 0.000299 | DOWN |
| ENSG00000137145 | DENND4C | -1.62026 | 4.79E-08 | 8.56E-05 | DOWN |
| ENSG00000170522 | ELOVL6 | -1.62065 | 3.33E-05 | 0.003522 | DOWN |
| ENSG00000164221 | CCDC112 | -1.62252 | 2.66E-06 | 0.00084 | DOWN |
| ENSG00000118496 | FBXO30 | -1.62414 | 9.40E-09 | 3.37E-05 | DOWN |
| ENSG00000273703 | HIST1H2BM | -1.62641 | 1.08E-14 | 1.52E-08 | DOWN |
| ENSG00000071082 | RPL31 | -1.62735 | 2.16E-07 | 0.000203 | DOWN |
| ENSG00000235608 | NKX1-1 | -1.63123 | 1.80E-14 | 2.03E-08 | DOWN |
| ENSG00000116830 | TTF2 | -1.63281 | 1.68E-10 | 3.47E-06 | DOWN |
| ENSG00000018408 | WWTR1 | -1.63635 | 1.54E-10 | 3.28E-06 | DOWN |
| ENSG00000125249 | RAP2A | -1.63919 | 2.72E-08 | 6.21E-05 | DOWN |
| ENSG00000225830 | ERCC6 | -1.64 | 3.26E-12 | 3.68E-07 | DOWN |
| ENSG00000187583 | PLEKHN1 | -1.64073 | 2.82E-09 | 1.74E-05 | DOWN |
| ENSG00000117155 | SSX2IP | -1.64228 | 1.94E-16 | 1.42E-09 | DOWN |
| ENSG00000091009 | RBM27 | -1.64232 | 2.79E-11 | 1.23E-06 | DOWN |
| ENSG00000138346 | DNA2 | -1.64313 | 2.37E-07 | 0.000213 | DOWN |
| ENSG00000152778 | IFIT5 | -1.64516 | 4.58E-05 | 0.004215 | DOWN |
| ENSG00000172209 | GPR22 | -1.64524 | 1.33E-07 | 0.000154 | DOWN |
| ENSG00000115604 | IL18R1 | -1.64647 | 0.000286 | 0.011936 | DOWN |
| ENSG00000123892 | RAB38 | -1.64975 | 0.000431 | 0.015028 | DOWN |
| ENSG00000170647 | TMEM133 | -1.65468 | 7.81E-05 | 0.005728 | DOWN |
| ENSG00000149269 | PAK1 | -1.65489 | 1.47E-10 | 3.20E-06 | DOWN |
| ENSG00000176155 | CCDC57 | -1.65544 | 2.67E-17 | 4.62E-10 | DOWN |
| ENSG00000109881 | CCDC34 | -1.65838 | 1.93E-06 | 0.000701 | DOWN |
| ENSG00000196074 | SYCP2 | -1.66205 | 4.40E-13 | 1.20E-07 | DOWN |
| ENSG00000104267 | CA2 | -1.66425 | 0.003274 | 0.046445 | DOWN |
| ENSG00000163577 | EIF5A2 | -1.66956 | 8.69E-10 | 8.95E-06 | DOWN |
| ENSG00000205542 | TMSB4X | -1.67054 | 6.18E-17 | 7.28E-10 | DOWN |
| ENSG00000114209 | PDCD10 | -1.67134 | 6.70E-10 | 7.75E-06 | DOWN |
| ENSG00000139354 | GAS2L3 | -1.67162 | 2.23E-10 | 4.06E-06 | DOWN |
| ENSG00000197045 | GMFB | -1.67233 | 2.46E-18 | 1.17E-10 | DOWN |
| ENSG00000107796 | ACTA2 | -1.67486 | 2.09E-08 | 5.38E-05 | DOWN |
| ENSG00000232112 | TMA7 | -1.67577 | 1.52E-11 | 8.67E-07 | DOWN |
| ENSG00000175449 | RFESD | -1.67828 | 1.81E-11 | 9.56E-07 | DOWN |
| ENSG00000162976 | PQLC3 | -1.67862 | 1.96E-14 | 2.12E-08 | DOWN |
| ENSG00000075089 | ACTR6 | -1.68551 | 6.47E-13 | 1.48E-07 | DOWN |
| ENSG00000077152 | UBE2T | -1.68861 | 2.81E-07 | 0.000234 | DOWN |
| ENSG00000146007 | ZMAT2 | -1.68915 | 1.20E-05 | 0.001989 | DOWN |
| ENSG00000163564 | PYHIN1 | -1.69168 | 7.01E-06 | 0.001457 | DOWN |
| ENSG00000170540 | ARL6IP1 | -1.69238 | 2.42E-10 | 4.25E-06 | DOWN |
| ENSG00000175105 | ZNF654 | -1.69548 | 2.00E-11 | 1.01E-06 | DOWN |
| ENSG00000164120 | HPGD | -1.69614 | 2.29E-14 | 2.32E-08 | DOWN |
| ENSG00000151773 | CCDC122 | -1.69906 | 2.88E-05 | 0.003251 | DOWN |
| ENSG00000133641 | C12orf29 | -1.70249 | 2.35E-18 | 1.15E-10 | DOWN |
| ENSG00000165097 | KDM1B | -1.70417 | 2.31E-13 | 8.53E-08 | DOWN |
| ENSG00000104522 | TSTA3 | -1.70534 | 5.34E-06 | 0.001246 | DOWN |
| ENSG00000168229 | PTGDR | -1.70735 | 0.000497 | 0.016258 | DOWN |
| ENSG00000160868 | CYP3A4 | -1.70747 | 4.73E-05 | 0.004296 | DOWN |
| ENSG00000156876 | SASS6 | -1.70799 | 6.48E-13 | 1.48E-07 | DOWN |
| ENSG00000145723 | GIN1 | -1.71573 | 1.40E-13 | 6.44E-08 | DOWN |
| ENSG00000105894 | PTN | -1.71613 | 1.15E-16 | 1.03E-09 | DOWN |
| ENSG00000101888 | NXT2 | -1.71632 | 4.70E-10 | 6.28E-06 | DOWN |
| ENSG00000177694 | NAALADL2 | -1.72885 | 2.15E-05 | 0.002759 | DOWN |
| ENSG00000011426 | ANLN | -1.73158 | 5.71E-09 | 2.55E-05 | DOWN |
| ENSG00000138061 | CYP1B1 | -1.73244 | 0.000303 | 0.012315 | DOWN |
| ENSG00000163114 | PDHA2 | -1.73738 | 7.73E-16 | 3.16E-09 | DOWN |
| ENSG00000182944 | EWSR1 | -1.74203 | 0.000346 | 0.01328 | DOWN |
| ENSG00000134758 | RNF138 | -1.74948 | 5.26E-12 | 4.86E-07 | DOWN |
| ENSG00000270276 | HIST2H4B | -1.7541 | 0.001245 | 0.027112 | DOWN |
| ENSG00000138411 | HECW2 | -1.75424 | 3.53E-07 | 0.000267 | DOWN |
| ENSG00000183542 | KLRC4 | -1.75487 | 0.000595 | 0.018011 | DOWN |
| ENSG00000169583 | CLIC3 | -1.75574 | 0.000323 | 0.012772 | DOWN |
| ENSG00000196151 | WDSUB1 | -1.76124 | 9.32E-11 | 2.48E-06 | DOWN |
| ENSG00000168209 | DDIT4 | -1.76225 | 3.71E-05 | 0.003749 | DOWN |
| ENSG00000173064 | HECTD4 | -1.76602 | 9.73E-11 | 2.53E-06 | DOWN |
| ENSG00000080293 | SCTR | -1.76864 | 0.002673 | 0.041468 | DOWN |
| ENSG00000269433 | OPN1MW3 | -1.77215 | 3.19E-06 | 0.000932 | DOWN |
| ENSG00000100346 | CACNA1I | -1.7757 | 1.08E-09 | 1.01E-05 | DOWN |
| ENSG00000181061 | HIGD1A | -1.78107 | 8.82E-09 | 3.26E-05 | DOWN |
| ENSG00000185122 | HSF1 | -1.78466 | 3.15E-09 | 1.85E-05 | DOWN |
| ENSG00000129988 | LBP | -1.78505 | 9.41E-07 | 0.000466 | DOWN |
| ENSG00000100227 | POLDIP3 | -1.78876 | 4.78E-10 | 6.32E-06 | DOWN |
| ENSG00000126467 | TSKS | -1.79826 | 5.69E-12 | 5.06E-07 | DOWN |
| ENSG00000144485 | HES6 | -1.79899 | 1.76E-05 | 0.002461 | DOWN |
| ENSG00000058085 | LAMC2 | -1.80392 | 9.63E-15 | 1.43E-08 | DOWN |
| ENSG00000169439 | SDC2 | -1.8146 | 6.69E-05 | 0.005241 | DOWN |
| ENSG00000184357 | HIST1H1B | -1.81475 | 3.03E-07 | 0.000245 | DOWN |
| ENSG00000148019 | CEP78 | -1.81606 | 6.23E-16 | 2.86E-09 | DOWN |
| ENSG00000187753 | C9orf153 | -1.81689 | 2.43E-10 | 4.26E-06 | DOWN |
| ENSG00000138326 | RPS24 | -1.82034 | 3.26E-13 | 1.02E-07 | DOWN |
| ENSG00000183439 | TRIM61 | -1.82658 | 4.20E-07 | 0.000295 | DOWN |
| ENSG00000146469 | VIP | -1.82915 | 2.34E-09 | 1.57E-05 | DOWN |
| ENSG00000104722 | NEFM | -1.84017 | 5.51E-13 | 1.37E-07 | DOWN |
| ENSG00000171951 | SCG2 | -1.8414 | 7.51E-12 | 5.85E-07 | DOWN |
| ENSG00000172382 | PRSS27 | -1.85248 | 0.00092 | 0.022917 | DOWN |
| ENSG00000186105 | LRRC70 | -1.85426 | 8.56E-06 | 0.001641 | DOWN |
| ENSG00000163634 | THOC7 | -1.85647 | 8.31E-14 | 4.80E-08 | DOWN |
| ENSG00000278463 | HIST1H2AB | -1.85712 | 4.34E-12 | 4.33E-07 | DOWN |
| ENSG00000152266 | PTH | -1.86741 | 4.13E-06 | 0.001077 | DOWN |
| ENSG00000152102 | FAM168B | -1.86743 | 0.000762 | 0.020631 | DOWN |
| ENSG00000135862 | LAMC1 | -1.86952 | 2.63E-08 | 6.10E-05 | DOWN |
| ENSG00000169139 | UBE2V2 | -1.87026 | 4.82E-16 | 2.48E-09 | DOWN |
| ENSG00000165240 | ATP7A | -1.87466 | 1.35E-06 | 0.000573 | DOWN |
| ENSG00000114854 | TNNC1 | -1.877 | 3.37E-12 | 3.74E-07 | DOWN |
| ENSG00000183662 | FAM19A1 | -1.88298 | 0.000464 | 0.015632 | DOWN |
| ENSG00000132541 | HRSP12 | -1.88657 | 5.24E-13 | 1.33E-07 | DOWN |
| ENSG00000128609 | NDUFA5 | -1.89887 | 3.53E-18 | 1.43E-10 | DOWN |
| ENSG00000145386 | CCNA2 | -1.90333 | 2.07E-10 | 3.91E-06 | DOWN |
| ENSG00000066279 | ASPM | -1.90517 | 1.02E-12 | 1.93E-07 | DOWN |
| ENSG00000188582 | PAQR9 | -1.90981 | 3.36E-11 | 1.37E-06 | DOWN |
| ENSG00000132855 | ANGPTL3 | -1.91332 | 3.22E-05 | 0.003459 | DOWN |
| ENSG00000181409 | AATK | -1.91769 | 2.52E-11 | 1.16E-06 | DOWN |
| ENSG00000122483 | CCDC18 | -1.93477 | 1.04E-08 | 3.57E-05 | DOWN |
| ENSG00000172156 | CCL11 | -1.93999 | 2.09E-06 | 0.000732 | DOWN |
| ENSG00000168589 | DYNLRB2 | -1.94647 | 2.56E-08 | 6.03E-05 | DOWN |
| ENSG00000109805 | NCAPG | -1.96331 | 7.54E-14 | 4.63E-08 | DOWN |
| ENSG00000154839 | SKA1 | -1.96424 | 1.96E-06 | 0.000706 | DOWN |
| ENSG00000166797 | FAM96A | -1.96746 | 3.89E-12 | 4.07E-07 | DOWN |
| ENSG00000153574 | RPIA | -1.97388 | 5.67E-17 | 7.04E-10 | DOWN |
| ENSG00000067606 | PRKCZ | -1.97487 | 1.15E-12 | 2.06E-07 | DOWN |
| ENSG00000146757 | ZNF92 | -1.97509 | 4.96E-11 | 1.71E-06 | DOWN |
| ENSG00000119782 | FKBP1B | -1.97796 | 3.15E-13 | 1.01E-07 | DOWN |
| ENSG00000145736 | GTF2H2 | -1.98036 | 2.77E-13 | 9.51E-08 | DOWN |
| ENSG00000114698 | PLSCR4 | -1.98739 | 1.48E-06 | 0.000606 | DOWN |
| ENSG00000196839 | ADA | -2.00736 | 1.08E-14 | 1.52E-08 | DOWN |
| ENSG00000019582 | CD74 | -2.00969 | 2.95E-09 | 1.78E-05 | DOWN |
| ENSG00000151575 | TEX9 | -2.00979 | 1.83E-09 | 1.36E-05 | DOWN |
| ENSG00000171016 | PYGO1 | -2.01334 | 6.37E-05 | 0.005103 | DOWN |
| ENSG00000006210 | CX3CL1 | -2.01592 | 1.14E-06 | 0.000522 | DOWN |
| ENSG00000163217 | BMP10 | -2.01653 | 0.00015 | 0.00829 | DOWN |
| ENSG00000137563 | GGH | -2.01704 | 3.46E-07 | 0.000265 | DOWN |
| ENSG00000021645 | NRXN3 | -2.01863 | 0.000328 | 0.01289 | DOWN |
| ENSG00000110848 | CD69 | -2.02252 | 3.56E-06 | 0.000993 | DOWN |
| ENSG00000243317 | C7orf73 | -2.02583 | 4.05E-13 | 1.15E-07 | DOWN |
| ENSG00000100335 | MIEF1 | -2.03245 | 2.18E-11 | 1.06E-06 | DOWN |
| ENSG00000115414 | FN1 | -2.03801 | 0.000712 | 0.019849 | DOWN |
| ENSG00000126787 | DLGAP5 | -2.04416 | 6.66E-11 | 2.04E-06 | DOWN |
| ENSG00000038945 | MSR1 | -2.0525 | 1.71E-08 | 4.78E-05 | DOWN |
| ENSG00000170312 | CDK1 | -2.05996 | 1.11E-07 | 0.000139 | DOWN |
| ENSG00000170180 | GYPA | -2.08532 | 7.87E-12 | 5.98E-07 | DOWN |
| ENSG00000187193 | MT1X | -2.09659 | 3.80E-05 | 0.003794 | DOWN |
| ENSG00000186787 | SPIN2B | -2.1064 | 2.85E-06 | 0.000875 | DOWN |
| ENSG00000112077 | RHAG | -2.11212 | 9.85E-12 | 6.79E-07 | DOWN |
| ENSG00000196576 | PLXNB2 | -2.13467 | 1.17E-06 | 0.000527 | DOWN |
| ENSG00000144366 | GULP1 | -2.15004 | 3.07E-07 | 0.000246 | DOWN |
| ENSG00000133739 | LRRCC1 | -2.15227 | 1.79E-09 | 1.35E-05 | DOWN |
| ENSG00000119636 | BBOF1 | -2.15426 | 1.05E-14 | 1.51E-08 | DOWN |
| ENSG00000198286 | CARD11 | -2.16863 | 5.21E-05 | 0.004551 | DOWN |
| ENSG00000163558 | PRKCI | -2.19236 | 1.19E-08 | 3.86E-05 | DOWN |
| ENSG00000163535 | SGOL2 | -2.19741 | 4.80E-09 | 2.31E-05 | DOWN |
| ENSG00000188643 | S100A16 | -2.19765 | 2.25E-07 | 0.000208 | DOWN |
| ENSG00000019169 | MARCO | -2.20048 | 1.16E-09 | 1.05E-05 | DOWN |
| ENSG00000120327 | PCDHB14 | -2.20628 | 3.59E-07 | 0.000269 | DOWN |
| ENSG00000180992 | MRPL14 | -2.2148 | 1.24E-16 | 1.07E-09 | DOWN |
| ENSG00000172543 | CTSW | -2.26018 | 5.74E-08 | 9.52E-05 | DOWN |
| ENSG00000109171 | SLAIN2 | -2.26276 | 0.000195 | 0.009635 | DOWN |
| ENSG00000165895 | ARHGAP42 | -2.26958 | 2.63E-13 | 9.22E-08 | DOWN |
| ENSG00000274744 | TCEB3CL2 | -2.28062 | 2.72E-12 | 3.33E-07 | DOWN |
| ENSG00000150687 | PRSS23 | -2.28581 | 0.003482 | 0.048142 | DOWN |
| ENSG00000138778 | CENPE | -2.28825 | 1.56E-14 | 1.89E-08 | DOWN |
| ENSG00000171320 | ESCO2 | -2.31564 | 5.49E-15 | 9.99E-09 | DOWN |
| ENSG00000168653 | NDUFS5 | -2.32546 | 1.16E-05 | 0.001946 | DOWN |
| ENSG00000117650 | NEK2 | -2.33541 | 2.23E-09 | 1.53E-05 | DOWN |
| ENSG00000125148 | MT2A | -2.35046 | 0.003045 | 0.044617 | DOWN |
| ENSG00000173110 | HSPA6 | -2.37389 | 2.05E-08 | 5.32E-05 | DOWN |
| ENSG00000067191 | CACNB1 | -2.37755 | 9.29E-05 | 0.006339 | DOWN |
| ENSG00000117519 | CNN3 | -2.4512 | 3.38E-13 | 1.04E-07 | DOWN |
| ENSG00000179002 | TAS1R2 | -2.45131 | 1.15E-10 | 2.79E-06 | DOWN |
| ENSG00000111339 | ART4 | -2.47062 | 2.22E-05 | 0.002809 | DOWN |
| ENSG00000167618 | LAIR2 | -2.48538 | 3.77E-06 | 0.001023 | DOWN |
| ENSG00000102230 | PCYT1B | -2.48941 | 0.003175 | 0.045683 | DOWN |
| ENSG00000145349 | CAMK2D | -2.50696 | 0.000361 | 0.0136 | DOWN |
| ENSG00000244482 | LILRA6 | -2.55263 | 1.97E-07 | 0.000193 | DOWN |
| ENSG00000203812 | HIST2H2AA3 | -2.55542 | 6.39E-06 | 0.001383 | DOWN |
| ENSG00000072571 | HMMR | -2.58337 | 1.00E-16 | 9.59E-10 | DOWN |
| ENSG00000198538 | ZNF28 | -2.60571 | 0.001539 | 0.030515 | DOWN |
| ENSG00000095739 | BAMBI | -2.69043 | 3.70E-05 | 0.003744 | DOWN |
| ENSG00000052802 | MSMO1 | -2.69055 | 3.97E-11 | 1.51E-06 | DOWN |
| ENSG00000134352 | IL6ST | -2.71418 | 5.58E-11 | 1.84E-06 | DOWN |
| ENSG00000276368 | HIST1H2AJ | -2.76502 | 8.82E-12 | 6.35E-07 | DOWN |
| ENSG00000180353 | HCLS1 | -2.76647 | 1.77E-07 | 0.000182 | DOWN |
| ENSG00000091490 | SEL1L3 | -2.81173 | 4.16E-10 | 5.85E-06 | DOWN |
| ENSG00000170142 | UBE2E1 | -2.83667 | 0.000757 | 0.020559 | DOWN |
| ENSG00000178752 | FAM132B | -2.87368 | 7.92E-12 | 5.99E-07 | DOWN |
| ENSG00000137441 | FGFBP2 | -2.91996 | 1.47E-06 | 0.000602 | DOWN |
| ENSG00000024526 | DEPDC1 | -2.994 | 3.61E-15 | 7.88E-09 | DOWN |
| ENSG00000153002 | CPB1 | -3.06634 | 2.39E-15 | 6.22E-09 | DOWN |
| ENSG00000104723 | TUSC3 | -3.10754 | 0.000264 | 0.011404 | DOWN |
| ENSG00000147689 | FAM83A | -3.29512 | 2.11E-05 | 0.002726 | DOWN |
| ENSG00000077092 | RARB | -3.31008 | 4.58E-05 | 0.004215 | DOWN |
| ENSG00000165949 | IFI27 | -3.57559 | 0.001264 | 0.027343 | DOWN |
| ENSG00000168269 | FOXI1 | -3.61015 | 0.000125 | 0.007464 | DOWN |
